# Maternal education and its influence on child growth and nutritional status during the first two years of life: a systematic review and meta-analysis

**DOI:** 10.1016/j.eclinm.2024.102574

**Published:** 2024-04-04

**Authors:** Golnaz Rezaeizadeh, Mohammad Ali Mansournia, Abbasali Keshtkar, Zahra Farahani, Fatemeh Zarepour, Maryam Sharafkhah, Roya Kelishadi, Hossein Poustchi

**Affiliations:** a Digestive Diseases Research Center, Digestive Diseases Research Institute, Tehran University of Medical Sciences, Tehran, Iran; b Department of Epidemiology and Biostatistics, School of Public Health, Tehran University of Medical Sciences, Tehran, Iran; c Department of Disaster and Emergency Health, School of Public Health, Tehran University of Medical Sciences, Tehran, Iran; d Maternal, Fetal, and Neonatal Research Center, Family Health Research Institute, Tehran University of Medical Sciences, Tehran, Iran; e School of Medicine, Kashan University of Medical Sciences, Kashan, Iran; f Liver and Pancreatobiliary Diseases Research Center, Digestive Diseases Research Institute, Tehran University of Medical Sciences, Tehran, Iran; g Department of Paediatrics, Child Growth and Development Research Center, Research Institute for Primordial Prevention of Non-communicable Disease, Isfahan University of Medical Sciences, Isfahan, Iran

**Keywords:** Maternal education, Child growth, Country income level, Systematic review, Meta-analysis

## Abstract

**Background:**

The first 1000 days of life are critical for a child's health and development. Impaired growth during this period is linked to increased child morbidity, mortality, and long-term consequences. Undernutrition is the main cause, and addressing it within the first 1000 days of life is vital. Maternal education is consistently identified as a significant predictor of child undernutrition, but its specific impact remains to be determined. This study presents a systematic review and meta-analysis investigating the influence of high versus low maternal education levels on child growth from birth to age two, using population-based cohort studies.

**Methods:**

Databases including PubMed, Scopus, EMBASE, Web of Science, ERIC, and Google Scholar were searched from January 1990 to January 2024 using appropriate search terms. We included population-based cohort studies of healthy children aged two years and under and their mothers, categorizing maternal education levels. Child growth and nutritional outcomes were assessed using various indicators. Two reviewers independently conducted data extraction and assessed study quality. The Newcastle Ottawa scale was utilized for quality assessment. Random-effects models were used for meta-analysis, and heterogeneity was assessed using the Cochrane Q and I^2^ statistic. Subgroup and sensitivity analyses were performed, and publication bias was evaluated.

**Findings:**

The literature search retrieved 14,295 titles, and after full-text screening of 639 reports, 35 studies were included, covering eight outcomes: weight for age z-score (WAZ), height for age z-score (HAZ), BMI for age z-scores (BMIZ), overweight, underweight, stunting, wasting, and rapid weight gain. In middle-income countries, higher maternal education is significantly associated with elevated WAZ (MD 0.398, 95% CI 0.301–0.496) and HAZ (MD 0.388, 95% CI 0.102–0.673) in children. Similarly, in studies with low-educated population, higher maternal education is significantly linked to increased WAZ (MD 0.186, 95% CI 0.078–0.294) and HAZ (0.200, 95% CI 0.036–0.365). However, in high-income and highly educated population, this association is either absent or reversed. In high-income countries, higher maternal education is associated with a non-significant lower BMI-Z (MD −0.028, 95% CI −0.061 to 0.006). Notably, this inverse association is statistically significant in low-educated populations (MD −0.045, 95% CI −0.079 to −0.011) but not in highly educated populations (MD 0.003, 95% CI −0.093 to 0.098).

**Interpretation:**

Maternal education's association with child growth varies based on country income and education levels. Further research is needed to understand this relationship better.

**Funding:**

This study was a student thesis supported financially by 10.13039/501100004484Tehran University of Medical Sciences (TUMS).

**Research in context**

**Evidence before this study**

Early-life growth impairment is associated with increased child morbidity, mortality, and long-term consequences. Undernutrition is the primary cause, resulting in stunting, wasting, underweight, and nutrient deficiencies. Prior to this study, systematic reviews have focused on exploring the social determinants of undernutrition in children. These reviews consistently emphasize the significant role of maternal education in predicting child undernutrition. However, there is still a lack of comprehensive understanding regarding the specific ways in which maternal education influences undernutrition. While one systematic review and meta-analysis has examined the impact of parental education on under-5 mortality, we found no systematic reviews on its association with child growth and nutritional status. This review revealed a clear inverse relationship between parental education, particularly maternal education, and under-5 mortality. However, it is important to note that the authors acknowledged the potential influence of geographical heterogeneity on the overall association between maternal education and child mortality.

**Added value of this study**

This study assessed the influence of maternal educational status on offspring growth through a systematic review of 35 eligible studies. It examined eight growth outcomes, including weight for age z-score (WAZ), height for age z-score (HAZ), BMI for age z-scores (BMIZ), overweight, underweight, stunting, wasting, and rapid weight gain, covering the period from birth to two years of age. The focus was solely on population-based cohort studies.

The findings highlighted that the impact of maternal education on child growth varies depending on the income and education level of the population. In middle-income and/or low-educated populations, high maternal education was positively associated with child growth. However, this positive association was not observed in high-income countries. Instead, there was a notable difference in effect size between studies with high-educated and low-educated participants in these countries.

**Implications of all the available evidence**

Our study highlights the crucial role of maternal education in influencing child growth across diverse income and education settings. It emphasizes the significance of evaluating how maternal education contributes to varied outcomes in child growth, considering the specific characteristics of the population under study.

## Introduction

The concept of developmental origins of health and disease (DOHaD) shows that factors during early life greatly impact the development of diseases later in life. The DOHaD theory introduces a fresh outlook on disease prevention in adulthood. Extensive evidence supports the critical role of the first 1000 days of life, starting from conception and including the pregnancy period, until the child reaches their second birthday. This timeframe is crucial for both the child's overall health and their subsequent development.^1^^,^^2^ Growth impairment during this period has been linked to increased child morbidity and mortality, as well as long-term implications such as diminished intellectual development, short stature, and increased risk of metabolic and cardiovascular diseases. Furthermore, these consequences have significant impacts on future educational attainment, income, and productivity outcomes.^3^^,^^4^

Undernutrition is the primary driver of growth impairment, which encompasses stunting (below-average length/height for age), wasting (below-average weight for length/height), underweight (below-average weight for age), and deficiencies in essential vitamins and minerals.^4^, ^5^, ^6^ Additionally, studies have demonstrated a link between undernutrition and childhood overweight.^7^^,^^8^ The persistent prevalence of undernutrition poses a major public health concern, particularly in low- and middle-income countries.^4^ Extensive research consistently emphasizes that early intervention is crucial for preventing undernutrition, with the most effective strategies being implemented by the age of two.^4^ Consequently, there is a need for thoughtful interventions and policies to address this issue, ensuring the healthy growth and development of children.

The UNICEF causal framework for maternal and child undernutrition identifies three levels of causes. Insufficient dietary intake and diseases are immediate causes, influenced by three underlying causes: food, health, and care. These underlying causes, in turn, are influenced by basic causes, including social, economic, and political factors.^3^^,^^9^ Previous systematic reviews that have examined the social determinants of undernutrition in children have consistently highlighted the significant role of maternal education status as a predictor of child undernutrition.^1^^,^^9^, ^10^, ^11^ However, these studies have not provided a comprehensive understanding of how maternal education affects child undernutrition. Only one systematic review and meta-analysis^12^ that specifically investigated the impact of parental education on under-five mortality revealed a clear inverse dose–response relationship between parental education and child mortality, with maternal education exhibiting a stronger association. Notably, the study also demonstrated that for each additional year of maternal education, the risk of child mortality decreased by 3.04% (95% CI, 2.82%–3.23%). The authors of this study reported that the between-study heterogeneity was due to geographical heterogeneity. Therefore, it appears that a globally dose response association may not provide a clear insight into the association between maternal education and child mortality.

Numerous cross-sectional analyses of Demographic and Health Surveys (DHS) data have explored the relationship between maternal education and child growth. However, it is important to acknowledge the inherent limitations in cross-sectional studies. The primary reason for emphasizing longitudinal studies is to investigate the temporal relationship between maternal education and the growth and nutritional status of under-two children across crucial stages—specifically, at 6, 12, and 24 months. This temporal perspective is essential for understanding evolving patterns, aspects that may pose challenges in cross-sectional studies. The temporal relationship between maternal education and nutritional variables cannot be adequately defined in both longitudinal and cross-sectional studies. Birth cohorts provide a unique opportunity to observe the same group of individuals over time (at 6, 12, and 24 months), facilitating the capture of the dynamic nature of child growth.

In light of these considerations, this systematic review and meta-analysis aim to explore the statistical associations between maternal educational status and the growth of offspring from birth through two years of age (excluding the birth period) by focusing solely on population-based cohort studies. Through a comprehensive examination of longitudinal studies, we aim to uncover insights into how maternal education is statistically associated with the growth and nutritional status of children.

## Methods

This study presents a systematic review and meta-analysis investigating the impact of high (>12 years of schooling) versus low (<9 years of schooling) maternal education levels on child growth from birth to age two, using population-based cohort studies. The threshold values are based on midpoints calculated for the range of years corresponding to the level of education in each study. The study adhered to the PRISMA (Preferred Reporting Items for Systematic Reviews and Meta-Analyses) guidelines for reporting. To ensure transparency and accountability, the protocol for this review was registered with PROSPERO (ID: CRD42022313445).

### Literature search strategy

We searched databases of PubMed, Scopus, EMBASE, Web of Science, ERIC, Google Scholar from 1 January 1990 to 30 January 2024. For electronic search we used MESH terms, free text method and also expert opinion. We used terms including (((maternal OR mother OR parent) AND (“educational status” OR schooling OR literacy OR “socioeconomic status”)) AND ((child OR infant OR under-five OR under-two OR pediatric OR early-life OR offspring) AND (growth OR “nutrition disorder” OR anthropometrics)) AND (“cohort study” OR “longitudinal study” OR “follow-up study” OR “incidence study” OR “prospective study”) AND (1990/1/01:2022/1/31)); The detailed search syntax for each database used in this study is provided in Supplementary File 1 (Supplementary 1).

### Inclusion and exclusion criteria

We included only population-based cohort studies in our analysis. Cross-sectional studies and clinical trials with follow-up were not considered. The study population consisted of healthy children aged two years and under (excluding birth) and their mothers. Mothers of any age, ethnicity, and parity were included. In eligible studies that evaluated the impact of parental educational status on offspring growth, we only included studies that provided separate information for each parent. We included articles that assessed maternal education levels as a categorical variable. In the studies exploring the association between maternal education and child growth up to the age of five, we only included those in the systematic review if they provided data on children under the age of two. Child growth outcomes were assessed using weight-for-age z-score (WAZ), height-for-age z-score (HAZ), BMI-for-age z-score (BMI-Z), as well as indicators such as stunting, wasting, underweight, overweight, and rapid weight gain (RWG).

We excluded studies that lacked sufficient detail on key elements, such as sample size, population, or variables used in adjusted models, due to poor reporting quality. Studies that used maternal education as part of a composite measure rather than an independent variable were also excluded. If only abstracts were available, we attempted to obtain the full articles for review; otherwise, they were excluded. To avoid double counting, if two studies used data from the same source, we only included the study with the larger sample size, adjusted effect size, and those that measured the effect size at multiple time points or had more exposure layers. Studies that treated maternal education as a continuous variable (e.g., years of schooling) or reported a single effect size for all categories were excluded. Studies that only reported the growth trajectory as the outcome or reported weight or height gain with varying time intervals were also excluded from the analysis.

### Screening and selection

The study selection process was conducted in two stages. In the first stage, a screening of titles and abstracts were performed, and studies for which the full text was unavailable after three attempts to contact the authors and journal editor were excluded from our study. In the second stage, the full texts of the remaining studies were independently reviewed by two reviewers (GR and ZF) based on the predetermined inclusion and exclusion criteria. Any discrepancies between the reviewers were resolved through consensus or, if necessary, by involving a third party (RK) for adjudication.

### Risk of bias in studies

Two authors (GR, FZ) independently assessed the studies using the Newcastle Ottawa quality assessment scale^13^ for cohort studies, and any disagreements were resolved through consensus. This scale uses a star system to rate the quality of eight items in three domains: selection (maximum, 4 stars), comparability (maximum, 2 stars), and outcome (maximum, 3 stars). Thresholds for converting the Newcastle–Ottawa scales to the Agency for Healthcare Research and Quality (AHRQ) standards (good, fair and poor) were as follows: Good quality: 3 or 4 stars in the selection domain and 1 or 2 stars in the comparability domain, and 2 or 3 stars in the outcome/exposure domain. Fair quality: 2 stars in selection and 1 or 2 stars in comparability and 2 or 3 stars in outcome/exposure. Poor quality: 0 or 1 star in the selection domain or 0 stars in the comparability domain or 0 or 1 stars in the outcome/exposure domain. All studies were included in the analysis; however, subgroup analyses were conducted based on the quality of the studies.

### Certainty of evidence assessment

A modified version of the Grading of Recommendations Assessment, Development, and Evaluation (GRADE) method, as detailed in Supplementary File 2, was utilized for assessing the certainty of evidence. This method evaluates evidence strength based on three downgrading factors (risk of bias, inconsistency, imprecision) and two upgrading factors (large effects, dose–response effect). Indirectness assessment was excluded from the downgrading factors since all included studies were deemed eligible based on the study question. Additionally, due to varying results in each subgroup, certainty of evidence was assessed individually for each subgroup, precluding the evaluation of publication bias in subgroups with a small number of studies. Opposing bias and confounding were not considered in the upgrading factors, as most studies adjusted the association measure for mediators rather than confounding factors. The dose–response gradients were generated using the restricted cubic spline method and are displayed in Supplementary File 2.

High-quality evidence indicates that additional studies are unlikely to change confidence in the effect size estimate. Moderate-quality evidence suggests that further research could significantly impact and potentially alter the estimate. Low-quality evidence implies that additional research is unlikely to substantially influence the current estimate but may alter it. Very low-quality evidence signifies uncertainty regarding the estimate.

### Data collection process

Data extraction was independently performed by two reviewers (GR and FZ). The following information was extracted from each study: the last name of the first author, publication year, study country (geographic location of sample selection), study country income group, characteristics of the study population (child age and sex), inclusion and exclusion criteria, method of participant recruitment, total population at the start of the study, withdrawals and exclusions, educational groups for comparison along with their definitions, number of individuals in each comparison group, outcome measures (including mean, standard deviation, and 95% confidence interval for continuous outcomes, and odds ratio and 95% confidence interval for categorical outcomes), method of outcome measurement, and statistical methods used in the study. Furthermore, in instances where studies did not present data in tables or the text of the article, but instead relied solely on plots, we utilized the web plot digitizer^14^ tool to extract the data from these plots during the data extraction process. In cases where outcome measures were not reported as z-scores, we converted weight, height, and BMI to WAZ, HAZ, and BMI-Z using WHO child growth standards data. Data availability varied across studies. These calculations have been included in a designated repository at https://github.com/GolnazRezaei80/Maternal-Education-Child-Growth.

The definitions of the eight outcomes^6^ obtained from these studies include.

1. WAZ: A standardized measure that assesses how a child's weight compares to the average weight of children of the same age and sex, based on the WHO, CDC or national growth standards.^15^
2. HAZ: A standardized measure that assesses how a child's height compares to the average height of children of the same age and sex, based on the WHO, CDC or national growth standards.^15^
3. BMI-Z: A standardized measure that assesses how a child's BMI compares to the average BMI of children of the same age and sex, based on the WHO, CDC or national growth standards.^15^
4. Stunting^6^: A condition where a child's height-for-age falls below −2 standard deviations from the median.
5. Wasting^6^: A condition where a child's weight-for-height falls below −2 standard deviations from the median.
6. Underweight^6^^,^^16^: A condition where a child's weight-for-age falls below −2 standard deviations from the median or weight-for-height z-score below the 5th percentile.
7. Overweight (includes obesity): a condition where a child's BMI falls within the range of 25–30. To further classify a child as overweight, their BMI-Z must be equal to or above the 85th percentile, 95th percentile, or +2 standard deviations from the median. Additionally, their weight-for-height z-score should exceed the 97th percentile for their age and sex.^6^^,^^16^
8. RWG: A WAZ change of >0.67 between birth and 6 months or birth and 24 months of age.^17^

The studies were classified into four groups based on the World Bank Group country classifications by income level^18^: low-income, lower-middle-income, upper-middle-income, and high-income. In consideration of the significance of educational attainment, which reflects the percentage of a population who have completed a particular level of education and have a qualification at that level, and given that educational attainment is commonly employed as a measure of human capital and an individual's skills,^19^ an additional categorization was applied. This involved further grouping based on the proportion (less than 20%) of maternal education at a lower level, enabling the distinction between low-educated and high-educated populations.

To determine the most appropriate threshold for classifying studies into low- and high-educated categories, we plotted the distribution of low education level percentages among studies and also applied the K-Means clustering method. This analytical approach facilitated the identification of an optimal cutoff point, with a threshold of 20% chosen for our classification (Supplementary 3).

In this manuscript, we refer to “effect measures”, although it is important to recognize that, in observational research such as cohort studies, these metrics more accurately represent “association measures” due to their focus on observed associations rather than causal effects.

### Statistical analysis

The mean difference were used as effect measures for continuous variables (WAZ, HAZ and BMI-Z) and odds ratio were used as effect measures for categorical outcomes (stunting, underweight, overweight and RWG). We handled cases where multiple effect measures were reported as follows: when studies presented results separately for boys and girls, we merged them using a fixed effects model. For studies that reported results across multiple time points, we utilized a robust variance estimation method (with a correlation of 1) to calculate an overall estimate.^20^ Random-effects model (DerSimonian–Laird) was employed to conduct the meta-analysis. Every statistical test was two-sided, and p values of <0.05 were considered significant.

The Cochrane Q and I^2^ statistic were used to assess the heterogeneity among the studies included in the meta-analysis. A value of I^2^ greater than 50% indicated substantial heterogeneity. A significance level of p < 0.10 was used to determine whether the observed heterogeneity was statistically significant.

To investigate the potential sources of heterogeneity, we performed subgroup analyses based on several factors. These factors included the income status of the countries, population education status, rate of loss to follow-up and study quality.

Sensitivity analyses were conducted to evaluate whether the estimated effects differed between studies that reported crude and adjusted effect sizes separately. We had three types of studies: those reporting only crude effect size, those reporting only adjusted effect size, and those presenting both adjusted and crude effect sizes. Initially, to provide the overall effect size, for studies that reported both crude and adjusted effect sizes, we included the adjusted effect size. This approach was implemented to avoid double counting. Subsequently, we conducted sensitivity analyses separately for studies reporting only crude effect sizes and those reporting only adjusted effect sizes. As mentioned earlier, some studies presented both adjusted and crude effect sizes. In these instances, each study was included once in the adjusted meta-analysis and once in the crude meta-analysis. Additionally, we investigated the influence of child ages at 6, 12, and 24 months on the results. To further assess the robustness of our findings, we applied a one-out remove method.

In order to evaluate the presence of publication bias, we employed several methods including funnel plot analysis, Begg's and Egger's statistical tests, and the Trim and Fill method.

Stata, version 17.0 (StataCorp, College Station, TX, United States) were used to analyze the data.

### Role of the funding source

The financial support for this study, provided by Tehran University of Medical Sciences (TUMS), was solely for the student thesis. TUMS had no involvement in the design or execution of the study. The aggregated data utilized in this study will be made available through a designated repository at https://github.com/GolnazRezaei80/Maternal-Education-Child-Growth.

The decision to submit the manuscript for publication was made independently by the authors based on the research findings and significance of the study.

## Results

### Study selection

**Fig. 1:**
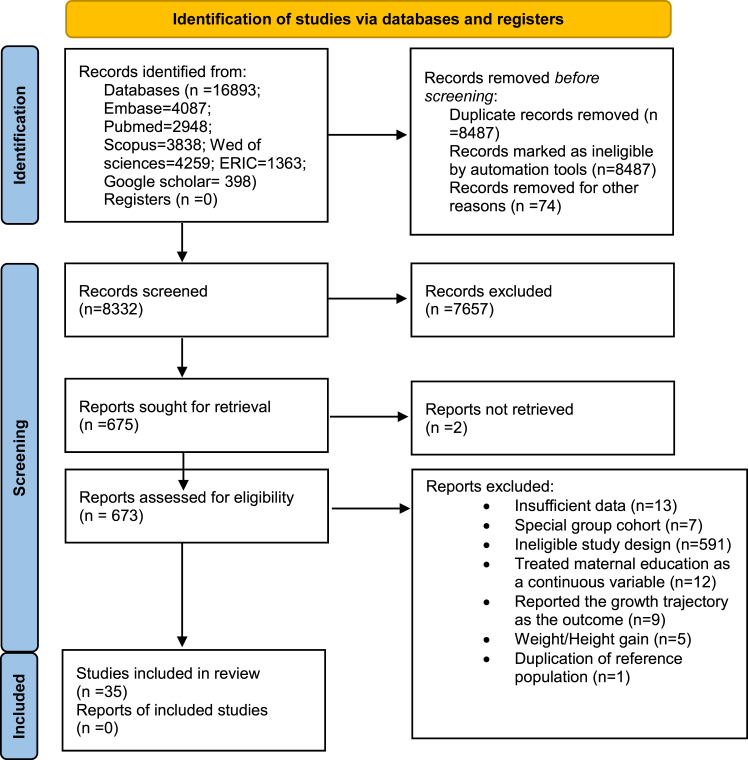
PRISMA flow diagram.

In the initial electronic search, a total of 16,893 studies were retrieved. After removing duplicates, the abstracts of 8332 studies were screened. Out of these, 673 studies were selected for full-text review. Finally, 35 studies were included in this systematic review and meta-analysis (Fig. 1). Among these studies, two cohort studies were identified, each providing information on two separate cohorts.^1^^,^^2^ The reference list of excluded studies and the reasons for their exclusion can be found in Supplementary 4.

### Study characteristics

The characteristics of the included studies are outlined in Table 1. A total of 37 studies met the eligibility criteria and were included in the systematic review. Eight outcomes were retrieved from these studies: WAZ (10 studies with 75,320 participants), HAZ (12 studies with 64,151 participants), BMI-Z (10 studies with 108,536 participants), overweight (10 studies with 59,658 participants), underweight (4 studies with 3841 participants), stunting (6 studies with 6063 participants), wasting (2 studies with 3145 participants), and rapid weight gain (4 studies with 12,270 participants). We did not find any population-based cohort studies that specifically examined the impact of maternal education on child growth in low-income countries. Furthermore, there were only one study available that assessed the effects of maternal education on child WAZ, and two studies available that assessed the effects on child BMI-Z in middle-income countries.

**Table 1:** Characteristics of studies included in the systematic review.

Author	Year	Sex	Month	Country	Inclusion criteria	N	Reference population	Maternal education levels	Outcome	Adjusted covariates	Country income category	Quality
Grjibovski AM**^21^**	2004	Combined^a^	12	Russia	Singleton Infants	1067	CDC	Basic, Secondary, Vocational, University (At least 3 years)	WAZ, HAZ	BF, Sex, SES, Preterm birth, SGA, BWZ-score, P, Prepregnancy maternal weight, Maternal age, Maternal occupation, Marital status	Upper middle income	Fair
Mesman I**^22^**	2009	Combined	14	Netherlands	Singleton & full-term Infants	3171	WHO	<5 after primary, 5–10 after primary, >10 after primary	WAZ, HAZ, BMI-Z	–	High income	Poor
Hui LL**^23^**	2010	Combined	12	Hong Kong	Singleton, full-term & healthy Infants	5949	WHO	9th grade or less, 10th to 11th grade, Greater than or equal to 12th grade	WAZ	Sex, GA, Birth order, Mother's place of birth, Father's education, Hospitalized for diarrhea by 3 months, Exclusively breast-fed >1 month, Second hand smoke exposure	High income	Good
van Rossem L**^24^**	2010	Combined	1, 6, 24	Netherlands	Singleton & first births	2954	National reference	Low (less than 4 years of high school), Mid-low (college), Mid-high (Bachelor's degree), High (Master's degree)	WAZ, HAZ, BMI-Z, Overweight	BF, Sex, BW, GA (Only for BMI-Z at 24 mo)	High income	Poor (WAZ, HAZ), good (BMI-Z, Overweight)
Chen YJ**^25^**	2014	Combined	1,6, 12, 14, 18, 24	Taiwan	All infants	21,193	WHO	Less than high school, High school, College and above	WAZ, HAZ	BF, Sex, GA, Method of delivery, Maternal age, Daycare caregiver at months	High income	Good
Hong SA**^26^**	2017	Combined	6, 12, 18, 24	Thailand	Singleton & healthy Infants	4178	WHO	Primary school or less (≤6 y of schooling), High school (7–12 y of schooling), College or higher (≥13 y of schooling).	WAZ, HAZ, Underweight, Stunting, Wasting	Sex, Study site	Upper middle income	Good
Kachi Y**^27^**	2018 (2001)	Combined	18	Japan	Singleton, full-term & not LBW Infants	34,503	WHO	Junior high school, High school, Some college, College or greater	WAZ	–	High income	Poor
Kachi Y**^27^**	2018 (2011)	Combined	18	Japan	Singleton, full-term & not LBW Infants	21,111	WHO	Junior high school, High school, Some college, College or greater	WAZ	–	High income	Poor
Ballon M**^28^**	2018	Boy/Girl^b^	1,6,12	France	Singleton Infants & literate mothers	1735	WHO	Low (failed to complete high school), Intermediate (high school diploma to 2-year university degree), High (3-year university degree or more).	WAZ, HAZ, BMI-Z, Overweight	Center	High income	Fair
Mekonnen T**^29^**	2021	Combined	1, 2, 6, 12, 18, 24	Norway	Singleton & healthy Infants	59,927	WHO	Low (≤12 years of education), Medium (13–16 years of education), High (≥17 years of education)	WAZ, HAZ, BMI-Z	Sex, BW, SES, GA, P	High income	Fair
Matijasevich A**^30^**	2012	Boy/Girl	24	Brazil	All infants	4053	WHO	0-4, 5–8,≥9	HAZ	–	Upper middle income	poor
Howe LD**^31^**	2012	Boy/Girl	12, 24	South-West England	All infants	12,366	WHO	Less than O-Level, O-Level, A-Level, Degree	HAZ	–	High income	poor
Silva LM**^32^**	2012	Combined	2, 6, 14	Netherlands	Singleton & first births	2972	WHO	Low (less than 4 years of high school), Mid-low (college), Mid-high (Bachelor's degree), and High (Master's degree).	HAZ, BMI-Z	BF, BW, GA, Maternal & paternal height, day care attendance	High income	Good (HAZ), poor (BMI-Z)
Dal Bom JP**^33^**	2019	Combined	10–15	Brazil	Singleton	759	WHO	≤9 years, 10–12 years, or >12 years	HAZ, BMI-Z	Sex	Upper middle income	Fair
Diana A**^34^**	2021	Combined	6, 9, 12	Indonesia	Healthy full term & breast fed infants	275	WHO	Less than high school, At least high school	HAZ	Sex, SES, Maternal height	Lower middle income	Good
Howe LD**^35^**	2010	Boy/Girl	24	South-West England	All infants	12,010	WHO	Less than O-Level, O-Level, A-Level, Degree	BMI-Z	–	High income	Poor
McCrory C**^36^**	2019	Boy/Girl	12, 24	Portugal	All infants	27,647	WHO	Primary, Secondary, Tertiary	BMI-Z	–	High income	Poor
McCrory C**^36^**	2019	Boy/Girl	12, 24	Ireland	All infants	27,647	WHO	Primary, Secondary, Tertiary	BMI-Z	–	High income	Poor
Morgen CS**^37^**	2017	Boy/Girl	5,12	Denmark	Singleton, full-term & healthy Infants	85,062	Internal reference value	<10 YEARS, 10–12 YEARS, >12 YEARS	BMI-Z	–	High income	Poor
Karaolis-Danckert N**^38^**	2008	Combined	24	Germany	Singleton, full-term & healthy Infants	353	German reference	<12 y of schooling,≥12 y of schooling	Rapid weight gain	–	High income	Poor
Mendez MA**^39^**	2011	Combined	6	Spain	Singleton & full-term	516	WHO	Primary, Secondary, University	Rapid weight gain	–	High income	Poor
Criswell R**^40^**	2017	Combined	6	Norway	Singleton	778	WHO	<12, 12, >12	Rapid weight gain	–	High income	Poor
Rotevatn TA**^41^**	2019	Combined	10, 24	Denmark	Full-term & not LBW	19,894	WHO	Lower secondary education, post-secondary non-tertiary education, short-cycle tertiary education, or Bachelor's degree or equivalent and Master's/Doctoral degree or equivalent	Rapid weight gain, Overweight	BF, Sex, BW, P, Prepregnancy maternal BMI, GA	High income	Poor (RWG), Good (Overweight)
Anderson SE**^42^**	2010	Combined	24	USA	Full-term & healthy	990	CDC2000	≤High school graduate, Some college, ≥College degree	Overweight	–	High income	Poor
Mastroeni MF**^43^**	2017	Combined	24	Brazil	Singleton, full-term & healthy Infants	305	WHO	<9 years for the mothers who completed primary school, 9–11, ≥12 years for those who started/finished undergraduate courses	Overweight	BF, Sex, BW, P, Prepregnancy maternal BMI, Maternal age, Income, Marital status, Type of delivery, Smoking after delivery	Upper middle income	Fair
Feldman-Winter L**^44^**	2017	Combined	24	USA	Singleton & healthy Infants	306	WHO	Some middle school, Some high school, High school graduate	Overweight	–	High income	Poor
Shay M**^45^**	2020	Combined	24	Canada	All infants	1570	WHO	High school or less, Some or complete university/college, and Some or complete graduate school	Overweight	–	High income	Poor
Xinmei Chen**^46^**	2020	Combined	24	Bangladesh	Healthy mother and infant	5752	WHO	≤9, 10–12,≥13	Overweight	–	Lower middle income	Poor
Zhou S**^47^**	2021	Combined	12	China	Healthy mother and infant, singleton & first birth	10,537	WHO	High school or below, Junior college, University or above	Overweight	–	Upper middle income	Poor
Mamabolo RL**^48^**	2003	Combined	12	South Africa	Full-term	276	NCHS	Primary, Secondary	Underweight, Stunting, Wasting	–	Upper middle income	Poor
Wightkin J**^49^**	2007	Combined	12	USA	All infants	4694	CDC2000	≤8 grade, 9–11 grade, 12 grade, college	Underweight	–	High income	Poor
Mondal D**^50^**	2012	Combined	12	Bangladesh	Healthy infants	147	WHO	No education, Educated	Underweight, Stunting	BWZ-score, Sex, SES, Prepregnancy maternal BMI	Lower middle income	Good
Barbara A**^51^**	2007	Combined	12, 24	South Africa	All infants	3275	CDC/WHO 1978 & CDC/NCHS 2000	<Matriculation, Matriculation	Stunting	Sex, SES, SGA	Upper middle income	Good
LL Jones**^52^**	2008	Combined	12, 24	Philippines	Singleton	2513	WHO	Less than high school, High school and more	Stunting	Sex, SES, Maternal height	Lower middle income	Fair
Mongkolchati A**^53^**	2010	Combined	6, 12, 18, 24	Thailand	Singleton & healthy Infants	4245	WHO	Primary school and lower, High school, College, University	Stunting	Sex, SES, Maternal height	Upper middle income	Good
Slemming W**^54^**	2017	Combined	24	South Africa	All infants	3275	WHO	≤7, 8–10, 11–12, Post school training	Stunting	–	Upper middle income	Poor
Das S**^55^**	2019	Combined	24	Bangladesh	Singleton & healthy Infants	211	WHO	No education, <5 y, >5 y	Stunting	–	Lower middle income	Poor

WAZ-score, Weight for Age Z-score, HAZ-score, Height for Age Z-score, BMIZ-score, Body Mass Index for Age Z-score, BWZ-score, Birth Weight Z-score, BW, Birth weight, BF, Breastfeeding, GA, Gestational age, P, Parity, SGA, Small for gestational age, SES, Socioeconomic status, WHO, World Health Organization, CDC, Centers for Disease Control and Prevention, NCHS, National Center for Health Statistics.

a Combined: The effect size is reported for boys and girls combined.

b Boy/Girl: The effect size is reported separately for boys and girls.

### Risk of bias in studies

**Fig. 2:**
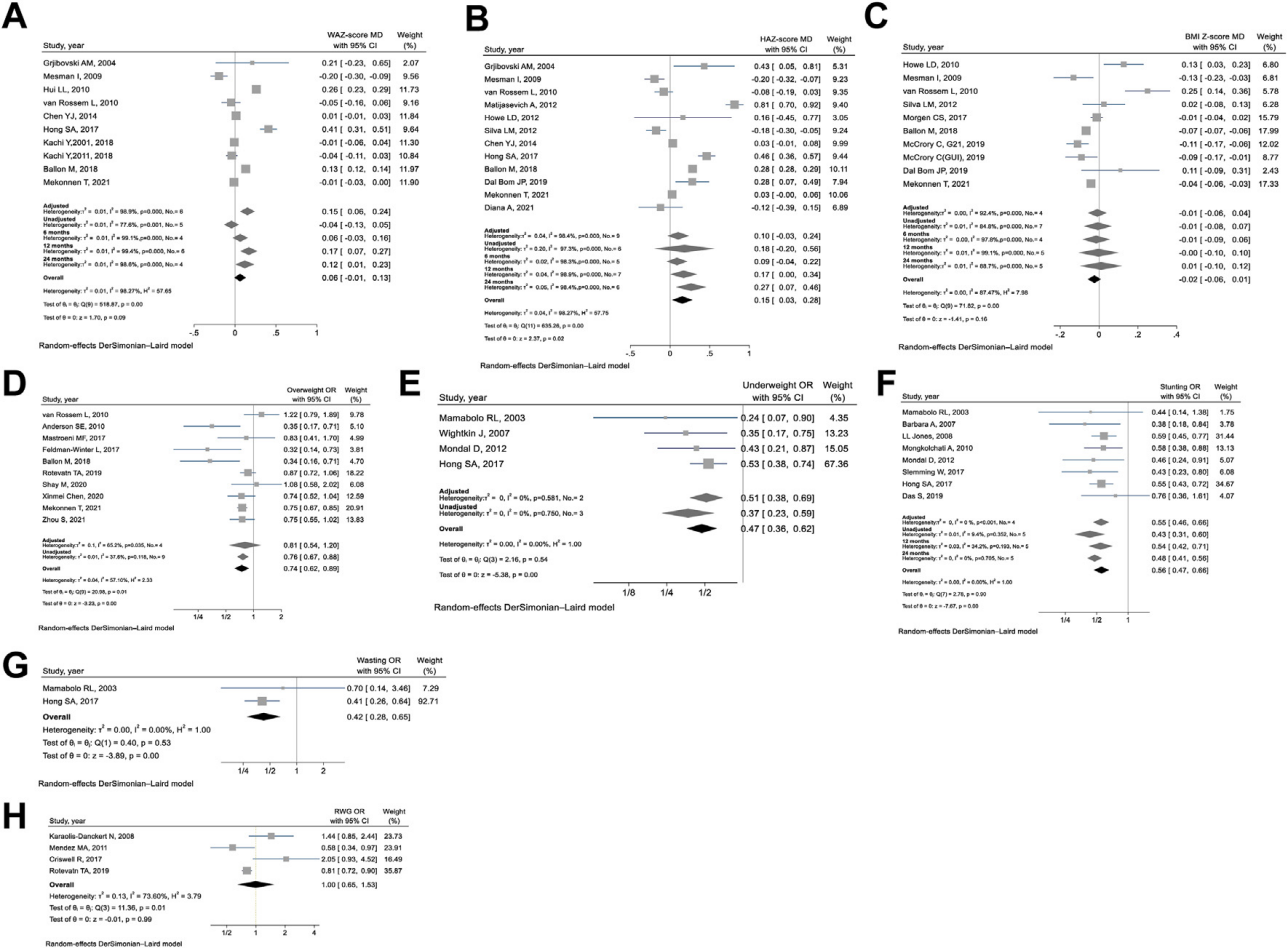
**Forest plots illustrating the impact of high versus low maternal education levels on the child's: A. Weight for Age Z-score, B. Height for Age Z-score, C. BMI for Age Z-score, D. Overweight, E. Underweight, F. Stunting, G. Wasting, and H. Rapid Weight Gain, from birth to age two**. High maternal education level: >12 years of schooling; Low maternal education level: <9 years of schooling; Overweight: BMI for Age Z-score ≥ the 85th percentile, 95th percentile, or >2 standard deviations from the median. Additionally, Weight-for-Height Z-score > the 97th percentile for their age and sex; Underweight: Weight for Age Z-score < −2 standard deviations from the median or Weight-for-Height Z-score < the 5th percentile; Stunting: Height for Age Z-score < −2 standard deviations from the median; Wasting: Weight-for-Height Z-score < −2 standard deviations from the median. WAZ-score, Weight for Age Z-score, HAZ-score, Height for Age Z-score, BMIZ-score, Body Mass Index for Age Z-score, RWG, Rapid Weight Gain, MD, Mean difference, OR, odds ratio.

Information regarding the quality rating can be found in Supplementary File 5. In most of the studies, the main factor contributing to a “not good” quality rating was related to the comparability domain, which evaluates the adjustment for mediators and confounding factors. As a result, we conducted separate sensitivity analyses for adjusted and unadjusted data, as illustrated in Fig. 2.

The question within the selection domain that exhibited the greatest variability was regarding the method of ascertaining exposure. However, in our study, the differences in the methods of ascertaining exposure are not critical, since all of them involve various forms of self-reporting.

**Fig. 3:**
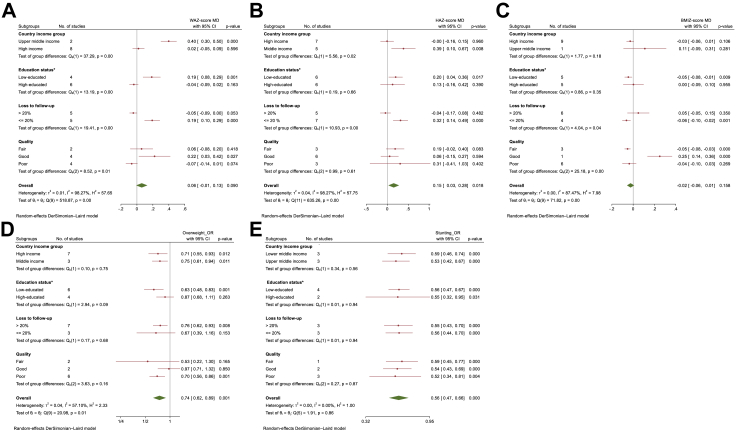
**The subgroup analysis was performed by country income group, education status of the country, loss to follow-up rate and study quality, for evaluating the effect of high versus low maternal education levels on child A. Weight for Age Z-score; B. Height for Age Z-score; C. BMI for Age Z-scores; D. Overweight and E. Stunting**. ∗Education status: Low-educated refers to equal or more than 20% of maternal education within each study population at the lowest level, while high-educated indicates less than 20% of maternal education within each study population at the lowest level. High maternal education level: >12 years of schooling; Low maternal education level: <9 years of schooling, WAZ-score, Weight for Age Z-score, HAZscore, Height for Age Z-score, BMIZ-score, Body Mass Index for Age Z-score, MD, Mean difference, OR, odds ratio.

Within the outcome domain, the question that displayed the greatest variability was related to the adequacy of cohort follow-up. Therefore, we conducted subgroup analyses based on this item (Fig. 3 & Supplementary 6).

### Certainty of evidence

Because the association between maternal education level and child growth and nutritional status varies across different country income groups and study population education statuses, as will be discussed further, the certainty levels of evidence were assessed in subgroups. The certainty levels of evidence assigned to these subgroups for each outcome are shown in Table 2.

**Table 2:** The subgroup analysis for evaluating the effect of high vs. low maternal education levels on child growth and certainty of evidence assessment.

Potential factors	Subgroups	Effect size (CI95%)^a^	No. of studies	Q	Heterogeneity p-value	I^2^	Interaction p-value	GRADE^d^					GRADE score
								Downgrades			Upgrades		
								Risk of bias	Inconsistency	Imprecision	Large effect	Dose- response	
WAZ^b^													
Country income group	Middle income	0.398 (0.301–0.496)	2	0.73	0.394	0%	<0.001						**High**
	High income	0.020 (−0.053 to 0.092)	8	483.62	<0.001	98.6%							**Very Low**
Education status^c^	Low-educated	0.186 (0.078–0.294)	4	352.41	<0.001	99.1%	<0.001						**Very Low**
	High-educated	−0.039 (−0.093 to 0.016)	6	18.42	0.002	72.9%							**Low**
Loss to follow-up	>20%	−0.045 (−0.091 to 0.001)	5	12.70	0.013	68.50	<0.001						
	≤ 20%	0.191 (0.097–0.286)	5	209.99	<0.001	98.10							
Quality	Fair	0.058 (−0.082 to 0.199)	2	229.62	<0.001	99.6%	0.014						
	Good	0.221 (0.025–0.417)	4	205.41	<0.001	98.5%	**-**						
	Poor	−0.066 (−0.137 to 0.006)	4	10.34	0.016	71.0%							
All studies	**-**	**0.061(**−**0.009 to 0.131)**	**10**	**518.87**	**<0.001**	**98.3%**							
HAZ^b^													
Country income group	Middle income	0.388 (0.102–0.673)	5	53.98	<0.001	92.6%	0.018						**Moderate**
	High income	−0.004 (−0.161 to 0.153)	7	518.10	<0.001	98.8%							**Moderate**
Education status	Low-educated	0.200 (0.036–0.365)	6	294.95	<0.001	98.3%	0.664						**Moderate**
	High-educated	0.127 (−0.162 to 0.416)	6	219.90	<0.001	97.7%							**Very Low**
Loss to follow-up	>20%	−0.044 (−0.168 to 0.079)	5	29.01	<0.001	86.21	0.001						
	≤ 20%	0.317 (0.142–0.491)	7	229.09	<0.001	97.34							
Quality	Fair	0.189 (−0.025 to 0.402)	3	274.74	<0.001	99.3%	0.609						
	Good	0.057 (−0.153 to 0.267)	6	91.13	<0.001	94.5%							
	Poor	0.308 (−0.413 to 1.030)	3	121.63	<0.001	98.4%							
All studies	**-**	**0.154 (0.027**–**0.282)**	**12**	**635.26**	**<0.001**	**98.3%**	–						
BMI-Z^b^													
Country income group	Middle income	0.110 (−0.090 to 0.310)	1	0	–	–	0.183	–	–	–	–	–	–
	High income	−0.028 (−0.061 to 0.006)	9	68.97	<0.001	88.40%							**Low**
Education status	Low-educated	−0.045 (−0.079 to −0.011)	5	27.36	<0.001	85.38%	0.354						**Moderate**
	High-educated	0.003 (−0.093 to 0.098)	5	29.38	<0.001	86.39%							**Low**
Loss to follow-up	>20%	0.048 (−0.053 to 0.150)	5	40.61	<0.001	87.69%	0.044						
	≤ 20%	−0.063 (−0.101 to −0.025)	3	15.41	0.001	80.53%							
Quality	Fair	−0.052 (−0.080 to −0.025)	3	11.05	<0.001	81.89%	<0.001						
	Good	0.250 (0.135–0.365)	1	0	–	–							
	Poor	−0.036 (−0.101 to 0.028)	6	25.59	<0.001	80.46%							
All studies	–	**−0.024 (−0.058 to 0.009)**	**10**	**71.82**	**<0.001**	**87.5%**	**–**						
Overweight													
Country income group	Middle income	0.753 (0.606–0.937)	3	0.09	0.956	0%	0.748						**Low**
	High income	0.712 (0.548–0.927)	7	20.84	0.002	71.21%							**Low**
Education status	Low-educated	0.634 (0.484–0.830)	4	15.30	0.009	67.33%	0.087						V**ery Low**
	High-educated	0.871 (0.683–1.110)	6	4.43	0.218	32.30%							**Low**
Loss to follow-up	>20%	0.761 (0.622–0.930)	7	14.97	0.020	59.92%	0.680						
	≤ 20%	0.674 (0.392–1.157)	3	5.66	0.059	64.67%							
Quality	Fair	0.532 (0.219–1.296)	2	2.99	0.084	66.60%	0.162						
	Good	0.970 (0.712–1.323)	2	1.92	0.166	47.86%							
	Poor	0.697 (0.563–0.863)	6	9.64	0.086	48.13%							
All studies	**–**	**0.744 (0.621**–**0.890)**	**10**	**20.98**	**0.013**	**57.1%**	**–**						
Stunting													
Country income group	Lower middle income	0.585 (0.461–0.743)	3	0.90	0.638	0%	0.558						**High**
	Upper middle income	0.530 (0.419–0.669)	3	0.67	0.717	0%							**High**
Education status	Low-educated	0.558 (0.467–0.667)	4	1.30	0.899	23.15%	0.943						**High**
	High-educated	0.547 (0.316–0.947)	2	0.59	0.254	0%							**Moderate**
Loss to follow-up	>20%	0.552 (0.433–0.704)	3	0.99	0.611	0%	0.940						
	≤ 20%	0.560 (0.445–0.704)	3	0.92	0.633	0%							
Quality	Fair	0.587 (0.448–0.770)	1	0	–	–	0.874						
	Good	0.542 (0.426–0.690)	2	0.23	0.633	0%							
	Poor	0.523 (0.337–0.813)	3	1.41	0.494	0%							
All studies	**–**	**0.556 (0.471**–**0.657)**	**6**	**1.91**	**0.862**	**0%**	**–**						

a Mean difference (MD) and odds ratio (OR) were used as the effect sizes.

b WAZ: weight for age z-score; HAZ: height for age z-score; BMI-Z: BMI for age z-scores.

c Education status: Low-educated refers to equal or more than 20% of maternal education within each study population at the lowest level, while high-educated indicates less than 20% of maternal education within each study population at the lowest level.

d We employed a modified GRADE method, outlined in detail in Supplementary File 2.  = 0;  = −1/+1;  = −2/+2

### Results of syntheses

#### Weight for age z-score

The overall impact (Fig. 2A) of high versus low maternal education level on WAZ indicates that a high maternal education level is more likely to be associated with higher WAZ. However, it should be noted that this effect was not found to be statistically significant (MD 0.061, 95% CI –0.009 to 0.131, p = 0.090, I^2^ = 98.3%). Only one study^28^ examined the association between maternal education and child WAZ separately for boys (MD 0.208, 95% CI 0.200–0.217) and girls (MD −0.058, 95% CI −0.070 to −0.046).

Based on the subgroup analysis (Fig. 3A & Table 2), a statistically significant difference was observed in the effect size between high-income and middle-income countries (p for interaction <0.001), as well as between high-educated and low-educated studies (p for interaction <0.001). Additionally, there was a significant difference found between studies with a loss to follow-up exceeding 20% and those with a loss to follow-up equal to or less than 20% (p for interaction <0.001). In high-income countries (MD 0.020, 95% CI −0.053 to 0.092) and high-educated population (MD −0.039, 95% CI −0.093 to 0.016), the association between maternal education level and WAZ is either absent or negative, but the negative association does not reach statistical significance. However, in middle-income (MD 0.398, 95% CI 0.301–0.496) and low-educated countries (MD 0.186, 95% CI 0.078–0.294), a high maternal education level is significantly associated with higher WAZ.

In studies where the loss to follow-up exceeded 20%, all of them were conducted in high-income countries, and the findings were consistent with studies that had a highly educated population. Importantly, in this particular case, the effect was found to be statistically significant (Fig. 3A & Supplementary 6).

The sensitivity analysis comparing crude and adjusted meta-analyses (Fig. 2A), reveals that a low maternal education level is favored in the crude analysis as a factor associated with higher WAZ (MD −0.044, 95% CI −0.133 to 0.045, p = 0.328, I^2^ = 77.6%). However, in the adjusted analysis, a high maternal education level is favored as the factor associated with higher WAZ (MD 0.151, 95% CI 0.062–0.239, p = 0.001, I^2^ = 98.9%). In contrast to the adjusted analysis, which includes studies from both middle-income and high-income countries and studies with a more balanced distribution of maternal education levels, the crude analysis focuses solely on high income and also highly educated studies (Supplementary 3).

A sensitivity analysis was conducted to assess the robustness of the results across different child ages (6, 12, and 24 months). Fig. 2A illustrates that the positive association between maternal education and child WAZ remained consistent across different child ages. However, this association was found to be statistically significant only in children aged 12 and 24 months.

#### Height for age z-score

The overall analysis (Fig. 2B) reveals a significant association between a higher level of maternal education and higher HAZ scores (MD 0.154, 95% CI 0.027–0.282, p = 0.018, I^2^ = 98.3%). Furthermore, there is no difference (p for interaction = 0.771) in this association between boys (MD −0.515, 95% CI 0.051–0.979, p = 0.029, I^2^ = 95.3%) and girls (MD 0.413, 95% CI −0.093 to 0.920, p = 0.110, I^2^ = 96.5%).

The subgroup analysis (Fig. 3B & Table 2) revealed a statistically significant difference in the effect size between high-income and middle-income countries (p for interaction = 0.018). However, no significant difference was observed between high-educated and low-educated studies (p for interaction = 0.664). Furthermore, a significant difference was found between studies with a loss to follow-up exceeding 20% and those with a loss to follow-up equal to or less than 20% (p for interaction = 0.001). Fig. 3B & Supplementary 6 illustrates the results of the subgroup analysis, indicating that this effect is only present in middle-income and low-educated countries. The majority of studies with a loss to follow-up exceeding 20% were conducted in high-income countries, and their findings were consistent with those of other high-income countries, where the effect was not statistically significant.

A sensitivity analysis demonstrates that this association remains consistent in both the crude and adjusted meta-analyses. However, it is crucial to note that although this association is consistent, it lacks statistical significance (Fig. 2B).

To evaluate the robustness of the results across different child ages (6, 12, and 24 months), a sensitivity analysis was performed. Fig. 2B illustrates that the positive association between maternal education and child HAZ remains consistent across different child ages. However, this association is statistically significant only in children aged 12 and 24 months.

#### BMI for age z-score

The overall analysis (Fig. 2C) indicates an association between a higher level of maternal education and lower BMI-Z, although this association is not statistically significant (MD −0.024, 95% CI −0.058 to 0.009, p = 0.158, I^2^ = 87.5%). Furthermore, there is no difference (Q = 0.97, p = 0.325) in this association between boys (MD −0.004, 95% CI −0.062 to 0.054, p = 0.894, I^2^ = 81.8%) and girls (MD −0.082, 95% CI −0.225 to 0.061, p = 0.264, I^2^ = 97.4%).

The subgroup analysis (Fig. 3C & Table 2) revealed no significant difference between high-income and middle-income countries (p for interaction = 0.183) and between high-educated and low-educated studies (p for interaction = 0.354). However, a significant difference was observed between studies with a loss to follow-up exceeding 20% and those with a loss to follow-up equal to or less than 20% (p for interaction = 0.044). All of the studies included in the analysis were conducted in high-income countries, except for one study conducted in an upper-middle-income country (Brazil) (Fig. 2C & Table 1). Subgroup analysis shows that among low-educated studies, there was a statistically significant negative association between a higher level of maternal education and lower BMI-Z (MD −0.045, 95% CI −0.079 to −0.011, p = 0.009, I^2^ = 85.4%). However, this negative association disappears among high-educated studies (MD 0.003, 95% CI −0.093 to 0.098, p = 0.955, I^2^ = 86.4%) (Fig. 3C & Supplementary 5). In studies with a loss to follow-up exceeding 20%, the findings were consistent with those of populations with higher levels of education. However, these findings were more positive in nature and did not reach statistical significance (Fig. 3C & Supplementary 5).

A sensitivity analysis demonstrates that the nonsignificant negative association between a higher level of maternal education and lower BMI-Z remains consistent in both the crude and adjusted meta-analyses (Fig. 2C).

To assess the robustness of the results across different child ages (6, 12, and 24 months), a sensitivity analysis was conducted. Fig. 2C illustrates that the association between maternal education and child BMI-Z does not vary across different child ages.

#### Overweight

The overall analysis indicates a statistically significant inverse association between maternal education level and the odds of being overweight. (OR 0.744, 95% CI 0.621–0.890, p = 0.001, I^2^ = 57.1%). The studies did not have separate data for boys and girls, and with the exception of one study conducted at 12 months,^47^ all the other studies focused on children aged 24 months.

Based on subgroup analysis (Fig. 3D & Table 2), there is no difference in the effect size between high-income and middle-income countries (p for interaction = 0.748). In both cases, there is a statistically significant association between a higher level of maternal education and a lower odds of being overweight. The subgroup analysis reveals a statistically significant difference in the effect size between low-educated and high-educated countries (p for interaction = 0.087), but only in low-educated countries, the effect size was statistically significant. Furthermore, there was no statistically significant difference between studies with a loss to follow-up exceeding 20% and those with a loss to follow-up equal to or less than 20% (p for interaction = 0.680).

A sensitivity analysis illustrated that the inverse association between maternal education level and the odds of being overweight remains consistent in both the crude and adjusted meta-analyses, but is only statistically significant in the crude analysis (Fig. 2D).

#### Underweight

Only four studies evaluated the effect of maternal education level on child underweight. The overall analysis indicates a statistically significant inverse association between maternal education level and the odds of being underweight (OR 0.473, 95% CI 0.360–0.621, p < 0.001, I^2^ = 0%). The studies did not have separate data for boys and girls, and with the exception of one study conducted at 6, 12, 18 and 24 months,^26^ all the other studies focused on children aged 12 months. All the studies were conducted in middle-income and also low-educated countries, except for one study which was conducted in a population sample of high-educated but low-income individuals in the United States of America.^49^

A sensitivity analysis illustrated that this inverse association remains consistent in both the unadjusted and adjusted meta-analyses, and it was statistically significant in both cases (Fig. 2E).

#### Stunting

The overall analysis indicates a statistically significant inverse association between maternal education level and the odds of being stunting (OR 0.556, 95% CI 0.471–0.657, p < 0.001, I^2^ = 0%). The studies did not have separate data for boys and girls. All the studies were conducted in middle-income countries and only two of them were high-educated (Fig. 2F & Table 1). The subgroup analysis (Fig. 3E & Table 2) indicates that there is no significant difference in the effect size between upper and lower middle-income countries (p for interaction = 0.558), as well as between low-educated and high-educated countries (p for interaction = 0.940). Furthermore, there was no statistically significant difference between studies with a loss to follow-up exceeding 20% and those with a loss to follow-up equal to or less than 20% (p for interaction = 0.943).

A sensitivity analysis illustrated that this inverse association remains consistent in both the unadjusted and adjusted meta-analyses, and it was statistically significant in both cases (Fig. 2F).

#### Wasting

Only two studies in upper middle-income countries were evaluated the effect of maternal education on wasting. The overall effect was protective and statistically significant (OR 0.424, 95% CI 0.275–0.654, p < 0.001, I^2^ = 0%).

#### Rapid weight gain

Only four studies were available to evaluate the effect of maternal education on rapid weight gain (RWG). The overall analysis suggests that there is no association between maternal education level and RWG (OR 0.997, 95% CI 0.648–1.534, p = 0.988, I^2^ = 73.6%). All of these studies were conducted in high-income countries, and only one study^40^ had a highly educated population. In this particular study, higher maternal education was associated with increased RWG, but the association was not statistically significant (OR 2.053, 95% CI 0.933–4.518, p = 0.074).

**Fig. 4:**
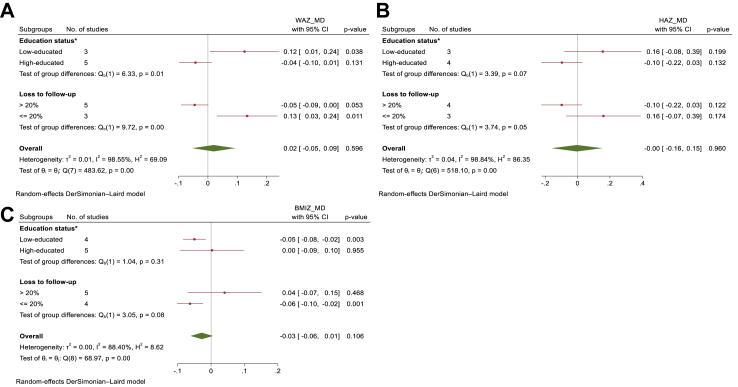
**In high-income countries the subgroup analysis was performed by education status of the country and loss to follow-up rate, for evaluating the effect of high versus low maternal education levels on child's: A. Weight for Age Z-score; B. Height for Age Zscore; C. BMI for Age Z-scores**. ∗Education status: Low-educated refers to equal or more than 20% of maternal education within each study population at the lowest level, while high-educated indicates less than 20% of maternal education within each study population at the lowest level. High maternal education level: >12 years of schooling; Low maternal education level: <9 years of schooling, WAZ-score, Weight for Age Z-score, HAZ-score, Height for Age Z-score, BMIZ, Body Mass Index for Age Z-score, MD, Mean difference.

Since the majority of studies examining the outcomes of WAZ and BMI-Z were conducted in high-income countries, we conducted a meta-analysis exclusively within this context. This approach allowed us to assess the impact of maternal education on WAZ, HAZ, and BMI-Z simultaneously (Fig. 4).

The results of leave-one-out method sensitivity analysis are presented in Supplementary File 7. The outcomes of the “leave-one-out” sensitivity analyses exhibited consistent trends with those of the primary analyses.

### Publication bias

As a general guideline, tests for funnel plot asymmetry should only be applied when there are a minimum of 10 studies included in the meta-analysis.^56^ This is because with fewer studies, the tests lack sufficient power to distinguish chance from genuine asymmetry. Our dataset comprises ten or more studies in the outcomes of WAZ, HAZ, BMI-Z, and overweight. Therefore, funnel plots can be assessed for these outcomes, all of which exhibited asymmetry, except for HAZ (Supplementary 8).

Since the evaluation of funnel plots is subjective,^56^ and the observed asymmetry in the funnel plot may originate from other sources, such as heterogeneity between results or chance,^57^ we complemented our evaluation by conducting Begg's and Egger's tests. Detailed results of these tests are available in Supplementary File 8. The tests did not yield statistically significant results for outcomes other than BMI-Z (Begg's p = 0.152; Egger's p = 0.008), overweight (Begg's p = 0.283; Egger's p = 0.076), underweight (Begg's p = 0.089; Egger's p = 0.151), and RWG (Begg's p = 0.089; Egger's p = 0.330). It is important to note that these tests are designed to identify the presence of publication bias rather than adjust for it.^56^ Consequently, we employed the Trim and Fill method to account for potential publication bias. This method first estimated the number of missing studies using symmetry assumptions and then imputed the missing values through a ‘trim and fill’ approach. This enabled us to derive adjusted estimates of the overall effect, considering the potential impact of publication bias.^56^^,^^58^

For the overweight outcome, this method did not identify any unpublished studies. However, for RWG and stunting, it estimated the presence of one unpublished study, and for BMI-Z and underweight, it estimated the presence of two unpublished studies. Nevertheless, the adjusted summary effects for BMI-Z (MD −0.054, 95% CI −0.090 to −0.017), underweight (MD −0.513, 95% CI 0.399–0.660), stunting (MD 0.568, 95% CI 0.483–0.667), and RWG (MD −0.864, 95% CI 0.565–1.320) exhibited no significant deviation from the observed summary effects (MD −0.024, 95% CI −0.058 to 0.009), (MD −0.473, 95% CI 0.360–0.621), (MD 0.556, 95% CI 0.471–0.657), and (MD 0.997, 95% CI 0.648–1.534), respectively. (Supplementary 8).

Finally, while some indications of potential publication bias were identified, the overall assessment does not strongly support the considerable publication bias across the analyzed outcomes.

## Discussion

To the best of our knowledge, this study is the first systematic review and meta-analysis examining the influence of maternal education on the growth of children under two years of age, using population-based cohort studies. The findings suggest that in countries with middle-income and/or low levels of education, a higher level of maternal education is significantly associated with higher WAZ and HAZ in children. However, in high-income and/or highly educated countries, this association is either absent or even reversed. In the meta-analysis of studies examining the relationship between maternal education and BMI-Z, which were primarily conducted in high-income countries, higher levels of maternal education were associated with lower BMI-Z (not statistically significant). Notably, this inverse association was statistically significant in studies involving low-educated populations, but not in studies involving highly educated populations. Numerous studies have established an association between undernutrition and childhood overweight.^7^^,^^8^^,^^59^ Our meta-analysis, in conjunction with Cole's findings,^60^ provides valuable insights into how undernutrition can contribute to the development of overweight in children. Cole^60^ conducted a simultaneous assessment of WAZ, HAZ, and BMI-Z, revealing a stronger correlation between BMI-Z and WAZ compared to HAZ. According to this study, the formula for BMI-Z is 1.434 × WAZ−0.794 × HAZ. Furthermore, to maintain a constant BMI-Z, HAZ must change at about twice the rate of WAZ, in the same direction. The interpretation of the effects of changes in HAZ, WAZ, and BMI-Z concurrently relies on the similarity of the studies included in the meta-analysis. In our meta-analysis, the significant unexplained heterogeneity observed in the impact of maternal education across different populations poses a challenge when interpreting changes in HAZ, WAZ, and BMI-Z. However, to enable concurrent interpretation of the effects of maternal education on HAZ, WAZ, and BMI-Z, we conducted a targeted meta-analysis specifically focusing on high-income countries, as the majority of studies examining the outcomes of WAZ and BMI-Z were conducted in these countries. Fig. 4 illustrates the divergent results observed in high-income countries between studies involving populations with high versus low levels of education. In studies involving low-educated populations, higher maternal education tended to be associated with higher WAZ and HAZ, but lower BMI-Z. Conversely, in highly educated studies, higher maternal education was linked to lower WAZ and HAZ. However, the effect on BMI-Z was either not discernible or showed a slight increase that was not statistically significant. The studies involving highly educated populations exhibited greater similarity because, they were primarily conducted in the Netherlands.^22^^,^^24^^,^^32^ In these studies, the mean difference in HAZ was approximately twice as large as the mean difference in WAZ. Consequently, based on Cole's finding,^60^ the mean difference in BMI-Z remained constant. Given the substantial unexplained heterogeneity observed in this meta-analysis, it is advisable to interpret the simultaneous impact of maternal education on HAZ, WAZ, and BMI-Z separately within each specific population. The diversity among study populations, including variations in the Social-Economic-Political-Emotional (SEPE) framework^61^—acknowledging the intricate interplay between biological development and community-specific socio-economic, cultural, political, and emotional conditions—can influence the relationship between maternal education and growth indicators in children. Consequently, this diversity may contribute to heterogeneity in the results obtained from different studies.

In the meta-analysis of studies conducted solely in high-income countries, investigating the relationship between maternal education and rapid child weight gain (RWG), no statistically significant association was found. RWG is recognized as a risk factor for childhood overweight.^62^ However, among these studies, the study conducted by Criswell et al. in a highly educated population in Norway showed a tendency for higher maternal education to be associated with increased RWG, although this association did not reach statistical significance.^40^ On the other hand, higher maternal education was found to be associated with a lower odds of being overweight in both high and middle-income countries. However, it is important to note that this association did not reach statistical significance in studies involving highly educated populations (Fig. 3D).

All the studies included in the current meta-analyses evaluating the impact of maternal education on underweight, stunting, and wasting, were conducted in middle-income countries. Higher maternal education was found to be associated with a reduced risk of these outcomes.

High rates of loss to follow-up in cohort studies often lead to the nonparticipation of individuals from less educated populations. As a result, the composition of study samples tends to resemble that of highly educated populations.^63^ In our study, we observed a similar trend in the association between maternal education and child growth indicators, as depicted in Fig. 3, to that seen in studies conducted with highly educated populations.

As we mentioned before and depicted in Table 2, significant unexplained heterogeneity was observed among the studies within each subgroup. Additionally, the issue of “no effect” in our subgroup meta-analysis was deemed as crucial as detecting a large effect. Therefore the certainty of evidence in most of subgroups is not sufficient to assert the association of higher maternal education with child growth and nutritional status. Except that there is high certainty of evidence that higher maternal education in middle-income countries is associated with higher WAZ and also lower stunting.

This study has some notable limitations. Firstly, we were unable to find any population-based cohort studies that specifically examined the impact of maternal education on child growth in low-income countries. Additionally, there were only a limited number of studies available that assessed the effects of maternal education on child WAZ and BMI-Z in middle-income countries. Consequently, the generalizability of our findings to different income contexts may be limited. These limitations highlight the need for further research in low-income countries and more comprehensive investigations in middle-income countries to better understand the influence of maternal education on child growth outcomes.

The main strength of this study is to simultaneously examine the impact of maternal education on all types of child growth impairments, while specifically considering country income levels and population education status. This unique approach enhances the current body of research and contributes to a deeper understanding of the relationship between maternal education and child growth status.

In conclusion, the impact of maternal education on child growth and nutritional status varied depending on the income and education level of the population. In middle-income and/or low-educated populations, high maternal education was positively associated with child growth. However, in high-income populations, this positive association was not observed. When we conducted a subgroup analysis, a statistically significant difference was observed in the effect size between high-educated and low-educated studies in high-income countries, indicating a notable interaction between maternal education level and the education status of the studies. Visually, these effects were on both sides of the null in low and high educated populations, hence, referring to Cole's study formula, we can consider obesity in highly educated populations from a different perspective–not solely as a result of overnutrition, but also as a complication of undernutrition. Yet, from a compatibility standpoint, the confidence intervals of both of these effects can include clinically not important amounts.^64^, ^65^, ^66^ Therefore, the issue of how maternal education leads to different outcomes in child growth and nutritional status, should be evaluated in each specific population, based on the characteristics of the population studied. It is important to identify the specific mediators involved in this relationship in order to gain a comprehensive understanding.

## Contributors

GR conducted literature research, analyzed the data, and authored the article. GR and ZF were responsible for selecting full-text articles. GR and FZ were accountable for the quality assessment of studies and data extraction. AK contributed to manuscript editing, accessed and verified data, and provided methodology consultation. RK served as the project administrator and pediatric consultant. MSH accessed and verified the data, providing biostatistics consultation. MM, as a supervisor, offered manuscript editing, accessed and verified data, and provided epidemiology consultation. HP, also a supervisor, contributed to manuscript editing and epidemiology consultation.

## Data sharing statement

The aggregated data utilized in this study will be made available upon publication through a designated repository (https://github.com/GolnazRezaei80/Maternal-Education-Child-Growth).

## Declaration of generative AI and AI-assisted technologies in the writing processd

During the preparation of this work the author(s) used ChatGPT, an AI language model developed by OpenAI in order to enhance the readability and language of our study, without replacing essential researcher tasks like generating scientific insights, analyzing and interpreting data, or drawing scientific conclusions. After using this tool/service, the author(s) reviewed and edited the content as needed and take(s) full responsibility for the content of the publication.

## Declaration of interests

We declare no competing interests.

## References

[1] Akombi B.J., Agho K.E., Hall J.J. (2017). http://www.mdpi.com/journal/ijerph.

[2] Juan J., Yang H. (2022). Early life 1000 days: opportunities for preventing adult diseases. Chin Med J.

[3] Improving Child Nutrition: the achievable imperative for global progress - UNICEF DATA. https://data.unicef.org/resources/improving-child-nutrition-the-achievable-imperative-for-global-progress/.

[4] Victora C.G., De Onis M., Hallal P.C., Blössner M., Shrimpton R. (2010). Worldwide timing of growth faltering: revisiting implications for interventions. Pediatrics.

[5] (UK) NGA (2017). https://www.ncbi.nlm.nih.gov/books/NBK458459/.

[6] de Onis M., Blössner M. (2003). The World Health Organization global database on child growth and malnutrition: methodology and applications. Int J Epidemiol.

[7] Matrins V.J.B., Toledo Florêncio T.M.M., Grillo L.P. (2011). Long-lasting effects of undernutrition. Int J Environ Res Public Health.

[8] Black R.E., Victora C.G., Walker S.P. (2013). Maternal and Child Nutrition 1 Maternal and child undernutrition and overweight in low-income and middle-income countries. Lancet.

[9] Ngandu C.B., Momberg D., Magan A., Chola L., Norris S.A., Said-Mohamed R. (2020). The association between household socio-economic status, maternal socio-demographic characteristics and adverse birth and infant growth outcomes in sub-Saharan Africa: a systematic review. J Dev Orig Health Dis.

[10] Abate K.H., Belachew T. (2019). Chronic malnutrition among under five children of Ethiopia may not Be economic. A systematic review and meta-analysis. Ethiop J Health Sci.

[11] Keino S., Plasqui G., Ettyang G., Van Den Borne B. (2014). Determinants of stunting and overweight among young children and adolescents in sub-Saharan Africa. Food Nutr Bull.

[12] Balaj M., York H.W., Sripada K. (2021). Articles Parental education and inequalities in child mortality: a global systematic review and meta-analysis. Lancet.

[13] Wells G., Shea B., O'Connell D., Peterson J. (2000). https://scholar.archive.org/work/zuw33wskgzf4bceqgi7opslsre/access/wayback/http://www3.med.unipmn.it/dispense_ebm/2009-2010/CorsoPerfezionamentoEBM_Faggiano/NOS_oxford.pdf.

[14] Rohatgi A. (2022). http://www.arohatgi.info/WebPlotDigitizer.

[15] Mei Z., Grummer-Strawn L.M. (2007). Standard deviation of anthropometric Z-scores as a data quality assessment tool using the 2006 WHO growth standards: a cross country analysis. Bull World Health Organ.

[16] Wang Y., Chen H.J. (2012). Use of percentiles and Z -scores in anthropometry. Handb Anthr Phys Meas Hum Form Heal Dis.

[17] Halilagic A., Moschonis G. (2021). The effect of growth rate during infancy on the risk of developing obesity in childhood: a systematic literature review. Nutrients.

[18] New World Bank country classifications by income level: 2022-2023. https://blogs.worldbank.org/opendata/new-world-bank-country-classifications-income-level-2022-2023.

[19] OECD (2023). https://www.oecd-ilibrary.org/education/education-at-a-glance-2023_e13bef63-en.

[20] Borenstein M., Hedges L.V., Higgins J.P.T., Rothstein H.R. (2009). https://onlinelibrary.wiley.com/doi/book/10.1002/9780470743386.

[21] Grjibovski A.M., Bygren L.O., Yngve A., Sjostrom M. (2004). Social variations in infant growth performance in Severodvinsk, Northwest Russia: community-based cohort study. Croat Med J.

[22] Mesman I., Roseboom T.J., Bonsel G.J., Gemke R.J., van der Wal M.F., Vrijkotte T.G.M. (2009). Maternal pre-pregnancy body mass index explains infant's weight and BMI at 14 months: results from a multi-ethnic birth cohort study. Arch Dis Child.

[23] Hui L.L., Leung G.M., Cowling B.J., Lam T.H., Schooling C.M. (2010). Determinants of infant growth: evidence from Hong Kong's “Children of 1997” birth cohort. Ann Epidemiol.

[24] van Rossem L., Silva L.M., Hokken-Koelega A. (2010). Socioeconomic status is not inversely associated with overweight in preschool children. J Pediatr.

[25] Chen Y.-J., Li C.-R., Lee S.-H. (2014). Growth changes in infants born of adolescent mothers: results of a national cohort study in Taiwan. Iran J Reprod Med.

[26] Hong S.A., Winichagoon P., Mongkolchati A. (2017). Inequality in malnutrition by maternal education levels in early childhood: the Prospective Cohort of Thai Children (PCTC). Asia Pac J Clin Nutr.

[27] Kachi Y., Fujiwara T., Yamaoka Y., Kato T. (2018). Parental socioeconomic status and weight faltering in infants in Japan. Front Pediatr.

[28] Ballon M., Botton J., Charles M.A. (2018). Socioeconomic inequalities in weight, height and body mass index from birth to 5 years. Int J Obes.

[29] Mekonnen T., Papadopoulou E., Arah O.A., Brantsæter A.L., Lien N., Gebremariam M.K. (2021). Socioeconomic inequalities in children's weight, height and BMI trajectories in Norway. Sci Rep.

[30] Matijasevich A., Howe L.D., Tilling K., Santos I.S., Barros A.J.D., Lawlor D.A. (2012). Maternal education inequalities in height growth rates in early childhood: 2004 Pelotas birth cohort study. Paediatr Perinat Epidemiol.

[31] Howe L.D., Tilling K., Galobardes B., Smith G.D., Gunnell D., Lawlor D.A. (2012). Socioeconomic differences in childhood growth trajectories: at what age do height inequalities emerge?. J Epidemiol Community Health.

[32] Silva L.M., van Rossem L., Jansen P.W. (2012). Children of low socioeconomic status show accelerated linear growth in early childhood; results from the Generation R Study. PLoS One.

[33] Dal Bom J.P., Mazzucchetti L., Malta M.B. (2019). Early determinants of linear growth and weight attained in the first year of life in a malaria endemic region. PLoS One.

[34] Diana A., Haszard J.J., Sari S.Y.I. (2021). Determination of modifiable risk factors for length-for-age z-scores among resource-poor Indonesian infants. PLoS One.

[35] Howe L.D., Tilling K., Galobardes B., Lawlor D. (2009). 6th World congress on developmental origins of health & disease.

[36] McCrory C., Leahy S., Ribeiro A.I. (2019). Maternal educational inequalities in measured body mass index trajectories in three European countries. Paediatr Perinat Epidemiol.

[37] Morgen C.S., Andersen P.K., Mortensen L.H. (2017). Socioeconomic disparities in birth weight and body mass index during infancy through age 7 years: a study within the Danish National Birth Cohort. BMJ Open.

[38] Karaolis-Danckert N., Buyken A.E., Kulig M. (2008). How pre- and postnatal risk factors modify the effect of rapid weight gain in infancy and early childhood on subsequent fat mass development: results from the Multicenter Allergy Study 90. Am J Clin Nutr.

[39] Mendez M.A., Garcia-Esteban R., Guxens M., Kogevinas M., Sunyer J. (2010). Organochlorine compounds and rapid infant growth as a marker of obesity risk: the INMA study. Obes Rev.

[40] Criswell R., Lenters V., Mandal S., Stigum H., Iszatt N., Eggesbø M. (2017). Persistent environmental toxicants in breast milk and rapid infant growth. Ann Nutr Metab.

[41] Rotevatn T.A., Overgaard C., Melendez-Torres G.J. (2019). Infancy weight gain, parental socioeconomic position, and childhood overweight and obesity: a Danish register-based cohort study. BMC Publ Health.

[42] Anderson S.E., He X., Schoppe-Sullivan S., Must A. (2010). Externalizing behavior in early childhood and body mass index from age 2 to 12 years: longitudinal analyses of a prospective cohort study. BMC Pediatr.

[43] Mastroeni M.F., De Barros Silva Mastroeni S.S., Czarnobay S.A., Ekwaru J.P., Loehr S.A., Veugelers P.J. (2017). Breast-feeding duration for the prevention of excess body weight of mother-child pairs concurrently: a 2-year cohort study. Public Health Nutr.

[44] Feldman-Winter L., Burnham L., Grossman X., Matlak S., Chen N., Merewood A. (2018). Weight gain in the first week of life predicts overweight at 2 years: a prospective cohort study. Matern Child Nutr.

[45] Shay M., Tomfohr-Madsen L., Tough S. (2020). Maternal psychological distress and child weight at 24 months: investigating indirect effects through breastfeeding in the All Our Families cohort. Can J Public Health.

[46] Chen X., Liao J., Xu S. (2020). Associations of exposure to nitrogen dioxide and major roadways with growth trajectories and obesity at 2 years old: a prospective cohort study. Atmos Environ.

[47] Zhou S., Lin L., Bao Z. (2021). The association of prenatal exposure to particulate matter with infant growth: a birth cohort study in Beijing, China. Environ Pollut.

[48] Mamabolo R.L., Alberts M., Mbenyane G.X. (2004). Feeding practices and growth of infants from birth to 12 months in the central region of the Limpopo Province of South Africa. Nutrition.

[49] Wightkin J., Magnus J.H., Farley T.A., Boris N.W., Kotelchuck M. (2007). Psychosocial predictors of being an underweight infant differ by racial group: a prospective study of Louisiana WIC program participants. Matern Child Health J.

[50] Mondal D., Minak J., Alam M. (2012). Contribution of enteric infection, altered intestinal barrier function, and maternal malnutrition to infant malnutrition in Bangladesh. Clin Infect Dis.

[51] Willey B.A. (2007).

[52] Jones L.L., Griffiths P.L., Adair L.S., Norris S.A., Richter L.M., Cameron N. (2008). A comparison of the socio-economic determinants of growth retardation in South African and Filipino infants. Public Health Nutr.

[53] Mongkolchati A., Thinkhamrop B., Mo-Suwan L., Chittchang U., Choprapawon C. (2010). Prevalence and incidence of child stunting from birth to two years of life in Thai children: based on the Prospective Cohort Study of Thai Children (PCTC). J Med Assoc Thai.

[54] Slemming W., Kagura J., Saloojee H., Richter L.M. (2017). Early life risk exposure and stunting in urban South African 2-year old children. J Dev Orig Health Dis.

[55] Das S., Alam M.A., Mahfuz M., Arifeen S.E., Ahmed T. (2019). Relative contributions of the correlates of stunting in explaining the mean length-for-age z-score difference between 24-month-old stunted and non-stunted children living in a slum of Dhaka, Bangladesh: results from a decomposition analysis. BMJ Open.

[56] Duval S., Tweedie R. (2000). Trim and fill: a simple funnel-plot-based method of testing and adjusting for publication bias in meta-analysis. Biometrics.

[57] Egger M., Smith G.D., Schneider M., Minder C. (1997). Bias in meta-analysis detected by a simple, graphical test. BMJ.

[58] Duval S., Tweedie R. (2000). A nonparametric “trim and fill” method of accounting for publication bias in meta-analysis. J Am Stat Assoc.

[59] Hermanussen M., Novine M., Scheffler C., Groth D. (2022). The arithmetic dilemma when defining thinness, overweight and obesity in stunted populations. Hum Biol Public Heal.

[60] Cole T.J. (2002). A chart to link child centiles of body mass index, weight and height. Eur J Clin Nutr.

[61] Bogin B. (2021).

[62] Monteiro P.O.A., Victora C.G. (2005). https://onlinelibrary.wiley.com/doi/10.1111/j.1467-789X.2005.00183.x.

[63] Howe L.D., Tilling K., Galobardes B., Lawlor D.A. (2013). Loss to follow-up in cohort studies: bias in estimates of socioeconomic inequalities. Epidemiology.

[64] Mansournia M.A., Nazemipour M. (2024). Recommendations for accurate reporting in medical research statistics. Lancet.

[65] Greenland S., Mansournia M.A., Joffe M. (2022). To curb research misreporting, replace significance and confidence by compatibility: a Preventive Medicine Golden Jubilee article. Prev Med.

[66] Mansournia M.A., Nazemipour M., Etminan M. (2022). P-value, compatibility, and S-value. Glob Epidemiol.

