# Supplementary material for: Maternal education and its influence on child growth and nutritional status during the first two years of life: a systematic review and meta-analysis
Source: eClinicalMedicine. 2024 Apr 4;71:102574. doi: 10.1016/j.eclinm.2024.102574 (PMC11001623; doi:10.1016/j.eclinm.2024.102574)
Supplement: Supplementary File 1 [file mmc1.pdf]

Supplementary 1

➢ Pubmed (reference database) Syntax

Search Round	*Pubmed (reference database) Syntax*	*NNR*	*Output No.*	*Output No. after importing to mendely*	*Output No. after Removing duplications*
1	(((“Educational Status”[all] AND Maternal[all]) OR (Status[all] AND “Maternal Educational”[all]) OR “Maternal Educational Status”[all] OR (Maternal[all] AND education[all]) OR (Maternal[all] AND Illiteracy[all]) OR (Maternal[all] AND Literacy[all]) OR (maternal[all] AND “schooling”[all]) OR (mother*[all] AND education[all]) OR (mother*[all] AND Illiteracy[all]) OR (mother*[all] AND Literacy[all]) OR (mother*[all] AND “schooling”[all]) OR (parent*[all] AND Illiteracy[all]) OR (parent*[all] AND Literacy[all]) OR (parent*[all] AND education[all]) OR (parent*[all] AND “schooling”[all]) OR (parent*[all] AND socio-economic[all]) OR (parent*[all] AND socioeconomic[all]) OR (parent*[all] AND “Social Class*”[all]) OR (parent*[all] AND Socio-demographic[all]) OR (parent*[all] AND Sociodemographic[all]) OR (parent*[all] AND Socio-cultural[all]) OR (parent*[all] AND Sociocultural[all]) OR (mother*[all] AND socio-economic[all]) OR (mother*[all] AND socioeconomic[all]) OR (mother*[all] AND “Social Class*”[all]) OR (mother*[all] AND Socio-demographic[all]) OR (mother*[all] AND Sociodemographic[all]) OR (mother*[all] AND Socio-cultural[all]) OR (mother*[all] AND Sociocultural[all]) OR (maternal[all] AND socio-economic[all]) OR (maternal[all] AND socioeconomic[all]) OR (maternal[all] AND “Social Class*”[all]) OR (maternal[all] AND Socio-demographic[all]) OR (maternal[all] AND Sociodemographic[all]) OR (maternal[all] AND Socio-cultural[all]) OR (maternal[all] AND Sociocultural[all]) OR “maternal socioeconomic status”[all] OR “parental socioeconomic status”[all])<br>AND<br>((Child*[all] AND Growth[all]) OR (Child*[all] AND “Growth Retardation”[all]) OR (Child*[all] AND “Growth Disorder*”[all]) OR (Child*[all] AND “Growth Falter*”[all]) OR (Child*[all] AND “Growth Deficit*”[all]) OR (Child*[all] AND “Growth failure”[all]) OR (Child*[all] AND “Growth Trajectory”[all]) OR Stunting*[all] OR “Stunted Growth”[all] OR (Growth[all] AND Stunted[all]) OR “short stature”[all] OR wast*[all] OR (Child*[all] AND “Nutrition Disorder*”[all]) OR (Child*[all] AND Malnutrition[all]) OR (Child*[all] AND Undernutrition[all]) OR (Child*[all] AND Under-nutrition[all]) OR (Child*[all] AND Undernourish*[all]) OR (Child*[all] AND Overnutrition[all]) OR (Child*[all] AND Underweight[all]) OR (Child*[all] AND Leanness[all]) OR (Child*[all] AND Thinness[all]) OR (Child*[all] AND Slimness[all]) OR (Child*[all] AND Overweight[all]) OR (Child*[all] AND Obes*[all]) OR (Child*[all] AND Height*[all]) OR (Child*[all] AND Weight*[all]) OR (Child*[all] AND Length[all]) OR Anthropometric*[all] OR Length-for-age[all] OR Height-for-age[all] OR Weight-for-age[all] OR Weight-for-height[all] OR weight-for-length[all] OR “Body mass index-for-age”[all] OR BMI-for-age[all] OR (child*[all] AND “failure to thrive”[all]) OR (Infan*[all] AND Growth[all]) OR (Infan*[all] AND “Growth Retardation”[all]) OR (Infan*[all] AND “Growth Disorder*”[all]) OR (Infan*[all] AND “Growth Falter*”[all]) OR (Infan*[all] AND “Growth Deficit*”[all]) OR (Infan*[all] AND “Growth failure”[all]) OR (Infan*[all] AND “Growth Trajectory”[all]) OR (Infan*[all] AND “Nutrition Disorder*”[all]) OR (Infan*[all] AND Malnutrition[all]) OR (Infan*[all] AND Undernutrition[all]) OR (Infan*[all] AND Under-nutrition[all]) OR (Infan*[all] AND Undernourish*[all]) OR (Infan*[all] AND Overnutrition[all]) OR (Infan*[all] AND Underweight[all]) OR (Infan*[all] AND Leanness[all]) OR (Infan*[all] AND Thinness[all]) OR (Infan*[all] AND Slimness[all]) OR (Infan*[all] AND Overweight[all]) OR (Infan*[all] AND Obes*[all]) OR (Infan*[all] AND Height*[all]) OR (Infan*[all] AND Weight*[all]) OR (Infan*[all] AND Length[all]) OR (infan*[all] AND “failure to thrive”[all]) OR (Under-five[all] AND Growth[all]) OR (Under-five[all] AND “Growth Retardation”[all]) OR (Under-five[all] AND “Growth Disorder*”[all]) OR (Under-five[all] AND “Growth Falter*”[all]) OR (Under-five[all] AND “Growth Deficit*”[all]) OR (Under-five[all] AND “Growth failure”[all]) OR (Under-five[all] AND “Growth Trajectory”[all]) OR (Under-five[all] AND “Nutrition Disorder*”[all]) OR (Under-five[all] AND Malnutrition[all]) OR (Under-five[all] AND Undernutrition[all]) OR (Under-five[all] AND Under-nutrition[all]) OR (Under-five[all] AND Undernourish*[all]) OR (Under-five[all] AND Overnutrition[all]) OR (Under-five[all] AND Underweight[all]) OR (Under-five[all] AND Leanness[all]) OR (Under-five[all] AND Thinness[all]) OR (Under-five[all] AND Slimness[all]) OR (Under-five[all] AND Overweight[all]) OR (Under-five[all] AND Obes*[all]) OR (Under-five[all] AND Height*[all]) OR (Under-five[all] AND Weight*[all]) OR (Under-five[all] AND Length[all]) OR (under-five[all] AND “failure to thrive”[all]) OR (“under five”[all] AND Growth[all]) OR (“under five”[all] AND “Growth Retardation”[all]) OR (“under five”[all] AND “Growth Disorder*”[all]) OR (“under five”[all] AND “Growth Falter*”[all]) OR (“under five”[all] AND “Growth Deficit*”[all]) OR (“under five”[all] AND “Growth failure”[all]) OR (“under five”[all] AND “Growth Trajectory”[all]) OR (“under five”[all] AND “Nutrition Disorder*”[all]) OR (“under five”[all] AND Malnutrition[all]) OR (“under five”[all] AND Undernutrition[all]) OR (“under five”[all] AND	200	13,552		

	Under-nutrition[all]) OR (“under five”[all] AND Undernourish*[all]) OR (“under five”[all] AND Overnutrition[all]) OR (“under five”[all] AND Underweight[all]) OR (“under five”[all] AND Leanness[all]) OR (“under five”[all] AND Thinness[all]) OR (“under five”[all] AND Slimness[all]) OR (“under five”[all] AND Overweight[all]) OR (“under five”[all] AND Obes*[all]) OR (“under five”[all] AND Height*[all]) OR (“under five”[all] AND Weight*[all]) OR (“under five”[all] AND Length[all]) OR (“under five”[all] AND “failure to thrive”[all]) OR (“under 5”[all] AND Growth[all]) OR (“under 5”[all] AND “Growth Retardation”[all]) OR (“under 5”[all] AND “Growth Disorder*”[all]) OR (“under 5”[all] AND “Growth Falter*”[all]) OR (“under 5”[all] AND “Growth Deficit*”[all]) OR (“under 5”[all] AND “Growth failure”[all])OR (“under 5”[all] AND “Growth Trajectory”[all]) OR (“under 5”[all] AND “Nutrition Disorder*”[all]) OR (“under 5”[all] AND Malnutrition[all]) OR (“under 5”[all] AND Undernutrition[all]) OR (“under 5”[all] AND Under-nutrition[all]) OR (“under 5”[all] AND Undernourish*[all]) OR (“under 5”[all] AND Overnutrition[all]) OR (“under 5”[all] AND Underweight[all]) OR (“under 5”[all] AND Leanness[all]) OR (“under 5”[all] AND Thinness[all]) OR (“under 5”[all] AND Slimness[all]) OR (“under 5”[all] AND Overweight[all]) OR (“under 5”[all] AND Obes*[all]) OR (“under 5”[all] AND Height*[all]) OR (“under 5”[all] AND Weight*[all]) OR (“under 5”[all] AND Length[all]) OR (“under 5”[all] AND “failure to thrive”[all]) OR (Under-5[all] AND Growth[all]) OR (Under-5[all] AND “Growth Retardation”[all]) OR (Under-5[all] AND “Growth Disorder*”[all]) OR (Under-5[all] AND “Growth Falter*”[all]) OR (Under-5[all] AND “Growth Deficit*”[all]) OR (Under-5[all] AND “Growth failure”[all]) OR (Under-5[all] AND “Growth Trajectory”[all]) OR (Under-5[all] AND “Nutrition Disorder*”[all]) OR (Under-5[all] AND Malnutrition[all]) OR (Under-5[all] AND Undernutrition[all]) OR (Under-5[all] AND Under-nutrition[all]) OR (Under-5[all] AND Undernourish*[all]) OR (Under-5[all] AND Overnutrition[all]) OR (Under-5[all] AND Underweight[all]) OR (Under-5[all] AND Leanness[all]) OR (Under-5[all] AND Thinness[all]) OR (Under-5[all] AND Slimness[all]) OR (Under-5[all] AND Overweight[all]) OR (Under-5[all] AND Obes*[all]) OR (Under-5[all] AND Height*[all]) OR (Under-5[all] AND Weight*[all]) OR (Under-5[all] AND Length[all]) OR (Under-5[all] AND “failure to thrive”[all]) OR (Under-two[all] AND Growth[all]) OR (Under-two[all] AND “Growth Retardation”[all]) OR (Under-two[all] AND “Growth Disorder*”[all]) OR (Under-two[all] AND “Growth Falter*”[all]) OR (Under-two[all] AND “Growth Deficit*”[all]) OR (Under-two[all] AND “Growth failure”[all]) OR (Under-two[all] AND “Growth Trajectory”[all]) OR (Under-two[all] AND “Nutrition Disorder*”[all]) OR (Under-two[all] AND Malnutrition[all]) OR (Under-two[all] AND Undernutrition[all]) OR (Under-two[all] AND Under-nutrition[all]) OR (Under-two[all] AND Undernourish*[all]) OR (Under-two[all] AND Overnutrition[all]) OR (Under-two[all] AND Underweight[all]) OR (Under-two[all] AND Leanness[all]) OR (Under-two[all] AND Thinness[all]) OR (Under-two[all] AND Slimness[all]) OR (Under-two[all] AND Overweight[all]) OR (Under-two[all] AND Obes*[all]) OR (Under-two[all] AND Height*[all]) OR (Under-two[all] AND Weight*[all]) OR (Under-two[all] AND Length[all]) OR (Under-two[all] AND “failure to thrive”[all]) OR (“under two”[all] AND Growth[all]) OR (“under two”[all] AND “Growth Retardation”[all]) OR (“under two”[all] AND “Growth Disorder*”[all]) OR (“under two”[all] AND “Growth Falter*”[all]) OR (“under two”[all] AND “Growth Deficit*”[all]) OR (“under two”[all] AND “Growth failure”[all]) OR (“under two”[all] AND “Growth Trajectory”[all]) OR (“under two”[all] AND “Nutrition Disorder*”[all]) OR (“under two”[all] AND Malnutrition[all]) OR (“under two”[all] AND Undernutrition[all]) OR (“under two”[all] AND Under-nutrition[all]) OR (“under two”[all] AND Undernourish*[all]) OR (“under two”[all] AND Overnutrition[all]) OR (“under two”[all] AND Underweight[all]) OR (“under two”[all] AND Leanness[all]) OR (“under two”[all] AND Thinness[all]) OR (“under two”[all] AND Slimness[all]) OR (“under two”[all] AND Overweight[all]) OR (“under two”[all] AND Obes*[all]) OR (“under two”[all] AND Height*[all]) OR (“under two”[all] AND Weight*[all]) OR (“under two”[all] AND Length[all]) OR (“under two”[all] AND “failure to thrive”[all]) OR (“under 2”[all] AND Growth[all]) OR (“under 2”[all] AND “Growth Retardation”[all]) OR (“under 2”[all] AND “Growth Disorder*”[all]) OR (“under 2”[all] AND “Growth Falter*”[all]) OR (“under 2”[all] AND “Growth Deficit*”[all]) OR (“under 2”[all] AND “Growth failure”[all]) OR (“under 2”[all] AND “Growth Trajectory”[all]) OR (“under 2”[all] AND “Nutrition Disorder*”[all]) OR (“under 2”[all] AND Malnutrition[all]) OR (“under 2”[all] AND Undernutrition[all]) OR (“under 2”[all] AND Under-nutrition[all]) OR (“under 2”[all] AND Undernourish*[all]) OR (“under 2”[all] AND Overnutrition[all]) OR (“under 2”[all] AND Underweight[all]) OR (“under 2”[all] AND Leanness[all]) OR (“under 2”[all] AND Thinness[all]) OR (“under 2”[all] AND Slimness[all]) OR (“under 2”[all] AND Overweight[all]) OR (“under 2”[all] AND Obes*[all]) OR (“under 2”[all] AND Height*[all]) OR (“under 2”[all] AND Weight*[all]) OR (“under 2”[all] AND Length[all]) OR (“under 2”[all] AND “failure to thrive”[all]) OR (Under-2[all] AND Growth[all]) OR (Under-2[all] AND “Growth Retardation”[all]) OR (Under-2[all] AND “Growth Disorder*”[all]) OR (Under-2[all] AND “Growth Falter*”[all]) OR (Under-2[all] AND “Growth Deficit*”[all]) OR (Under-2[all] AND “Growth failure”[all]) OR (Under-2[all] AND “Growth Trajectory”[all]) OR (Under-2[all] AND “Nutrition Disorder*”[all]) OR (Under-2[all] AND Malnutrition[all]) OR (Under-2[all] AND Undernutrition[all]) OR (Under-2[all] AND Under-nutrition[all]) OR (Under-2[all] AND Undernourish*[all]) OR (Under-2[all] AND Overnutrition[all]) OR (Under-2[all] AND Underweight[all]) OR (Under-2[all] AND Leanness[all]) OR (Under-2[all] AND Thinness[all]) OR (Under-2[all] AND Slimness[all]) OR (Under-2[all] AND Overweight[all]) OR (Under-2[all] AND Obes*[all]) OR (Under-2[all] AND Height*[all]) OR (Under-2[all] AND Weight*[all]) OR (Under-2[all] AND Length[all]) OR (Under-2[all] AND “failure to thrive”[all]) OR (Offspring[all] AND Growth[all]) OR (Offspring[all] AND “Growth Retardation”[all]) OR (Offspring[all] AND “Growth Disorder*”[all]) OR (Offspring[all] AND “Growth Falter*”[all]) OR (Offspring[all] AND “Growth Deficit*”[all]) OR (Offspring[all] AND “Growth failure”[all]) OR (Offspring[all] AND “Growth				

	Trajectory”[all]) OR (Offspring[all] AND “Nutrition Disorder*”[all]) OR (Offspring[all] AND Malnutrition[all]) OR (Offspring[all] AND Undernutrition[all]) OR (Offspring[all] AND Under-nutrition[all]) OR (Offspring[all] AND Undernourish*[all]) OR (Offspring[all] AND Overnutrition[all]) OR (Offspring[all] AND Underweight[all]) OR (Offspring[all] AND Leanness[all]) OR (Offspring[all] AND Thinness[all]) OR (Offspring[all] AND Slimness[all]) OR (Offspring[all] AND Overweight[all]) OR (Offspring[all] AND Obes*[all]) OR (Offspring[all] AND Height*[all]) OR (Offspring[all] AND Weight*[all]) OR (Offspring[all] AND Length[all]) OR (Offspring[all] AND “failure to thrive”[all]) OR (Early-life[all] AND Growth[all]) OR (Early-life[all] AND “Growth Retardation”[all]) OR (Early-life[all] AND “Growth Disorder*”[all]) OR (Early-life[all] AND “Growth Falter*”[all]) OR (Early-life[all] AND “Growth Deficit*”[all]) OR (Early-life[all] AND “Growth failure”[all]) OR (Early-life[all] AND “Growth Trajectory”[all]) OR (Early-life[all] AND “Nutrition Disorder*”[all]) OR (Early-life[all] AND Malnutrition[all]) OR (Early-life[all] AND Undernutrition[all]) OR (Early-life[all] AND Under-nutrition[all]) OR (Early-life[all] AND Undernourish*[all]) OR (Early-life[all] AND Overnutrition[all]) OR (Early-life[all] AND Underweight[all]) OR (Early-life[all] AND Leanness[all]) OR (Early-life[all] AND Thinness[all]) OR (Early-life[all] AND Slimness[all]) OR (Early-life[all] AND Overweight[all]) OR (Early-life[all] AND Obes*[all]) OR (Early-life[all] AND Height*[all]) OR (Early-life[all] AND Weight*[all]) OR (Early-life[all] AND Length[all]) OR (Early-life[all] AND “failure to thrive”[all]) OR (“Early life”[all] AND Growth[all]) OR (“Early life”[all] AND “Growth Retardation”[all]) OR (“Early life”[all] AND “Growth Disorder*”[all]) OR (“Early life”[all] AND “Growth Falter*”[all]) OR (“Early life”[all] AND “Growth Deficit*”[all]) OR (“Early life”[all] AND “Growth failure”[all]) OR (“Early life”[all] AND “Growth Trajectory”[all]) OR (“Early life”[all] AND “Nutrition Disorder*”[all]) OR (“Early life”[all] AND Malnutrition[all]) OR (“Early life”[all] AND Undernutrition[all]) OR (“Early life”[all] AND Under-nutrition[all]) OR (“Early life”[all] AND Undernourish*[all]) OR (“Early life”[all] AND Overnutrition[all]) OR (“Early life”[all] AND Underweight[all]) OR (“Early life”[all] AND Leanness[all]) OR (“Early life”[all] AND Thinness[all]) OR (“Early life”[all] AND Slimness[all]) OR (“Early life”[all] AND Overweight[all]) OR (“Early life”[all] AND Obes*[all]) OR (“Early life”[all] AND Height*[all]) OR (“Early life”[all] AND Weight*[all]) OR (“Early life”[all] AND Length[all]) OR (“Early life”[all] AND “failure to thrive”[all]) OR (Pediatric[all] AND Growth[all]) OR (Pediatric[all] AND “Growth Retardation”[all]) OR (Pediatric[all] AND “Growth Disorder*”[all]) OR (Pediatric[all] AND “Growth Falter*”[all]) OR (Pediatric[all] AND “Growth Deficit*”[all]) OR (Pediatric[all] AND “Growth failure”[all]) OR (Pediatric[all] AND “Growth Trajectory”[all]) OR (Pediatric[all] AND “Nutrition Disorder*”[all]) OR (Pediatric[all] AND Malnutrition[all]) OR (Pediatric[all] AND Undernutrition[all]) OR (Pediatric[all] AND Under-nutrition[all]) OR (Pediatric[all] AND Undernourish*[all]) OR (Pediatric[all] AND Overnutrition[all]) OR (Pediatric[all] AND Underweight[all]) OR (Pediatric[all] AND Leanness[all]) OR (Pediatric[all] AND Thinness[all]) OR (Pediatric[all] AND Slimness[all]) OR (Pediatric[all] AND Overweight[all]) OR (Pediatric[all] AND Obes*[all]) OR (Pediatric[all] AND Height*[all]) OR (Pediatric[all] AND Weight*[all]) OR (Pediatric[all] AND Length[all]) OR (Pediatric[all] AND “failure to thrive”[all]) OR “child health”[all])<br>AND<br>(cohort[tiab] OR longitudinal[tiab] OR (Cohort[tiab] AND Study[tiab]) OR (Concurrent[tiab] AND Study[tiab]) OR “birth cohort”[tiab] OR (cohort[tiab] AND analysis[tiab]) OR (cohort[tiab] AND analyses[tiab]) OR “incidence study”[tiab] OR “Follow Up Study”[tiab] OR “Follow-Up Study”[tiab] OR “Followup Study”[tiab] OR (Longitudinal[tiab] AND Study[tiab]) OR (Prospective[tiab] AND study[tiab]))<br>AND<br>(1990/1/01:2024/1/31[dp]))				
2	(((“Educational Status”[tiab] AND Maternal[tiab]) OR (Status[tiab] AND “Maternal Educational”[tiab]) OR “Maternal Educational Status”[tiab] OR (Maternal[all] AND education[all]) OR (Maternal[all] AND Illiteracy[all]) OR (Maternal[all] AND Literacy[all]) OR (maternal[all] AND “schooling”[all]) OR (mother*[all] AND education[all]) OR (mother*[all] AND Illiteracy[all]) OR (mother*[all] AND Literacy[all]) OR (mother*[all] AND “schooling”[all]) OR (parent*[all] AND Illiteracy[all]) OR (parent*[all] AND Literacy[all]) OR (parent*[all] AND education[all]) OR (parent*[all] AND “schooling”[all]) OR (parent*[tiab] AND socio-economic[tiab]) OR (parent*[tiab] AND socioeconomic[tiab]) OR (parent*[tiab] AND “Social Class*”[tiab]) OR (parent*[tiab] AND Socio-demographic[tiab]) OR (parent*[tiab] AND Sociodemographic[tiab]) OR (parent*[tiab] AND Socio-cultural[tiab]) OR (parent*[tiab] AND Sociocultural[tiab]) OR (mother*[tiab] AND socio-economic[tiab]) OR (mother*[tiab] AND socioeconomic[tiab]) OR (mother*[tiab] AND “Social Class*”[tiab]) OR (mother*[tiab] AND Socio-demographic[tiab]) OR (mother*[tiab] AND Sociodemographic[tiab]) OR (mother*[tiab] AND Socio-cultural[tiab]) OR (mother*[tiab] AND Sociocultural[tiab]) OR (maternal[tiab] AND socio-economic[tiab]) OR (maternal[tiab] AND socioeconomic[tiab]) OR (maternal[tiab] AND “Social Class*”[tiab]) OR (maternal[tiab] AND Socio-demographic[tiab]) OR (maternal[tiab] AND Sociodemographic[tiab]) OR (maternal[tiab] AND Socio-cultural[tiab]) OR (maternal[tiab] AND Sociocultural[tiab]) OR “maternal socioeconomic status”[tiab] OR “parental socioeconomic status”[tiab])<br>AND<br>((Child*[tiab] AND Growth[tiab]) OR (Child*[tiab] AND “Growth Retardation”[tiab]) OR (Child*[tiab] AND “Growth Disorder*”[tiab]) OR (Child*[tiab] AND “Growth Falter*”[tiab]) OR (Child*[tiab] AND “Growth Deficit*”[tiab]) OR (Child*[tiab] AND “Growth failure”[tiab]) OR (Child*[tiab] AND “Growth Trajectory”[tiab]) OR Stunting*[all] OR “Stunted Growth”[all] OR (Growth[tiab] AND Stunted[tiab]) OR “short stature”[tiab] OR wast*[all] OR (Child*[tiab] AND	100	11,285		

	“Nutrition Disorder*”[tiab]) OR (Child*[tiab] AND Malnutrition[tiab]) OR (Child*[tiab] AND Undernutrition[tiab]) OR (Child*[tiab] AND Under-nutrition[tiab]) OR (Child*[tiab] AND Undernourish*[tiab]) OR (Child*[tiab] AND Overnutrition[tiab]) OR (Child*[all] AND Underweight[all]) OR (Child*[tiab] AND Leanness[tiab]) OR (Child*[tiab] AND Thinness[tiab]) OR (Child*[tiab] AND Slimness[tiab]) OR (Child*[tiab] AND Overweight[tiab]) OR (Child*[tiab] AND Obes*[tiab]) OR (Child*[all] AND Height*[all]) OR (Child*[all] AND Weight*[all]) OR (Child*[all] AND Length[all]) OR Anthropometric*[tiab] OR Length-for-age[all] OR Height-for-age[all] OR Weight-for-age[all] OR Weight-for-height[all] OR weight-for-length[all] OR “Body mass index-for-age”[all] OR BMI-for-age[all] OR (child*[tiab] AND “failure to thrive”[tiab]) OR (Infan*[tiab] AND Growth[tiab]) OR (Infan*[tiab] AND “Growth Retardation”[tiab]) OR (Infan*[tiab] AND “Growth Disorder*”[tiab]) OR (Infan*[tiab] AND “Growth Falter*”[tiab]) OR (Infan*[tiab] AND “Growth Deficit*”[tiab]) OR (Infan*[tiab] AND “Growth failure”[tiab]) OR (Infan*[tiab] AND “Growth Trajectory”[tiab]) OR (Infan*[tiab] AND “Nutrition Disorder*”[tiab]) OR (Infan*[tiab] AND Malnutrition[tiab]) OR (Infan*[tiab] AND Undernutrition[tiab]) OR (Infan*[tiab] AND Under-nutrition[tiab]) OR (Infan*[tiab] AND Undernourish*[tiab]) OR (Infan*[tiab] AND Overnutrition[tiab]) OR (Infan*[all] AND Underweight[all]) OR (Infan*[tiab] AND Leanness[tiab]) OR (Infan*[tiab] AND Thinness[tiab]) OR (Infan*[tiab] AND Slimness[tiab]) OR (Infan*[tiab] AND Overweight[tiab]) OR (Infan*[tiab] AND Obes*[tiab]) OR (Infan*[all] AND Height*[all]) OR (Infan*[all] AND Weight*[all]) OR (Infan*[all] AND Length[all]) OR (infan*[tiab] AND “failure to thrive”[tiab]) OR (Under-five[tiab] AND Growth[tiab]) OR (Under-five[tiab] AND “Growth Retardation”[tiab]) OR (Under-five[tiab] AND “Growth Disorder*”[tiab]) OR (Under-five[tiab] AND “Growth Falter*”[tiab]) OR (Under-five[tiab] AND “Growth Deficit*”[tiab]) OR (Under-five[tiab] AND “Growth failure”[tiab]) OR (Under-five[tiab] AND “Growth Trajectory”[tiab]) OR (Under-five[tiab] AND “Nutrition Disorder*”[tiab]) OR (Under-five[tiab] AND Malnutrition[tiab]) OR (Under-five[tiab] AND Undernutrition[tiab]) OR (Under-five[tiab] AND Under-nutrition[tiab]) OR (Under-five[tiab] AND Undernourish*[tiab]) OR (Under-five[tiab] AND Overnutrition[tiab]) OR (Under-five[all] AND Underweight[all]) OR (Under-five[tiab] AND Leanness[tiab]) OR (Under-five[tiab] AND Thinness[tiab]) OR (Under-five[tiab] AND Slimness[tiab]) OR (Under-five[tiab] AND Overweight[tiab]) OR (Under-five[tiab] AND Obes*[tiab]) OR (Under-five[all] AND Height*[all]) OR (Under-five[all] AND Weight*[all]) OR (Under-five[all] AND Length[all]) OR (under-five[tiab] AND “failure to thrive”[tiab]) OR (“under five”[tiab] AND Growth[tiab]) OR (“under five”[tiab] AND “Growth Retardation”[tiab]) OR (“under five”[tiab] AND “Growth Disorder*”[tiab]) OR (“under five”[tiab] AND “Growth Falter*”[tiab]) OR (“under five”[tiab] AND “Growth Deficit*”[tiab]) OR (“under five”[tiab] AND “Growth failure”[tiab]) OR (“under five”[tiab] AND “Growth Trajectory”[tiab]) OR (“under five”[tiab] AND “Nutrition Disorder*”[tiab]) OR (“under five”[tiab] AND Malnutrition[tiab]) OR (“under five”[tiab] AND Undernutrition[tiab]) OR (“under five”[tiab] AND Under-nutrition[tiab]) OR (“under five”[tiab] AND Undernourish*[tiab]) OR (“under five”[tiab] AND Overnutrition[tiab]) OR (“under five”[all] AND Underweight[all]) OR (“under five”[tiab] AND Leanness[tiab]) OR (“under five”[tiab] AND Thinness[tiab]) OR (“under five”[tiab] AND Slimness[tiab]) OR (“under five”[tiab] AND Overweight[tiab]) OR (“under five”[tiab] AND Obes*[tiab]) OR (“under five”[all] AND Height*[all]) OR (“under five”[all] AND Weight*[all]) OR (“under five”[all] AND Length[all]) OR (“under five”[tiab] AND “failure to thrive”[tiab]) OR (“under 5”[tiab] AND Growth[tiab]) OR (“under 5”[tiab] AND “Growth Retardation”[tiab]) OR (“under 5”[tiab] AND “Growth Disorder*”[tiab]) OR (“under 5”[tiab] AND “Growth Falter*”[tiab]) OR (“under 5”[tiab] AND “Growth Deficit*”[tiab]) OR (“under 5”[tiab] AND “Growth failure”[tiab])OR (“under 5”[tiab] AND “Growth Trajectory”[tiab]) OR (“under 5”[tiab] AND “Nutrition Disorder*”[tiab]) OR (“under 5”[tiab] AND Malnutrition[tiab]) OR (“under 5”[tiab] AND Undernutrition[tiab]) OR (“under 5”[tiab] AND Under-nutrition[tiab]) OR (“under 5”[tiab] AND Undernourish*[tiab]) OR (“under 5”[tiab] AND Overnutrition[tiab]) OR (“under 5”[all] AND Underweight[all]) OR (“under 5”[tiab] AND Leanness[tiab]) OR (“under 5”[tiab] AND Thinness[tiab]) OR (“under 5”[tiab] AND Slimness[tiab]) OR (“under 5”[tiab] AND Overweight[tiab]) OR (“under 5”[tiab] AND Obes*[tiab]) OR (“under 5”[all] AND Height*[all]) OR (“under 5”[all] AND Weight*[all]) OR (“under 5”[all] AND Length[all]) OR (“under 5”[tiab] AND “failure to thrive”[tiab]) OR (Under-5[tiab] AND Growth[tiab]) OR (Under-5[tiab] AND “Growth Retardation”[tiab]) OR (Under-5[tiab] AND “Growth Disorder*”[tiab]) OR (Under-5[tiab] AND “Growth Falter*”[tiab]) OR (Under-5[tiab] AND “Growth Deficit*”[tiab]) OR (Under-5[tiab] AND “Growth failure”[tiab]) OR (Under-5[tiab] AND “Growth Trajectory”[tiab]) OR (Under-5[tiab] AND “Nutrition Disorder*”[tiab]) OR (Under-5[tiab] AND Malnutrition[tiab]) OR (Under-5[tiab] AND Undernutrition[tiab]) OR (Under-5[tiab] AND Under-nutrition[tiab]) OR (Under-5[tiab] AND Undernourish*[tiab]) OR (Under-5[tiab] AND Overnutrition[tiab]) OR (Under-5[all] AND Underweight[all]) OR (Under-5[tiab] AND Leanness[tiab]) OR (Under-5[tiab] AND Thinness[tiab]) OR (Under-5[tiab] AND Slimness[tiab]) OR (Under-5[tiab] AND Overweight[tiab]) OR (Under-5[tiab] AND Obes*[tiab]) OR (Under-5[all] AND Height*[all]) OR (Under-5[all] AND Weight*[all]) OR (Under-5[all] AND Length[all]) OR (Under-5[tiab] AND “failure to thrive”[tiab]) OR (Under-two[tiab] AND Growth[tiab]) OR (Under-two[tiab] AND “Growth Retardation”[tiab]) OR (Under-two[tiab] AND “Growth Disorder*”[tiab]) OR (Under-two[tiab] AND “Growth Falter*”[tiab]) OR (Under-two[tiab] AND “Growth Deficit*”[tiab]) OR (Under-two[tiab] AND “Growth failure”[tiab]) OR (Under-two[tiab] AND “Growth Trajectory”[tiab]) OR (Under-two[tiab] AND “Nutrition Disorder*”[tiab]) OR (Under-two[tiab] AND Malnutrition[tiab]) OR (Under-two[tiab] AND Undernutrition[tiab]) OR (Under-two[tiab] AND Under-nutrition[tiab]) OR (Under-two[tiab] AND Undernourish*[tiab]) OR (Under-two[tiab] AND Overnutrition[tiab]) OR (Under-two[all] AND Underweight[all]) OR (Under-two[tiab] AND Leanness[tiab]) OR (Under-two[tiab] AND Thinness[tiab]) OR (Under-two[tiab]				

	AND Slimness[tiab]) OR (Under-two[tiab] AND Overweight[tiab]) OR (Under-two[tiab] AND Obes*[tiab]) OR (Under-two[all] AND Height*[all]) OR (Under-two[all] AND Weight*[all]) OR (Under-two[all] AND Length[all]) OR (Under-two[tiab] AND “failure to thrive”[tiab]) OR (“under two”[tiab] AND Growth[tiab]) OR (“under two”[tiab] AND “Growth Retardation”[tiab]) OR (“under two”[tiab] AND “Growth Disorder*”[tiab]) OR (“under two”[tiab] AND “Growth Falter*”[tiab]) OR (“under two”[tiab] AND “Growth Deficit*”[tiab]) OR (“under two”[tiab] AND “Growth failure”[tiab]) OR (“under two”[tiab] AND “Growth Trajectory”[tiab]) OR (“under two”[tiab] AND “Nutrition Disorder*”[tiab]) OR (“under two”[tiab] AND Malnutrition[tiab]) OR (“under two”[tiab] AND Undernutrition[tiab]) OR (“under two”[tiab] AND Under-nutrition[tiab]) OR (“under two”[tiab] AND Undernourish*[tiab]) OR (“under two”[tiab] AND Overnutrition[tiab]) OR (“under two”[all] AND Underweight[all]) OR (“under two”[tiab] AND Leanness[tiab]) OR (“under two”[tiab] AND Thinness[tiab]) OR (“under two”[tiab] AND Slimness[tiab]) OR (“under two”[tiab] AND Overweight[tiab]) OR (“under two”[tiab] AND Obes*[tiab]) OR (“under two”[all] AND Height*[all]) OR (“under two”[all] AND Weight*[all]) OR (“under two”[all] AND Length[all]) OR (“under two”[tiab] AND “failure to thrive”[tiab]) OR (“under 2”[tiab] AND Growth[tiab]) OR (“under 2”[tiab] AND “Growth Retardation”[tiab]) OR (“under 2”[tiab] AND “Growth Disorder*”[tiab]) OR (“under 2”[tiab] AND “Growth Falter*”[tiab]) OR (“under 2”[tiab] AND “Growth Deficit*”[tiab]) OR (“under 2”[tiab] AND “Growth failure”[tiab]) OR (“under 2”[tiab] AND “Growth Trajectory”[tiab]) OR (“under 2”[tiab] AND “Nutrition Disorder*”[tiab]) OR (“under 2”[tiab] AND Malnutrition[tiab]) OR (“under 2”[tiab] AND Undernutrition[tiab]) OR (“under 2”[tiab] AND Under-nutrition[tiab]) OR (“under 2”[tiab] AND Undernourish*[tiab]) OR (“under 2”[tiab] AND Overnutrition[tiab]) OR (“under 2”[all] AND Underweight[all]) OR (“under 2”[tiab] AND Leanness[tiab]) OR (“under 2”[tiab] AND Thinness[tiab]) OR (“under 2”[tiab] AND Slimness[tiab]) OR (“under 2”[tiab] AND Overweight[tiab]) OR (“under 2”[tiab] AND Obes*[tiab]) OR (“under 2”[all] AND Height*[all]) OR (“under 2”[all] AND Weight*[all]) OR (“under 2”[all] AND Length[all]) OR (“under 2”[tiab] AND “failure to thrive”[tiab]) OR (Under-2[tiab] AND Growth[tiab]) OR (Under-2[tiab] AND “Growth Retardation”[tiab]) OR (Under-2[tiab] AND “Growth Disorder*”[tiab]) OR (Under-2[tiab] AND “Growth Falter*”[tiab]) OR (Under-2[tiab] AND “Growth Deficit*”[tiab]) OR (Under-2[tiab] AND “Growth failure”[tiab]) OR (Under-2[tiab] AND “Growth Trajectory”[tiab]) OR (Under-2[tiab] AND “Nutrition Disorder*”[tiab]) OR (Under-2[tiab] AND Malnutrition[tiab]) OR (Under-2[tiab] AND Undernutrition[tiab]) OR (Under-2[tiab] AND Under-nutrition[tiab]) OR (Under-2[tiab] AND Undernourish*[tiab]) OR (Under-2[tiab] AND Overnutrition[tiab]) OR (Under-2[all] AND Underweight[all]) OR (Under-2[tiab] AND Leanness[tiab]) OR (Under-2[tiab] AND Thinness[tiab]) OR (Under-2[tiab] AND Slimness[tiab]) OR (Under-2[tiab] AND Overweight[tiab]) OR (Under-2[tiab] AND Obes*[tiab]) OR (Under-2[all] AND Height*[all]) OR (Under-2[all] AND Weight*[all]) OR (Under-2[all] AND Length[all]) OR (Under-2[tiab] AND “failure to thrive”[tiab]) OR (Offspring[tiab] AND Growth[tiab]) OR (Offspring[tiab] AND “Growth Retardation”[tiab]) OR (Offspring[tiab] AND “Growth Disorder*”[tiab]) OR (Offspring[tiab] AND “Growth Falter*”[tiab]) OR (Offspring[tiab] AND “Growth Deficit*”[tiab]) OR (Offspring[tiab] AND “Growth failure”[tiab]) OR (Offspring[tiab] AND “Growth Trajectory”[tiab]) OR (Offspring[tiab] AND “Nutrition Disorder*”[tiab]) OR (Offspring[tiab] AND Malnutrition[tiab]) OR (Offspring[tiab] AND Undernutrition[tiab]) OR (Offspring[tiab] AND Under-nutrition[tiab]) OR (Offspring[tiab] AND Undernourish*[tiab]) OR (Offspring[tiab] AND Overnutrition[tiab]) OR (Offspring[all] AND Underweight[all]) OR (Offspring[tiab] AND Leanness[tiab]) OR (Offspring[tiab] AND Thinness[tiab]) OR (Offspring[tiab] AND Slimness[tiab]) OR (Offspring[tiab] AND Overweight[tiab]) OR (Offspring[tiab] AND Obes*[tiab]) OR (Offspring[all] AND Height*[all]) OR (Offspring[all] AND Weight*[all]) OR (Offspring[all] AND Length[all]) OR (Offspring[tiab] AND “failure to thrive”[tiab]) OR (Early-life[tiab] AND Growth[tiab]) OR (Early-life[tiab] AND “Growth Retardation”[tiab]) OR (Early-life[tiab] AND “Growth Disorder*”[tiab]) OR (Early-life[tiab] AND “Growth Falter*”[tiab]) OR (Early-life[tiab] AND “Growth Deficit*”[tiab]) OR (Early-life[tiab] AND “Growth failure”[tiab]) OR (Early-life[tiab] AND “Growth Trajectory”[tiab]) OR (Early-life[tiab] AND “Nutrition Disorder*”[tiab]) OR (Early-life[tiab] AND Malnutrition[tiab]) OR (Early-life[tiab] AND Undernutrition[tiab]) OR (Early-life[tiab] AND Under-nutrition[tiab]) OR (Early-life[tiab] AND Undernourish*[tiab]) OR (Early-life[tiab] AND Overnutrition[tiab]) OR (Early-life[all] AND Underweight[all]) OR (Early-life[tiab] AND Leanness[tiab]) OR (Early-life[tiab] AND Thinness[tiab]) OR (Early-life[tiab] AND Slimness[tiab]) OR (Early-life[tiab] AND Overweight[tiab]) OR (Early-life[tiab] AND Obes*[tiab]) OR (Early-life[all] AND Height*[all]) OR (Early-life[all] AND Weight*[all]) OR (Early-life[all] AND Length[all]) OR (Early-life[tiab] AND “failure to thrive”[tiab]) OR (“Early life”[tiab] AND Growth[tiab]) OR (“Early life”[tiab] AND “Growth Retardation”[tiab]) OR (“Early life”[tiab] AND “Growth Disorder*”[tiab]) OR (“Early life”[tiab] AND “Growth Falter*”[tiab]) OR (“Early life”[tiab] AND “Growth Deficit*”[tiab]) OR (“Early life”[tiab] AND “Growth failure”[tiab]) OR (“Early life”[tiab] AND “Growth Trajectory”[tiab]) OR (“Early life”[tiab] AND “Nutrition Disorder*”[tiab]) OR (“Early life”[tiab] AND Malnutrition[tiab]) OR (“Early life”[tiab] AND Undernutrition[tiab]) OR (“Early life”[tiab] AND Under-nutrition[tiab]) OR (“Early life”[tiab] AND Undernourish*[tiab]) OR (“Early life”[tiab] AND Overnutrition[tiab]) OR (“Early life”[all] AND Underweight[all]) OR (“Early life”[tiab] AND Leanness[tiab]) OR (“Early life”[tiab] AND Thinness[tiab]) OR (“Early life”[tiab] AND Slimness[tiab]) OR (“Early life”[tiab] AND Overweight[tiab]) OR (“Early life”[tiab] AND Obes*[tiab]) OR (“Early life”[all] AND Height*[all]) OR (“Early life”[all] AND Weight*[all]) OR (“Early life”[all] AND Length[all]) OR (“Early life”[tiab] AND “failure to thrive”[tiab]) OR (Pediatric[tiab] AND Growth[tiab]) OR (Pediatric[tiab] AND “Growth Retardation”[tiab]) OR (Pediatric[tiab] AND “Growth Disorder*”[tiab]) OR (Pediatric[tiab] AND “Growth Falter*”[tiab])				

	OR (Pediatric[tiab] AND “Growth Deficit*”[tiab]) OR (Pediatric[tiab] AND “Growth failure”[tiab]) OR (Pediatric[tiab] AND “Growth Trajectory”[tiab]) OR (Pediatric[tiab] AND “Nutrition Disorder*”[tiab]) OR (Pediatric[tiab] AND Malnutrition[tiab]) OR (Pediatric[tiab] AND Undernutrition[tiab]) OR (Pediatric[tiab] AND Under-nutrition[tiab]) OR (Pediatric[tiab] AND Undernourish*[tiab]) OR (Pediatric[tiab] AND Overnutrition[tiab]) OR (Pediatric[all] AND Underweight[all]) OR (Pediatric[tiab] AND Leanness[tiab]) OR (Pediatric[tiab] AND Thinness[tiab]) OR (Pediatric[tiab] AND Slimness[tiab]) OR (Pediatric[tiab] AND Overweight[tiab]) OR (Pediatric[tiab] AND Obes*[tiab]) OR (Pediatric[all] AND Height*[all]) OR (Pediatric[all] AND Weight*[all]) OR (Pediatric[all] AND Length[all]) OR (Pediatric[tiab] AND “failure to thrive”[tiab]) OR “child health”[all])<br>AND<br>(cohort[tiab] OR longitudinal[tiab] OR (Cohort[tiab] AND Study[tiab]) OR (Concurrent[tiab] AND Study[tiab]) OR “birth cohort”[tiab] OR (cohort[tiab] AND analysis[tiab]) OR (cohort[tiab] AND analyses[tiab]) OR “incidence study”[tiab] OR “Follow Up Study”[tiab] OR “Follow-Up Study”[tiab] OR “Followup Study”[tiab] OR (Longitudinal[tiab] AND Study[tiab]) OR (Prospective[tiab] AND study[tiab]))<br>AND<br>(1990/1/01:2024/1/31[dp]))				
3	(((“Educational Status”[tiab] AND Maternal[tiab]) OR (Status[tiab] AND “Maternal Educational”[tiab]) OR “Maternal Educational Status”[tiab] OR (Maternal[all] AND education[all]) OR (Maternal[all] AND Illiteracy[all]) OR (Maternal[all] AND Literacy[all]) OR (maternal[all] AND “schooling”[all]) OR (mother*[all] AND education[all]) OR (mother*[all] AND Illiteracy[all]) OR (mother*[all] AND Literacy[all]) OR (mother*[all] AND “schooling”[all]) OR (parent*[all] AND Illiteracy[all]) OR (parent*[all] AND Literacy[all]) OR (parent*[all] AND education[all]) OR (parent*[all] AND “schooling”[all]) OR (parent*[tiab] AND socio-economic[tiab]) OR (parent*[tiab] AND socioeconomic[tiab]) OR (parent*[tiab] AND “Social Class*”[tiab]) OR (parent*[tiab] AND Socio-demographic[tiab]) OR (parent*[tiab] AND Sociodemographic[tiab]) OR (parent*[tiab] AND Socio-cultural[tiab]) OR (parent*[tiab] AND Sociocultural[tiab]) OR (mother*[tiab] AND socio-economic[tiab]) OR (mother*[tiab] AND socioeconomic[tiab]) OR (mother*[tiab] AND “Social Class*”[tiab]) OR (mother*[tiab] AND Socio-demographic[tiab]) OR (mother*[tiab] AND Sociodemographic[tiab]) OR (mother*[tiab] AND Socio-cultural[tiab]) OR (mother*[tiab] AND Sociocultural[tiab]) OR (maternal[tiab] AND socio-economic[tiab]) OR (maternal[tiab] AND socioeconomic[tiab]) OR (maternal[tiab] AND “Social Class*”[tiab]) OR (maternal[tiab] AND Socio-demographic[tiab]) OR (maternal[tiab] AND Sociodemographic[tiab]) OR (maternal[tiab] AND Socio-cultural[tiab]) OR (maternal[tiab] AND Sociocultural[tiab]) OR “maternal socioeconomic status”[tiab] OR “parental socioeconomic status”[tiab])<br>AND<br>((Child*[tiab] AND Growth[tiab]) OR (Child*[tiab] AND “Growth Retardation”[tiab]) OR (Child*[tiab] AND “Growth Disorder*”[tiab]) OR (Child*[tiab] AND “Growth Falter*”[tiab]) OR (Child*[tiab] AND “Growth Deficit*”[tiab]) OR (Child*[tiab] AND “Growth failure”[tiab]) OR (Child*[tiab] AND “Growth Trajectory”[tiab]) OR Stunting*[all] OR “Stunted Growth”[all] OR (Growth[tiab] AND Stunted[tiab]) OR “short stature”[tiab] OR wast*[all] OR (Child*[tiab] AND “Nutrition Disorder*”[tiab]) OR (Child*[tiab] AND Malnutrition[tiab]) OR (Child*[tiab] AND Undernutrition[tiab]) OR (Child*[tiab] AND Under-nutrition[tiab]) OR (Child*[tiab] AND Undernourish*[tiab]) OR (Child*[tiab] AND Overnutrition[tiab]) OR (Child*[all] AND Underweight[all]) OR (Child*[tiab] AND Leanness[tiab]) OR (Child*[tiab] AND Thinness[tiab]) OR (Child*[tiab] AND Slimness[tiab]) OR (Child*[tiab] AND Overweight[tiab]) OR (Child*[tiab] AND Obes*[tiab]) OR (Child*[all] AND Height*[all]) OR (Child*[all] AND Weight*[all]) OR (Child*[all] AND Length[all]) OR Anthropometric*[tiab] OR Length-for-age[all] OR Height-for-age[all] OR Weight-for-age[all] OR Weight-for-height[all] OR weight-for-length[all] OR “Body mass index-for-age”[all] OR BMI-for-age[all] OR (child*[tiab] AND “failure to thrive”[tiab]) OR (Infan*[tiab] AND Growth[tiab]) OR (Infan*[tiab] AND “Growth Retardation”[tiab]) OR (Infan*[tiab] AND “Growth Disorder*”[tiab]) OR (Infan*[tiab] AND “Growth Falter*”[tiab]) OR (Infan*[tiab] AND “Growth Deficit*”[tiab]) OR (Infan*[tiab] AND “Growth failure”[tiab]) OR (Infan*[tiab] AND “Growth Trajectory”[tiab]) OR (Infan*[tiab] AND “Nutrition Disorder*”[tiab]) OR (Infan*[tiab] AND Malnutrition[tiab]) OR (Infan*[tiab] AND Undernutrition[tiab]) OR (Infan*[tiab] AND Under-nutrition[tiab]) OR (Infan*[tiab] AND Undernourish*[tiab]) OR (Infan*[tiab] AND Overnutrition[tiab]) OR (Infan*[all] AND Underweight[all]) OR (Infan*[tiab] AND Leanness[tiab]) OR (Infan*[tiab] AND Thinness[tiab]) OR (Infan*[tiab] AND Slimness[tiab]) OR (Infan*[tiab] AND Overweight[tiab]) OR (Infan*[tiab] AND Obes*[tiab]) OR (Infan*[all] AND Height*[all]) OR (Infan*[all] AND Weight*[all]) OR (Infan*[all] AND Length[all]) OR (infan*[tiab] AND “failure to thrive”[tiab]) OR (Under-five[tiab] AND Growth[tiab]) OR (Under-five[tiab] AND “Growth Retardation”[tiab]) OR (Under-five[tiab] AND “Growth Disorder*”[tiab]) OR (Under-five[tiab] AND “Growth Falter*”[tiab]) OR (Under-five[tiab] AND “Growth Deficit*”[tiab]) OR (Under-five[tiab] AND “Growth failure”[tiab]) OR (Under-five[tiab] AND “Growth Trajectory”[tiab]) OR (Under-five[tiab] AND “Nutrition Disorder*”[tiab]) OR (Under-five[tiab] AND Malnutrition[tiab]) OR (Under-five[tiab] AND Undernutrition[tiab]) OR (Under-five[tiab] AND Under-nutrition[tiab]) OR (Under-five[tiab] AND Undernourish*[tiab]) OR (Under-five[tiab] AND Overnutrition[tiab]) OR (Under-five[all] AND Underweight[all]) OR (Under-five[tiab] AND Leanness[tiab]) OR (Under-five[tiab] AND Thinness[tiab]) OR (Under-five[tiab] AND Slimness[tiab]) OR (Under-five[tiab] AND Overweight[tiab]) OR (Under-five[tiab] AND Obes*[tiab]) OR (Under-five[all] AND Height*[all]) OR (Under-five[all] AND Weight*[all]) OR (Under-five[all] AND Length[all]) OR	50	9,340		

	(under-five[tiab] AND “failure to thrive”[tiab]) OR (“under five”[tiab] AND Growth[tiab]) OR (“under five”[tiab] AND “Growth Retardation”[tiab]) OR (“under five”[tiab] AND “Growth Disorder*”[tiab]) OR (“under five”[tiab] AND “Growth Falter*”[tiab]) OR (“under five”[tiab] AND “Growth Deficit*”[tiab]) OR (“under five”[tiab] AND “Growth failure”[tiab]) OR (“under five”[tiab] AND “Growth Trajectory”[tiab]) OR (“under five”[tiab] AND “Nutrition Disorder*”[tiab]) OR (“under five”[tiab] AND Malnutrition[tiab]) OR (“under five”[tiab] AND Undernutrition[tiab]) OR (“under five”[tiab] AND Under-nutrition[tiab]) OR (“under five”[tiab] AND Undernourish*[tiab]) OR (“under five”[tiab] AND Overnutrition[tiab]) OR (“under five”[all] AND Underweight[all]) OR (“under five”[tiab] AND Leanness[tiab]) OR (“under five”[tiab] AND Thinness[tiab]) OR (“under five”[tiab] AND Slimness[tiab]) OR (“under five”[tiab] AND Overweight[tiab]) OR (“under five”[tiab] AND Obes*[tiab]) OR (“under five”[all] AND Height*[all]) OR (“under five”[all] AND Weight*[all]) OR (“under five”[all] AND Length[all]) OR (“under five”[tiab] AND “failure to thrive”[tiab]) OR (“under 5”[tiab] AND Growth[tiab]) OR (“under 5”[tiab] AND “Growth Retardation”[tiab]) OR (“under 5”[tiab] AND “Growth Disorder*”[tiab]) OR (“under 5”[tiab] AND “Growth Falter*”[tiab]) OR (“under 5”[tiab] AND “Growth Deficit*”[tiab]) OR (“under 5”[tiab] AND “Growth failure”[tiab])OR (“under 5”[tiab] AND “Growth Trajectory”[tiab]) OR (“under 5”[tiab] AND “Nutrition Disorder*”[tiab]) OR (“under 5”[tiab] AND Malnutrition[tiab]) OR (“under 5”[tiab] AND Undernutrition[tiab]) OR (“under 5”[tiab] AND Under-nutrition[tiab]) OR (“under 5”[tiab] AND Undernourish*[tiab]) OR (“under 5”[tiab] AND Overnutrition[tiab]) OR (“under 5”[all] AND Underweight[all]) OR (“under 5”[tiab] AND Leanness[tiab]) OR (“under 5”[tiab] AND Thinness[tiab]) OR (“under 5”[tiab] AND Slimness[tiab]) OR (“under 5”[tiab] AND Overweight[tiab]) OR (“under 5”[tiab] AND Obes*[tiab]) OR (“under 5”[all] AND Height*[all]) OR (“under 5”[all] AND Weight*[all]) OR (“under 5”[all] AND Length[all]) OR (“under 5”[tiab] AND “failure to thrive”[tiab]) OR (Under-5[tiab] AND Growth[tiab]) OR (Under-5[tiab] AND “Growth Retardation”[tiab]) OR (Under-5[tiab] AND “Growth Disorder*”[tiab]) OR (Under-5[tiab] AND “Growth Falter*”[tiab]) OR (Under-5[tiab] AND “Growth Deficit*”[tiab]) OR (Under-5[tiab] AND “Growth failure”[tiab]) OR (Under-5[tiab] AND “Growth Trajectory”[tiab]) OR (Under-5[tiab] AND “Nutrition Disorder*”[tiab]) OR (Under-5[tiab] AND Malnutrition[tiab]) OR (Under-5[tiab] AND Undernutrition[tiab]) OR (Under-5[tiab] AND Under-nutrition[tiab]) OR (Under-5[tiab] AND Undernourish*[tiab]) OR (Under-5[tiab] AND Overnutrition[tiab]) OR (Under-5[all] AND Underweight[all]) OR (Under-5[tiab] AND Leanness[tiab]) OR (Under-5[tiab] AND Thinness[tiab]) OR (Under-5[tiab] AND Slimness[tiab]) OR (Under-5[tiab] AND Overweight[tiab]) OR (Under-5[tiab] AND Obes*[tiab]) OR (Under-5[all] AND Height*[all]) OR (Under-5[all] AND Weight*[all]) OR (Under-5[all] AND Length[all]) OR (Under-5[tiab] AND “failure to thrive”[tiab]) OR (Under-two[tiab] AND Growth[tiab]) OR (Under-two[tiab] AND “Growth Retardation”[tiab]) OR (Under-two[tiab] AND “Growth Disorder*”[tiab]) OR (Under-two[tiab] AND “Growth Falter*”[tiab]) OR (Under-two[tiab] AND “Growth Deficit*”[tiab]) OR (Under-two[tiab] AND “Growth failure”[tiab]) OR (Under-two[tiab] AND “Growth Trajectory”[tiab]) OR (Under-two[tiab] AND “Nutrition Disorder*”[tiab]) OR (Under-two[tiab] AND Malnutrition[tiab]) OR (Under-two[tiab] AND Undernutrition[tiab]) OR (Under-two[tiab] AND Under-nutrition[tiab]) OR (Under-two[tiab] AND Undernourish*[tiab]) OR (Under-two[tiab] AND Overnutrition[tiab]) OR (Under-two[all] AND Underweight[all]) OR (Under-two[tiab] AND Leanness[tiab]) OR (Under-two[tiab] AND Thinness[tiab]) OR (Under-two[tiab] AND Slimness[tiab]) OR (Under-two[tiab] AND Overweight[tiab]) OR (Under-two[tiab] AND Obes*[tiab]) OR (Under-two[all] AND Height*[all]) OR (Under-two[all] AND Weight*[all]) OR (Under-two[all] AND Length[all]) OR (Under-two[tiab] AND “failure to thrive”[tiab]) OR (“under two”[tiab] AND Growth[tiab]) OR (“under two”[tiab] AND “Growth Retardation”[tiab]) OR (“under two”[tiab] AND “Growth Disorder*”[tiab]) OR (“under two”[tiab] AND “Growth Falter*”[tiab]) OR (“under two”[tiab] AND “Growth Deficit*”[tiab]) OR (“under two”[tiab] AND “Growth failure”[tiab]) OR (“under two”[tiab] AND “Growth Trajectory”[tiab]) OR (“under two”[tiab] AND “Nutrition Disorder*”[tiab]) OR (“under two”[tiab] AND Malnutrition[tiab]) OR (“under two”[tiab] AND Undernutrition[tiab]) OR (“under two”[tiab] AND Under-nutrition[tiab]) OR (“under two”[tiab] AND Undernourish*[tiab]) OR (“under two”[tiab] AND Overnutrition[tiab]) OR (“under two”[all] AND Underweight[all]) OR (“under two”[tiab] AND Leanness[tiab]) OR (“under two”[tiab] AND Thinness[tiab]) OR (“under two”[tiab] AND Slimness[tiab]) OR (“under two”[tiab] AND Overweight[tiab]) OR (“under two”[tiab] AND Obes*[tiab]) OR (“under two”[all] AND Height*[all]) OR (“under two”[all] AND Weight*[all]) OR (“under two”[all] AND Length[all]) OR (“under two”[tiab] AND “failure to thrive”[tiab]) OR (“under 2”[tiab] AND Growth[tiab]) OR (“under 2”[tiab] AND “Growth Retardation”[tiab]) OR (“under 2”[tiab] AND “Growth Disorder*”[tiab]) OR (“under 2”[tiab] AND “Growth Falter*”[tiab]) OR (“under 2”[tiab] AND “Growth Deficit*”[tiab]) OR (“under 2”[tiab] AND “Growth failure”[tiab]) OR (“under 2”[tiab] AND “Growth Trajectory”[tiab]) OR (“under 2”[tiab] AND “Nutrition Disorder*”[tiab]) OR (“under 2”[tiab] AND Malnutrition[tiab]) OR (“under 2”[tiab] AND Undernutrition[tiab]) OR (“under 2”[tiab] AND Under-nutrition[tiab]) OR (“under 2”[tiab] AND Undernourish*[tiab]) OR (“under 2”[tiab] AND Overnutrition[tiab]) OR (“under 2”[all] AND Underweight[all]) OR (“under 2”[tiab] AND Leanness[tiab]) OR (“under 2”[tiab] AND Thinness[tiab]) OR (“under 2”[tiab] AND Slimness[tiab]) OR (“under 2”[tiab] AND Overweight[tiab]) OR (“under 2”[tiab] AND Obes*[tiab]) OR (“under 2”[all] AND Height*[all]) OR (“under 2”[all] AND Weight*[all]) OR (“under 2”[all] AND Length[all]) OR (“under 2”[tiab] AND “failure to thrive”[tiab]) OR (Under-2[tiab] AND Growth[tiab]) OR (Under-2[tiab] AND “Growth Retardation”[tiab]) OR (Under-2[tiab] AND “Growth Disorder*”[tiab]) OR (Under-2[tiab] AND “Growth Falter*”[tiab]) OR (Under-2[tiab] AND “Growth Deficit*”[tiab]) OR (Under-2[tiab] AND “Growth failure”[tiab]) OR (Under-2[tiab] AND “Growth Trajectory”[tiab]) OR (Under-2[tiab]				

	AND “Nutrition Disorder*”[tiab]) OR (Under-2[tiab] AND Malnutrition[tiab]) OR (Under-2[tiab] AND Undernutrition[tiab]) OR (Under-2[tiab] AND Under-nutrition[tiab]) OR (Under-2[tiab] AND Undernourish*[tiab]) OR (Under-2[tiab] AND Overnutrition[tiab]) OR (Under-2[all] AND Underweight[all]) OR (Under-2[tiab] AND Leanness[tiab]) OR (Under-2[tiab] AND Thinness[tiab]) OR (Under-2[tiab] AND Slimness[tiab]) OR (Under-2[tiab] AND Overweight[tiab]) OR (Under-2[tiab] AND Obes*[tiab]) OR (Under-2[all] AND Height*[all]) OR (Under-2[all] AND Weight*[all]) OR (Under-2[all] AND Length[all]) OR (Under-2[tiab] AND “failure to thrive”[tiab]) OR (Offspring[tiab] AND Growth[tiab]) OR (Offspring[tiab] AND “Growth Retardation”[tiab]) OR (Offspring[tiab] AND “Growth Disorder*”[tiab]) OR (Offspring[tiab] AND “Growth Falter*”[tiab]) OR (Offspring[tiab] AND “Growth Deficit*”[tiab]) OR (Offspring[tiab] AND “Growth failure”[tiab]) OR (Offspring[tiab] AND “Growth Trajectory”[tiab]) OR (Offspring[tiab] AND “Nutrition Disorder*”[tiab]) OR (Offspring[tiab] AND Malnutrition[tiab]) OR (Offspring[tiab] AND Undernutrition[tiab]) OR (Offspring[tiab] AND Under-nutrition[tiab]) OR (Offspring[tiab] AND Undernourish*[tiab]) OR (Offspring[tiab] AND Overnutrition[tiab]) OR (Offspring[all] AND Underweight[all]) OR (Offspring[tiab] AND Leanness[tiab]) OR (Offspring[tiab] AND Thinness[tiab]) OR (Offspring[tiab] AND Slimness[tiab]) OR (Offspring[tiab] AND Overweight[tiab]) OR (Offspring[tiab] AND Obes*[tiab]) OR (Offspring[all] AND Height*[all]) OR (Offspring[all] AND Weight*[all]) OR (Offspring[all] AND Length[all]) OR (Offspring[tiab] AND “failure to thrive”[tiab]) OR (Early-life[tiab] AND Growth[tiab]) OR (Early-life[tiab] AND “Growth Retardation”[tiab]) OR (Early-life[tiab] AND “Growth Disorder*”[tiab]) OR (Early-life[tiab] AND “Growth Falter*”[tiab]) OR (Early-life[tiab] AND “Growth Deficit*”[tiab]) OR (Early-life[tiab] AND “Growth failure”[tiab]) OR (Early-life[tiab] AND “Growth Trajectory”[tiab]) OR (Early-life[tiab] AND “Nutrition Disorder*”[tiab]) OR (Early-life[tiab] AND Malnutrition[tiab]) OR (Early-life[tiab] AND Undernutrition[tiab]) OR (Early-life[tiab] AND Under-nutrition[tiab]) OR (Early-life[tiab] AND Undernourish*[tiab]) OR (Early-life[tiab] AND Overnutrition[tiab]) OR (Early-life[all] AND Underweight[all]) OR (Early-life[tiab] AND Leanness[tiab]) OR (Early-life[tiab] AND Thinness[tiab]) OR (Early-life[tiab] AND Slimness[tiab]) OR (Early-life[tiab] AND Overweight[tiab]) OR (Early-life[tiab] AND Obes*[tiab]) OR (Early-life[all] AND Height*[all]) OR (Early-life[all] AND Weight*[all]) OR (Early-life[all] AND Length[all]) OR (Early-life[tiab] AND “failure to thrive”[tiab]) OR (“Early life”[tiab] AND Growth[tiab]) OR (“Early life”[tiab] AND “Growth Retardation”[tiab]) OR (“Early life”[tiab] AND “Growth Disorder*”[tiab]) OR (“Early life”[tiab] AND “Growth Falter*”[tiab]) OR (“Early life”[tiab] AND “Growth Deficit*”[tiab]) OR (“Early life”[tiab] AND “Growth failure”[tiab]) OR (“Early life”[tiab] AND “Growth Trajectory”[tiab]) OR (“Early life”[tiab] AND “Nutrition Disorder*”[tiab]) OR (“Early life”[tiab] AND Malnutrition[tiab]) OR (“Early life”[tiab] AND Undernutrition[tiab]) OR (“Early life”[tiab] AND Under-nutrition[tiab]) OR (“Early life”[tiab] AND Undernourish*[tiab]) OR (“Early life”[tiab] AND Overnutrition[tiab]) OR (“Early life”[all] AND Underweight[all]) OR (“Early life”[tiab] AND Leanness[tiab]) OR (“Early life”[tiab] AND Thinness[tiab]) OR (“Early life”[tiab] AND Slimness[tiab]) OR (“Early life”[tiab] AND Overweight[tiab]) OR (“Early life”[tiab] AND Obes*[tiab]) OR (“Early life”[all] AND Height*[all]) OR (“Early life”[all] AND Weight*[all]) OR (“Early life”[all] AND Length[all]) OR (“Early life”[tiab] AND “failure to thrive”[tiab]) OR (Pediatric[tiab] AND Growth[tiab]) OR (Pediatric[tiab] AND “Growth Retardation”[tiab]) OR (Pediatric[tiab] AND “Growth Disorder*”[tiab]) OR (Pediatric[tiab] AND “Growth Falter*”[tiab]) OR (Pediatric[tiab] AND “Growth Deficit*”[tiab]) OR (Pediatric[tiab] AND “Growth failure”[tiab]) OR (Pediatric[tiab] AND “Growth Trajectory”[tiab]) OR (Pediatric[tiab] AND “Nutrition Disorder*”[tiab]) OR (Pediatric[tiab] AND Malnutrition[tiab]) OR (Pediatric[tiab] AND Undernutrition[tiab]) OR (Pediatric[tiab] AND Under-nutrition[tiab]) OR (Pediatric[tiab] AND Undernourish*[tiab]) OR (Pediatric[tiab] AND Overnutrition[tiab]) OR (Pediatric[all] AND Underweight[all]) OR (Pediatric[tiab] AND Leanness[tiab]) OR (Pediatric[tiab] AND Thinness[tiab]) OR (Pediatric[tiab] AND Slimness[tiab]) OR (Pediatric[tiab] AND Overweight[tiab]) OR (Pediatric[tiab] AND Obes*[tiab]) OR (Pediatric[all] AND Height*[all]) OR (Pediatric[all] AND Weight*[all]) OR (Pediatric[all] AND Length[all]) OR (Pediatric[tiab] AND “failure to thrive”[tiab]) OR “child health”[tiab])<br>AND<br>(cohort[tiab] OR longitudinal[tiab] OR (Cohort[tiab] AND Study[tiab]) OR (Concurrent[tiab] AND Study[tiab]) OR “birth cohort”[tiab] OR (cohort[tiab] AND analysis[tiab]) OR (cohort[tiab] AND analyses[tiab]) OR “incidence study”[tiab] OR “Follow Up Study”[tiab] OR “Follow-Up Study”[tiab] OR “Followup Study”[tiab] OR (Longitudinal[tiab] AND Study[tiab]) OR (Prospective[tiab] AND study[tiab]))<br>AND<br>(1990/1/01:2024/1/31[dp]))				
4	(((“Educational Status”[tiab] AND Maternal[tiab]) OR (Status[tiab] AND “Maternal Educational”[tiab]) OR “Maternal Educational Status”[tiab] OR (Maternal[all] AND education[all]) OR (Maternal[all] AND Illiteracy[all]) OR (Maternal[all] AND Literacy[all]) OR (maternal[all] AND “schooling”[all]) OR (mother*[all] AND education[all]) OR (mother*[all] AND Illiteracy[all]) OR (mother*[all] AND Literacy[all]) OR (mother*[all] AND “schooling”[all]) OR (parent*[all] AND Illiteracy[all]) OR (parent*[all] AND Literacy[all]) OR (parent*[all] AND education[all]) OR (parent*[all] AND “schooling”[all]) OR (parent*[tiab] AND socio-economic[tiab]) OR (parent*[tiab] AND socioeconomic[tiab]) OR (parent*[tiab] AND “Social Class*”[tiab]) OR (parent*[tiab] AND Socio-demographic[tiab]) OR (parent*[tiab] AND Sociodemographic[tiab]) OR (parent*[tiab] AND Socio-cultural[tiab]) OR (parent*[tiab] AND Sociocultural[tiab]) OR (mother*[tiab] AND socio-economic[tiab]) OR (mother*[tiab] AND socioeconomic[tiab]) OR (mother*[tiab] AND “Social Class*”[tiab]) OR (mother*[tiab] AND	50	8,037		

	Socio-demographic[tiab]) OR (mother*[tiab] AND Sociodemographic[tiab]) OR (mother*[tiab] AND Socio-cultural[tiab]) OR (mother*[tiab] AND Sociocultural[tiab]) OR (maternal[tiab] AND socio-economic[tiab]) OR (maternal[tiab] AND socioeconomic[tiab]) OR (maternal[tiab] AND “Social Class*”[tiab]) OR (maternal[tiab] AND Socio-demographic[tiab]) OR (maternal[tiab] AND Sociodemographic[tiab]) OR (maternal[tiab] AND Socio-cultural[tiab]) OR (maternal[tiab] AND Sociocultural[tiab]) OR “maternal socioeconomic status”[tiab] OR “parental socioeconomic status”[tiab])<br>AND<br>((Child*[tiab] AND Growth[tiab]) OR (Child*[tiab] AND “Growth Retardation”[tiab]) OR (Child*[tiab] AND “Growth Disorder*”[tiab]) OR (Child*[tiab] AND “Growth Falter*”[tiab]) OR (Child*[tiab] AND “Growth Deficit*”[tiab]) OR (Child*[tiab] AND “Growth failure”[tiab]) OR (Child*[tiab] AND “Growth Trajectory”[tiab]) OR Stunting*[tiab] OR “Stunted Growth”[tiab] OR (Growth[tiab] AND Stunted[tiab]) OR “short stature”[tiab] OR wast*[tiab] OR (Child*[tiab] AND “Nutrition Disorder*”[tiab]) OR (Child*[tiab] AND Malnutrition[tiab]) OR (Child*[tiab] AND Undernutrition[tiab]) OR (Child*[tiab] AND Under-nutrition[tiab]) OR (Child*[tiab] AND Undernourish*[tiab]) OR (Child*[tiab] AND Overnutrition[tiab]) OR (Child*[tiab] AND Underweight[tiab]) OR (Child*[tiab] AND Leanness[tiab]) OR (Child*[tiab] AND Thinness[tiab]) OR (Child*[tiab] AND Slimness[tiab]) OR (Child*[tiab] AND Overweight[tiab]) OR (Child*[tiab] AND Obes*[tiab]) OR (Child*[tiab] AND Height*[tiab]) OR (Child*[tiab] AND Weight*[tiab]) OR (Child*[tiab] AND Length[tiab]) OR Anthropometric*[tiab] OR Length-for-age[all] OR Height-for-age[all] OR Weight-for-age[all] OR Weight-for-height[all] OR weight-for-length[all] OR “Body mass index-for-age”[all] OR BMI-for-age[all] OR (child*[tiab] AND “failure to thrive”[tiab]) OR (Infan*[tiab] AND Growth[tiab]) OR (Infan*[tiab] AND “Growth Retardation”[tiab]) OR (Infan*[tiab] AND “Growth Disorder*”[tiab]) OR (Infan*[tiab] AND “Growth Falter*”[tiab]) OR (Infan*[tiab] AND “Growth Deficit*”[tiab]) OR (Infan*[tiab] AND “Growth failure”[tiab]) OR (Infan*[tiab] AND “Growth Trajectory”[tiab]) OR (Infan*[tiab] AND “Nutrition Disorder*”[tiab]) OR (Infan*[tiab] AND Malnutrition[tiab]) OR (Infan*[tiab] AND Undernutrition[tiab]) OR (Infan*[tiab] AND Under-nutrition[tiab]) OR (Infan*[tiab] AND Undernourish*[tiab]) OR (Infan*[tiab] AND Overnutrition[tiab]) OR (Infan*[tiab] AND Underweight[tiab]) OR (Infan*[tiab] AND Leanness[tiab]) OR (Infan*[tiab] AND Thinness[tiab]) OR (Infan*[tiab] AND Slimness[tiab]) OR (Infan*[tiab] AND Overweight[tiab]) OR (Infan*[tiab] AND Obes*[tiab]) OR (Infan*[tiab] AND Height*[tiab]) OR (Infan*[tiab] AND Weight*[tiab]) OR (Infan*[tiab] AND Length[tiab]) OR (infan*[tiab] AND “failure to thrive”[tiab]) OR (Under-five[tiab] AND Growth[tiab]) OR (Under-five[tiab] AND “Growth Retardation”[tiab]) OR (Under-five[tiab] AND “Growth Disorder*”[tiab]) OR (Under-five[tiab] AND “Growth Falter*”[tiab]) OR (Under-five[tiab] AND “Growth Deficit*”[tiab]) OR (Under-five[tiab] AND “Growth failure”[tiab]) OR (Under-five[tiab] AND “Growth Trajectory”[tiab]) OR (Under-five[tiab] AND “Nutrition Disorder*”[tiab]) OR (Under-five[tiab] AND Malnutrition[tiab]) OR (Under-five[tiab] AND Undernutrition[tiab]) OR (Under-five[tiab] AND Under-nutrition[tiab]) OR (Under-five[tiab] AND Undernourish*[tiab]) OR (Under-five[tiab] AND Overnutrition[tiab]) OR (Under-five[tiab] AND Underweight[tiab]) OR (Under-five[tiab] AND Leanness[tiab]) OR (Under-five[tiab] AND Thinness[tiab]) OR (Under-five[tiab] AND Slimness[tiab]) OR (Under-five[tiab] AND Overweight[tiab]) OR (Under-five[tiab] AND Obes*[tiab]) OR (Under-five[tiab] AND Height*[tiab]) OR (Under-five[tiab] AND Weight*[tiab]) OR (Under-five[tiab] AND Length[tiab]) OR (under-five[tiab] AND “failure to thrive”[tiab]) OR (“under five”[tiab] AND Growth[tiab]) OR (“under five”[tiab] AND “Growth Retardation”[tiab]) OR (“under five”[tiab] AND “Growth Disorder*”[tiab]) OR (“under five”[tiab] AND “Growth Falter*”[tiab]) OR (“under five”[tiab] AND “Growth Deficit*”[tiab]) OR (“under five”[tiab] AND “Growth failure”[tiab]) OR (“under five”[tiab] AND “Growth Trajectory”[tiab]) OR (“under five”[tiab] AND “Nutrition Disorder*”[tiab]) OR (“under five”[tiab] AND Malnutrition[tiab]) OR (“under five”[tiab] AND Undernutrition[tiab]) OR (“under five”[tiab] AND Under-nutrition[tiab]) OR (“under five”[tiab] AND Undernourish*[tiab]) OR (“under five”[tiab] AND Overnutrition[tiab]) OR (“under five”[tiab] AND Underweight[tiab]) OR (“under five”[tiab] AND Leanness[tiab]) OR (“under five”[tiab] AND Thinness[tiab]) OR (“under five”[tiab] AND Slimness[tiab]) OR (“under five”[tiab] AND Overweight[tiab]) OR (“under five”[tiab] AND Obes*[tiab]) OR (“under five”[tiab] AND Height*[tiab]) OR (“under five”[tiab] AND Weight*[tiab]) OR (“under five”[tiab] AND Length[tiab]) OR (“under five”[tiab] AND “failure to thrive”[tiab]) OR (“under 5”[tiab] AND Growth[tiab]) OR (“under 5”[tiab] AND “Growth Retardation”[tiab]) OR (“under 5”[tiab] AND “Growth Disorder*”[tiab]) OR (“under 5”[tiab] AND “Growth Falter*”[tiab]) OR (“under 5”[tiab] AND “Growth Deficit*”[tiab]) OR (“under 5”[tiab] AND “Growth failure”[tiab])OR (“under 5”[tiab] AND “Growth Trajectory”[tiab]) OR (“under 5”[tiab] AND “Nutrition Disorder*”[tiab]) OR (“under 5”[tiab] AND Malnutrition[tiab]) OR (“under 5”[tiab] AND Undernutrition[tiab]) OR (“under 5”[tiab] AND Under-nutrition[tiab]) OR (“under 5”[tiab] AND Undernourish*[tiab]) OR (“under 5”[tiab] AND Overnutrition[tiab]) OR (“under 5”[tiab] AND Underweight[tiab]) OR (“under 5”[tiab] AND Leanness[tiab]) OR (“under 5”[tiab] AND Thinness[tiab]) OR (“under 5”[tiab] AND Slimness[tiab]) OR (“under 5”[tiab] AND Overweight[tiab]) OR (“under 5”[tiab] AND Obes*[tiab]) OR (“under 5”[tiab] AND Height*[tiab]) OR (“under 5”[tiab] AND Weight*[tiab]) OR (“under 5”[tiab] AND Length[tiab]) OR (“under 5”[tiab] AND “failure to thrive”[tiab]) OR (Under-5[tiab] AND Growth[tiab]) OR (Under-5[tiab] AND “Growth Retardation”[tiab]) OR (Under-5[tiab] AND “Growth Disorder*”[tiab]) OR (Under-5[tiab] AND “Growth Falter*”[tiab]) OR (Under-5[tiab] AND “Growth Deficit*”[tiab]) OR (Under-5[tiab] AND “Growth failure”[tiab]) OR (Under-5[tiab] AND “Growth Trajectory”[tiab]) OR (Under-5[tiab] AND “Nutrition Disorder*”[tiab]) OR (Under-5[tiab] AND Malnutrition[tiab]) OR (Under-5[tiab] AND Undernutrition[tiab]) OR (Under-5[tiab] AND Under-nutrition[tiab]) OR (Under-5[tiab] AND				

	Undernourish*[tiab]) OR (Under-5[tiab] AND Overnutrition[tiab]) OR (Under-5[tiab] AND Underweight[tiab]) OR (Under-5[tiab] AND Leanness[tiab]) OR (Under-5[tiab] AND Thinness[tiab]) OR (Under-5[tiab] AND Slimness[tiab]) OR (Under-5[tiab] AND Overweight[tiab]) OR (Under-5[tiab] AND Obes*[tiab]) OR (Under-5[tiab] AND Height*[tiab]) OR (Under-5[tiab] AND Weight*[tiab]) OR (Under-5[tiab] AND Length[tiab]) OR (Under-5[tiab] AND “failure to thrive”[tiab]) OR (Under-two[tiab] AND Growth[tiab]) OR (Under-two[tiab] AND “Growth Retardation”[tiab]) OR (Under-two[tiab] AND “Growth Disorder*”[tiab]) OR (Under-two[tiab] AND “Growth Falter*”[tiab]) OR (Under-two[tiab] AND “Growth Deficit*”[tiab]) OR (Under-two[tiab] AND “Growth failure”[tiab]) OR (Under-two[tiab] AND “Growth Trajectory”[tiab]) OR (Under-two[tiab] AND “Nutrition Disorder*”[tiab]) OR (Under-two[tiab] AND Malnutrition[tiab]) OR (Under-two[tiab] AND Undernutrition[tiab]) OR (Under-two[tiab] AND Under-nutrition[tiab]) OR (Under-two[tiab] AND Undernourish*[tiab]) OR (Under-two[tiab] AND Overnutrition[tiab]) OR (Under-two[tiab] AND Underweight[tiab]) OR (Under-two[tiab] AND Leanness[tiab]) OR (Under-two[tiab] AND Thinness[tiab]) OR (Under-two[tiab] AND Slimness[tiab]) OR (Under-two[tiab] AND Overweight[tiab]) OR (Under-two[tiab] AND Obes*[tiab]) OR (Under-two[tiab] AND Height*[tiab]) OR (Under-two[tiab] AND Weight*[tiab]) OR (Under-two[tiab] AND Length[tiab]) OR (Under-two[tiab] AND “failure to thrive”[tiab]) OR (“under two”[tiab] AND Growth[tiab]) OR (“under two”[tiab] AND “Growth Retardation”[tiab]) OR (“under two”[tiab] AND “Growth Disorder*”[tiab]) OR (“under two”[tiab] AND “Growth Falter*”[tiab]) OR (“under two”[tiab] AND “Growth Deficit*”[tiab]) OR (“under two”[tiab] AND “Growth failure”[tiab]) OR (“under two”[tiab] AND “Growth Trajectory”[tiab]) OR (“under two”[tiab] AND “Nutrition Disorder*”[tiab]) OR (“under two”[tiab] AND Malnutrition[tiab]) OR (“under two”[tiab] AND Undernutrition[tiab]) OR (“under two”[tiab] AND Under-nutrition[tiab]) OR (“under two”[tiab] AND Undernourish*[tiab]) OR (“under two”[tiab] AND Overnutrition[tiab]) OR (“under two”[tiab] AND Underweight[tiab]) OR (“under two”[tiab] AND Leanness[tiab]) OR (“under two”[tiab] AND Thinness[tiab]) OR (“under two”[tiab] AND Slimness[tiab]) OR (“under two”[tiab] AND Overweight[tiab]) OR (“under two”[tiab] AND Obes*[tiab]) OR (“under two”[tiab] AND Height*[tiab]) OR (“under two”[tiab] AND Weight*[tiab]) OR (“under two”[tiab] AND Length[tiab]) OR (“under two”[tiab] AND “failure to thrive”[tiab]) OR (“under 2”[tiab] AND Growth[tiab]) OR (“under 2”[tiab] AND “Growth Retardation”[tiab]) OR (“under 2”[tiab] AND “Growth Disorder*”[tiab]) OR (“under 2”[tiab] AND “Growth Falter*”[tiab]) OR (“under 2”[tiab] AND “Growth Deficit*”[tiab]) OR (“under 2”[tiab] AND “Growth failure”[tiab]) OR (“under 2”[tiab] AND “Growth Trajectory”[tiab]) OR (“under 2”[tiab] AND “Nutrition Disorder*”[tiab]) OR (“under 2”[tiab] AND Malnutrition[tiab]) OR (“under 2”[tiab] AND Undernutrition[tiab]) OR (“under 2”[tiab] AND Under-nutrition[tiab]) OR (“under 2”[tiab] AND Undernourish*[tiab]) OR (“under 2”[tiab] AND Overnutrition[tiab]) OR (“under 2”[tiab] AND Underweight[tiab]) OR (“under 2”[tiab] AND Leanness[tiab]) OR (“under 2”[tiab] AND Thinness[tiab]) OR (“under 2”[tiab] AND Slimness[tiab]) OR (“under 2”[tiab] AND Overweight[tiab]) OR (“under 2”[tiab] AND Obes*[tiab]) OR (“under 2”[tiab] AND Height*[tiab]) OR (“under 2”[tiab] AND Weight*[tiab]) OR (“under 2”[tiab] AND Length[tiab]) OR (“under 2”[tiab] AND “failure to thrive”[tiab]) OR (Under-2[tiab] AND Growth[tiab]) OR (Under-2[tiab] AND “Growth Retardation”[tiab]) OR (Under-2[tiab] AND “Growth Disorder*”[tiab]) OR (Under-2[tiab] AND “Growth Falter*”[tiab]) OR (Under-2[tiab] AND “Growth Deficit*”[tiab]) OR (Under-2[tiab] AND “Growth failure”[tiab]) OR (Under-2[tiab] AND “Growth Trajectory”[tiab]) OR (Under-2[tiab] AND “Nutrition Disorder*”[tiab]) OR (Under-2[tiab] AND Malnutrition[tiab]) OR (Under-2[tiab] AND Undernutrition[tiab]) OR (Under-2[tiab] AND Under-nutrition[tiab]) OR (Under-2[tiab] AND Undernourish*[tiab]) OR (Under-2[tiab] AND Overnutrition[tiab]) OR (Under-2[tiab] AND Underweight[tiab]) OR (Under-2[tiab] AND Leanness[tiab]) OR (Under-2[tiab] AND Thinness[tiab]) OR (Under-2[tiab] AND Slimness[tiab]) OR (Under-2[tiab] AND Overweight[tiab]) OR (Under-2[tiab] AND Obes*[tiab]) OR (Under-2[tiab] AND Height*[tiab]) OR (Under-2[tiab] AND Weight*[tiab]) OR (Under-2[tiab] AND Length[tiab]) OR (Under-2[tiab] AND “failure to thrive”[tiab]) OR (Offspring[tiab] AND Growth[tiab]) OR (Offspring[tiab] AND “Growth Retardation”[tiab]) OR (Offspring[tiab] AND “Growth Disorder*”[tiab]) OR (Offspring[tiab] AND “Growth Falter*”[tiab]) OR (Offspring[tiab] AND “Growth Deficit*”[tiab]) OR (Offspring[tiab] AND “Growth failure”[tiab]) OR (Offspring[tiab] AND “Growth Trajectory”[tiab]) OR (Offspring[tiab] AND “Nutrition Disorder*”[tiab]) OR (Offspring[tiab] AND Malnutrition[tiab]) OR (Offspring[tiab] AND Undernutrition[tiab]) OR (Offspring[tiab] AND Under-nutrition[tiab]) OR (Offspring[tiab] AND Undernourish*[tiab]) OR (Offspring[tiab] AND Overnutrition[tiab]) OR (Offspring[tiab] AND Underweight[tiab]) OR (Offspring[tiab] AND Leanness[tiab]) OR (Offspring[tiab] AND Thinness[tiab]) OR (Offspring[tiab] AND Slimness[tiab]) OR (Offspring[tiab] AND Overweight[tiab]) OR (Offspring[tiab] AND Obes*[tiab]) OR (Offspring[tiab] AND Height*[tiab]) OR (Offspring[tiab] AND Weight*[tiab]) OR (Offspring[tiab] AND Length[tiab]) OR (Offspring[tiab] AND “failure to thrive”[tiab]) OR (Early-life[tiab] AND Growth[tiab]) OR (Early-life[tiab] AND “Growth Retardation”[tiab]) OR (Early-life[tiab] AND “Growth Disorder*”[tiab]) OR (Early-life[tiab] AND “Growth Falter*”[tiab]) OR (Early-life[tiab] AND “Growth Deficit*”[tiab]) OR (Early-life[tiab] AND “Growth failure”[tiab]) OR (Early-life[tiab] AND “Growth Trajectory”[tiab]) OR (Early-life[tiab] AND “Nutrition Disorder*”[tiab]) OR (Early-life[tiab] AND Malnutrition[tiab]) OR (Early-life[tiab] AND Undernutrition[tiab]) OR (Early-life[tiab] AND Under-nutrition[tiab]) OR (Early-life[tiab] AND Undernourish*[tiab]) OR (Early-life[tiab] AND Overnutrition[tiab]) OR (Early-life[tiab] AND Underweight[tiab]) OR (Early-life[tiab] AND Leanness[tiab]) OR (Early-life[tiab] AND Thinness[tiab]) OR (Early-life[tiab] AND Slimness[tiab]) OR (Early-life[tiab] AND Overweight[tiab]) OR (Early-life[tiab] AND Obes*[tiab]) OR (Early-life[tiab] AND Height*[tiab]) OR (Early-life[tiab] AND Weight*[tiab]) OR (Early-life[tiab] AND Length[tiab]) OR (Early-life[tiab]				

	AND “failure to thrive”[tiab]) OR (“Early life”[tiab] AND Growth[tiab]) OR (“Early life”[tiab] AND “Growth Retardation”[tiab]) OR (“Early life”[tiab] AND “Growth Disorder*”[tiab]) OR (“Early life”[tiab] AND “Growth Falter*”[tiab]) OR (“Early life”[tiab] AND “Growth Deficit*”[tiab]) OR (“Early life”[tiab] AND “Growth failure”[tiab]) OR (“Early life”[tiab] AND “Growth Trajectory”[tiab]) OR (“Early life”[tiab] AND “Nutrition Disorder*”[tiab]) OR (“Early life”[tiab] AND Malnutrition[tiab]) OR (“Early life”[tiab] AND Undernutrition[tiab]) OR (“Early life”[tiab] AND Under-nutrition[tiab]) OR (“Early life”[tiab] AND Undernourish*[tiab]) OR (“Early life”[tiab] AND Overnutrition[tiab]) OR (“Early life”[tiab] AND Underweight[tiab]) OR (“Early life”[tiab] AND Leanness[tiab]) OR (“Early life”[tiab] AND Thinness[tiab]) OR (“Early life”[tiab] AND Slimness[tiab]) OR (“Early life”[tiab] AND Overweight[tiab]) OR (“Early life”[tiab] AND Obes*[tiab]) OR (“Early life”[tiab] AND Height*[tiab]) OR (“Early life”[tiab] AND Weight*[tiab]) OR (“Early life”[tiab] AND Length[tiab]) OR (“Early life”[tiab] AND “failure to thrive”[tiab]) OR (Pediatric[tiab] AND Growth[tiab]) OR (Pediatric[tiab] AND “Growth Retardation”[tiab]) OR (Pediatric[tiab] AND “Growth Disorder*”[tiab]) OR (Pediatric[tiab] AND “Growth Falter*”[tiab]) OR (Pediatric[tiab] AND “Growth Deficit*”[tiab]) OR (Pediatric[tiab] AND “Growth failure”[tiab]) OR (Pediatric[tiab] AND “Growth Trajectory”[tiab]) OR (Pediatric[tiab] AND “Nutrition Disorder*”[tiab]) OR (Pediatric[tiab] AND Malnutrition[tiab]) OR (Pediatric[tiab] AND Undernutrition[tiab]) OR (Pediatric[tiab] AND Under-nutrition[tiab]) OR (Pediatric[tiab] AND Undernourish*[tiab]) OR (Pediatric[tiab] AND Overnutrition[tiab]) OR (Pediatric[tiab] AND Underweight[tiab]) OR (Pediatric[tiab] AND Leanness[tiab]) OR (Pediatric[tiab] AND Thinness[tiab]) OR (Pediatric[tiab] AND Slimness[tiab]) OR (Pediatric[tiab] AND Overweight[tiab]) OR (Pediatric[tiab] AND Obes*[tiab]) OR (Pediatric[tiab] AND Height*[tiab]) OR (Pediatric[tiab] AND Weight*[tiab]) OR (Pediatric[tiab] AND Length[tiab]) OR (Pediatric[tiab] AND “failure to thrive”[tiab]) OR “child health”[tiab])<br>AND<br>(cohort[tiab] OR longitudinal[tiab] OR (Cohort[tiab] AND Study[tiab]) OR (Concurrent[tiab] AND Study[tiab]) OR “birth cohort”[tiab] OR (cohort[tiab] AND analysis[tiab]) OR (cohort[tiab] AND analyses[tiab]) OR “incidence study”[tiab] OR “Follow Up Study”[tiab] OR “Follow-Up Study”[tiab] OR “Followup Study”[tiab] OR (Longitudinal[tiab] AND Study[tiab]) OR (Prospective[tiab] AND study[tiab]))<br>AND<br>(1990/1/01:2024/1/31[dp]))				
5	(((“Educational Status”[ti] AND Maternal[ti]) OR (Status[ti] AND “Maternal Educational”[ti]) OR “Maternal Educational Status”[ti] OR (Maternal[all] AND education[all]) OR (Maternal[tiab] AND Illiteracy[tiab]) OR (Maternal[tiab] AND Literacy[tiab]) OR (maternal[all] AND “schooling”[all]) OR (mother*[all] AND education[all]) OR (mother*[tiab] AND Illiteracy[tiab]) OR (mother*[tiab] AND Literacy[tiab]) OR (mother*[all] AND “schooling”[all]) OR (parent*[tiab] AND Illiteracy[tiab]) OR (parent*[tiab] AND Literacy[tiab]) OR (parent*[all] AND education[all]) OR (parent*[all] AND “schooling”[all]) OR (parent*[tiab] AND socio-economic[tiab]) OR (parent*[tiab] AND socioeconomic[tiab]) OR (parent*[ti] AND “Social Class*”[ti]) OR (parent*[tiab] AND Socio-demographic[tiab]) OR (parent*[tiab] AND Sociodemographic[tiab]) OR (parent*[ti] AND Socio-cultural[ti]) OR (parent*[ti] AND Sociocultural[ti]) OR (mother*[tiab] AND socio-economic[tiab]) OR (mother*[tiab] AND socioeconomic[tiab]) OR (mother*[ti] AND “Social Class*”[ti]) OR (mother*[tiab] AND Socio-demographic[tiab]) OR (mother*[tiab] AND Sociodemographic[tiab]) OR (mother*[ti] AND Socio-cultural[ti]) OR (mother*[ti] AND Sociocultural[ti]) OR (maternal[tiab] AND socio-economic[tiab]) OR (maternal[tiab] AND socioeconomic[tiab]) OR (maternal[ti] AND “Social Class*”[ti]) OR (maternal[tiab] AND Socio-demographic[tiab]) OR (maternal[tiab] AND Sociodemographic[tiab]) OR (maternal[ti] AND Socio-cultural[ti]) OR (maternal[ti] AND Sociocultural[ti]) OR “maternal socioeconomic status”[ti] OR “parental socioeconomic status”[ti])<br>AND<br>((Child*[tiab] AND Growth[tiab]) OR (Child*[ti] AND “Growth Retardation”[ti]) OR (Child*[ti] AND “Growth Disorder*”[ti]) OR (Child*[ti] AND “Growth Falter*”[ti]) OR (Child*[ti] AND “Growth Deficit*”[ti]) OR (Child*[ti] AND “Growth failure”[ti]) OR (Child*[ti] AND “Growth Trajectory”[ti]) OR Stunting*[tiab] OR “Stunted Growth”[ti] OR (Growth[ti] AND Stunted[ti]) OR “short stature”[ti] OR wast*[tiab] OR (Child*[ti] AND “Nutrition Disorder*”[ti]) OR (Child*[tiab] AND Malnutrition[tiab]) OR (Child*[ti] AND Undernutrition[ti]) OR (Child*[ti] AND Under-nutrition[ti]) OR (Child*[ti] AND Undernourish*[ti]) OR (Child*[ti] AND Overnutrition[ti]) OR (Child*[ti] AND Underweight[ti]) OR (Child*[ti] AND Leanness[ti]) OR (Child*[ti] AND Thinness[ti]) OR (Child*[ti] AND Slimness[ti]) OR (Child*[ti] AND Overweight[ti]) OR (Child*[ti] AND Obes*[ti]) OR (Child*[tiab] AND Height*[tiab]) OR (Child*[tiab] AND Weight*[tiab]) OR (Child*[tiab] AND Length[tiab]) OR Anthropometric*[tiab] OR Length-for-age[all] OR Height-for-age[all] OR Weight-for-age[all] OR Weight-for-height[all] OR weight-for-length[all] OR “Body mass index-for-age”[all] OR BMI-for-age[all] OR (child*[ti] AND “failure to thrive”[ti]) OR (Infan*[tiab] AND Growth[tiab]) OR (Infan*[ti] AND “Growth Retardation”[ti]) OR (Infan*[ti] AND “Growth Disorder*”[ti]) OR (Infan*[ti] AND “Growth Falter*”[ti]) OR (Infan*[ti] AND “Growth Deficit*”[ti]) OR (Infan*[ti] AND “Growth failure”[ti]) OR (Infan*[ti] AND “Growth Trajectory”[ti]) OR (Infan*[ti] AND “Nutrition Disorder*”[ti]) OR (Infan*[tiab] AND Malnutrition[tiab]) OR (Infan*[ti] AND Undernutrition[ti]) OR (Infan*[ti] AND Under-nutrition[ti]) OR (Infan*[ti] AND Undernourish*[ti]) OR (Infan*[ti] AND Overnutrition[ti]) OR (Infan*[ti] AND Underweight[ti]) OR (Infan*[ti] AND Leanness[ti]) OR (Infan*[ti] AND Thinness[ti]) OR (Infan*[ti] AND Slimness[ti]) OR (Infan*[ti] AND Overweight[ti]) OR (Infan*[ti] AND Obes*[ti]) OR (Infan*[tiab] AND Height*[tiab]) OR (Infan*[tiab] AND Weight*[tiab]) OR (Infan*[tiab] AND Length[tiab]) OR	33	6,747		

	(infan*[ti] AND “failure to thrive”[ti]) OR (Under-five[tiab] AND Growth[tiab]) OR (Under-five[ti] AND “Growth Retardation”[ti]) OR (Under-five[ti] AND “Growth Disorder*”[ti]) OR (Under-five[ti] AND “Growth Falter*”[ti]) OR (Under-five[ti] AND “Growth Deficit*”[ti]) OR (Under-five[ti] AND “Growth failure”[ti]) OR (Under-five[ti] AND “Growth Trajectory”[ti]) OR (Under-five[ti] AND “Nutrition Disorder*”[ti]) OR (Under-five[tiab] AND Malnutrition[tiab]) OR (Under-five[ti] AND Undernutrition[ti]) OR (Under-five[ti] AND Under-nutrition[ti]) OR (Under-five[ti] AND Undernourish*[ti]) OR (Under-five[ti] AND Overnutrition[ti]) OR (Under-five[ti] AND Underweight[ti]) OR (Under-five[tiab] AND Leanness[ti]) OR (Under-five[ti] AND Thinness[ti]) OR (Under-five[ti] AND Slimness[ti]) OR (Under-five[ti] AND Overweight[ti]) OR (Under-five[ti] AND Obes*[ti]) OR (Under-five[tiab] AND Height*[tiab]) OR (Under-five[tiab] AND Weight*[tiab]) OR (Under-five[tiab] AND Length[tiab]) OR (under-five[ti] AND “failure to thrive”[ti]) OR (“under five”[tiab] AND Growth[tiab]) OR (“under five”[ti] AND “Growth Retardation”[ti]) OR (“under five”[ti] AND “Growth Disorder*”[ti]) OR (“under five”[ti] AND “Growth Falter*”[ti]) OR (“under five”[ti] AND “Growth Deficit*”[ti]) OR (“under five”[ti] AND “Growth failure”[ti]) OR (“under five”[ti] AND “Growth Trajectory”[ti]) OR (“under five”[ti] AND “Nutrition Disorder*”[ti]) OR (“under five”[tiab] AND Malnutrition[tiab]) OR (“under five”[ti] AND Undernutrition[ti]) OR (“under five”[ti] AND Under-nutrition[ti]) OR (“under five”[ti] AND Undernourish*[ti]) OR (“under five”[ti] AND Overnutrition[ti]) OR (“under five”[ti] AND Underweight[ti]) OR (“under five”[ti] AND Leanness[ti]) OR (“under five”[ti] AND Thinness[ti]) OR (“under five”[ti] AND Slimness[ti]) OR (“under five”[ti] AND Overweight[ti]) OR (“under five”[ti] AND Obes*[ti]) OR (“under five”[tiab] AND Height*[tiab]) OR (“under five”[tiab] AND Weight*[tiab]) OR (“under five”[tiab] AND Length[tiab]) OR (“under five”[ti] AND “failure to thrive”[ti]) OR (“under 5”[tiab] AND Growth[tiab]) OR (“under 5”[ti] AND “Growth Retardation”[ti]) OR (“under 5”[ti] AND “Growth Disorder*”[ti]) OR (“under 5”[ti] AND “Growth Falter*”[ti]) OR (“under 5”[ti] AND “Growth Deficit*”[ti]) OR (“under 5”[ti] AND “Growth failure”[ti])OR (“under 5”[ti] AND “Growth Trajectory”[ti]) OR (“under 5”[ti] AND “Nutrition Disorder*”[ti]) OR (“under 5”[tiab] AND Malnutrition[tiab]) OR (“under 5”[ti] AND Undernutrition[ti]) OR (“under 5”[ti] AND Under-nutrition[ti]) OR (“under 5”[ti] AND Undernourish*[ti]) OR (“under 5”[ti] AND Overnutrition[ti]) OR (“under 5”[ti] AND Underweight[ti]) OR (“under 5”[ti] AND Leanness[ti]) OR (“under 5”[ti] AND Thinness[ti]) OR (“under 5”[ti] AND Slimness[ti]) OR (“under 5”[ti] AND Overweight[ti]) OR (“under 5”[ti] AND Obes*[ti]) OR (“under 5”[tiab] AND Height*[tiab]) OR (“under 5”[tiab] AND Weight*[tiab]) OR (“under 5”[tiab] AND Length[tiab]) OR (“under 5”[ti] AND “failure to thrive”[ti]) OR (Under-5[tiab] AND Growth[tiab]) OR (Under-5[ti] AND “Growth Retardation”[ti]) OR (Under-5[ti] AND “Growth Disorder*”[ti]) OR (Under-5[ti] AND “Growth Falter*”[ti]) OR (Under-5[ti] AND “Growth Deficit*”[ti]) OR (Under-5[ti] AND “Growth failure”[ti]) OR (Under-5[ti] AND “Growth Trajectory”[ti]) OR (Under-5[ti] AND “Nutrition Disorder*”[ti]) OR (Under-5[tiab] AND Malnutrition[tiab]) OR (Under-5[ti] AND Undernutrition[ti]) OR (Under-5[ti] AND Under-nutrition[ti]) OR (Under-5[ti] AND Undernourish*[ti]) OR (Under-5[ti] AND Overnutrition[ti]) OR (Under-5[ti] AND Underweight[ti]) OR (Under-5[ti] AND Leanness[ti]) OR (Under-5[ti] AND Thinness[ti]) OR (Under-5[ti] AND Slimness[ti]) OR (Under-5[ti] AND Overweight[ti]) OR (Under-5[ti] AND Obes*[ti]) OR (Under-5[tiab] AND Height*[tiab]) OR (Under-5[tiab] AND Weight*[tiab]) OR (Under-5[tiab] AND Length[tiab]) OR (Under-5[ti] AND “failure to thrive”[ti]) OR (Under-two[tiab] AND Growth[tiab]) OR (Under-two[ti] AND “Growth Retardation”[ti]) OR (Under-two[ti] AND “Growth Disorder*”[ti]) OR (Under-two[ti] AND “Growth Falter*”[ti]) OR (Under-two[ti] AND “Growth Deficit*”[ti]) OR (Under-two[ti] AND “Growth failure”[ti]) OR (Under-two[ti] AND “Growth Trajectory”[ti]) OR (Under-two[ti] AND “Nutrition Disorder*”[ti]) OR (Under-two[tiab] AND Malnutrition[tiab]) OR (Under-two[ti] AND Undernutrition[ti]) OR (Under-two[ti] AND Under-nutrition[ti]) OR (Under-two[ti] AND Undernourish*[ti]) OR (Under-two[ti] AND Overnutrition[ti]) OR (Under-two[ti] AND Underweight[ti]) OR (Under-two[ti] AND Leanness[ti]) OR (Under-two[ti] AND Thinness[ti]) OR (Under-two[ti] AND Slimness[ti]) OR (Under-two[tiab] AND Overweight[tiab]) OR (Under-two[ti] AND Obes*[ti]) OR (Under-two[tiab] AND Height*[tiab]) OR (Under-two[tiab] AND Weight*[tiab]) OR (Under-two[tiab] AND Length[tiab]) OR (Under-two[ti] AND “failure to thrive”[ti]) OR (“under two”[tiab] AND Growth[tiab]) OR (“under two”[ti] AND “Growth Retardation”[ti]) OR (“under two”[ti] AND “Growth Disorder*”[ti]) OR (“under two”[ti] AND “Growth Falter*”[ti]) OR (“under two”[ti] AND “Growth Deficit*”[ti]) OR (“under two”[ti] AND “Growth failure”[ti]) OR (“under two”[ti] AND “Growth Trajectory”[ti]) OR (“under two”[ti] AND “Nutrition Disorder*”[ti]) OR (“under two”[tiab] AND Malnutrition[tiab]) OR (“under two”[ti] AND Undernutrition[ti]) OR (“under two”[ti] AND Under-nutrition[ti]) OR (“under two”[ti] AND Undernourish*[ti]) OR (“under two”[ti] AND Overnutrition[ti]) OR (“under two”[ti] AND Underweight[ti]) OR (“under two”[ti] AND Leanness[ti]) OR (“under two”[ti] AND Thinness[ti]) OR (“under two”[ti] AND Slimness[ti]) OR (“under two”[ti] AND Overweight[ti]) OR (“under two”[ti] AND Obes*[ti]) OR (“under two”[tiab] AND Height*[tiab]) OR (“under two”[tiab] AND Weight*[tiab]) OR (“under two”[tiab] AND Length[tiab]) OR (“under two”[ti] AND “failure to thrive”[ti]) OR (“under 2”[tiab] AND Growth[tiab]) OR (“under 2”[ti] AND “Growth Retardation”[ti]) OR (“under 2”[ti] AND “Growth Disorder*”[ti]) OR (“under 2”[ti] AND “Growth Falter*”[ti]) OR (“under 2”[ti] AND “Growth Deficit*”[ti]) OR (“under 2”[ti] AND “Growth failure”[ti]) OR (“under 2”[ti] AND “Growth Trajectory”[ti]) OR (“under 2”[ti] AND “Nutrition Disorder*”[ti]) OR (“under 2”[tiab] AND Malnutrition[tiab]) OR (“under 2”[ti] AND Undernutrition[ti]) OR (“under 2”[ti] AND Under-nutrition[ti]) OR (“under 2”[ti] AND Undernourish*[ti]) OR (“under 2”[ti] AND Overnutrition[ti]) OR (“under 2”[ti] AND Underweight[ti]) OR (“under 2”[ti] AND Leanness[ti]) OR (“under 2”[ti] AND Thinness[ti]) OR (“under 2”[ti] AND Slimness[ti]) OR (“under 2”[ti] AND Overweight[ti]) OR (“under 2”[ti] AND				

	Obes*[ti]) OR (“under 2”[tiab] AND Height*[tiab]) OR (“under 2”[tiab] AND Weight*[tiab]) OR (“under 2”[tiab] AND Length[tiab]) OR (“under 2”[ti] AND “failure to thrive”[ti]) OR (Under-2[tiab] AND Growth[tiab]) OR (Under-2[ti] AND “Growth Retardation”[ti]) OR (Under-2[ti] AND “Growth Disorder*”[ti]) OR (Under-2[ti] AND “Growth Falter*”[ti]) OR (Under-2[ti] AND “Growth Deficit*”[ti]) OR (Under-2[ti] AND “Growth failure”[ti]) OR (Under-2[ti] AND “Growth Trajectory”[ti]) OR (Under-2[ti] AND “Nutrition Disorder*”[ti]) OR (Under-2[tiab] AND Malnutrition[tiab]) OR (Under-2[ti] AND Undernutrition[ti]) OR (Under-2[ti] AND Under-nutrition[ti]) OR (Under-2[ti] AND Undernourish*[ti]) OR (Under-2[ti] AND Overnutrition[ti]) OR (Under-2[ti] AND Underweight[ti]) OR (Under-2[ti] AND Leanness[ti]) OR (Under-2[ti] AND Thinness[ti]) OR (Under-2[ti] AND Slimness[ti]) OR (Under-2[ti] AND Overweight[ti]) OR (Under-2[ti] AND Obes*[ti]) OR (Under-2[tiab] AND Height*[tiab]) OR (Under-2[tiab] AND Weight*[tiab]) OR (Under-2[tiab] AND Length[tiab]) OR (Under-2[ti] AND “failure to thrive”[ti]) OR (Offspring[tiab] AND Growth[tiab]) OR (Offspring[ti] AND “Growth Retardation”[ti]) OR (Offspring[ti] AND “Growth Disorder*”[ti]) OR (Offspring[ti] AND “Growth Falter*”[ti]) OR (Offspring[ti] AND “Growth Deficit*”[ti]) OR (Offspring[ti] AND “Growth failure”[ti]) OR (Offspring[ti] AND “Growth Trajectory”[ti]) OR (Offspring[ti] AND “Nutrition Disorder*”[ti]) OR (Offspring[tiab] AND Malnutrition[tiab]) OR (Offspring[ti] AND Undernutrition[ti]) OR (Offspring[ti] AND Under-nutrition[ti]) OR (Offspring[ti] AND Undernourish*[ti]) OR (Offspring[ti] AND Overnutrition[ti]) OR (Offspring[ti] AND Underweight[ti]) OR (Offspring[ti] AND Leanness[ti]) OR (Offspring[ti] AND Thinness[ti]) OR (Offspring[ti] AND Slimness[ti]) OR (Offspring[ti] AND Overweight[ti]) OR (Offspring[ti] AND Obes*[ti]) OR (Offspring[tiab] AND Height*[tiab]) OR (Offspring[tiab] AND Weight*[tiab]) OR (Offspring[tiab] AND Length[tiab]) OR (Offspring[ti] AND “failure to thrive”[ti]) OR (Early-life[tiab] AND Growth[tiab]) OR (Early-life[ti] AND “Growth Retardation”[ti]) OR (Early-life[ti] AND “Growth Disorder*”[ti]) OR (Early-life[ti] AND “Growth Falter*”[ti]) OR (Early-life[ti] AND “Growth Deficit*”[ti]) OR (Early-life[ti] AND “Growth failure”[ti]) OR (Early-life[ti] AND “Growth Trajectory”[ti]) OR (Early-life[ti] AND “Nutrition Disorder*”[ti]) OR (Early-life[tiab] AND Malnutrition[tiab]) OR (Early-life[ti] AND Undernutrition[ti]) OR (Early-life[ti] AND Under-nutrition[ti]) OR (Early-life[ti] AND Undernourish*[ti]) OR (Early-life[ti] AND Overnutrition[ti]) OR (Early-life[ti] AND Underweight[ti]) OR (Early-life[ti] AND Leanness[ti]) OR (Early-life[ti] AND Thinness[ti]) OR (Early-life[ti] AND Slimness[ti]) OR (Early-life[ti] AND Overweight[ti]) OR (Early-life[ti] AND Obes*[ti]) OR (Early-life[tiab] AND Height*[tiab]) OR (Early-life[tiab] AND Weight*[tiab]) OR (Early-life[tiab] AND Length[tiab]) OR (Early-life[ti] AND “failure to thrive”[ti]) OR (“Early life”[tiab] AND Growth[tiab]) OR (“Early life”[ti] AND “Growth Retardation”[ti]) OR (“Early life”[ti] AND “Growth Disorder*”[ti]) OR (“Early life”[ti] AND “Growth Falter*”[ti]) OR (“Early life”[ti] AND “Growth Deficit*”[ti]) OR (“Early life”[ti] AND “Growth failure”[ti]) OR (“Early life”[ti] AND “Growth Trajectory”[ti]) OR (“Early life”[ti] AND “Nutrition Disorder*”[ti]) OR (“Early life”[tiab] AND Malnutrition[tiab]) OR (“Early life”[ti] AND Undernutrition[ti]) OR (“Early life”[ti] AND Under-nutrition[ti]) OR (“Early life”[ti] AND Undernourish*[ti]) OR (“Early life”[ti] AND Overnutrition[ti]) OR (“Early life”[ti] AND Underweight[ti]) OR (“Early life”[ti] AND Leanness[ti]) OR (“Early life”[ti] AND Thinness[ti]) OR (“Early life”[ti] AND Slimness[ti]) OR (“Early life”[ti] AND Overweight[ti]) OR (“Early life”[ti] AND Obes*[ti]) OR (“Early life”[tiab] AND Height*[tiab]) OR (“Early life”[tiab] AND Weight*[tiab]) OR (“Early life”[tiab] AND Length[tiab]) OR (“Early life”[ti] AND “failure to thrive”[ti]) OR (Pediatric[tiab] AND Growth[tiab]) OR (Pediatric[ti] AND “Growth Retardation”[ti]) OR (Pediatric[ti] AND “Growth Disorder*”[ti]) OR (Pediatric[ti] AND “Growth Falter*”[ti]) OR (Pediatric[ti] AND “Growth Deficit*”[ti]) OR (Pediatric[ti] AND “Growth failure”[ti]) OR (Pediatric[ti] AND “Growth Trajectory”[ti]) OR (Pediatric[ti] AND “Nutrition Disorder*”[ti]) OR (Pediatric[tiab] AND Malnutrition[tiab]) OR (Pediatric[ti] AND Undernutrition[ti]) OR (Pediatric[ti] AND Under-nutrition[ti]) OR (Pediatric[ti] AND Undernourish*[ti]) OR (Pediatric[ti] AND Overnutrition[ti]) OR (Pediatric[ti] AND Underweight[ti]) OR (Pediatric[ti] AND Leanness[ti]) OR (Pediatric[ti] AND Thinness[ti]) OR (Pediatric[ti] AND Slimness[ti]) OR (Pediatric[ti] AND Overweight[ti]) OR (Pediatric[ti] AND Obes*[ti]) OR (Pediatric[tiab] AND Height*[tiab]) OR (Pediatric[tiab] AND Weight*[tiab]) OR (Pediatric[tiab] AND Length[tiab]) OR (Pediatric[ti] AND “failure to thrive”[ti]) OR “child health”[ti])<br>AND<br>(cohort[tiab] OR longitudinal[tiab] OR (Cohort[tiab] AND Study[tiab]) OR (Concurrent[tiab] AND Study[tiab]) OR “birth cohort”[tiab] OR (cohort[tiab] AND analysis[tiab]) OR (cohort[tiab] AND analyses[tiab]) OR “incidence study”[tiab] OR “Follow Up Study”[tiab] OR “Follow-Up Study”[tiab] OR “Followup Study”[tiab] OR (Longitudinal[tiab] AND Study[tiab]) OR (Prospective[tiab] AND study[tiab]))<br>AND<br>(1990/1/01:2024/1/31[dp]))				
6	(((“Educational Status”[ti] AND Maternal[ti]) OR (Status[ti] AND “Maternal Educational”[ti]) OR “Maternal Educational Status”[ti] OR (Maternal[all] AND education[all]) OR (Maternal[ti] AND Illiteracy[ti]) OR (Maternal[ti] AND Literacy[ti]) OR (maternal[all] AND “schooling”[all]) OR (mother*[all] AND education[all]) OR (mother*[ti] AND Illiteracy[ti]) OR (mother*[ti] AND Literacy[ti]) OR (mother*[all] AND “schooling”[all]) OR (parent*[ti] AND Illiteracy[ti]) OR (parent*[ti] AND Literacy[ti]) OR (parent*[tiab] AND education[tiab]) OR (parent*[tiab] AND “schooling”[tiab]) OR (parent*[tiab] AND socio-economic[tiab]) OR (parent*[tiab] AND socioeconomic[tiab]) OR (parent*[ti] AND “Social Class*”[ti]) OR (parent*[tiab] AND Socio-demographic[tiab]) OR (parent*[tiab] AND Sociodemographic[tiab]) OR (parent*[ti] AND Socio-cultural[ti]) OR (parent*[ti] AND Sociocultural[ti]) OR (mother*[tiab] AND socio-economic[tiab])	33	5,878		

	OR (mother*[tiab] AND socioeconomic[tiab]) OR (mother*[ti] AND “Social Class*”[ti]) OR (mother*[tiab] AND Socio-demographic[tiab]) OR (mother*[tiab] AND Sociodemographic[tiab]) OR (mother*[ti] AND Socio-cultural[ti]) OR (mother*[ti] AND Sociocultural[ti]) OR (maternal[tiab] AND socio-economic[tiab]) OR (maternal[tiab] AND socioeconomic[tiab]) OR (maternal[ti] AND “Social Class*”[ti]) OR (maternal[tiab] AND Socio-demographic[tiab]) OR (maternal[tiab] AND Sociodemographic[tiab]) OR (maternal[ti] AND Socio-cultural[ti]) OR (maternal[ti] AND Sociocultural[ti]) OR “maternal socioeconomic status”[ti] OR “parental socioeconomic status”[ti])<br>AND<br>((Child*[tiab] AND Growth[tiab]) OR (Child*[ti] AND “Growth Retardation”[ti]) OR (Child*[ti] AND “Growth Disorder*”[ti]) OR (Child*[ti] AND “Growth Falter*”[ti]) OR (Child*[ti] AND “Growth Deficit*”[ti]) OR (Child*[ti] AND “Growth failure”[ti]) OR (Child*[ti] AND “Growth Trajectory”[ti]) OR Stunting*[tiab] OR “Stunted Growth”[ti] OR (Growth[ti] AND Stunted[ti]) OR “short stature”[ti] OR wast*[tiab] OR (Child*[ti] AND “Nutrition Disorder*”[ti]) OR (Child*[tiab] AND Malnutrition[tiab]) OR (Child*[ti] AND Undernutrition[ti]) OR (Child*[ti] AND Under-nutrition[ti]) OR (Child*[ti] AND Undernourish*[ti]) OR (Child*[ti] AND Overnutrition[ti]) OR (Child*[ti] AND Underweight[ti]) OR (Child*[ti] AND Leanness[ti]) OR (Child*[ti] AND Thinness[ti]) OR (Child*[ti] AND Slimness[ti]) OR (Child*[ti] AND Overweight[ti]) OR (Child*[ti] AND Obes*[ti]) OR (Child*[tiab] AND Height*[tiab]) OR (Child*[tiab] AND Weight*[tiab]) OR (Child*[tiab] AND Length[tiab]) OR Anthropometric*[ti] OR Length-for-age[all] OR Height-for-age[all] OR Weight-for-age[all] OR Weight-for-height[all] OR weight-for-length[all] OR “Body mass index-for-age”[all] OR BMI-for-age[all] OR (child*[ti] AND “failure to thrive”[ti]) OR (Infan*[tiab] AND Growth[tiab]) OR (Infan*[ti] AND “Growth Retardation”[ti]) OR (Infan*[ti] AND “Growth Disorder*”[ti]) OR (Infan*[ti] AND “Growth Falter*”[ti]) OR (Infan*[ti] AND “Growth Deficit*”[ti]) OR (Infan*[ti] AND “Growth failure”[ti]) OR (Infan*[ti] AND “Growth Trajectory”[ti]) OR (Infan*[ti] AND “Nutrition Disorder*”[ti]) OR (Infan*[tiab] AND Malnutrition[tiab]) OR (Infan*[ti] AND Undernutrition[ti]) OR (Infan*[ti] AND Under-nutrition[ti]) OR (Infan*[ti] AND Undernourish*[ti]) OR (Infan*[ti] AND Overnutrition[ti]) OR (Infan*[ti] AND Underweight[ti]) OR (Infan*[ti] AND Leanness[ti]) OR (Infan*[ti] AND Thinness[ti]) OR (Infan*[ti] AND Slimness[ti]) OR (Infan*[ti] AND Overweight[ti]) OR (Infan*[ti] AND Obes*[ti]) OR (Infan*[tiab] AND Height*[tiab]) OR (Infan*[tiab] AND Weight*[tiab]) OR (Infan*[tiab] AND Length[tiab]) OR (infan*[ti] AND “failure to thrive”[ti]) OR (Under-five[tiab] AND Growth[tiab]) OR (Under-five[ti] AND “Growth Retardation”[ti]) OR (Under-five[ti] AND “Growth Disorder*”[ti]) OR (Under-five[ti] AND “Growth Falter*”[ti]) OR (Under-five[ti] AND “Growth Deficit*”[ti]) OR (Under-five[ti] AND “Growth failure”[ti]) OR (Under-five[ti] AND “Growth Trajectory”[ti]) OR (Under-five[ti] AND “Nutrition Disorder*”[ti]) OR (Under-five[tiab] AND Malnutrition[tiab]) OR (Under-five[ti] AND Undernutrition[ti]) OR (Under-five[ti] AND Under-nutrition[ti]) OR (Under-five[ti] AND Undernourish*[ti]) OR (Under-five[ti] AND Overnutrition[ti]) OR (Under-five[ti] AND Underweight[ti]) OR (Under-five[tiab] AND Leanness[ti]) OR (Under-five[ti] AND Thinness[ti]) OR (Under-five[ti] AND Slimness[ti]) OR (Under-five[ti] AND Overweight[ti]) OR (Under-five[ti] AND Obes*[ti]) OR (Under-five[tiab] AND Height*[tiab]) OR (Under-five[tiab] AND Weight*[tiab]) OR (Under-five[tiab] AND Length[tiab]) OR (under-five[ti] AND “failure to thrive”[ti]) OR (“under five”[tiab] AND Growth[tiab]) OR (“under five”[ti] AND “Growth Retardation”[ti]) OR (“under five”[ti] AND “Growth Disorder*”[ti]) OR (“under five”[ti] AND “Growth Falter*”[ti]) OR (“under five”[ti] AND “Growth Deficit*”[ti]) OR (“under five”[ti] AND “Growth failure”[ti]) OR (“under five”[ti] AND “Growth Trajectory”[ti]) OR (“under five”[ti] AND “Nutrition Disorder*”[ti]) OR (“under five”[tiab] AND Malnutrition[tiab]) OR (“under five”[ti] AND Undernutrition[ti]) OR (“under five”[ti] AND Under-nutrition[ti]) OR (“under five”[ti] AND Undernourish*[ti]) OR (“under five”[ti] AND Overnutrition[ti]) OR (“under five”[ti] AND Underweight[ti]) OR (“under five”[ti] AND Leanness[ti]) OR (“under five”[ti] AND Thinness[ti]) OR (“under five”[ti] AND Slimness[ti]) OR (“under five”[ti] AND Overweight[ti]) OR (“under five”[ti] AND Obes*[ti]) OR (“under five”[tiab] AND Height*[tiab]) OR (“under five”[tiab] AND Weight*[tiab]) OR (“under five”[tiab] AND Length[tiab]) OR (“under five”[ti] AND “failure to thrive”[ti]) OR (“under 5”[tiab] AND Growth[tiab]) OR (“under 5”[ti] AND “Growth Retardation”[ti]) OR (“under 5”[ti] AND “Growth Disorder*”[ti]) OR (“under 5”[ti] AND “Growth Falter*”[ti]) OR (“under 5”[ti] AND “Growth Deficit*”[ti]) OR (“under 5”[ti] AND “Growth failure”[ti])OR (“under 5”[ti] AND “Growth Trajectory”[ti]) OR (“under 5”[ti] AND “Nutrition Disorder*”[ti]) OR (“under 5”[tiab] AND Malnutrition[tiab]) OR (“under 5”[ti] AND Undernutrition[ti]) OR (“under 5”[ti] AND Under-nutrition[ti]) OR (“under 5”[ti] AND Undernourish*[ti]) OR (“under 5”[ti] AND Overnutrition[ti]) OR (“under 5”[ti] AND Underweight[ti]) OR (“under 5”[ti] AND Leanness[ti]) OR (“under 5”[ti] AND Thinness[ti]) OR (“under 5”[ti] AND Slimness[ti]) OR (“under 5”[ti] AND Overweight[ti]) OR (“under 5”[ti] AND Obes*[ti]) OR (“under 5”[tiab] AND Height*[tiab]) OR (“under 5”[tiab] AND Weight*[tiab]) OR (“under 5”[tiab] AND Length[tiab]) OR (“under 5”[ti] AND “failure to thrive”[ti]) OR (Under-5[tiab] AND Growth[tiab]) OR (Under-5[ti] AND “Growth Retardation”[ti]) OR (Under-5[ti] AND “Growth Disorder*”[ti]) OR (Under-5[ti] AND “Growth Falter*”[ti]) OR (Under-5[ti] AND “Growth Deficit*”[ti]) OR (Under-5[ti] AND “Growth failure”[ti]) OR (Under-5[ti] AND “Growth Trajectory”[ti]) OR (Under-5[ti] AND “Nutrition Disorder*”[ti]) OR (Under-5[tiab] AND Malnutrition[tiab]) OR (Under-5[ti] AND Undernutrition[ti]) OR (Under-5[ti] AND Under-nutrition[ti]) OR (Under-5[ti] AND Undernourish*[ti]) OR (Under-5[ti] AND Overnutrition[ti]) OR (Under-5[ti] AND Underweight[ti]) OR (Under-5[ti] AND Leanness[ti]) OR (Under-5[ti] AND Thinness[ti]) OR (Under-5[ti] AND Slimness[ti]) OR (Under-5[ti] AND Overweight[ti]) OR (Under-5[ti] AND Obes*[ti]) OR (Under-5[tiab] AND Height*[tiab]) OR (Under-5[tiab] AND Weight*[tiab]) OR (Under-5[tiab] AND Length[tiab]) OR (Under-5[ti] AND				

	"failure to thrive"[ti]) OR (Under-two[tiab] AND Growth[tiab]) OR (Under-two[ti] AND "Growth Retardation"[ti]) OR (Under-two[ti] AND "Growth Disorder*"[ti]) OR (Under-two[ti] AND "Growth Falter*"[ti]) OR (Under-two[ti] AND "Growth Deficit*"[ti]) OR (Under-two[ti] AND "Growth failure"[ti]) OR (Under-two[ti] AND "Growth Trajectory"[ti]) OR (Under-two[ti] AND "Nutrition Disorder*"[ti]) OR (Under-two[tiab] AND Malnutrition[tiab]) OR (Under-two[ti] AND Undernutrition[ti]) OR (Under-two[ti] AND Under-nutrition[ti]) OR (Under-two[ti] AND Undernourish*[ti]) OR (Under-two[ti] AND Overnutrition[ti]) OR (Under-two[ti] AND Underweight[ti]) OR (Under-two[ti] AND Leanness[ti]) OR (Under-two[ti] AND Thinness[ti]) OR (Under-two[ti] AND Slimness[ti]) OR (Under-two[tiab] AND Overweight[tiab]) OR (Under-two[ti] AND Obes*[ti]) OR (Under-two[tiab] AND Height*[tiab]) OR (Under-two[tiab] AND Weight*[tiab]) OR (Under-two[tiab] AND Length[tiab]) OR (Under-two[ti] AND "failure to thrive"[ti]) OR ("under two"[tiab] AND Growth[tiab]) OR ("under two"[ti] AND "Growth Retardation"[ti]) OR ("under two"[ti] AND "Growth Disorder*"[ti]) OR ("under two"[ti] AND "Growth Falter*"[ti]) OR ("under two"[ti] AND "Growth Deficit*"[ti]) OR ("under two"[ti] AND "Growth failure"[ti]) OR ("under two"[ti] AND "Growth Trajectory"[ti]) OR ("under two"[ti] AND "Nutrition Disorder*"[ti]) OR ("under two"[tiab] AND Malnutrition[tiab]) OR ("under two"[ti] AND Undernutrition[ti]) OR ("under two"[ti] AND Under-nutrition[ti]) OR ("under two"[ti] AND Undernourish*[ti]) OR ("under two"[ti] AND Overnutrition[ti]) OR ("under two"[ti] AND Underweight[ti]) OR ("under two"[ti] AND Leanness[ti]) OR ("under two"[ti] AND Thinness[ti]) OR ("under two"[ti] AND Slimness[ti]) OR ("under two"[ti] AND Overweight[ti]) OR ("under two"[ti] AND Obes*[ti]) OR ("under two"[tiab] AND Height*[tiab]) OR ("under two"[tiab] AND Weight*[tiab]) OR ("under two"[tiab] AND Length[tiab]) OR ("under two"[ti] AND "failure to thrive"[ti]) OR ("under 2"[tiab] AND Growth[tiab]) OR ("under 2"[ti] AND "Growth Retardation"[ti]) OR ("under 2"[ti] AND "Growth Disorder*"[ti]) OR ("under 2"[ti] AND "Growth Falter*"[ti]) OR ("under 2"[ti] AND "Growth Deficit*"[ti]) OR ("under 2"[ti] AND "Growth failure"[ti]) OR ("under 2"[ti] AND "Growth Trajectory"[ti]) OR ("under 2"[ti] AND "Nutrition Disorder*"[ti]) OR ("under 2"[tiab] AND Malnutrition[tiab]) OR ("under 2"[ti] AND Undernutrition[ti]) OR ("under 2"[ti] AND Under-nutrition[ti]) OR ("under 2"[ti] AND Undernourish*[ti]) OR ("under 2"[ti] AND Overnutrition[ti]) OR ("under 2"[ti] AND Underweight[ti]) OR ("under 2"[ti] AND Leanness[ti]) OR ("under 2"[ti] AND Thinness[ti]) OR ("under 2"[ti] AND Slimness[ti]) OR ("under 2"[ti] AND Overweight[ti]) OR ("under 2"[ti] AND Obes*[ti]) OR ("under 2"[tiab] AND Height*[tiab]) OR ("under 2"[tiab] AND Weight*[tiab]) OR ("under 2"[tiab] AND Length[tiab]) OR ("under 2"[ti] AND "failure to thrive"[ti]) OR (Under-2[tiab] AND Growth[tiab]) OR (Under-2[ti] AND "Growth Retardation"[ti]) OR (Under-2[ti] AND "Growth Disorder*"[ti]) OR (Under-2[ti] AND "Growth Falter*"[ti]) OR (Under-2[ti] AND "Growth Deficit*"[ti]) OR (Under-2[ti] AND "Growth failure"[ti]) OR (Under-2[ti] AND "Growth Trajectory"[ti]) OR (Under-2[ti] AND "Nutrition Disorder*"[ti]) OR (Under-2[tiab] AND Malnutrition[tiab]) OR (Under-2[ti] AND Undernutrition[ti]) OR (Under-2[ti] AND Under-nutrition[ti]) OR (Under-2[ti] AND Undernourish*[ti]) OR (Under-2[ti] AND Overnutrition[ti]) OR (Under-2[ti] AND Underweight[ti]) OR (Under-2[ti] AND Leanness[ti]) OR (Under-2[ti] AND Thinness[ti]) OR (Under-2[ti] AND Slimness[ti]) OR (Under-2[ti] AND Overweight[ti]) OR (Under-2[ti] AND Obes*[ti]) OR (Under-2[tiab] AND Height*[tiab]) OR (Under-2[tiab] AND Weight*[tiab]) OR (Under-2[tiab] AND Length[tiab]) OR (Under-2[ti] AND "failure to thrive"[ti]) OR (Offspring[tiab] AND Growth[tiab]) OR (Offspring[ti] AND "Growth Retardation"[ti]) OR (Offspring[ti] AND "Growth Disorder*"[ti]) OR (Offspring[ti] AND "Growth Falter*"[ti]) OR (Offspring[ti] AND "Growth Deficit*"[ti]) OR (Offspring[ti] AND "Growth failure"[ti]) OR (Offspring[ti] AND "Growth Trajectory"[ti]) OR (Offspring[ti] AND "Nutrition Disorder*"[ti]) OR (Offspring[tiab] AND Malnutrition[tiab]) OR (Offspring[ti] AND Undernutrition[ti]) OR (Offspring[ti] AND Under-nutrition[ti]) OR (Offspring[ti] AND Undernourish*[ti]) OR (Offspring[ti] AND Overnutrition[ti]) OR (Offspring[ti] AND Underweight[ti]) OR (Offspring[ti] AND Leanness[ti]) OR (Offspring[ti] AND Thinness[ti]) OR (Offspring[ti] AND Slimness[ti]) OR (Offspring[ti] AND Overweight[ti]) OR (Offspring[ti] AND Obes*[ti]) OR (Offspring[tiab] AND Height*[tiab]) OR (Offspring[tiab] AND Weight*[tiab]) OR (Offspring[tiab] AND Length[tiab]) OR (Offspring[ti] AND "failure to thrive"[ti]) OR (Early-life[tiab] AND Growth[tiab]) OR (Early-life[ti] AND "Growth Retardation"[ti]) OR (Early-life[ti] AND "Growth Disorder*"[ti]) OR (Early-life[ti] AND "Growth Falter*"[ti]) OR (Early-life[ti] AND "Growth Deficit*"[ti]) OR (Early-life[ti] AND "Growth failure"[ti]) OR (Early-life[ti] AND "Growth Trajectory"[ti]) OR (Early-life[ti] AND "Nutrition Disorder*"[ti]) OR (Early-life[tiab] AND Malnutrition[tiab]) OR (Early-life[ti] AND Undernutrition[ti]) OR (Early-life[ti] AND Under-nutrition[ti]) OR (Early-life[ti] AND Undernourish*[ti]) OR (Early-life[ti] AND Overnutrition[ti]) OR (Early-life[ti] AND Underweight[ti]) OR (Early-life[ti] AND Leanness[ti]) OR (Early-life[ti] AND Thinness[ti]) OR (Early-life[ti] AND Slimness[ti]) OR (Early-life[ti] AND Overweight[ti]) OR (Early-life[ti] AND Obes*[ti]) OR (Early-life[tiab] AND Height*[tiab]) OR (Early-life[tiab] AND Weight*[tiab]) OR (Early-life[tiab] AND Length[tiab]) OR (Early-life[ti] AND "failure to thrive"[ti]) OR ("Early life"[tiab] AND Growth[tiab]) OR ("Early life"[ti] AND "Growth Retardation"[ti]) OR ("Early life"[ti] AND "Growth Disorder*"[ti]) OR ("Early life"[ti] AND "Growth Falter*"[ti]) OR ("Early life"[ti] AND "Growth Deficit*"[ti]) OR ("Early life"[ti] AND "Growth failure"[ti]) OR ("Early life"[ti] AND "Growth Trajectory"[ti]) OR ("Early life"[ti] AND "Nutrition Disorder*"[ti]) OR ("Early life"[tiab] AND Malnutrition[tiab]) OR ("Early life"[ti] AND Undernutrition[ti]) OR ("Early life"[ti] AND Under-nutrition[ti]) OR ("Early life"[ti] AND Undernourish*[ti]) OR ("Early life"[ti] AND Overnutrition[ti]) OR ("Early life"[ti] AND Underweight[ti]) OR ("Early life"[ti] AND Leanness[ti]) OR ("Early life"[ti] AND Thinness[ti]) OR ("Early life"[ti] AND Slimness[ti]) OR ("Early life"[ti] AND Overweight[ti]) OR ("Early life"[ti] AND Obes*[ti]) OR ("Early life"[tiab] AND				

	Height*[tiab]) OR (“Early life”[tiab] AND Weight*[tiab]) OR (“Early life”[tiab] AND Length[tiab]) OR (“Early life”[ti] AND “failure to thrive”[ti]) OR (Pediatric[tiab] AND Growth[tiab]) OR (Pediatric[ti] AND “Growth Retardation”[ti]) OR (Pediatric[ti] AND “Growth Disorder*”[ti]) OR (Pediatric[ti] AND “Growth Falter*”[ti]) OR (Pediatric[ti] AND “Growth Deficit*”[ti]) OR (Pediatric[ti] AND “Growth failure”[ti]) OR (Pediatric[ti] AND “Growth Trajectory”[ti]) OR (Pediatric[ti] AND “Nutrition Disorder*”[ti]) OR (Pediatric[tiab] AND Malnutrition[tiab]) OR (Pediatric[ti] AND Undernutrition[ti]) OR (Pediatric[ti] AND Under-nutrition[ti]) OR (Pediatric[ti] AND Undernourish*[ti]) OR (Pediatric[ti] AND Overnutrition[ti]) OR (Pediatric[ti] AND Underweight[ti]) OR (Pediatric[ti] AND Leanness[ti]) OR (Pediatric[ti] AND Thinness[ti]) OR (Pediatric[ti] AND Slimness[ti]) OR (Pediatric[ti] AND Overweight[ti]) OR (Pediatric[ti] AND Obes*[ti]) OR (Pediatric[tiab] AND Height*[tiab]) OR (Pediatric[tiab] AND Weight*[tiab]) OR (Pediatric[tiab] AND Length[tiab]) OR (Pediatric[ti] AND “failure to thrive”[ti]) OR “child health”[ti])<br>AND<br>(cohort[tiab] OR longitudinal[tiab] OR (Cohort[tiab] AND Study[tiab]) OR (Concurrent[tiab] AND Study[tiab]) OR “birth cohort”[tiab] OR (cohort[tiab] AND analysis[tiab]) OR (cohort[tiab] AND analyses[tiab]) OR “incidence study”[tiab] OR “Follow Up Study”[tiab] OR “Follow-Up Study”[tiab] OR “Followup Study”[tiab] OR (Longitudinal[tiab] AND Study[tiab]) OR (Prospective[tiab] AND study[tiab]))<br>AND<br>(1990/1/01:2024/1/31[dp]))				
7	(((“Educational Status”[ti] AND Maternal[ti]) OR (Status[ti] AND “Maternal Educational”[ti]) OR “Maternal Educational Status”[ti] OR (Maternal[all] AND education[all]) OR (Maternal[ti] AND Illiteracy[ti]) OR (Maternal[ti] AND Literacy[ti]) OR (maternal[all] AND “schooling”[all]) OR (mother*[all] AND education[all]) OR (mother*[ti] AND Illiteracy[ti]) OR (mother*[ti] AND Literacy[ti]) OR (mother*[all] AND “schooling”[all]) OR (parent*[ti] AND Illiteracy[ti]) OR (parent*[ti] AND Literacy[ti]) OR (parent*[ti] AND education[ti]) OR (parent*[ti] AND “schooling”[ti]) OR (parent*[ti] AND socio-economic[ti]) OR (parent*[ti] AND socioeconomic[ti]) OR (parent*[ti] AND “Social Class*”[ti]) OR (parent*[ti] AND Socio-demographic[ti]) OR (parent*[ti] AND Sociodemographic[ti]) OR (parent*[ti] AND Socio-cultural[ti]) OR (parent*[ti] AND Sociocultural[ti]) OR (mother*[tiab] AND socio-economic[tiab]) OR (mother*[tiab] AND socioeconomic[tiab]) OR (mother*[ti] AND “Social Class*”[ti]) OR (mother*[tiab] AND Socio-demographic[tiab]) OR (mother*[tiab] AND Sociodemographic[tiab]) OR (mother*[ti] AND Socio-cultural[ti]) OR (mother*[ti] AND Sociocultural[ti]) OR (maternal[tiab] AND socio-economic[tiab]) OR (maternal[tiab] AND socioeconomic[tiab]) OR (maternal[ti] AND “Social Class*”[ti]) OR (maternal[tiab] AND Socio-demographic[tiab]) OR (maternal[tiab] AND Sociodemographic[tiab]) OR (maternal[ti] AND Socio-cultural[ti]) OR (maternal[ti] AND Sociocultural[ti]) OR “maternal socioeconomic status”[ti] OR “parental socioeconomic status”[ti])<br>AND<br>((Child*[tiab] AND Growth[tiab]) OR (Child*[ti] AND “Growth Retardation”[ti]) OR (Child*[ti] AND “Growth Disorder*”[ti]) OR (Child*[ti] AND “Growth Falter*”[ti]) OR (Child*[ti] AND “Growth Deficit*”[ti]) OR (Child*[ti] AND “Growth failure”[ti]) OR (Child*[ti] AND “Growth Trajectory”[ti]) OR Stunting*[tiab] OR “Stunted Growth”[ti] OR (Growth[ti] AND Stunted[ti]) OR “short stature”[ti] OR wast*[tiab] OR (Child*[ti] AND “Nutrition Disorder*”[ti]) OR (Child*[tiab] AND Malnutrition[tiab]) OR (Child*[ti] AND Undernutrition[ti]) OR (Child*[ti] AND Under-nutrition[ti]) OR (Child*[ti] AND Undernourish*[ti]) OR (Child*[ti] AND Overnutrition[ti]) OR (Child*[ti] AND Underweight[ti]) OR (Child*[ti] AND Leanness[ti]) OR (Child*[ti] AND Thinness[ti]) OR (Child*[ti] AND Slimness[ti]) OR (Child*[ti] AND Overweight[ti]) OR (Child*[ti] AND Obes*[ti]) OR (Child*[tiab] AND Height*[tiab]) OR (Child*[tiab] AND Weight*[tiab]) OR (Child*[tiab] AND Length[tiab]) OR Anthropometric*[ti] OR Length-for-age[tiab] OR Height-for-age[tiab] OR Weight-for-age[tiab] OR Weight-for-height[tiab] OR weight-for-length[tiab] OR “Body mass index-for-age”[tiab] OR BMI-for-age[tiab] OR (child*[ti] AND “failure to thrive”[ti]) OR (Infan*[tiab] AND Growth[tiab]) OR (Infan*[ti] AND “Growth Retardation”[ti]) OR (Infan*[ti] AND “Growth Disorder*”[ti]) OR (Infan*[ti] AND “Growth Falter*”[ti]) OR (Infan*[ti] AND “Growth Deficit*”[ti]) OR (Infan*[ti] AND “Growth failure”[ti]) OR (Infan*[ti] AND “Growth Trajectory”[ti]) OR (Infan*[ti] AND “Nutrition Disorder*”[ti]) OR (Infan*[tiab] AND Malnutrition[tiab]) OR (Infan*[ti] AND Undernutrition[ti]) OR (Infan*[ti] AND Under-nutrition[ti]) OR (Infan*[ti] AND Undernourish*[ti]) OR (Infan*[ti] AND Overnutrition[ti]) OR (Infan*[ti] AND Underweight[ti]) OR (Infan*[ti] AND Leanness[ti]) OR (Infan*[ti] AND Thinness[ti]) OR (Infan*[ti] AND Slimness[ti]) OR (Infan*[ti] AND Overweight[ti]) OR (Infan*[ti] AND Obes*[ti]) OR (Infan*[tiab] AND Height*[tiab]) OR (Infan*[tiab] AND Weight*[tiab]) OR (Infan*[tiab] AND Length[tiab]) OR (infan*[ti] AND “failure to thrive”[ti]) OR (Under-five[tiab] AND Growth[tiab]) OR (Under-five[ti] AND “Growth Retardation”[ti]) OR (Under-five[ti] AND “Growth Disorder*”[ti]) OR (Under-five[ti] AND “Growth Falter*”[ti]) OR (Under-five[ti] AND “Growth Deficit*”[ti]) OR (Under-five[ti] AND “Growth failure”[ti]) OR (Under-five[ti] AND “Growth Trajectory”[ti]) OR (Under-five[ti] AND “Nutrition Disorder*”[ti]) OR (Under-five[tiab] AND Malnutrition[tiab]) OR (Under-five[ti] AND Undernutrition[ti]) OR (Under-five[ti] AND Under-nutrition[ti]) OR (Under-five[ti] AND Undernourish*[ti]) OR (Under-five[ti] AND Overnutrition[ti]) OR (Under-five[ti] AND Underweight[ti]) OR (Under-five[tiab] AND Leanness[ti]) OR (Under-five[ti] AND Thinness[ti]) OR (Under-five[ti] AND Slimness[ti]) OR (Under-five[ti] AND Overweight[ti]) OR (Under-five[ti] AND Obes*[ti]) OR (Under-five[tiab] AND Height*[tiab]) OR (Under-five[tiab] AND Weight*[tiab]) OR (Under-five[tiab] AND Length[tiab]) OR (under-five[ti] AND “failure to thrive”[ti]) OR (“under five”[tiab] AND Growth[tiab]) OR (“under five”[ti] AND	33	4,926		

	“Growth Retardation”[ti]) OR (“under five”[ti] AND “Growth Disorder*”[ti]) OR (“under five”[ti] AND “Growth Falter*”[ti]) OR (“under five”[ti] AND “Growth Deficit*”[ti]) OR (“under five”[ti] AND “Growth failure”[ti]) OR (“under five”[ti] AND “Growth Trajectory”[ti]) OR (“under five”[ti] AND “Nutrition Disorder*”[ti]) OR (“under five”[tiab] AND Malnutrition[tiab]) OR (“under five”[ti] AND Undernutrition[ti]) OR (“under five”[ti] AND Under-nutrition[ti]) OR (“under five”[ti] AND Undernourish*[ti]) OR (“under five”[ti] AND Overnutrition[ti]) OR (“under five”[ti] AND Underweight[ti]) OR (“under five”[ti] AND Leanness[ti]) OR (“under five”[ti] AND Thinness[ti]) OR (“under five”[ti] AND Slimness[ti]) OR (“under five”[ti] AND Overweight[ti]) OR (“under five”[ti] AND Obes*[ti]) OR (“under five”[tiab] AND Height*[tiab]) OR (“under five”[tiab] AND Weight*[tiab]) OR (“under five”[tiab] AND Length[tiab]) OR (“under five”[ti] AND “failure to thrive”[ti]) OR (“under 5”[tiab] AND Growth[tiab]) OR (“under 5”[ti] AND “Growth Retardation”[ti]) OR (“under 5”[ti] AND “Growth Disorder*”[ti]) OR (“under 5”[ti] AND “Growth Falter*”[ti]) OR (“under 5”[ti] AND “Growth Deficit*”[ti]) OR (“under 5”[ti] AND “Growth failure”[ti])OR (“under 5”[ti] AND “Growth Trajectory”[ti]) OR (“under 5”[ti] AND “Nutrition Disorder*”[ti]) OR (“under 5”[tiab] AND Malnutrition[tiab]) OR (“under 5”[ti] AND Undernutrition[ti]) OR (“under 5”[ti] AND Under-nutrition[ti]) OR (“under 5”[ti] AND Undernourish*[ti]) OR (“under 5”[ti] AND Overnutrition[ti]) OR (“under 5”[ti] AND Underweight[ti]) OR (“under 5”[ti] AND Leanness[ti]) OR (“under 5”[ti] AND Thinness[ti]) OR (“under 5”[ti] AND Slimness[ti]) OR (“under 5”[ti] AND Overweight[ti]) OR (“under 5”[ti] AND Obes*[ti]) OR (“under 5”[tiab] AND Height*[tiab]) OR (“under 5”[tiab] AND Weight*[tiab]) OR (“under 5”[tiab] AND Length[tiab]) OR (“under 5”[ti] AND “failure to thrive”[ti]) OR (Under-5[tiab] AND Growth[tiab]) OR (Under-5[ti] AND “Growth Retardation”[ti]) OR (Under-5[ti] AND “Growth Disorder*”[ti]) OR (Under-5[ti] AND “Growth Falter*”[ti]) OR (Under-5[ti] AND “Growth Deficit*”[ti]) OR (Under-5[ti] AND “Growth failure”[ti]) OR (Under-5[ti] AND “Growth Trajectory”[ti]) OR (Under-5[ti] AND “Nutrition Disorder*”[ti]) OR (Under-5[tiab] AND Malnutrition[tiab]) OR (Under-5[ti] AND Undernutrition[ti]) OR (Under-5[ti] AND Under-nutrition[ti]) OR (Under-5[ti] AND Undernourish*[ti]) OR (Under-5[ti] AND Overnutrition[ti]) OR (Under-5[ti] AND Underweight[ti]) OR (Under-5[ti] AND Leanness[ti]) OR (Under-5[ti] AND Thinness[ti]) OR (Under-5[ti] AND Slimness[ti]) OR (Under-5[ti] AND Overweight[ti]) OR (Under-5[ti] AND Obes*[ti]) OR (Under-5[tiab] AND Height*[tiab]) OR (Under-5[tiab] AND Weight*[tiab]) OR (Under-5[tiab] AND Length[tiab]) OR (Under-5[ti] AND “failure to thrive”[ti]) OR (Under-two[tiab] AND Growth[tiab]) OR (Under-two[ti] AND “Growth Retardation”[ti]) OR (Under-two[ti] AND “Growth Disorder*”[ti]) OR (Under-two[ti] AND “Growth Falter*”[ti]) OR (Under-two[ti] AND “Growth Deficit*”[ti]) OR (Under-two[ti] AND “Growth failure”[ti]) OR (Under-two[ti] AND “Growth Trajectory”[ti]) OR (Under-two[ti] AND “Nutrition Disorder*”[ti]) OR (Under-two[tiab] AND Malnutrition[tiab]) OR (Under-two[ti] AND Undernutrition[ti]) OR (Under-two[ti] AND Under-nutrition[ti]) OR (Under-two[ti] AND Undernourish*[ti]) OR (Under-two[ti] AND Overnutrition[ti]) OR (Under-two[ti] AND Underweight[ti]) OR (Under-two[ti] AND Leanness[ti]) OR (Under-two[ti] AND Thinness[ti]) OR (Under-two[ti] AND Slimness[ti]) OR (Under-two[tiab] AND Overweight[tiab]) OR (Under-two[ti] AND Obes*[ti]) OR (Under-two[tiab] AND Height*[tiab]) OR (Under-two[tiab] AND Weight*[tiab]) OR (Under-two[tiab] AND Length[tiab]) OR (Under-two[ti] AND “failure to thrive”[ti]) OR (“under two”[tiab] AND Growth[tiab]) OR (“under two”[ti] AND “Growth Retardation”[ti]) OR (“under two”[ti] AND “Growth Disorder*”[ti]) OR (“under two”[ti] AND “Growth Falter*”[ti]) OR (“under two”[ti] AND “Growth Deficit*”[ti]) OR (“under two”[ti] AND “Growth failure”[ti]) OR (“under two”[ti] AND “Growth Trajectory”[ti]) OR (“under two”[ti] AND “Nutrition Disorder*”[ti]) OR (“under two”[tiab] AND Malnutrition[tiab]) OR (“under two”[ti] AND Undernutrition[ti]) OR (“under two”[ti] AND Under-nutrition[ti]) OR (“under two”[ti] AND Undernourish*[ti]) OR (“under two”[ti] AND Overnutrition[ti]) OR (“under two”[ti] AND Underweight[ti]) OR (“under two”[ti] AND Leanness[ti]) OR (“under two”[ti] AND Thinness[ti]) OR (“under two”[ti] AND Slimness[ti]) OR (“under two”[ti] AND Overweight[ti]) OR (“under two”[ti] AND Obes*[ti]) OR (“under two”[tiab] AND Height*[tiab]) OR (“under two”[tiab] AND Weight*[tiab]) OR (“under two”[tiab] AND Length[tiab]) OR (“under two”[ti] AND “failure to thrive”[ti]) OR (“under 2”[tiab] AND Growth[tiab]) OR (“under 2”[ti] AND “Growth Retardation”[ti]) OR (“under 2”[ti] AND “Growth Disorder*”[ti]) OR (“under 2”[ti] AND “Growth Falter*”[ti]) OR (“under 2”[ti] AND “Growth Deficit*”[ti]) OR (“under 2”[ti] AND “Growth failure”[ti]) OR (“under 2”[ti] AND “Growth Trajectory”[ti]) OR (“under 2”[ti] AND “Nutrition Disorder*”[ti]) OR (“under 2”[tiab] AND Malnutrition[tiab]) OR (“under 2”[ti] AND Undernutrition[ti]) OR (“under 2”[ti] AND Under-nutrition[ti]) OR (“under 2”[ti] AND Undernourish*[ti]) OR (“under 2”[ti] AND Overnutrition[ti]) OR (“under 2”[ti] AND Underweight[ti]) OR (“under 2”[ti] AND Leanness[ti]) OR (“under 2”[ti] AND Thinness[ti]) OR (“under 2”[ti] AND Slimness[ti]) OR (“under 2”[ti] AND Overweight[ti]) OR (“under 2”[ti] AND Obes*[ti]) OR (“under 2”[tiab] AND Height*[tiab]) OR (“under 2”[tiab] AND Weight*[tiab]) OR (“under 2”[tiab] AND Length[tiab]) OR (“under 2”[ti] AND “failure to thrive”[ti]) OR (Under-2[tiab] AND Growth[tiab]) OR (Under-2[ti] AND “Growth Retardation”[ti]) OR (Under-2[ti] AND “Growth Disorder*”[ti]) OR (Under-2[ti] AND “Growth Falter*”[ti]) OR (Under-2[ti] AND “Growth Deficit*”[ti]) OR (Under-2[ti] AND “Growth failure”[ti]) OR (Under-2[ti] AND “Growth Trajectory”[ti]) OR (Under-2[ti] AND “Nutrition Disorder*”[ti]) OR (Under-2[tiab] AND Malnutrition[tiab]) OR (Under-2[ti] AND Undernutrition[ti]) OR (Under-2[ti] AND Under-nutrition[ti]) OR (Under-2[ti] AND Undernourish*[ti]) OR (Under-2[ti] AND Overnutrition[ti]) OR (Under-2[ti] AND Underweight[ti]) OR (Under-2[ti] AND Leanness[ti]) OR (Under-2[ti] AND Thinness[ti]) OR (Under-2[ti] AND Slimness[ti]) OR (Under-2[ti] AND Overweight[ti]) OR (Under-2[ti] AND Obes*[ti]) OR (Under-2[tiab] AND Height*[tiab]) OR (Under-2[tiab] AND Weight*[tiab]) OR (Under-2[tiab] AND Length[tiab]) OR (Under-2[ti] AND				

	“failure to thrive”[ti]) OR (Offspring[tiab] AND Growth[tiab]) OR (Offspring[ti] AND “Growth Retardation”[ti]) OR (Offspring[ti] AND “Growth Disorder*”[ti]) OR (Offspring[ti] AND “Growth Falter*”[ti]) OR (Offspring[ti] AND “Growth Deficit*”[ti]) OR (Offspring[ti] AND “Growth failure”[ti]) OR (Offspring[ti] AND “Growth Trajectory”[ti]) OR (Offspring[ti] AND “Nutrition Disorder*”[ti]) OR (Offspring[tiab] AND Malnutrition[tiab]) OR (Offspring[ti] AND Undernutrition[ti]) OR (Offspring[ti] AND Under-nutrition[ti]) OR (Offspring[ti] AND Undernourish*[ti]) OR (Offspring[ti] AND Overnutrition[ti]) OR (Offspring[ti] AND Underweight[ti]) OR (Offspring[ti] AND Leanness[ti]) OR (Offspring[ti] AND Thinness[ti]) OR (Offspring[ti] AND Slimness[ti]) OR (Offspring[ti] AND Overweight[ti]) OR (Offspring[ti] AND Obes*[ti]) OR (Offspring[tiab] AND Height*[tiab]) OR (Offspring[tiab] AND Weight*[tiab]) OR (Offspring[tiab] AND Length[tiab]) OR (Offspring[ti] AND “failure to thrive”[ti]) OR (Early-life[tiab] AND Growth[tiab]) OR (Early-life[ti] AND “Growth Retardation”[ti]) OR (Early-life[ti] AND “Growth Disorder*”[ti]) OR (Early-life[ti] AND “Growth Falter*”[ti]) OR (Early-life[ti] AND “Growth Deficit*”[ti]) OR (Early-life[ti] AND “Growth failure”[ti]) OR (Early-life[ti] AND “Growth Trajectory”[ti]) OR (Early-life[ti] AND “Nutrition Disorder*”[ti]) OR (Early-life[tiab] AND Malnutrition[tiab]) OR (Early-life[ti] AND Undernutrition[ti]) OR (Early-life[ti] AND Under-nutrition[ti]) OR (Early-life[ti] AND Undernourish*[ti]) OR (Early-life[ti] AND Overnutrition[ti]) OR (Early-life[ti] AND Underweight[ti]) OR (Early-life[ti] AND Leanness[ti]) OR (Early-life[ti] AND Thinness[ti]) OR (Early-life[ti] AND Slimness[ti]) OR (Early-life[ti] AND Overweight[ti]) OR (Early-life[ti] AND Obes*[ti]) OR (Early-life[tiab] AND Height*[tiab]) OR (Early-life[tiab] AND Weight*[tiab]) OR (Early-life[tiab] AND Length[tiab]) OR (Early-life[ti] AND “failure to thrive”[ti]) OR (“Early life”[tiab] AND Growth[tiab]) OR (“Early life”[ti] AND “Growth Retardation”[ti]) OR (“Early life”[ti] AND “Growth Disorder*”[ti]) OR (“Early life”[ti] AND “Growth Falter*”[ti]) OR (“Early life”[ti] AND “Growth Deficit*”[ti]) OR (“Early life”[ti] AND “Growth failure”[ti]) OR (“Early life”[ti] AND “Growth Trajectory”[ti]) OR (“Early life”[ti] AND “Nutrition Disorder*”[ti]) OR (“Early life”[tiab] AND Malnutrition[tiab]) OR (“Early life”[ti] AND Undernutrition[ti]) OR (“Early life”[ti] AND Under-nutrition[ti]) OR (“Early life”[ti] AND Undernourish*[ti]) OR (“Early life”[ti] AND Overnutrition[ti]) OR (“Early life”[ti] AND Underweight[ti]) OR (“Early life”[ti] AND Leanness[ti]) OR (“Early life”[ti] AND Thinness[ti]) OR (“Early life”[ti] AND Slimness[ti]) OR (“Early life”[ti] AND Overweight[ti]) OR (“Early life”[ti] AND Obes*[ti]) OR (“Early life”[tiab] AND Height*[tiab]) OR (“Early life”[tiab] AND Weight*[tiab]) OR (“Early life”[tiab] AND Length[tiab]) OR (“Early life”[ti] AND “failure to thrive”[ti]) OR (Pediatric[tiab] AND Growth[tiab]) OR (Pediatric[ti] AND “Growth Retardation”[ti]) OR (Pediatric[ti] AND “Growth Disorder*”[ti]) OR (Pediatric[ti] AND “Growth Falter*”[ti]) OR (Pediatric[ti] AND “Growth Deficit*”[ti]) OR (Pediatric[ti] AND “Growth failure”[ti]) OR (Pediatric[ti] AND “Growth Trajectory”[ti]) OR (Pediatric[ti] AND “Nutrition Disorder*”[ti]) OR (Pediatric[tiab] AND Malnutrition[tiab]) OR (Pediatric[ti] AND Undernutrition[ti]) OR (Pediatric[ti] AND Under-nutrition[ti]) OR (Pediatric[ti] AND Undernourish*[ti]) OR (Pediatric[ti] AND Overnutrition[ti]) OR (Pediatric[ti] AND Underweight[ti]) OR (Pediatric[ti] AND Leanness[ti]) OR (Pediatric[ti] AND Thinness[ti]) OR (Pediatric[ti] AND Slimness[ti]) OR (Pediatric[ti] AND Overweight[ti]) OR (Pediatric[ti] AND Obes*[ti]) OR (Pediatric[tiab] AND Height*[tiab]) OR (Pediatric[tiab] AND Weight*[tiab]) OR (Pediatric[tiab] AND Length[tiab]) OR (Pediatric[ti] AND “failure to thrive”[ti]) OR “child health”[ti])<br>AND<br>(cohort[tiab] OR longitudinal[tiab] OR (Cohort[tiab] AND Study[tiab]) OR (Concurrent[tiab] AND Study[tiab]) OR “birth cohort”[tiab] OR (cohort[tiab] AND analysis[tiab]) OR (cohort[tiab] AND analyses[tiab]) OR “incidence study”[tiab] OR “Follow Up Study”[tiab] OR “Follow-Up Study”[tiab] OR “Followup Study”[tiab] OR (Longitudinal[tiab] AND Study[tiab]) OR (Prospective[tiab] AND study[tiab]))<br>AND<br>(1990/1/01:2024/1/31[dp]))				
8	(((“Educational Status”[ti] AND Maternal[ti]) OR (Status[ti] AND “Maternal Educational”[ti]) OR “Maternal Educational Status”[ti] OR (Maternal[tiab] AND education[tiab]) OR (Maternal[ti] AND Illiteracy[ti]) OR (Maternal[ti] AND Literacy[ti]) OR (maternal[all] AND “schooling”[all]) OR (mother*[all] AND education[all]) OR (mother*[ti] AND Illiteracy[ti]) OR (mother*[ti] AND Literacy[ti]) OR (mother*[all] AND “schooling”[all]) OR (parent*[ti] AND Illiteracy[ti]) OR (parent*[ti] AND Literacy[ti]) OR (parent*[ti] AND education[ti]) OR (parent*[ti] AND “schooling”[ti]) OR (parent*[ti] AND socio-economic[ti]) OR (parent*[ti] AND socioeconomic[ti]) OR (parent*[ti] AND “Social Class*”[ti]) OR (parent*[ti] AND Socio-demographic[ti]) OR (parent*[ti] AND Sociodemographic[ti]) OR (parent*[ti] AND Socio-cultural[ti]) OR (parent*[ti] AND Sociocultural[ti]) OR (mother*[tiab] AND socio-economic[tiab]) OR (mother*[tiab] AND socioeconomic[tiab]) OR (mother*[ti] AND “Social Class*”[ti]) OR (mother*[tiab] AND Socio-demographic[tiab]) OR (mother*[tiab] AND Sociodemographic[tiab]) OR (mother*[ti] AND Socio-cultural[ti]) OR (mother*[ti] AND Sociocultural[ti]) OR (maternal[tiab] AND socio-economic[tiab]) OR (maternal[tiab] AND socioeconomic[tiab]) OR (maternal[ti] AND “Social Class*”[ti]) OR (maternal[tiab] AND Socio-demographic[tiab]) OR (maternal[tiab] AND Sociodemographic[tiab]) OR (maternal[ti] AND Socio-cultural[ti]) OR (maternal[ti] AND Sociocultural[ti]) OR “maternal socioeconomic status”[ti] OR “parental socioeconomic status”[ti])<br>AND<br>((Child*[tiab] AND Growth[tiab]) OR (Child*[ti] AND “Growth Retardation”[ti]) OR (Child*[ti] AND “Growth Disorder*”[ti]) OR (Child*[ti] AND “Growth Falter*”[ti]) OR (Child*[ti] AND “Growth Deficit*”[ti]) OR (Child*[ti] AND “Growth failure”[ti]) OR (Child*[ti] AND “Growth Trajectory”[ti]) OR Stunting*[ti] OR “Stunted Growth”[ti] OR (Growth[ti] AND Stunted[ti]) OR	25	4,345		

	“short stature”[ti] OR wast*[tiab] OR (Child*[ti] AND “Nutrition Disorder*”[ti]) OR (Child*[tiab] AND Malnutrition[tiab]) OR (Child*[ti] AND Undernutrition[ti]) OR (Child*[ti] AND Under-nutrition[ti]) OR (Child*[ti] AND Undernourish*[ti]) OR (Child*[ti] AND Overnutrition[ti]) OR (Child*[ti] AND Underweight[ti]) OR (Child*[ti] AND Leanness[ti]) OR (Child*[ti] AND Thinness[ti]) OR (Child*[ti] AND Slimness[ti]) OR (Child*[ti] AND Overweight[ti]) OR (Child*[ti] AND Obes*[ti]) OR (Child*[tiab] AND Height*[tiab]) OR (Child*[tiab] AND Weight*[tiab]) OR (Child*[tiab] AND Length[tiab]) OR Anthropometric*[ti] OR Length-for-age[tiab] OR Height-for-age[tiab] OR Weight-for-age[tiab] OR Weight-for-height[tiab] OR weight-for-length[tiab] OR “Body mass index-for-age”[tiab] OR BMI-for-age[tiab] OR (child*[ti] AND “failure to thrive”[ti]) OR (Infan*[tiab] AND Growth[tiab]) OR (Infan*[ti] AND “Growth Retardation”[ti]) OR (Infan*[ti] AND “Growth Disorder*”[ti]) OR (Infan*[ti] AND “Growth Falter*”[ti]) OR (Infan*[ti] AND “Growth Deficit*”[ti]) OR (Infan*[ti] AND “Growth failure”[ti]) OR (Infan*[ti] AND “Growth Trajectory”[ti]) OR (Infan*[ti] AND “Nutrition Disorder*”[ti]) OR (Infan*[tiab] AND Malnutrition[tiab]) OR (Infan*[ti] AND Undernutrition[ti]) OR (Infan*[ti] AND Under-nutrition[ti]) OR (Infan*[ti] AND Undernourish*[ti]) OR (Infan*[ti] AND Overnutrition[ti]) OR (Infan*[ti] AND Underweight[ti]) OR (Infan*[ti] AND Leanness[ti]) OR (Infan*[ti] AND Thinness[ti]) OR (Infan*[ti] AND Slimness[ti]) OR (Infan*[ti] AND Overweight[ti]) OR (Infan*[ti] AND Obes*[ti]) OR (Infan*[tiab] AND Height*[tiab]) OR (Infan*[tiab] AND Weight*[tiab]) OR (Infan*[tiab] AND Length[tiab]) OR (infan*[ti] AND “failure to thrive”[ti]) OR (Under-five[tiab] AND Growth[tiab]) OR (Under-five[ti] AND “Growth Retardation”[ti]) OR (Under-five[ti] AND “Growth Disorder*”[ti]) OR (Under-five[ti] AND “Growth Falter*”[ti]) OR (Under-five[ti] AND “Growth Deficit*”[ti]) OR (Under-five[ti] AND “Growth failure”[ti]) OR (Under-five[ti] AND “Growth Trajectory”[ti]) OR (Under-five[ti] AND “Nutrition Disorder*”[ti]) OR (Under-five[tiab] AND Malnutrition[tiab]) OR (Under-five[ti] AND Undernutrition[ti]) OR (Under-five[ti] AND Under-nutrition[ti]) OR (Under-five[ti] AND Undernourish*[ti]) OR (Under-five[ti] AND Overnutrition[ti]) OR (Under-five[ti] AND Underweight[ti]) OR (Under-five[tiab] AND Leanness[ti]) OR (Under-five[ti] AND Thinness[ti]) OR (Under-five[ti] AND Slimness[ti]) OR (Under-five[ti] AND Overweight[ti]) OR (Under-five[ti] AND Obes*[ti]) OR (Under-five[tiab] AND Height*[tiab]) OR (Under-five[tiab] AND Weight*[tiab]) OR (Under-five[tiab] AND Length[tiab]) OR (under-five[ti] AND “failure to thrive”[ti]) OR (“under five”[tiab] AND Growth[tiab]) OR (“under five”[ti] AND “Growth Retardation”[ti]) OR (“under five”[ti] AND “Growth Disorder*”[ti]) OR (“under five”[ti] AND “Growth Falter*”[ti]) OR (“under five”[ti] AND “Growth Deficit*”[ti]) OR (“under five”[ti] AND “Growth failure”[ti]) OR (“under five”[ti] AND “Growth Trajectory”[ti]) OR (“under five”[ti] AND “Nutrition Disorder*”[ti]) OR (“under five”[tiab] AND Malnutrition[tiab]) OR (“under five”[ti] AND Undernutrition[ti]) OR (“under five”[ti] AND Under-nutrition[ti]) OR (“under five”[ti] AND Undernourish*[ti]) OR (“under five”[ti] AND Overnutrition[ti]) OR (“under five”[ti] AND Underweight[ti]) OR (“under five”[ti] AND Leanness[ti]) OR (“under five”[ti] AND Thinness[ti]) OR (“under five”[ti] AND Slimness[ti]) OR (“under five”[ti] AND Overweight[ti]) OR (“under five”[ti] AND Obes*[ti]) OR (“under five”[tiab] AND Height*[tiab]) OR (“under five”[tiab] AND Weight*[tiab]) OR (“under five”[tiab] AND Length[tiab]) OR (“under five”[ti] AND “failure to thrive”[ti]) OR (“under 5”[tiab] AND Growth[tiab]) OR (“under 5”[ti] AND “Growth Retardation”[ti]) OR (“under 5”[ti] AND “Growth Disorder*”[ti]) OR (“under 5”[ti] AND “Growth Falter*”[ti]) OR (“under 5”[ti] AND “Growth Deficit*”[ti]) OR (“under 5”[ti] AND “Growth failure”[ti])OR (“under 5”[ti] AND “Growth Trajectory”[ti]) OR (“under 5”[ti] AND “Nutrition Disorder*”[ti]) OR (“under 5”[tiab] AND Malnutrition[tiab]) OR (“under 5”[ti] AND Undernutrition[ti]) OR (“under 5”[ti] AND Under-nutrition[ti]) OR (“under 5”[ti] AND Undernourish*[ti]) OR (“under 5”[ti] AND Overnutrition[ti]) OR (“under 5”[ti] AND Underweight[ti]) OR (“under 5”[ti] AND Leanness[ti]) OR (“under 5”[ti] AND Thinness[ti]) OR (“under 5”[ti] AND Slimness[ti]) OR (“under 5”[ti] AND Overweight[ti]) OR (“under 5”[ti] AND Obes*[ti]) OR (“under 5”[tiab] AND Height*[tiab]) OR (“under 5”[tiab] AND Weight*[tiab]) OR (“under 5”[tiab] AND Length[tiab]) OR (“under 5”[ti] AND “failure to thrive”[ti]) OR (Under-5[tiab] AND Growth[tiab]) OR (Under-5[ti] AND “Growth Retardation”[ti]) OR (Under-5[ti] AND “Growth Disorder*”[ti]) OR (Under-5[ti] AND “Growth Falter*”[ti]) OR (Under-5[ti] AND “Growth Deficit*”[ti]) OR (Under-5[ti] AND “Growth failure”[ti]) OR (Under-5[ti] AND “Growth Trajectory”[ti]) OR (Under-5[ti] AND “Nutrition Disorder*”[ti]) OR (Under-5[tiab] AND Malnutrition[tiab]) OR (Under-5[ti] AND Undernutrition[ti]) OR (Under-5[ti] AND Under-nutrition[ti]) OR (Under-5[ti] AND Undernourish*[ti]) OR (Under-5[ti] AND Overnutrition[ti]) OR (Under-5[ti] AND Underweight[ti]) OR (Under-5[ti] AND Leanness[ti]) OR (Under-5[ti] AND Thinness[ti]) OR (Under-5[ti] AND Slimness[ti]) OR (Under-5[ti] AND Overweight[ti]) OR (Under-5[ti] AND Obes*[ti]) OR (Under-5[tiab] AND Height*[tiab]) OR (Under-5[tiab] AND Weight*[tiab]) OR (Under-5[tiab] AND Length[tiab]) OR (Under-5[ti] AND “failure to thrive”[ti]) OR (Under-two[tiab] AND Growth[tiab]) OR (Under-two[ti] AND “Growth Retardation”[ti]) OR (Under-two[ti] AND “Growth Disorder*”[ti]) OR (Under-two[ti] AND “Growth Falter*”[ti]) OR (Under-two[ti] AND “Growth Deficit*”[ti]) OR (Under-two[ti] AND “Growth failure”[ti]) OR (Under-two[ti] AND “Growth Trajectory”[ti]) OR (Under-two[ti] AND “Nutrition Disorder*”[ti]) OR (Under-two[tiab] AND Malnutrition[tiab]) OR (Under-two[ti] AND Undernutrition[ti]) OR (Under-two[ti] AND Under-nutrition[ti]) OR (Under-two[ti] AND Undernourish*[ti]) OR (Under-two[ti] AND Overnutrition[ti]) OR (Under-two[ti] AND Underweight[ti]) OR (Under-two[ti] AND Leanness[ti]) OR (Under-two[ti] AND Thinness[ti]) OR (Under-two[ti] AND Slimness[ti]) OR (Under-two[tiab] AND Overweight[tiab]) OR (Under-two[ti] AND Obes*[ti]) OR (Under-two[tiab] AND Height*[tiab]) OR (Under-two[tiab] AND Weight*[tiab]) OR (Under-two[tiab] AND Length[tiab]) OR (Under-two[ti] AND “failure to thrive”[ti]) OR (“under two”[tiab] AND Growth[tiab]) OR (“under two”[ti] AND “Growth Retardation”[ti]) OR (“under two”[ti] AND				

	"Growth Disorder*"[ti]) OR ("under two"[ti] AND "Growth Falter*"[ti]) OR ("under two"[ti] AND "Growth Deficit*"[ti]) OR ("under two"[ti] AND "Growth failure"[ti]) OR ("under two"[ti] AND "Growth Trajectory"[ti]) OR ("under two"[ti] AND "Nutrition Disorder*"[ti]) OR ("under two"[tiab] AND Malnutrition[tiab]) OR ("under two"[ti] AND Undernutrition[ti]) OR ("under two"[ti] AND Under-nutrition[ti]) OR ("under two"[ti] AND Undernourish*[ti]) OR ("under two"[ti] AND Overnutrition[ti]) OR ("under two"[ti] AND Underweight[ti]) OR ("under two"[ti] AND Leanness[ti]) OR ("under two"[ti] AND Thinness[ti]) OR ("under two"[ti] AND Slimness[ti]) OR ("under two"[ti] AND Overweight[ti]) OR ("under two"[ti] AND Obes*[ti]) OR ("under two"[tiab] AND Height*[tiab]) OR ("under two"[tiab] AND Weight*[tiab]) OR ("under two"[tiab] AND Length[tiab]) OR ("under two"[ti] AND "failure to thrive"[ti]) OR ("under 2"[tiab] AND Growth[tiab]) OR ("under 2"[ti] AND "Growth Retardation"[ti]) OR ("under 2"[ti] AND "Growth Disorder*"[ti]) OR ("under 2"[ti] AND "Growth Falter*"[ti]) OR ("under 2"[ti] AND "Growth Deficit*"[ti]) OR ("under 2"[ti] AND "Growth failure"[ti]) OR ("under 2"[ti] AND "Growth Trajectory"[ti]) OR ("under 2"[ti] AND "Nutrition Disorder*"[ti]) OR ("under 2"[tiab] AND Malnutrition[tiab]) OR ("under 2"[ti] AND Undernutrition[ti]) OR ("under 2"[ti] AND Under-nutrition[ti]) OR ("under 2"[ti] AND Undernourish*[ti]) OR ("under 2"[ti] AND Overnutrition[ti]) OR ("under 2"[ti] AND Underweight[ti]) OR ("under 2"[ti] AND Leanness[ti]) OR ("under 2"[ti] AND Thinness[ti]) OR ("under 2"[ti] AND Slimness[ti]) OR ("under 2"[ti] AND Overweight[ti]) OR ("under 2"[ti] AND Obes*[ti]) OR ("under 2"[tiab] AND Height*[tiab]) OR ("under 2"[tiab] AND Weight*[tiab]) OR ("under 2"[tiab] AND Length[tiab]) OR ("under 2"[ti] AND "failure to thrive"[ti]) OR (Under-2[tiab] AND Growth[tiab]) OR (Under-2[ti] AND "Growth Retardation"[ti]) OR (Under-2[ti] AND "Growth Disorder*"[ti]) OR (Under-2[ti] AND "Growth Falter*"[ti]) OR (Under-2[ti] AND "Growth Deficit*"[ti]) OR (Under-2[ti] AND "Growth failure"[ti]) OR (Under-2[ti] AND "Growth Trajectory"[ti]) OR (Under-2[ti] AND "Nutrition Disorder*"[ti]) OR (Under-2[tiab] AND Malnutrition[tiab]) OR (Under-2[ti] AND Undernutrition[ti]) OR (Under-2[ti] AND Under-nutrition[ti]) OR (Under-2[ti] AND Undernourish*[ti]) OR (Under-2[ti] AND Overnutrition[ti]) OR (Under-2[ti] AND Underweight[ti]) OR (Under-2[ti] AND Leanness[ti]) OR (Under-2[ti] AND Thinness[ti]) OR (Under-2[ti] AND Slimness[ti]) OR (Under-2[ti] AND Overweight[ti]) OR (Under-2[ti] AND Obes*[ti]) OR (Under-2[tiab] AND Height*[tiab]) OR (Under-2[tiab] AND Weight*[tiab]) OR (Under-2[tiab] AND Length[tiab]) OR (Under-2[ti] AND "failure to thrive"[ti]) OR (Offspring[tiab] AND Growth[tiab]) OR (Offspring[ti] AND "Growth Retardation"[ti]) OR (Offspring[ti] AND "Growth Disorder*"[ti]) OR (Offspring[ti] AND "Growth Falter*"[ti]) OR (Offspring[ti] AND "Growth Deficit*"[ti]) OR (Offspring[ti] AND "Growth failure"[ti]) OR (Offspring[ti] AND "Growth Trajectory"[ti]) OR (Offspring[ti] AND "Nutrition Disorder*"[ti]) OR (Offspring[tiab] AND Malnutrition[tiab]) OR (Offspring[ti] AND Undernutrition[ti]) OR (Offspring[ti] AND Under-nutrition[ti]) OR (Offspring[ti] AND Undernourish*[ti]) OR (Offspring[ti] AND Overnutrition[ti]) OR (Offspring[ti] AND Underweight[ti]) OR (Offspring[ti] AND Leanness[ti]) OR (Offspring[ti] AND Thinness[ti]) OR (Offspring[ti] AND Slimness[ti]) OR (Offspring[ti] AND Overweight[ti]) OR (Offspring[ti] AND Obes*[ti]) OR (Offspring[tiab] AND Height*[tiab]) OR (Offspring[tiab] AND Weight*[tiab]) OR (Offspring[tiab] AND Length[tiab]) OR (Offspring[ti] AND "failure to thrive"[ti]) OR (Early-life[tiab] AND Growth[tiab]) OR (Early-life[ti] AND "Growth Retardation"[ti]) OR (Early-life[ti] AND "Growth Disorder*"[ti]) OR (Early-life[ti] AND "Growth Falter*"[ti]) OR (Early-life[ti] AND "Growth Deficit*"[ti]) OR (Early-life[ti] AND "Growth failure"[ti]) OR (Early-life[ti] AND "Growth Trajectory"[ti]) OR (Early-life[ti] AND "Nutrition Disorder*"[ti]) OR (Early-life[tiab] AND Malnutrition[tiab]) OR (Early-life[ti] AND Undernutrition[ti]) OR (Early-life[ti] AND Under-nutrition[ti]) OR (Early-life[ti] AND Undernourish*[ti]) OR (Early-life[ti] AND Overnutrition[ti]) OR (Early-life[ti] AND Underweight[ti]) OR (Early-life[ti] AND Leanness[ti]) OR (Early-life[ti] AND Thinness[ti]) OR (Early-life[ti] AND Slimness[ti]) OR (Early-life[ti] AND Overweight[ti]) OR (Early-life[ti] AND Obes*[ti]) OR (Early-life[tiab] AND Height*[tiab]) OR (Early-life[tiab] AND Weight*[tiab]) OR (Early-life[tiab] AND Length[tiab]) OR (Early-life[ti] AND "failure to thrive"[ti]) OR ("Early life"[tiab] AND Growth[tiab]) OR ("Early life"[ti] AND "Growth Retardation"[ti]) OR ("Early life"[ti] AND "Growth Disorder*"[ti]) OR ("Early life"[ti] AND "Growth Falter*"[ti]) OR ("Early life"[ti] AND "Growth Deficit*"[ti]) OR ("Early life"[ti] AND "Growth failure"[ti]) OR ("Early life"[ti] AND "Growth Trajectory"[ti]) OR ("Early life"[ti] AND "Nutrition Disorder*"[ti]) OR ("Early life"[tiab] AND Malnutrition[tiab]) OR ("Early life"[ti] AND Undernutrition[ti]) OR ("Early life"[ti] AND Under-nutrition[ti]) OR ("Early life"[ti] AND Undernourish*[ti]) OR ("Early life"[ti] AND Overnutrition[ti]) OR ("Early life"[ti] AND Underweight[ti]) OR ("Early life"[ti] AND Leanness[ti]) OR ("Early life"[ti] AND Thinness[ti]) OR ("Early life"[ti] AND Slimness[ti]) OR ("Early life"[ti] AND Overweight[ti]) OR ("Early life"[ti] AND Obes*[ti]) OR ("Early life"[tiab] AND Height*[tiab]) OR ("Early life"[tiab] AND Weight*[tiab]) OR ("Early life"[tiab] AND Length[tiab]) OR ("Early life"[ti] AND "failure to thrive"[ti]) OR (Pediatric[tiab] AND Growth[tiab]) OR (Pediatric[ti] AND "Growth Retardation"[ti]) OR (Pediatric[ti] AND "Growth Disorder*"[ti]) OR (Pediatric[ti] AND "Growth Falter*"[ti]) OR (Pediatric[ti] AND "Growth Deficit*"[ti]) OR (Pediatric[ti] AND "Growth failure"[ti]) OR (Pediatric[ti] AND "Growth Trajectory"[ti]) OR (Pediatric[ti] AND "Nutrition Disorder*"[ti]) OR (Pediatric[tiab] AND Malnutrition[tiab]) OR (Pediatric[ti] AND Undernutrition[ti]) OR (Pediatric[ti] AND Under-nutrition[ti]) OR (Pediatric[ti] AND Undernourish*[ti]) OR (Pediatric[ti] AND Overnutrition[ti]) OR (Pediatric[ti] AND Underweight[ti]) OR (Pediatric[ti] AND Leanness[ti]) OR (Pediatric[ti] AND Thinness[ti]) OR (Pediatric[ti] AND Slimness[ti]) OR (Pediatric[ti] AND Overweight[ti]) OR (Pediatric[ti] AND Obes*[ti]) OR (Pediatric[tiab] AND Height*[tiab]) OR (Pediatric[tiab] AND Weight*[tiab]) OR (Pediatric[tiab] AND Length[tiab]) OR (Pediatric[ti] AND "failure to thrive"[ti]) OR "child health"[ti])				

	AND<br>(cohort[tiab] OR longitudinal[tiab] OR (Cohort[tiab] AND Study[tiab]) OR (Concurrent[tiab] AND Study[tiab]) OR “birth cohort”[tiab] OR (cohort[tiab] AND analysis[tiab]) OR (cohort[tiab] AND analyses[tiab]) OR “incidence study”[tiab] OR “Follow Up Study”[tiab] OR “Follow-Up Study”[tiab] OR “Followup Study”[tiab] OR (Longitudinal[tiab] AND Study[tiab]) OR (Prospective[tiab] AND study[tiab]))<br>AND<br>(1990/1/01:2024/1/31[dp])))				
9	(((“Educational Status”[ti] AND Maternal[ti]) OR (Status[ti] AND “Maternal Educational”[ti]) OR “Maternal Educational Status”[ti] OR (Maternal[tiab] AND education[tiab]) OR (Maternal[ti] AND Illiteracy[ti]) OR (Maternal[ti] AND Literacy[ti]) OR (maternal[all] AND “schooling”[all]) OR (mother*[tiab] AND education[tiab]) OR (mother*[ti] AND Illiteracy[ti]) OR (mother*[ti] AND Literacy[ti]) OR (mother*[all] AND “schooling”[all]) OR (parent*[ti] AND Illiteracy[ti]) OR (parent*[ti] AND Literacy[ti]) OR (parent*[ti] AND education[ti]) OR (parent*[ti] AND “schooling”[ti]) OR (parent*[ti] AND socio-economic[ti]) OR (parent*[ti] AND socioeconomic[ti]) OR (parent*[ti] AND “Social Class*”[ti]) OR (parent*[ti] AND Socio-demographic[ti]) OR (parent*[ti] AND Sociodemographic[ti]) OR (parent*[ti] AND Socio-cultural[ti]) OR (parent*[ti] AND Sociocultural[ti]) OR (mother*[tiab] AND socio-economic[tiab]) OR (mother*[tiab] AND socioeconomic[tiab]) OR (mother*[ti] AND “Social Class*”[ti]) OR (mother*[tiab] AND Socio-demographic[tiab]) OR (mother*[tiab] AND Sociodemographic[tiab]) OR (mother*[ti] AND Socio-cultural[ti]) OR (mother*[ti] AND Sociocultural[ti]) OR (maternal[tiab] AND socio-economic[tiab]) OR (maternal[tiab] AND socioeconomic[tiab]) OR (maternal[ti] AND “Social Class*”[ti]) OR (maternal[tiab] AND Socio-demographic[tiab]) OR (maternal[tiab] AND Sociodemographic[tiab]) OR (maternal[ti] AND Socio-cultural[ti]) OR (maternal[ti] AND Sociocultural[ti]) OR “maternal socioeconomic status”[ti] OR “parental socioeconomic status”[ti])<br>AND<br>((Child*[tiab] AND Growth[tiab]) OR (Child*[ti] AND “Growth Retardation”[ti]) OR (Child*[ti] AND “Growth Disorder*”[ti]) OR (Child*[ti] AND “Growth Falter*”[ti]) OR (Child*[ti] AND “Growth Deficit*”[ti]) OR (Child*[ti] AND “Growth failure”[ti]) OR (Child*[ti] AND “Growth Trajectory”[ti]) OR Stunting*[ti] OR “Stunted Growth”[ti] OR (Growth[ti] AND Stunted[ti]) OR “short stature”[ti] OR wast*[tiab] OR (Child*[ti] AND “Nutrition Disorder*”[ti]) OR (Child*[tiab] AND Malnutrition[tiab]) OR (Child*[ti] AND Undernutrition[ti]) OR (Child*[ti] AND Under-nutrition[ti]) OR (Child*[ti] AND Undernourish*[ti]) OR (Child*[ti] AND Overnutrition[ti]) OR (Child*[ti] AND Underweight[ti]) OR (Child*[ti] AND Leanness[ti]) OR (Child*[ti] AND Thinness[ti]) OR (Child*[ti] AND Slimness[ti]) OR (Child*[ti] AND Overweight[ti]) OR (Child*[ti] AND Obes*[ti]) OR (Child*[tiab] AND Height*[tiab]) OR (Child*[tiab] AND Weight*[tiab]) OR (Child*[tiab] AND Length[tiab]) OR Anthropometric*[ti] OR Length-for-age[tiab] OR Height-for-age[tiab] OR Weight-for-age[tiab] OR Weight-for-height[tiab] OR weight-for-length[tiab] OR “Body mass index-for-age”[tiab] OR BMI-for-age[tiab] OR (child*[ti] AND “failure to thrive”[ti]) OR (Infan*[tiab] AND Growth[tiab]) OR (Infan*[ti] AND “Growth Retardation”[ti]) OR (Infan*[ti] AND “Growth Disorder*”[ti]) OR (Infan*[ti] AND “Growth Falter*”[ti]) OR (Infan*[ti] AND “Growth Deficit*”[ti]) OR (Infan*[ti] AND “Growth failure”[ti]) OR (Infan*[ti] AND “Growth Trajectory”[ti]) OR (Infan*[ti] AND “Nutrition Disorder*”[ti]) OR (Infan*[tiab] AND Malnutrition[tiab]) OR (Infan*[ti] AND Undernutrition[ti]) OR (Infan*[ti] AND Under-nutrition[ti]) OR (Infan*[ti] AND Undernourish*[ti]) OR (Infan*[ti] AND Overnutrition[ti]) OR (Infan*[ti] AND Underweight[ti]) OR (Infan*[ti] AND Leanness[ti]) OR (Infan*[ti] AND Thinness[ti]) OR (Infan*[ti] AND Slimness[ti]) OR (Infan*[ti] AND Overweight[ti]) OR (Infan*[ti] AND Obes*[ti]) OR (Infan*[tiab] AND Height*[tiab]) OR (Infan*[tiab] AND Weight*[tiab]) OR (Infan*[tiab] AND Length[tiab]) OR (infan*[ti] AND “failure to thrive”[ti]) OR (Under-five[tiab] AND Growth[tiab]) OR (Under-five[ti] AND “Growth Retardation”[ti]) OR (Under-five[ti] AND “Growth Disorder*”[ti]) OR (Under-five[ti] AND “Growth Falter*”[ti]) OR (Under-five[ti] AND “Growth Deficit*”[ti]) OR (Under-five[ti] AND “Growth failure”[ti]) OR (Under-five[ti] AND “Growth Trajectory”[ti]) OR (Under-five[ti] AND “Nutrition Disorder*”[ti]) OR (Under-five[tiab] AND Malnutrition[tiab]) OR (Under-five[ti] AND Undernutrition[ti]) OR (Under-five[ti] AND Under-nutrition[ti]) OR (Under-five[ti] AND Undernourish*[ti]) OR (Under-five[ti] AND Overnutrition[ti]) OR (Under-five[ti] AND Underweight[ti]) OR (Under-five[tiab] AND Leanness[ti]) OR (Under-five[ti] AND Thinness[ti]) OR (Under-five[ti] AND Slimness[ti]) OR (Under-five[ti] AND Overweight[ti]) OR (Under-five[ti] AND Obes*[ti]) OR (Under-five[tiab] AND Height*[tiab]) OR (Under-five[tiab] AND Weight*[tiab]) OR (Under-five[tiab] AND Length[tiab]) OR (under-five[ti] AND “failure to thrive”[ti]) OR (“under five”[tiab] AND Growth[tiab]) OR (“under five”[ti] AND “Growth Retardation”[ti]) OR (“under five”[ti] AND “Growth Disorder*”[ti]) OR (“under five”[ti] AND “Growth Falter*”[ti]) OR (“under five”[ti] AND “Growth Deficit*”[ti]) OR (“under five”[ti] AND “Growth failure”[ti]) OR (“under five”[ti] AND “Growth Trajectory”[ti]) OR (“under five”[ti] AND “Nutrition Disorder*”[ti]) OR (“under five”[tiab] AND Malnutrition[tiab]) OR (“under five”[ti] AND Undernutrition[ti]) OR (“under five”[ti] AND Under-nutrition[ti]) OR (“under five”[ti] AND Undernourish*[ti]) OR (“under five”[ti] AND Overnutrition[ti]) OR (“under five”[ti] AND Underweight[ti]) OR (“under five”[ti] AND Leanness[ti]) OR (“under five”[ti] AND Thinness[ti]) OR (“under five”[ti] AND Slimness[ti]) OR (“under five”[ti] AND Overweight[ti]) OR (“under five”[ti] AND Obes*[ti]) OR (“under five”[tiab] AND Height*[tiab]) OR (“under five”[tiab] AND Weight*[tiab]) OR (“under five”[tiab] AND Length[tiab]) OR (“under five”[ti] AND “failure to thrive”[ti]) OR (“under 5”[tiab] AND Growth[tiab]) OR (“under 5”[ti] AND “Growth Retardation”[ti]) OR (“under 5”[ti] AND “Growth Disorder*”[ti]) OR (“under 5”[ti] AND “Growth Falter*”[ti]) OR (“under 5”[ti] AND “Growth Deficit*”[ti]) OR (“under 5”[ti] AND “Growth	20	3,486		

	failure”[ti])OR (“under 5”[ti] AND “Growth Trajectory”[ti]) OR (“under 5”[ti] AND “Nutrition Disorder*”[ti]) OR (“under 5”[tiab] AND Malnutrition[tiab]) OR (“under 5”[ti] AND Undernutrition[ti]) OR (“under 5”[ti] AND Under-nutrition[ti]) OR (“under 5”[ti] AND Undernourish*[ti]) OR (“under 5”[ti] AND Overnutrition[ti]) OR (“under 5”[ti] AND Underweight[ti]) OR (“under 5”[ti] AND Leanness[ti]) OR (“under 5”[ti] AND Thinness[ti]) OR (“under 5”[ti] AND Slimness[ti]) OR (“under 5”[ti] AND Overweight[ti]) OR (“under 5”[ti] AND Obes*[ti]) OR (“under 5”[tiab] AND Height*[tiab]) OR (“under 5”[tiab] AND Weight*[tiab]) OR (“under 5”[tiab] AND Length[tiab]) OR (“under 5”[ti] AND “failure to thrive”[ti]) OR (Under-5[tiab] AND Growth[tiab]) OR (Under-5[ti] AND “Growth Retardation”[ti]) OR (Under-5[ti] AND “Growth Disorder*”[ti]) OR (Under-5[ti] AND “Growth Falter*”[ti]) OR (Under-5[ti] AND “Growth Deficit*”[ti]) OR (Under-5[ti] AND “Growth failure”[ti]) OR (Under-5[ti] AND “Growth Trajectory”[ti]) OR (Under-5[ti] AND “Nutrition Disorder*”[ti]) OR (Under-5[tiab] AND Malnutrition[tiab]) OR (Under-5[ti] AND Undernutrition[ti]) OR (Under-5[ti] AND Under-nutrition[ti]) OR (Under-5[ti] AND Undernourish*[ti]) OR (Under-5[ti] AND Overnutrition[ti]) OR (Under-5[ti] AND Underweight[ti]) OR (Under-5[ti] AND Leanness[ti]) OR (Under-5[ti] AND Thinness[ti]) OR (Under-5[ti] AND Slimness[ti]) OR (Under-5[ti] AND Overweight[ti]) OR (Under-5[ti] AND Obes*[ti]) OR (Under-5[tiab] AND Height*[tiab]) OR (Under-5[tiab] AND Weight*[tiab]) OR (Under-5[tiab] AND Length[tiab]) OR (Under-5[ti] AND “failure to thrive”[ti]) OR (Under-two[tiab] AND Growth[tiab]) OR (Under-two[ti] AND “Growth Retardation”[ti]) OR (Under-two[ti] AND “Growth Disorder*”[ti]) OR (Under-two[ti] AND “Growth Falter*”[ti]) OR (Under-two[ti] AND “Growth Deficit*”[ti]) OR (Under-two[ti] AND “Growth failure”[ti]) OR (Under-two[ti] AND “Growth Trajectory”[ti]) OR (Under-two[ti] AND “Nutrition Disorder*”[ti]) OR (Under-two[tiab] AND Malnutrition[tiab]) OR (Under-two[ti] AND Undernutrition[ti]) OR (Under-two[ti] AND Under-nutrition[ti]) OR (Under-two[ti] AND Undernourish*[ti]) OR (Under-two[ti] AND Overnutrition[ti]) OR (Under-two[ti] AND Underweight[ti]) OR (Under-two[ti] AND Leanness[ti]) OR (Under-two[ti] AND Thinness[ti]) OR (Under-two[ti] AND Slimness[ti]) OR (Under-two[tiab] AND Overweight[tiab]) OR (Under-two[ti] AND Obes*[ti]) OR (Under-two[tiab] AND Height*[tiab]) OR (Under-two[tiab] AND Weight*[tiab]) OR (Under-two[tiab] AND Length[tiab]) OR (Under-two[ti] AND “failure to thrive”[ti]) OR (“under two”[tiab] AND Growth[tiab]) OR (“under two”[ti] AND “Growth Retardation”[ti]) OR (“under two”[ti] AND “Growth Disorder*”[ti]) OR (“under two”[ti] AND “Growth Falter*”[ti]) OR (“under two”[ti] AND “Growth Deficit*”[ti]) OR (“under two”[ti] AND “Growth failure”[ti]) OR (“under two”[ti] AND “Growth Trajectory”[ti]) OR (“under two”[ti] AND “Nutrition Disorder*”[ti]) OR (“under two”[tiab] AND Malnutrition[tiab]) OR (“under two”[ti] AND Undernutrition[ti]) OR (“under two”[ti] AND Under-nutrition[ti]) OR (“under two”[ti] AND Undernourish*[ti]) OR (“under two”[ti] AND Overnutrition[ti]) OR (“under two”[ti] AND Underweight[ti]) OR (“under two”[ti] AND Leanness[ti]) OR (“under two”[ti] AND Thinness[ti]) OR (“under two”[ti] AND Slimness[ti]) OR (“under two”[ti] AND Overweight[ti]) OR (“under two”[ti] AND Obes*[ti]) OR (“under two”[tiab] AND Height*[tiab]) OR (“under two”[tiab] AND Weight*[tiab]) OR (“under two”[tiab] AND Length[tiab]) OR (“under two”[ti] AND “failure to thrive”[ti]) OR (“under 2”[tiab] AND Growth[tiab]) OR (“under 2”[ti] AND “Growth Retardation”[ti]) OR (“under 2”[ti] AND “Growth Disorder*”[ti]) OR (“under 2”[ti] AND “Growth Falter*”[ti]) OR (“under 2”[ti] AND “Growth Deficit*”[ti]) OR (“under 2”[ti] AND “Growth failure”[ti]) OR (“under 2”[ti] AND “Growth Trajectory”[ti]) OR (“under 2”[ti] AND “Nutrition Disorder*”[ti]) OR (“under 2”[tiab] AND Malnutrition[tiab]) OR (“under 2”[ti] AND Undernutrition[ti]) OR (“under 2”[ti] AND Under-nutrition[ti]) OR (“under 2”[ti] AND Undernourish*[ti]) OR (“under 2”[ti] AND Overnutrition[ti]) OR (“under 2”[ti] AND Underweight[ti]) OR (“under 2”[ti] AND Leanness[ti]) OR (“under 2”[ti] AND Thinness[ti]) OR (“under 2”[ti] AND Slimness[ti]) OR (“under 2”[ti] AND Overweight[ti]) OR (“under 2”[ti] AND Obes*[ti]) OR (“under 2”[tiab] AND Height*[tiab]) OR (“under 2”[tiab] AND Weight*[tiab]) OR (“under 2”[tiab] AND Length[tiab]) OR (“under 2”[ti] AND “failure to thrive”[ti]) OR (Under-2[tiab] AND Growth[tiab]) OR (Under-2[ti] AND “Growth Retardation”[ti]) OR (Under-2[ti] AND “Growth Disorder*”[ti]) OR (Under-2[ti] AND “Growth Falter*”[ti]) OR (Under-2[ti] AND “Growth Deficit*”[ti]) OR (Under-2[ti] AND “Growth failure”[ti]) OR (Under-2[ti] AND “Growth Trajectory”[ti]) OR (Under-2[ti] AND “Nutrition Disorder*”[ti]) OR (Under-2[tiab] AND Malnutrition[tiab]) OR (Under-2[ti] AND Undernutrition[ti]) OR (Under-2[ti] AND Under-nutrition[ti]) OR (Under-2[ti] AND Undernourish*[ti]) OR (Under-2[ti] AND Overnutrition[ti]) OR (Under-2[ti] AND Underweight[ti]) OR (Under-2[ti] AND Leanness[ti]) OR (Under-2[ti] AND Thinness[ti]) OR (Under-2[ti] AND Slimness[ti]) OR (Under-2[ti] AND Overweight[ti]) OR (Under-2[ti] AND Obes*[ti]) OR (Under-2[tiab] AND Height*[tiab]) OR (Under-2[tiab] AND Weight*[tiab]) OR (Under-2[tiab] AND Length[tiab]) OR (Under-2[ti] AND “failure to thrive”[ti]) OR (Offspring[tiab] AND Growth[tiab]) OR (Offspring[ti] AND “Growth Retardation”[ti]) OR (Offspring[ti] AND “Growth Disorder*”[ti]) OR (Offspring[ti] AND “Growth Falter*”[ti]) OR (Offspring[ti] AND “Growth Deficit*”[ti]) OR (Offspring[ti] AND “Growth failure”[ti]) OR (Offspring[ti] AND “Growth Trajectory”[ti]) OR (Offspring[ti] AND “Nutrition Disorder*”[ti]) OR (Offspring[tiab] AND Malnutrition[tiab]) OR (Offspring[ti] AND Undernutrition[ti]) OR (Offspring[ti] AND Under-nutrition[ti]) OR (Offspring[ti] AND Undernourish*[ti]) OR (Offspring[ti] AND Overnutrition[ti]) OR (Offspring[ti] AND Underweight[ti]) OR (Offspring[ti] AND Leanness[ti]) OR (Offspring[ti] AND Thinness[ti]) OR (Offspring[ti] AND Slimness[ti]) OR (Offspring[ti] AND Overweight[ti]) OR (Offspring[ti] AND Obes*[ti]) OR (Offspring[tiab] AND Height*[tiab]) OR (Offspring[tiab] AND Weight*[tiab]) OR (Offspring[tiab] AND Length[tiab]) OR (Offspring[ti] AND “failure to thrive”[ti]) OR (Early-life[tiab] AND Growth[tiab]) OR (Early-life[ti] AND “Growth Retardation”[ti]) OR (Early-life[ti] AND “Growth Disorder*”[ti]) OR (Early-life[ti] AND “Growth Falter*”[ti]) OR (Early-life[ti] AND				

	"Growth Deficit*"[ti]) OR (Early-life[ti] AND "Growth failure"[ti]) OR (Early-life[ti] AND "Growth Trajectory"[ti]) OR (Early-life[ti] AND "Nutrition Disorder*"[ti]) OR (Early-life[tiab] AND Malnutrition[tiab]) OR (Early-life[ti] AND Undernutrition[ti]) OR (Early-life[ti] AND Under-nutrition[ti]) OR (Early-life[ti] AND Undernourish*[ti]) OR (Early-life[ti] AND Overnutrition[ti]) OR (Early-life[ti] AND Underweight[ti]) OR (Early-life[ti] AND Leanness[ti]) OR (Early-life[ti] AND Thinness[ti]) OR (Early-life[ti] AND Slimness[ti]) OR (Early-life[ti] AND Overweight[ti]) OR (Early-life[ti] AND Obes*[ti]) OR (Early-life[tiab] AND Height*[tiab]) OR (Early-life[tiab] AND Weight*[tiab]) OR (Early-life[tiab] AND Length[tiab]) OR (Early-life[ti] AND "failure to thrive"[ti]) OR ("Early life"[tiab] AND Growth[tiab]) OR ("Early life"[ti] AND "Growth Retardation"[ti]) OR ("Early life"[ti] AND "Growth Disorder*"[ti]) OR ("Early life"[ti] AND "Growth Falter*"[ti]) OR ("Early life"[ti] AND "Growth Deficit*"[ti]) OR ("Early life"[ti] AND "Growth failure"[ti]) OR ("Early life"[ti] AND "Growth Trajectory"[ti]) OR ("Early life"[ti] AND "Nutrition Disorder*"[ti]) OR ("Early life"[tiab] AND Malnutrition[tiab]) OR ("Early life"[ti] AND Undernutrition[ti]) OR ("Early life"[ti] AND Under-nutrition[ti]) OR ("Early life"[ti] AND Undernourish*[ti]) OR ("Early life"[ti] AND Overnutrition[ti]) OR ("Early life"[ti] AND Underweight[ti]) OR ("Early life"[ti] AND Leanness[ti]) OR ("Early life"[ti] AND Thinness[ti]) OR ("Early life"[ti] AND Slimness[ti]) OR ("Early life"[ti] AND Overweight[ti]) OR ("Early life"[ti] AND Obes*[ti]) OR ("Early life"[tiab] AND Height*[tiab]) OR ("Early life"[tiab] AND Weight*[tiab]) OR ("Early life"[tiab] AND Length[tiab]) OR ("Early life"[ti] AND "failure to thrive"[ti]) OR (Pediatric[tiab] AND Growth[tiab]) OR (Pediatric[ti] AND "Growth Retardation"[ti]) OR (Pediatric[ti] AND "Growth Disorder*"[ti]) OR (Pediatric[ti] AND "Growth Falter*"[ti]) OR (Pediatric[ti] AND "Growth Deficit*"[ti]) OR (Pediatric[ti] AND "Growth failure"[ti]) OR (Pediatric[ti] AND "Growth Trajectory"[ti]) OR (Pediatric[ti] AND "Nutrition Disorder*"[ti]) OR (Pediatric[tiab] AND Malnutrition[tiab]) OR (Pediatric[ti] AND Undernutrition[ti]) OR (Pediatric[ti] AND Under-nutrition[ti]) OR (Pediatric[ti] AND Undernourish*[ti]) OR (Pediatric[ti] AND Overnutrition[ti]) OR (Pediatric[ti] AND Underweight[ti]) OR (Pediatric[ti] AND Leanness[ti]) OR (Pediatric[ti] AND Thinness[ti]) OR (Pediatric[ti] AND Slimness[ti]) OR (Pediatric[ti] AND Overweight[ti]) OR (Pediatric[ti] AND Obes*[ti]) OR (Pediatric[tiab] AND Height*[tiab]) OR (Pediatric[tiab] AND Weight*[tiab]) OR (Pediatric[tiab] AND Length[tiab]) OR (Pediatric[ti] AND "failure to thrive"[ti]) OR "child health"[ti])<br>AND<br>(cohort[tiab] OR longitudinal[tiab] OR (Cohort[tiab] AND Study[tiab]) OR (Concurrent[tiab] AND Study[tiab]) OR "birth cohort"[tiab] OR (cohort[tiab] AND analysis[tiab]) OR (cohort[tiab] AND analyses[tiab]) OR "incidence study"[tiab] OR "Follow Up Study"[tiab] OR "Follow-Up Study"[tiab] OR "Followup Study"[tiab] OR (Longitudinal[tiab] AND Study[tiab]) OR (Prospective[tiab] AND study[tiab]))<br>AND<br>(1990/1/01:2024/1/31[dp]))				
10	((("Educational Status"[ti] AND Maternal[ti]) OR (Status[ti] AND "Maternal Educational"[ti]) OR "Maternal Educational Status"[ti] OR (Maternal[ti] AND education[ti]) OR (Maternal[ti] AND Illiteracy[ti]) OR (Maternal[ti] AND Literacy[ti]) OR (maternal[all] AND "schooling"[all]) OR (mother*[tiab] AND education[tiab]) OR (mother*[ti] AND Illiteracy[ti]) OR (mother*[ti] AND Literacy[ti]) OR (mother*[all] AND "schooling"[all]) OR (parent*[ti] AND Illiteracy[ti]) OR (parent*[ti] AND Literacy[ti]) OR (parent*[ti] AND education[ti]) OR (parent*[ti] AND "schooling"[ti]) OR (parent*[ti] AND socio-economic[ti]) OR (parent*[ti] AND socioeconomic[ti]) OR (parent*[ti] AND "Social Class*"[ti]) OR (parent*[ti] AND Socio-demographic[ti]) OR (parent*[ti] AND Sociodemographic[ti]) OR (parent*[ti] AND Socio-cultural[ti]) OR (parent*[ti] AND Sociocultural[ti]) OR (mother*[tiab] AND socio-economic[tiab]) OR (mother*[tiab] AND socioeconomic[tiab]) OR (mother*[ti] AND "Social Class*"[ti]) OR (mother*[tiab] AND Socio-demographic[tiab]) OR (mother*[tiab] AND Sociodemographic[tiab]) OR (mother*[ti] AND Socio-cultural[ti]) OR (mother*[ti] AND Sociocultural[ti]) OR (maternal[tiab] AND socio-economic[tiab]) OR (maternal[tiab] AND socioeconomic[tiab]) OR (maternal[ti] AND "Social Class*"[ti]) OR (maternal[tiab] AND Socio-demographic[tiab]) OR (maternal[tiab] AND Sociodemographic[tiab]) OR (maternal[ti] AND Socio-cultural[ti]) OR (maternal[ti] AND Sociocultural[ti]) OR "maternal socioeconomic status"[ti] OR "parental socioeconomic status"[ti])<br>AND<br>((Child*[tiab] AND Growth[tiab]) OR (Child*[ti] AND "Growth Retardation"[ti]) OR (Child*[ti] AND "Growth Disorder*"[ti]) OR (Child*[ti] AND "Growth Falter*"[ti]) OR (Child*[ti] AND "Growth Deficit*"[ti]) OR (Child*[ti] AND "Growth failure"[ti]) OR (Child*[ti] AND "Growth Trajectory"[ti]) OR Stunting*[ti] OR "Stunted Growth"[ti] OR (Growth[ti] AND Stunted[ti]) OR "short stature"[ti] OR wast*[tiab] OR (Child*[ti] AND "Nutrition Disorder*"[ti]) OR (Child*[tiab] AND Malnutrition[tiab]) OR (Child*[ti] AND Undernutrition[ti]) OR (Child*[ti] AND Under-nutrition[ti]) OR (Child*[ti] AND Undernourish*[ti]) OR (Child*[ti] AND Overnutrition[ti]) OR (Child*[ti] AND Underweight[ti]) OR (Child*[ti] AND Leanness[ti]) OR (Child*[ti] AND Thinness[ti]) OR (Child*[ti] AND Slimness[ti]) OR (Child*[ti] AND Overweight[ti]) OR (Child*[ti] AND Obes*[ti]) OR (Child*[tiab] AND Height*[tiab]) OR (Child*[tiab] AND Weight*[tiab]) OR (Child*[tiab] AND Length[tiab]) OR Anthropometric*[ti] OR Length-for-age[tiab] OR Height-for-age[tiab] OR Weight-for-age[tiab] OR Weight-for-height[tiab] OR weight-for-length[tiab] OR "Body mass index-for-age"[tiab] OR BMI-for-age[tiab] OR (child*[ti] AND "failure to thrive"[ti]) OR (Infan*[tiab] AND Growth[tiab]) OR (Infan*[ti] AND "Growth Retardation"[ti]) OR (Infan*[ti] AND "Growth Disorder*"[ti]) OR (Infan*[ti] AND "Growth Falter*"[ti]) OR (Infan*[ti] AND "Growth Deficit*"[ti]) OR (Infan*[ti] AND "Growth failure"[ti]) OR (Infan*[ti] AND "Growth Trajectory"[ti]) OR (Infan*[ti] AND "Nutrition Disorder*"[ti]) OR (Infan*[tiab] AND	**20**	**2,949**	**2,949**	**2948**

	Malnutrition[tiab]) OR (Infan*[ti] AND Undernutrition[ti]) OR (Infan*[ti] AND Under-nutrition[ti]) OR (Infan*[ti] AND Undernourish*[ti]) OR (Infan*[ti] AND Overnutrition[ti]) OR (Infan*[ti] AND Underweight[ti]) OR (Infan*[ti] AND Leanness[ti]) OR (Infan*[ti] AND Thinness[ti]) OR (Infan*[ti] AND Slimness[ti]) OR (Infan*[ti] AND Overweight[ti]) OR (Infan*[ti] AND Obes*[ti]) OR (Infan*[tiab] AND Height*[tiab]) OR (Infan*[tiab] AND Weight*[tiab]) OR (Infan*[tiab] AND Length[tiab]) OR (infan*[ti] AND "failure to thrive"[ti]) OR (Under-five[tiab] AND Growth[tiab]) OR (Under-five[ti] AND "Growth Retardation"[ti]) OR (Under-five[ti] AND "Growth Disorder*"[ti]) OR (Under-five[ti] AND "Growth Falter*"[ti]) OR (Under-five[ti] AND "Growth Deficit*"[ti]) OR (Under-five[ti] AND "Growth failure"[ti]) OR (Under-five[ti] AND "Growth Trajectory"[ti]) OR (Under-five[ti] AND "Nutrition Disorder*"[ti]) OR (Under-five[tiab] AND Malnutrition[tiab]) OR (Under-five[ti] AND Undernutrition[ti]) OR (Under-five[ti] AND Under-nutrition[ti]) OR (Under-five[ti] AND Undernourish*[ti]) OR (Under-five[ti] AND Overnutrition[ti]) OR (Under-five[ti] AND Underweight[ti]) OR (Under-five[tiab] AND Leanness[ti]) OR (Under-five[ti] AND Thinness[ti]) OR (Under-five[ti] AND Slimness[ti]) OR (Under-five[ti] AND Overweight[ti]) OR (Under-five[ti] AND Obes*[ti]) OR (Under-five[tiab] AND Height*[tiab]) OR (Under-five[tiab] AND Weight*[tiab]) OR (Under-five[tiab] AND Length[tiab]) OR (under-five[ti] AND "failure to thrive"[ti]) OR ("under five"[tiab] AND Growth[tiab]) OR ("under five"[ti] AND "Growth Retardation"[ti]) OR ("under five"[ti] AND "Growth Disorder*"[ti]) OR ("under five"[ti] AND "Growth Falter*"[ti]) OR ("under five"[ti] AND "Growth Deficit*"[ti]) OR ("under five"[ti] AND "Growth failure"[ti]) OR ("under five"[ti] AND "Growth Trajectory"[ti]) OR ("under five"[ti] AND "Nutrition Disorder*"[ti]) OR ("under five"[tiab] AND Malnutrition[tiab]) OR ("under five"[ti] AND Undernutrition[ti]) OR ("under five"[ti] AND Under-nutrition[ti]) OR ("under five"[ti] AND Undernourish*[ti]) OR ("under five"[ti] AND Overnutrition[ti]) OR ("under five"[ti] AND Underweight[ti]) OR ("under five"[ti] AND Leanness[ti]) OR ("under five"[ti] AND Thinness[ti]) OR ("under five"[ti] AND Slimness[ti]) OR ("under five"[ti] AND Overweight[ti]) OR ("under five"[ti] AND Obes*[ti]) OR ("under five"[tiab] AND Height*[tiab]) OR ("under five"[tiab] AND Weight*[tiab]) OR ("under five"[tiab] AND Length[tiab]) OR ("under five"[ti] AND "failure to thrive"[ti]) OR ("under 5"[tiab] AND Growth[tiab]) OR ("under 5"[ti] AND "Growth Retardation"[ti]) OR ("under 5"[ti] AND "Growth Disorder*"[ti]) OR ("under 5"[ti] AND "Growth Falter*"[ti]) OR ("under 5"[ti] AND "Growth Deficit*"[ti]) OR ("under 5"[ti] AND "Growth failure"[ti])OR ("under 5"[ti] AND "Growth Trajectory"[ti]) OR ("under 5"[ti] AND "Nutrition Disorder*"[ti]) OR ("under 5"[tiab] AND Malnutrition[tiab]) OR ("under 5"[ti] AND Undernutrition[ti]) OR ("under 5"[ti] AND Under-nutrition[ti]) OR ("under 5"[ti] AND Undernourish*[ti]) OR ("under 5"[ti] AND Overnutrition[ti]) OR ("under 5"[ti] AND Underweight[ti]) OR ("under 5"[ti] AND Leanness[ti]) OR ("under 5"[ti] AND Thinness[ti]) OR ("under 5"[ti] AND Slimness[ti]) OR ("under 5"[ti] AND Overweight[ti]) OR ("under 5"[ti] AND Obes*[ti]) OR ("under 5"[tiab] AND Height*[tiab]) OR ("under 5"[tiab] AND Weight*[tiab]) OR ("under 5"[tiab] AND Length[tiab]) OR ("under 5"[ti] AND "failure to thrive"[ti]) OR (Under-5[tiab] AND Growth[tiab]) OR (Under-5[ti] AND "Growth Retardation"[ti]) OR (Under-5[ti] AND "Growth Disorder*"[ti]) OR (Under-5[ti] AND "Growth Falter*"[ti]) OR (Under-5[ti] AND "Growth Deficit*"[ti]) OR (Under-5[ti] AND "Growth failure"[ti]) OR (Under-5[ti] AND "Growth Trajectory"[ti]) OR (Under-5[ti] AND "Nutrition Disorder*"[ti]) OR (Under-5[tiab] AND Malnutrition[tiab]) OR (Under-5[ti] AND Undernutrition[ti]) OR (Under-5[ti] AND Under-nutrition[ti]) OR (Under-5[ti] AND Undernourish*[ti]) OR (Under-5[ti] AND Overnutrition[ti]) OR (Under-5[ti] AND Underweight[ti]) OR (Under-5[ti] AND Leanness[ti]) OR (Under-5[ti] AND Thinness[ti]) OR (Under-5[ti] AND Slimness[ti]) OR (Under-5[ti] AND Overweight[ti]) OR (Under-5[ti] AND Obes*[ti]) OR (Under-5[tiab] AND Height*[tiab]) OR (Under-5[tiab] AND Weight*[tiab]) OR (Under-5[tiab] AND Length[tiab]) OR (Under-5[ti] AND "failure to thrive"[ti]) OR (Under-two[tiab] AND Growth[tiab]) OR (Under-two[ti] AND "Growth Retardation"[ti]) OR (Under-two[ti] AND "Growth Disorder*"[ti]) OR (Under-two[ti] AND "Growth Falter*"[ti]) OR (Under-two[ti] AND "Growth Deficit*"[ti]) OR (Under-two[ti] AND "Growth failure"[ti]) OR (Under-two[ti] AND "Growth Trajectory"[ti]) OR (Under-two[ti] AND "Nutrition Disorder*"[ti]) OR (Under-two[tiab] AND Malnutrition[tiab]) OR (Under-two[ti] AND Undernutrition[ti]) OR (Under-two[ti] AND Under-nutrition[ti]) OR (Under-two[ti] AND Undernourish*[ti]) OR (Under-two[ti] AND Overnutrition[ti]) OR (Under-two[ti] AND Underweight[ti]) OR (Under-two[ti] AND Leanness[ti]) OR (Under-two[ti] AND Thinness[ti]) OR (Under-two[ti] AND Slimness[ti]) OR (Under-two[tiab] AND Overweight[tiab]) OR (Under-two[ti] AND Obes*[ti]) OR (Under-two[tiab] AND Height*[tiab]) OR (Under-two[tiab] AND Weight*[tiab]) OR (Under-two[tiab] AND Length[tiab]) OR (Under-two[ti] AND "failure to thrive"[ti]) OR ("under two"[tiab] AND Growth[tiab]) OR ("under two"[ti] AND "Growth Retardation"[ti]) OR ("under two"[ti] AND "Growth Disorder*"[ti]) OR ("under two"[ti] AND "Growth Falter*"[ti]) OR ("under two"[ti] AND "Growth Deficit*"[ti]) OR ("under two"[ti] AND "Growth failure"[ti]) OR ("under two"[ti] AND "Growth Trajectory"[ti]) OR ("under two"[ti] AND "Nutrition Disorder*"[ti]) OR ("under two"[tiab] AND Malnutrition[tiab]) OR ("under two"[ti] AND Undernutrition[ti]) OR ("under two"[ti] AND Under-nutrition[ti]) OR ("under two"[ti] AND Undernourish*[ti]) OR ("under two"[ti] AND Overnutrition[ti]) OR ("under two"[ti] AND Underweight[ti]) OR ("under two"[ti] AND Leanness[ti]) OR ("under two"[ti] AND Thinness[ti]) OR ("under two"[ti] AND Slimness[ti]) OR ("under two"[ti] AND Overweight[ti]) OR ("under two"[ti] AND Obes*[ti]) OR ("under two"[tiab] AND Height*[tiab]) OR ("under two"[tiab] AND Weight*[tiab]) OR ("under two"[tiab] AND Length[tiab]) OR ("under two"[ti] AND "failure to thrive"[ti]) OR ("under 2"[tiab] AND Growth[tiab]) OR ("under 2"[ti] AND "Growth Retardation"[ti]) OR ("under 2"[ti] AND "Growth Disorder*"[ti]) OR ("under 2"[ti] AND "Growth Falter*"[ti]) OR ("under 2"[ti] AND "Growth Deficit*"[ti]) OR ("under 2"[ti] AND "Growth failure"[ti]) OR ("under 2"[ti] AND "Growth				

	Trajectory”[ti]) OR (“under 2”[ti] AND “Nutrition Disorder*”[ti]) OR (“under 2”[tiab] AND Malnutrition[tiab]) OR (“under 2”[ti] AND Undernutrition[ti]) OR (“under 2”[ti] AND Under-nutrition[ti]) OR (“under 2”[ti] AND Undernourish*[ti]) OR (“under 2”[ti] AND Overnutrition[ti]) OR (“under 2”[ti] AND Underweight[ti]) OR (“under 2”[ti] AND Leanness[ti]) OR (“under 2”[ti] AND Thinness[ti]) OR (“under 2”[ti] AND Slimness[ti]) OR (“under 2”[ti] AND Overweight[ti]) OR (“under 2”[ti] AND Obes*[ti]) OR (“under 2”[tiab] AND Height*[tiab]) OR (“under 2”[tiab] AND Weight*[tiab]) OR (“under 2”[tiab] AND Length[tiab]) OR (“under 2”[ti] AND “failure to thrive”[ti]) OR (Under-2[tiab] AND Growth[tiab]) OR (Under-2[ti] AND “Growth Retardation”[ti]) OR (Under-2[ti] AND “Growth Disorder*”[ti]) OR (Under-2[ti] AND “Growth Falter*”[ti]) OR (Under-2[ti] AND “Growth Deficit*”[ti]) OR (Under-2[ti] AND “Growth failure”[ti]) OR (Under-2[ti] AND “Growth Trajectory”[ti]) OR (Under-2[ti] AND “Nutrition Disorder*”[ti]) OR (Under-2[tiab] AND Malnutrition[tiab]) OR (Under-2[ti] AND Undernutrition[ti]) OR (Under-2[ti] AND Under-nutrition[ti]) OR (Under-2[ti] AND Undernourish*[ti]) OR (Under-2[ti] AND Overnutrition[ti]) OR (Under-2[ti] AND Underweight[ti]) OR (Under-2[ti] AND Leanness[ti]) OR (Under-2[ti] AND Thinness[ti]) OR (Under-2[ti] AND Slimness[ti]) OR (Under-2[ti] AND Overweight[ti]) OR (Under-2[ti] AND Obes*[ti]) OR (Under-2[tiab] AND Height*[tiab]) OR (Under-2[tiab] AND Weight*[tiab]) OR (Under-2[tiab] AND Length[tiab]) OR (Under-2[ti] AND “failure to thrive”[ti]) OR (Offspring[tiab] AND Growth[tiab]) OR (Offspring[ti] AND “Growth Retardation”[ti]) OR (Offspring[ti] AND “Growth Disorder*”[ti]) OR (Offspring[ti] AND “Growth Falter*”[ti]) OR (Offspring[ti] AND “Growth Deficit*”[ti]) OR (Offspring[ti] AND “Growth failure”[ti]) OR (Offspring[ti] AND “Growth Trajectory”[ti]) OR (Offspring[ti] AND “Nutrition Disorder*”[ti]) OR (Offspring[tiab] AND Malnutrition[tiab]) OR (Offspring[ti] AND Undernutrition[ti]) OR (Offspring[ti] AND Under-nutrition[ti]) OR (Offspring[ti] AND Undernourish*[ti]) OR (Offspring[ti] AND Overnutrition[ti]) OR (Offspring[ti] AND Underweight[ti]) OR (Offspring[ti] AND Leanness[ti]) OR (Offspring[ti] AND Thinness[ti]) OR (Offspring[ti] AND Slimness[ti]) OR (Offspring[ti] AND Overweight[ti]) OR (Offspring[ti] AND Obes*[ti]) OR (Offspring[tiab] AND Height*[tiab]) OR (Offspring[tiab] AND Weight*[tiab]) OR (Offspring[tiab] AND Length[tiab]) OR (Offspring[ti] AND “failure to thrive”[ti]) OR (Early-life[tiab] AND Growth[tiab]) OR (Early-life[ti] AND “Growth Retardation”[ti]) OR (Early-life[ti] AND “Growth Disorder*”[ti]) OR (Early-life[ti] AND “Growth Falter*”[ti]) OR (Early-life[ti] AND “Growth Deficit*”[ti]) OR (Early-life[ti] AND “Growth failure”[ti]) OR (Early-life[ti] AND “Growth Trajectory”[ti]) OR (Early-life[ti] AND “Nutrition Disorder*”[ti]) OR (Early-life[tiab] AND Malnutrition[tiab]) OR (Early-life[ti] AND Undernutrition[ti]) OR (Early-life[ti] AND Under-nutrition[ti]) OR (Early-life[ti] AND Undernourish*[ti]) OR (Early-life[ti] AND Overnutrition[ti]) OR (Early-life[ti] AND Underweight[ti]) OR (Early-life[ti] AND Leanness[ti]) OR (Early-life[ti] AND Thinness[ti]) OR (Early-life[ti] AND Slimness[ti]) OR (Early-life[ti] AND Overweight[ti]) OR (Early-life[ti] AND Obes*[ti]) OR (Early-life[tiab] AND Height*[tiab]) OR (Early-life[tiab] AND Weight*[tiab]) OR (Early-life[tiab] AND Length[tiab]) OR (Early-life[ti] AND “failure to thrive”[ti]) OR (“Early life”[tiab] AND Growth[tiab]) OR (“Early life”[ti] AND “Growth Retardation”[ti]) OR (“Early life”[ti] AND “Growth Disorder*”[ti]) OR (“Early life”[ti] AND “Growth Falter*”[ti]) OR (“Early life”[ti] AND “Growth Deficit*”[ti]) OR (“Early life”[ti] AND “Growth failure”[ti]) OR (“Early life”[ti] AND “Growth Trajectory”[ti]) OR (“Early life”[ti] AND “Nutrition Disorder*”[ti]) OR (“Early life”[tiab] AND Malnutrition[tiab]) OR (“Early life”[ti] AND Undernutrition[ti]) OR (“Early life”[ti] AND Under-nutrition[ti]) OR (“Early life”[ti] AND Undernourish*[ti]) OR (“Early life”[ti] AND Overnutrition[ti]) OR (“Early life”[ti] AND Underweight[ti]) OR (“Early life”[ti] AND Leanness[ti]) OR (“Early life”[ti] AND Thinness[ti]) OR (“Early life”[ti] AND Slimness[ti]) OR (“Early life”[ti] AND Overweight[ti]) OR (“Early life”[ti] AND Obes*[ti]) OR (“Early life”[tiab] AND Height*[tiab]) OR (“Early life”[tiab] AND Weight*[tiab]) OR (“Early life”[tiab] AND Length[tiab]) OR (“Early life”[ti] AND “failure to thrive”[ti]) OR (Pediatric[tiab] AND Growth[tiab]) OR (Pediatric[ti] AND “Growth Retardation”[ti]) OR (Pediatric[ti] AND “Growth Disorder*”[ti]) OR (Pediatric[ti] AND “Growth Falter*”[ti]) OR (Pediatric[ti] AND “Growth Deficit*”[ti]) OR (Pediatric[ti] AND “Growth failure”[ti]) OR (Pediatric[ti] AND “Growth Trajectory”[ti]) OR (Pediatric[ti] AND “Nutrition Disorder*”[ti]) OR (Pediatric[tiab] AND Malnutrition[tiab]) OR (Pediatric[ti] AND Undernutrition[ti]) OR (Pediatric[ti] AND Under-nutrition[ti]) OR (Pediatric[ti] AND Undernourish*[ti]) OR (Pediatric[ti] AND Overnutrition[ti]) OR (Pediatric[ti] AND Underweight[ti]) OR (Pediatric[ti] AND Leanness[ti]) OR (Pediatric[ti] AND Thinness[ti]) OR (Pediatric[ti] AND Slimness[ti]) OR (Pediatric[ti] AND Overweight[ti]) OR (Pediatric[ti] AND Obes*[ti]) OR (Pediatric[tiab] AND Height*[tiab]) OR (Pediatric[tiab] AND Weight*[tiab]) OR (Pediatric[tiab] AND Length[tiab]) OR (Pediatric[ti] AND “failure to thrive”[ti]) OR “child health”[ti])<br>AND<br>(cohort[tiab] OR longitudinal[tiab] OR (Cohort[tiab] AND Study[tiab]) OR (Concurrent[tiab] AND Study[tiab]) OR “birth cohort”[tiab] OR (cohort[tiab] AND analysis[tiab]) OR (cohort[tiab] AND analyses[tiab]) OR “incidence study”[tiab] OR “Follow Up Study”[tiab] OR “Follow-Up Study”[tiab] OR “Followup Study”[tiab] OR (Longitudinal[tiab] AND Study[tiab]) OR (Prospective[tiab] AND study[tiab]))<br>AND<br>(1990/1/01:2024/1/31[dp]))				

➢ Scopus Syntax

SCOPUS Syntax	*Coference paper*	*Original Article*	*Output No.*	*Output No. after importing to mendeley*	*Output No. after removing duplicates*
(TITLE(("Educational Status" AND Maternal)) OR TITLE((Status AND "Maternal Educational")) OR TITLE("Maternal Educational Status") OR TITLE((Maternal AND education)) OR TITLE((Maternal AND Illiteracy)) OR TITLE((Maternal AND Literacy)) OR ALL((maternal AND "schooling")) OR TITLE-ABS((mother AND education)) OR TITLE((mother* AND Illiteracy)) OR TITLE((mother* AND Literacy)) OR ALL((mother* AND "schooling")) OR TITLE((parent* AND Illiteracy)) OR TITLE((parent* AND Literacy)) OR TITLE((parent* AND education)) OR TITLE((parent* AND "schooling")) OR TITLE((parent* AND socio-economic)) OR TITLE((parent* AND socioeconomic)) OR TITLE((parent* AND "Social Class*")) OR TITLE((parent* AND Socio-demographic)) OR TITLE((parent* AND Sociodemographic)) OR TITLE((parent* AND Socio-cultural)) OR TITLE((parent* AND Sociocultural)) OR TITLE-ABS((mother* AND socio-economic)) OR TITLE-ABS((mother* AND socioeconomic)) OR TITLE((mother* AND "Social Class*")) OR TITLE-ABS((mother* AND Socio-demographic)) OR TITLE-ABS((mother* AND Sociodemographic)) OR TITLE((mother* AND Socio-cultural)) OR TITLE((mother* AND Sociocultural)) OR TITLE-ABS((maternal AND socio-economic)) OR TITLE-ABS((maternal AND socioeconomic)) OR TITLE((maternal AND "Social Class*")) OR TITLE-ABS((maternal AND Socio-demographic)) OR TITLE-ABS((maternal AND Sociodemographic)) OR TITLE((maternal AND Socio-cultural)) OR TITLE((maternal AND Sociocultural)) OR TITLE("maternal socioeconomic status") OR TITLE("parental socioeconomic status")) AND (TITLE-ABS((Child* AND Growth)) OR TITLE((Child* AND "Growth Retardation")) OR TITLE((Child* AND "Growth Disorder*")) OR TITLE((Child* AND "Growth Falter*")) OR TITLE((Child* AND "Growth Deficit*")) OR TITLE((Child* AND "Growth failure")) OR TITLE((Child* AND "Growth Trajectory")) OR TITLE(Stunting*) OR TITLE("Stunted Growth") OR TITLE((Growth AND Stunted)) OR TITLE("short stature") OR TITLE-ABS(wast*) OR TITLE((Child* AND "Nutrition Disorder*")) OR TITLE-ABS((Child* AND Malnutrition)) OR TITLE((Child* AND Undernutrition)) OR TITLE((Child* AND Under-nutrition)) OR TITLE((Child* AND Undernourish*)) OR TITLE((Child* AND Overnutrition)) OR TITLE((Child* AND Underweight)) OR TITLE((Child* AND Leanness)) OR TITLE((Child* AND Thinness)) OR TITLE((Child* AND Slimness)) OR TITLE((Child* AND Overweight)) OR TITLE((Child* AND Obes*)) OR TITLE-ABS((Child* AND Height*)) OR TITLE-ABS((Child* AND Weight*)) OR TITLE-ABS((Child* AND Length)) OR TITLE(Anthropometric*) OR TITLE-ABS(Length-for-age) OR TITLE-ABS(Height-for-age) OR TITLE-ABS(Weight-for-age) OR TITLE-ABS(Weight-for-height) OR TITLE-ABS(weight-for-length) OR TITLE-ABS("Body mass index-for-age") OR TITLE-ABS(BMI-for-age) OR TITLE((child* AND "failure to thrive")) OR TITLE-ABS((Infan* AND Growth)) OR TITLE((Infan* AND "Growth Retardation")) OR TITLE((Infan* AND "Growth Disorder*")) OR TITLE((Infan* AND "Growth Falter*")) OR TITLE((Infan* AND "Growth Deficit*")) OR TITLE((Infan* AND "Growth failure")) OR TITLE((Infan* AND "Growth Trajectory")) OR TITLE((Infan* AND "Nutrition Disorder*")) OR TITLE-ABS((Infan* AND Malnutrition)) OR TITLE((Infan* AND Undernutrition)) OR TITLE((Infan* AND Under-nutrition)) OR TITLE((Infan* AND Undernourish*)) OR TITLE((Infan* AND Overnutrition)) OR TITLE((Infan* AND Underweight)) OR TITLE((Infan* AND Leanness)) OR TITLE((Infan* AND Thinness)) OR TITLE((Infan* AND Slimness)) OR TITLE((Infan* AND Overweight)) OR TITLE((Infan* AND Obes*)) OR TITLE-ABS((Infan* AND Height*)) OR TITLE-ABS((Infan* AND Weight*)) OR TITLE-ABS((Infan* AND Length)) OR TITLE((infan* AND "failure to thrive")) OR TITLE-ABS((Under-five AND Growth)) OR TITLE((Under-five AND "Growth Retardation")) OR TITLE((Under-five AND "Growth Disorder*")) OR TITLE((Under-five AND "Growth Falter*")) OR TITLE((Under-five AND "Growth Deficit*")) OR TITLE((Under-five AND "Growth failure")) OR TITLE((Under-five AND "Growth Trajectory")) OR TITLE((Under-five AND "Nutrition Disorder*")) OR TITLE-ABS((Under-five AND Malnutrition)) OR TITLE((Under-five AND Undernutrition)) OR TITLE((Under-five AND Under-nutrition)) OR TITLE((Under-five AND Undernourish*)) OR TITLE((Under-five AND Overnutrition)) OR TITLE((Under-five AND Underweight)) OR TITLE-ABS((Under-five AND Leanness)) OR TITLE((Under-five AND Thinness)) OR TITLE((Under-five AND Slimness)) OR TITLE((Under-five AND Overweight)) OR TITLE((Under-five AND Obes*)) OR TITLE-ABS((Under-five AND Height*)) OR TITLE-ABS((Under-five AND Weight*)) OR TITLE-ABS((Under-five AND Length)) OR TITLE((under-five AND "failure to thrive")) OR TITLE-ABS(("under five" AND Growth)) OR TITLE(("under five" AND "Growth Retardation")) OR TITLE(("under five" AND "Growth Disorder*")) OR TITLE(("under five" AND "Growth Falter*")) OR TITLE(("under five" AND "Growth Deficit*")) OR TITLE(("under five" AND "Growth failure")) OR TITLE(("under five" AND "Growth Trajectory")) OR TITLE(("under five" AND "Nutrition Disorder*")) OR TITLE-ABS(("under five" AND Malnutrition)) OR TITLE(("under five" AND Undernutrition)) OR TITLE(("under five" AND Under-nutrition)) OR TITLE(("under five" AND Undernourish*)) OR TITLE(("under five" AND Overnutrition)) OR TITLE(("under five" AND Underweight)) OR TITLE(("under five"*	**25**	**3686**	**3,849**	**3841**	**3838**

AND Leanness)) OR TITLE(("under five" AND Thinness)) OR TITLE(("under five" AND Slimness)) OR TITLE(("under five" AND Overweight)) OR TITLE(("under five" AND Obes)) OR TITLE-ABS(("under five" AND Height*)) OR TITLE-ABS(("under five" AND Weight*)) OR TITLE-ABS(("under five" AND Length)) OR TITLE(("under five" AND "failure to thrive")) OR TITLE-ABS(("under 5" AND Growth)) OR TITLE(("under 5" AND "Growth Retardation")) OR TITLE(("under 5" AND "Growth Disorder*")) OR TITLE(("under 5" AND "Growth Falter*")) OR TITLE(("under 5" AND "Growth Deficit*")) OR TITLE(("under 5" AND "Growth failure"))OR TITLE(("under 5" AND "Growth Trajectory")) OR TITLE(("under 5" AND "Nutrition Disorder*")) OR TITLE-ABS(("under 5" AND Malnutrition)) OR TITLE(("under 5" AND Undernutrition)) OR TITLE(("under 5" AND Under-nutrition)) OR TITLE(("under 5" AND Undernourish*)) OR TITLE(("under 5" AND Overnutrition)) OR TITLE(("under 5" AND Underweight)) OR TITLE(("under 5" AND Leanness)) OR TITLE(("under 5" AND Thinness)) OR TITLE(("under 5" AND Slimness)) OR TITLE(("under 5" AND Overweight)) OR TITLE(("under 5" AND Obes*)) OR TITLE-ABS(("under 5" AND Height*)) OR TITLE-ABS(("under 5" AND Weight*)) OR TITLE-ABS(("under 5" AND Length)) OR TITLE(("under 5" AND "failure to thrive")) OR TITLE-ABS((Under-5 AND Growth)) OR TITLE((Under-5 AND "Growth Retardation")) OR TITLE((Under-5 AND "Growth Disorder*")) OR TITLE((Under-5 AND "Growth Falter*")) OR TITLE((Under-5 AND "Growth Deficit*")) OR TITLE((Under-5 AND "Growth failure")) OR TITLE((Under-5 AND "Growth Trajectory")) OR TITLE((Under-5 AND "Nutrition Disorder*")) OR TITLE-ABS((Under-5 AND Malnutrition)) OR TITLE((Under-5 AND Undernutrition)) OR TITLE((Under-5 AND Under-nutrition)) OR TITLE((Under-5 AND Undernourish*)) OR TITLE((Under-5 AND Overnutrition)) OR TITLE((Under-5 AND Underweight)) OR TITLE((Under-5 AND Leanness)) OR TITLE((Under-5 AND Thinness)) OR TITLE((Under-5 AND Slimness)) OR TITLE((Under-5 AND Overweight)) OR TITLE((Under-5 AND Obes*)) OR TITLE-ABS((Under-5 AND Height*)) OR TITLE-ABS((Under-5 AND Weight*)) OR TITLE-ABS((Under-5 AND Length)) OR TITLE((Under-5 AND "failure to thrive")) OR TITLE-ABS((Under-two AND Growth)) OR TITLE((Under-two AND "Growth Retardation")) OR TITLE((Under-two AND "Growth Disorder*")) OR TITLE((Under-two AND "Growth Falter*")) OR TITLE((Under-two AND "Growth Deficit*")) OR TITLE((Under-two AND "Growth failure")) OR TITLE((Under-two AND "Growth Trajectory")) OR TITLE((Under-two AND "Nutrition Disorder*")) OR TITLE-ABS((Under-two AND Malnutrition)) OR TITLE((Under-two AND Undernutrition)) OR TITLE((Under-two AND Under-nutrition)) OR TITLE((Under-two AND Undernourish*)) OR TITLE((Under-two AND Overnutrition)) OR TITLE((Under-two AND Underweight)) OR TITLE((Under-two AND Leanness)) OR TITLE((Under-two AND Thinness)) OR TITLE((Under-two AND Slimness)) OR TITLE-ABS((Under-two AND Overweight)) OR TITLE((Under-two AND Obes*)) OR TITLE-ABS((Under-two AND Height*)) OR TITLE-ABS((Under-two AND Weight*)) OR TITLE-ABS((Under-two AND Length)) OR TITLE((Under-two AND "failure to thrive")) OR TITLE-ABS(("under two" AND Growth)) OR TITLE(("under two" AND "Growth Retardation")) OR TITLE(("under two" AND "Growth Disorder*")) OR TITLE(("under two" AND "Growth Falter*")) OR TITLE(("under two" AND "Growth Deficit*")) OR TITLE(("under two" AND "Growth failure")) OR TITLE(("under two" AND "Growth Trajectory")) OR TITLE(("under two" AND "Nutrition Disorder*")) OR TITLE-ABS(("under two" AND Malnutrition)) OR TITLE(("under two" AND Undernutrition)) OR TITLE(("under two" AND Under-nutrition)) OR TITLE(("under two" AND Undernourish*)) OR TITLE(("under two" AND Overnutrition)) OR TITLE(("under two" AND Underweight)) OR TITLE(("under two" AND Leanness)) OR TITLE(("under two" AND Thinness)) OR TITLE(("under two" AND Slimness)) OR TITLE(("under two" AND Overweight)) OR TITLE(("under two" AND Obes*)) OR TITLE-ABS(("under two" AND Height*)) OR TITLE-ABS(("under two" AND Weight*)) OR TITLE-ABS(("under two" AND Length)) OR TITLE(("under two" AND "failure to thrive")) OR TITLE-ABS(("under 2" AND Growth)) OR TITLE(("under 2" AND "Growth Retardation")) OR TITLE(("under 2" AND "Growth Disorder*")) OR TITLE(("under 2" AND "Growth Falter*")) OR TITLE(("under 2" AND "Growth Deficit*")) OR TITLE(("under 2" AND "Growth failure")) OR TITLE(("under 2" AND "Growth Trajectory")) OR TITLE(("under 2" AND "Nutrition Disorder*")) OR TITLE-ABS(("under 2" AND Malnutrition)) OR TITLE(("under 2" AND Undernutrition)) OR TITLE(("under 2" AND Under-nutrition)) OR TITLE(("under 2"AND Undernourish*)) OR TITLE(("under 2" AND Overnutrition)) OR TITLE(("under 2" AND Underweight)) OR TITLE(("under 2" AND Leanness)) OR TITLE(("under 2" AND Thinness)) OR TITLE(("under 2" AND Slimness)) OR TITLE(("under 2" AND Overweight)) OR TITLE(("under 2" AND Obes*)) OR TITLE-ABS(("under 2" AND Height*)) OR TITLE-ABS(("under 2" AND Weight*)) OR TITLE-ABS(("under 2" AND Length)) OR TITLE(("under 2" AND "failure to thrive")) OR TITLE-ABS((Under-2 AND Growth)) OR TITLE((Under-2 AND "Growth Retardation")) OR TITLE((Under-2 AND "Growth Disorder*")) OR TITLE((Under-2 AND "Growth Falter*")) OR TITLE((Under-2 AND "Growth Deficit*")) OR TITLE((Under-2 AND "Growth failure")) OR TITLE((Under-2 AND "Growth Trajectory")) OR TITLE((Under-2 AND "Nutrition Disorder*")) OR TITLE-ABS((Under-2 AND Malnutrition)) OR TITLE((Under-2 AND Undernutrition)) OR TITLE((Under-2 AND Under-nutrition)) OR TITLE((Under-2 AND Undernourish*)) OR TITLE((Under-2 AND Overnutrition)) OR TITLE((Under-2 AND Underweight)) OR TITLE((Under-2 AND Leanness)) OR TITLE((Under-2 AND Thinness)) OR TITLE((Under-2 AND Slimness)) OR TITLE((Under-2 AND Overweight)) OR TITLE((Under-2 AND Obes*)) OR TITLE-ABS((Under-2 AND Height*)) OR TITLE-ABS((Under-2 AND Weight*)) OR TITLE-ABS((Under-2 AND Length)) OR TITLE((Under-2 AND "failure to thrive")) OR TITLE-ABS((Offspring AND Growth)) OR TITLE((Offspring AND "Growth Retardation")) OR TITLE((Offspring AND*					

"Growth Disorder")) OR TITLE((Offspring AND "Growth Falter*")) OR TITLE((Offspring AND "Growth Deficit*")) OR TITLE((Offspring AND "Growth failure")) OR TITLE((Offspring AND "Growth Trajectory")) OR TITLE((Offspring AND "Nutrition Disorder*")) OR TITLE-ABS((Offspring AND Malnutrition)) OR TITLE((Offspring AND Undernutrition)) OR TITLE((Offspring AND Under-nutrition)) OR TITLE((Offspring AND Undernourish*)) OR TITLE((Offspring AND Overnutrition)) OR TITLE((Offspring AND Underweight)) OR TITLE((Offspring AND Leanness)) OR TITLE((Offspring AND Thinness)) OR TITLE((Offspring AND Slimness)) OR TITLE((Offspring AND Overweight)) OR TITLE((Offspring AND Obes*)) OR TITLE-ABS((Offspring AND Height*)) OR TITLE-ABS((Offspring AND Weight*)) OR TITLE-ABS((Offspring AND Length)) OR TITLE((Offspring AND "failure to thrive")) OR TITLE-ABS((Early-life AND Growth)) OR TITLE((Early-life AND "Growth Retardation")) OR TITLE((Early-life AND "Growth Disorder*")) OR TITLE((Early-life AND "Growth Falter*")) OR TITLE((Early-life AND "Growth Deficit*")) OR TITLE((Early-life AND "Growth failure")) OR TITLE((Early-life AND "Growth Trajectory")) OR TITLE((Early-life AND "Nutrition Disorder*")) OR TITLE-ABS((Early-life AND Malnutrition)) OR TITLE((Early-life AND Undernutrition)) OR TITLE((Early-life AND Under-nutrition)) OR TITLE((Early-life AND Undernourish*)) OR TITLE((Early-life AND Overnutrition)) OR TITLE((Early-life AND Underweight)) OR TITLE((Early-life AND Leanness)) OR TITLE((Early-life AND Thinness)) OR TITLE((Early-life AND Slimness)) OR TITLE((Early-life AND Overweight)) OR TITLE((Early-life AND Obes*)) OR TITLE-ABS((Early-life AND Height*)) OR TITLE-ABS((Early-life AND Weight*)) OR TITLE-ABS((Early-life AND Length)) OR TITLE((Early-life AND "failure to thrive")) OR TITLE-ABS(("Early life" AND Growth)) OR TITLE(("Early life" AND "Growth Retardation")) OR TITLE(("Early life" AND "Growth Disorder*")) OR TITLE(("Early life" AND "Growth Falter*")) OR TITLE(("Early life" AND "Growth Deficit*")) OR TITLE(("Early life" AND "Growth failure")) OR TITLE(("Early life" AND "Growth Trajectory")) OR TITLE(("Early life" AND "Nutrition Disorder*")) OR TITLE-ABS(("Early life" AND Malnutrition)) OR TITLE(("Early life" AND Undernutrition)) OR TITLE(("Early life" AND Under-nutrition)) OR TITLE(("Early life" AND Undernourish*)) OR TITLE(("Early life" AND Overnutrition)) OR TITLE(("Early life" AND Underweight)) OR TITLE(("Early life" AND Leanness)) OR TITLE(("Early life" AND Thinness)) OR TITLE(("Early life" AND Slimness)) OR TITLE(("Early life" AND Overweight)) OR TITLE(("Early life" AND Obes*)) OR TITLE-ABS(("Early life" AND Height*)) OR TITLE-ABS(("Early life" AND Weight*)) OR TITLE-ABS(("Early life" AND Length)) OR TITLE(("Early life" AND "failure to thrive")) OR TITLE-ABS((Pediatric AND Growth)) OR TITLE((Pediatric AND "Growth Retardation")) OR TITLE((Pediatric AND "Growth Disorder*")) OR TITLE((Pediatric AND "Growth Falter*")) OR TITLE((Pediatric AND "Growth Deficit*")) OR TITLE((Pediatric AND "Growth failure")) OR TITLE((Pediatric AND "Growth Trajectory")) OR TITLE((Pediatric AND "Nutrition Disorder*")) OR TITLE-ABS((Pediatric AND Malnutrition)) OR TITLE((Pediatric AND Undernutrition)) OR TITLE((Pediatric AND Under-nutrition)) OR TITLE((Pediatric AND Undernourish*)) OR TITLE((Pediatric AND Overnutrition)) OR TITLE((Pediatric AND Underweight)) OR TITLE((Pediatric AND Leanness)) OR TITLE((Pediatric AND Thinness)) OR TITLE((Pediatric AND Slimness)) OR TITLE((Pediatric AND Overweight)) OR TITLE((Pediatric AND Obes*)) OR TITLE-ABS((Pediatric AND Height*)) OR TITLE-ABS((Pediatric AND Weight*)) OR TITLE-ABS((Pediatric AND Length)) OR TITLE((Pediatric AND "failure to thrive")) OR TITLE("child health")) AND (TITLE-ABS(cohort) OR TITLE-ABS(longitudinal) OR TITLE-ABS((Cohort AND Study)) OR TITLE-ABS((Concurrent AND Study)) OR TITLE-ABS("birth cohort") OR TITLE-ABS((cohort AND analysis)) OR TITLE-ABS((cohort AND analyses)) OR TITLE-ABS("incidence study") OR TITLE-ABS("Follow Up Study") OR TITLE-ABS("Follow-Up Study") OR TITLE-ABS("Followup Study") OR TITLE-ABS((Longitudinal AND Study)) OR TITLE-ABS((Prospective AND study))) AND ((PUBYEAR > 1989 AND PUBYEAR < 2024) OR PUBDATETXT(January 2024))*					

➢ Web Of Sciences Syntax

WOS Syntax	*Output No.*	*Output No. after importing to mendeley*	*Output No. after removing duplicates*
((TI=(("Educational Status" AND Maternal)) OR TI=((Status AND "Maternal Educational")) OR TI=("Maternal Educational Status") OR TI=((Maternal AND education)) OR TI=((Maternal AND Illiteracy)) OR TI=((Maternal AND Literacy)) OR ALL=((maternal AND "schooling")) OR TS=((mother AND education)) OR TI=((mother* AND Illiteracy)) OR TI=((mother* AND Literacy)) OR ALL=((mother* AND "schooling")) OR TI=((parent* AND Illiteracy)) OR TI=((parent* AND Literacy)) OR TI=((parent* AND education)) OR TI=((parent* AND "schooling")) OR TI=((parent* AND socio-economic)) OR TI=((parent* AND socioeconomic)) OR TI=((parent* AND "Social Class*")) OR TI=((parent* AND Socio-demographic)) OR TI=((parent* AND Sociodemographic)) OR TI=((parent* AND Socio-cultural)) OR TI=((parent* AND Sociocultural)) OR TS=((mother* AND socio-economic)) OR TS=((mother* AND socioeconomic)) OR TI=((mother* AND "Social Class*")) OR TS=((mother* AND Socio-demographic)) OR TS=((mother* AND Sociodemographic)) OR TI=((mother* AND Socio-cultural)) OR TI=((mother* AND Sociocultural)) OR TS=((maternal AND socio-economic)) OR TS=((maternal AND socioeconomic)) OR TI=((maternal AND "Social Class*")) OR TS=((maternal AND Socio-demographic)) OR TS=((maternal AND Sociodemographic)) OR TI=((maternal AND Socio-cultural)) OR TI=((maternal AND Sociocultural)) OR TI=("maternal socioeconomic status") OR TI=("parental socioeconomic status")) AND (TS=((Child* AND Growth)) OR TI=((Child* AND "Growth Retardation")) OR TI=((Child* AND "Growth Disorder*")) OR TI=((Child* AND "Growth Falter*")) OR TI=((Child* AND "Growth Deficit*")) OR TI=((Child* AND "Growth failure")) OR TI=((Child* AND "Growth Trajectory")) OR TI=(Stunting*) OR TI=("Stunted Growth") OR TI=((Growth AND Stunted)) OR TI=("short stature") OR TS=(wast*) OR TI=((Child* AND "Nutrition Disorder*")) OR TS=((Child* AND Malnutrition)) OR TI=((Child* AND Undernutrition)) OR TI=((Child* AND Under-nutrition)) OR TI=((Child* AND Undernourish*)) OR TI=((Child* AND Overnutrition)) OR TI=((Child* AND Underweight)) OR TI=((Child* AND Leanness)) OR TI=((Child* AND Thinness)) OR TI=((Child* AND Slimness)) OR TI=((Child* AND Overweight)) OR TI=((Child* AND Obes*)) OR TS=((Child* AND Height*)) OR TS=((Child* AND Weight*)) OR TS=((Child* AND Length)) OR TI=(Anthropometric*) OR TS=(Length-for-age) OR TS=(Height-for-age) OR TS=(Weight-for-age) OR TS=(Weight-for-height) OR TS=(weight-for-length) OR TS=("Body mass index-for-age") OR TS=(BMI-for-age) OR TI=((child* AND "failure to thrive")) OR TS=((Infan* AND Growth)) OR TI=((Infan* AND "Growth Retardation")) OR TI=((Infan* AND "Growth Disorder*")) OR TI=((Infan* AND "Growth Falter*")) OR TI=((Infan* AND "Growth Deficit*")) OR TI=((Infan* AND "Growth failure")) OR TI=((Infan* AND "Growth Trajectory")) OR TI=((Infan* AND "Nutrition Disorder*")) OR TS=((Infan* AND Malnutrition)) OR TI=((Infan* AND Undernutrition)) OR TI=((Infan* AND Under-nutrition)) OR TI=((Infan* AND Undernourish*)) OR TI=((Infan* AND Overnutrition)) OR TI=((Infan* AND Underweight)) OR TI=((Infan* AND Leanness)) OR TI=((Infan* AND Thinness)) OR TI=((Infan* AND Slimness)) OR TI=((Infan* AND Overweight)) OR TI=((Infan* AND Obes*)) OR TS=((Infan* AND Height*)) OR TS=((Infan* AND Weight*)) OR TS=((Infan* AND Length)) OR TI=((infan* AND "failure to thrive")) OR TS=((Under-five AND Growth)) OR TI=((Under-five AND "Growth Retardation")) OR TI=((Under-five AND "Growth Disorder*")) OR TI=((Under-five AND "Growth Falter*")) OR TI=((Under-five AND "Growth Deficit*")) OR TI=((Under-five AND "Growth failure")) OR TI=((Under-five AND "Growth Trajectory")) OR TI=((Under-five AND "Nutrition Disorder*")) OR TS=((Under-five AND Malnutrition)) OR TI=((Under-five AND Undernutrition)) OR TI=((Under-five AND Under-nutrition)) OR TI=((Under-five AND Undernourish*)) OR TI=((Under-five AND Overnutrition)) OR TI=((Under-five AND Underweight)) OR TS=((Under-five AND Leanness)) OR TI=((Under-five AND Thinness)) OR TI=((Under-five AND Slimness)) OR TI=((Under-five AND Overweight)) OR TI=((Under-five AND Obes*)) OR TS=((Under-five AND Height*)) OR TS=((Under-five AND Weight*)) OR TS=((Under-five AND Length)) OR TI=((under-five AND "failure to thrive")) OR TS=(("under five" AND Growth)) OR TI=(("under five" AND "Growth Retardation")) OR TI=(("under five" AND "Growth Disorder*")) OR TI=(("under five" AND "Growth Falter*")) OR TI=(("under five" AND "Growth Deficit*")) OR TI=(("under five" AND "Growth failure")) OR TI=(("under five" AND "Growth Trajectory")) OR TI=(("under five" AND "Nutrition Disorder*")) OR TS=(("under five" AND Malnutrition)) OR TI=(("under five" AND Undernutrition)) OR TI=(("under five" AND Under-nutrition)) OR TI=(("under five" AND Undernourish*)) OR TI=(("under five" AND Overnutrition)) OR TI=(("under five" AND Underweight)) OR TI=(("under five" AND Leanness)) OR TI=(("under five" AND Thinness)) OR TI=(("under five" AND Slimness)) OR TI=(("under five" AND Overweight)) OR TI=(("under five" AND Obes*)) OR TS=(("under five" AND Height*)) OR TS=(("under five" AND Weight*)) OR TS=(("under five" AND Length)) OR TI=(("under five" AND "failure to thrive")) OR TS=(("under 5" AND Growth)) OR TI=(("under 5" AND "Growth Retardation")) OR TI=(("under 5" AND "Growth Disorder*")) OR TI=(("under 5" AND "Growth Falter*")) OR TI=(("under 5" AND "Growth Deficit*")) OR TI=(("under 5" AND "Growth failure"))OR TI=(("under 5" AND "Growth Trajectory")) OR TI=(("under 5" AND "Nutrition Disorder*")) OR TS=(("under 5" AND Malnutrition)) OR TI=(("under 5" AND Undernutrition)) OR TI=(("under 5" AND Under-nutrition)) OR TI=(("under 5" AND Undernourish*)) OR TI=(("under 5" AND Overnutrition)) OR TI=(("under 5" AND Underweight)) OR TI=(("under 5" AND Leanness)) OR TI=(("under 5" AND Thinness)) OR TI=(("under 5" AND Slimness)) OR TI=(("under 5" AND Overweight)) OR TI=(("under 5" AND Obes*)) OR TS=(("under 5" AND Height*)) OR TS=(("under 5" AND Weight*)) OR TS=(("under 5" AND Length)) OR TI=(("under 5" AND "failure to thrive")) OR TS=((Under-5 AND Growth)) OR TI=((Under-5 AND "Growth Retardation")) OR TI=((Under-5 AND "Growth Disorder*")) OR TI=((Under-5 AND "Growth Falter*")) OR TI=((Under-5 AND "Growth Deficit*")) OR TI=((Under-5 AND "Growth failure")) OR TI=((Under-5 AND "Growth Trajectory")) OR TI=((Under-5 AND "Nutrition Disorder*")) OR TS=((Under-5 AND Malnutrition)) OR TI=((Under-5 AND Undernutrition)) OR TI=((Under-5 AND Under-nutrition)) OR TI=((Under-5 AND Undernourish*)) OR TI=((Under-5 AND Overnutrition)) OR TI=((Under-5 AND Underweight)) OR TI=((Under-5 AND Leanness)) OR TI=((Under-5 AND Thinness)) OR TI=((Under-5 AND Slimness)) OR TI=((Under-5 AND Overweight)) OR TI=((Under-5 AND Obes*)) OR TS=((Under-5 AND Height*)) OR TS=((Under-5 AND Weight*)) OR TS=((Under-5 AND Length)) OR TI=((Under-5 AND "failure to thrive")) OR TS=((Under-two AND Growth))*	**4,266**	**4,259**	**4,259**

OR TI=((Under-two AND "Growth Retardation")) OR TI=((Under-two AND "Growth Disorder*")) OR TI=((Under-two AND "Growth Falter*")) OR TI=((Under-two AND "Growth Deficit*")) OR TI=((Under-two AND "Growth failure")) OR TI=((Under-two AND "Growth Trajectory")) OR TI=((Under-two AND "Nutrition Disorder*")) OR TS=((Under-two AND Malnutrition)) OR TI=((Under-two AND Undernutrition)) OR TI=((Under-two AND Under-nutrition)) OR TI=((Under-two AND Undernourish*)) OR TI=((Under-two AND Overnutrition)) OR TI=((Under-two AND Underweight)) OR TI=((Under-two AND Leanness)) OR TI=((Under-two AND Thinness)) OR TI=((Under-two AND Slimness)) OR TS=((Under-two AND Overweight)) OR TI=((Under-two AND Obes*)) OR TS=((Under-two AND Height*)) OR TS=((Under-two AND Weight*)) OR TS=((Under-two AND Length)) OR TI=((Under-two AND "failure to thrive")) OR TS=(("under two" AND Growth)) OR TI=(("under two" AND "Growth Retardation")) OR TI=(("under two" AND "Growth Disorder*")) OR TI=(("under two" AND "Growth Falter*")) OR TI=(("under two" AND "Growth Deficit*")) OR TI=(("under two" AND "Growth failure")) OR TI=(("under two" AND "Growth Trajectory")) OR TI=(("under two" AND "Nutrition Disorder*")) OR TS=(("under two" AND Malnutrition)) OR TI=(("under two" AND Undernutrition)) OR TI=(("under two" AND Under-nutrition)) OR TI=(("under two" AND Undernourish*)) OR TI=(("under two" AND Overnutrition)) OR TI=(("under two" AND Underweight)) OR TI=(("under two" AND Leanness)) OR TI=(("under two" AND Thinness)) OR TI=(("under two" AND Slimness)) OR TI=(("under two" AND Overweight)) OR TI=(("under two" AND Obes*)) OR TS=(("under two" AND Height*)) OR TS=(("under two" AND Weight*)) OR TS=(("under two" AND Length)) OR TI=(("under two" AND "failure to thrive")) OR TS=(("under 2" AND Growth)) OR TI=(("under 2" AND "Growth Retardation")) OR TI=(("under 2" AND "Growth Disorder*")) OR TI=(("under 2" AND "Growth Falter*")) OR TI=(("under 2" AND "Growth Deficit*")) OR TI=(("under 2" AND "Growth failure")) OR TI=(("under 2" AND "Growth Trajectory")) OR TI=(("under 2" AND "Nutrition Disorder*")) OR TS=(("under 2" AND Malnutrition)) OR TI=(("under 2" AND Undernutrition)) OR TI=(("under 2" AND Under-nutrition)) OR TI=(("under 2"AND Undernourish*)) OR TI=(("under 2" AND Overnutrition)) OR TI=(("under 2" AND Underweight)) OR TI=(("under 2" AND Leanness)) OR TI=(("under 2" AND Thinness)) OR TI=(("under 2" AND Slimness)) OR TI=(("under 2" AND Overweight)) OR TI=(("under 2" AND Obes*)) OR TS=(("under 2" AND Height*)) OR TS=(("under 2" AND Weight*)) OR TS=(("under 2" AND Length)) OR TI=(("under 2" AND "failure to thrive")) OR TS=((Under-2 AND Growth)) OR TI=((Under-2 AND "Growth Retardation")) OR TI=((Under-2 AND "Growth Disorder*")) OR TI=((Under-2 AND "Growth Falter*")) OR TI=((Under-2 AND "Growth Deficit*")) OR TI=((Under-2 AND "Growth failure")) OR TI=((Under-2 AND "Growth Trajectory")) OR TI=((Under-2 AND "Nutrition Disorder*")) OR TS=((Under-2 AND Malnutrition)) OR TI=((Under-2 AND Undernutrition)) OR TI=((Under-2 AND Under-nutrition)) OR TI=((Under-2 AND Undernourish*)) OR TI=((Under-2 AND Overnutrition)) OR TI=((Under-2 AND Underweight)) OR TI=((Under-2 AND Leanness)) OR TI=((Under-2 AND Thinness)) OR TI=((Under-2 AND Slimness)) OR TI=((Under-2 AND Overweight)) OR TI=((Under-2 AND Obes*)) OR TS=((Under-2 AND Height*)) OR TS=((Under-2 AND Weight*)) OR TS=((Under-2 AND Length)) OR TI=((Under-2 AND "failure to thrive")) OR TS=((Offspring AND Growth)) OR TI=((Offspring AND "Growth Retardation")) OR TI=((Offspring AND "Growth Disorder*")) OR TI=((Offspring AND "Growth Falter*")) OR TI=((Offspring AND "Growth Deficit*")) OR TI=((Offspring AND "Growth failure")) OR TI=((Offspring AND "Growth Trajectory")) OR TI=((Offspring AND "Nutrition Disorder*")) OR TS=((Offspring AND Malnutrition)) OR TI=((Offspring AND Undernutrition)) OR TI=((Offspring AND Under-nutrition)) OR TI=((Offspring AND Undernourish*)) OR TI=((Offspring AND Overnutrition)) OR TI=((Offspring AND Underweight)) OR TI=((Offspring AND Leanness)) OR TI=((Offspring AND Thinness)) OR TI=((Offspring AND Slimness)) OR TI=((Offspring AND Overweight)) OR TI=((Offspring AND Obes*)) OR TS=((Offspring AND Height*)) OR TS=((Offspring AND Weight*)) OR TS=((Offspring AND Length)) OR TI=((Offspring AND "failure to thrive")) OR TS=((Early-life AND Growth)) OR TI=((Early-life AND "Growth Retardation")) OR TI=((Early-life AND "Growth Disorder*")) OR TI=((Early-life AND "Growth Falter*")) OR TI=((Early-life AND "Growth Deficit*")) OR TI=((Early-life AND "Growth failure")) OR TI=((Early-life AND "Growth Trajectory")) OR TI=((Early-life AND "Nutrition Disorder*")) OR TS=((Early-life AND Malnutrition)) OR TI=((Early-life AND Undernutrition)) OR TI=((Early-life AND Under-nutrition)) OR TI=((Early-life AND Undernourish*)) OR TI=((Early-life AND Overnutrition)) OR TI=((Early-life AND Underweight)) OR TI=((Early-life AND Leanness)) OR TI=((Early-life AND Thinness)) OR TI=((Early-life AND Slimness)) OR TI=((Early-life AND Overweight)) OR TI=((Early-life AND Obes*)) OR TS=((Early-life AND Height*)) OR TS=((Early-life AND Weight*)) OR TS=((Early-life AND Length)) OR TI=((Early-life AND "failure to thrive")) OR TS=(("Early life" AND Growth)) OR TI=(("Early life" AND "Growth Retardation")) OR TI=(("Early life" AND "Growth Disorder*")) OR TI=(("Early life" AND "Growth Falter*")) OR TI=(("Early life" AND "Growth Deficit*")) OR TI=(("Early life" AND "Growth failure")) OR TI=(("Early life" AND "Growth Trajectory")) OR TI=(("Early life" AND "Nutrition Disorder*")) OR TS=(("Early life" AND Malnutrition)) OR TI=(("Early life" AND Undernutrition)) OR TI=(("Early life" AND Under-nutrition)) OR TI=(("Early life" AND Undernourish*)) OR TI=(("Early life" AND Overnutrition)) OR TI=(("Early life" AND Underweight)) OR TI=(("Early life" AND Leanness)) OR TI=(("Early life" AND Thinness)) OR TI=(("Early life" AND Slimness)) OR TI=(("Early life" AND Overweight)) OR TI=(("Early life" AND Obes*)) OR TS=(("Early life" AND Height*)) OR TS=(("Early life" AND Weight*)) OR TS=(("Early life" AND Length)) OR TI=(("Early life" AND "failure to thrive")) OR TS=((Pediatric AND Growth)) OR TI=((Pediatric AND "Growth Retardation")) OR TI=((Pediatric AND "Growth Disorder*")) OR TI=((Pediatric AND "Growth Falter*")) OR TI=((Pediatric AND "Growth Deficit*")) OR TI=((Pediatric AND "Growth failure")) OR TI=((Pediatric AND "Growth Trajectory")) OR TI=((Pediatric AND "Nutrition Disorder*")) OR TS=((Pediatric AND Malnutrition)) OR TI=((Pediatric AND Undernutrition)) OR TI=((Pediatric AND Under-nutrition)) OR TI=((Pediatric AND Undernourish*)) OR TI=((Pediatric AND Overnutrition)) OR TI=((Pediatric AND Underweight)) OR TI=((Pediatric AND Leanness)) OR TI=((Pediatric AND Thinness)) OR TI=((Pediatric AND Slimness)) OR TI=((Pediatric AND Overweight)) OR TI=((Pediatric AND Obes*)) OR TS=((Pediatric AND Height*)) OR TS=((Pediatric AND Weight*)) OR TS=((Pediatric AND Length)) OR TI=((Pediatric AND "failure to thrive")) OR TI=("child health")) AND (TS=(cohort) OR TS=(longitudinal) OR TS=((Cohort AND Study)) OR TS=((Concurrent AND Study)) OR TS=("birth cohort") OR TS=((cohort AND analysis)) OR TS=((cohort AND analyses)) OR TS=("incidence study") OR TS=("Follow Up Study") OR TS=("Follow-Up Study") OR TS=("Followup Study") OR TS=((Longitudinal AND Study)) OR TS=((Prospective AND study))) AND ((PY=(1990-2024))))			

➢ Embase Syntax

EMBASE *Syntax*	*Conference paper*	*Original Articles*	*Output No.*	*Output No. after importing to mendely and removing unacceptable dates*	*Output No. after removing duplicates*
('maternal educational status':ti OR ('educational status':ti AND maternal:ti) OR (status:ti AND 'maternal educational':ti) OR (maternal:ti AND education:ti) OR (maternal:ti AND illiteracy:ti) OR (maternal:ti AND literacy:ti) OR (('maternal'/exp OR maternal) AND ('schooling'/exp OR 'schooling')) OR (mother:ti,ab AND education:ti,ab) OR (mother*:ti AND illiteracy:ti) OR (mother*:ti AND literacy:ti) OR (mother* AND ('schooling'/exp OR 'schooling')) OR (parent*:ti AND illiteracy:ti) OR (parent*:ti AND literacy:ti) OR (parent*:ti AND education:ti) OR (parent*:ti AND 'schooling':ti) OR (parent*:ti AND 'socio economic':ti) OR (parent*:ti AND socioeconomic:ti) OR (parent*:ti AND 'social class*':ti) OR (parent*:ti AND 'socio demographic':ti) OR (parent*:ti AND sociodemographic:ti) OR (parent*:ti AND 'socio cultural':ti) OR (parent*:ti AND sociocultural:ti) OR (mother*:ti,ab AND 'socio economic':ti,ab) OR (mother*:ti,ab AND socioeconomic:ti,ab) OR (mother*:ti AND 'social class*':ti) OR (mother*:ti,ab AND 'socio demographic':ti,ab) OR (mother*:ti,ab AND sociodemographic:ti,ab) OR (mother*:ti AND 'socio cultural':ti) OR (mother*:ti AND sociocultural:ti) OR (maternal:ti,ab AND 'socio economic':ti,ab) OR (maternal:ti,ab AND socioeconomic:ti,ab) OR (maternal:ti AND 'social class*':ti) OR (maternal:ti,ab AND 'socio demographic':ti,ab) OR (maternal:ti,ab AND sociodemographic:ti,ab) OR (maternal:ti AND 'socio cultural':ti) OR (maternal:ti AND sociocultural:ti) OR 'maternal socioeconomic status':ti OR 'parental socioeconomic status':ti) AND (stunting*:ti OR 'stunted growth':ti OR (child*:ti,ab AND growth:ti,ab) OR (child*:ti AND 'growth retardation':ti) OR (child*:ti AND 'growth disorder*':ti) OR (child*:ti AND 'growth falter*':ti) OR (child*:ti AND 'growth deficit*':ti) OR (child*:ti AND 'growth failure':ti) OR (child*:ti AND 'growth trajectory':ti) OR (growth:ti AND stunted:ti) OR 'short stature':ti OR wast*:ti,ab OR (child*:ti AND 'nutrition disorder*':ti) OR (child*:ti,ab AND malnutrition:ti,ab) OR (child*:ti AND undernutrition:ti) OR (child*:ti AND 'under nutrition':ti) OR (child*:ti AND undernourish*:ti) OR (child*:ti AND overnutrition:ti) OR (child*:ti AND underweight:ti) OR (child*:ti AND leanness:ti) OR (child*:ti AND thinness:ti) OR (child*:ti AND slimness:ti) OR (child*:ti AND overweight:ti) OR (child*:ti AND obes*:ti) OR (child*:ti,ab AND height*:ti,ab) OR (child*:ti,ab AND weight*:ti,ab) OR (child*:ti,ab AND length:ti,ab) OR anthropometric*:ti OR 'length for age':ti,ab OR 'height for age':ti,ab OR 'weight for age':ti,ab OR 'weight for height':ti,ab OR weight-for-length:ti,ab OR 'body mass index-for-age':ti,ab OR 'bmi for age':ti,ab OR (child*:ti AND 'failure to thrive':ti) OR (infan*:ti,ab AND growth:ti,ab) OR (infan*:ti AND 'growth retardation':ti) OR (infan*:ti AND 'growth disorder*':ti) OR (infan*:ti AND 'growth falter*':ti) OR (infan*:ti AND 'growth deficit*':ti) OR (infan*:ti AND 'growth failure':ti) OR (infan*:ti AND 'growth trajectory':ti) OR (infan*:ti AND 'nutrition disorder*':ti) OR (infan*:ti,ab AND malnutrition:ti,ab) OR (infan*:ti AND undernutrition:ti) OR (infan*:ti AND 'under nutrition':ti) OR (infan*:ti AND undernourish*:ti) OR (infan*:ti AND overnutrition:ti) OR (infan*:ti AND underweight:ti) OR (infan*:ti AND leanness:ti) OR (infan*:ti AND thinness:ti) OR (infan*:ti AND slimness:ti) OR (infan*:ti AND overweight:ti) OR (infan*:ti AND obes*:ti) OR (infan*:ti,ab AND height*:ti,ab) OR (infan*:ti,ab AND weight*:ti,ab) OR (infan*:ti,ab AND length:ti,ab) OR (infan*:ti AND 'failure to thrive':ti) OR ('under five':ti,ab AND leanness:ti) OR ('under five':ti,ab AND growth:ti,ab) OR ('under five':ti AND 'growth retardation':ti) OR ('under five':ti AND 'growth disorder*':ti) OR ('under five':ti AND 'growth falter*':ti) OR ('under five':ti AND 'growth deficit*':ti) OR ('under five':ti AND 'growth failure':ti) OR ('under five':ti AND 'growth trajectory':ti) OR ('under five':ti AND 'nutrition disorder*':ti) OR ('under five':ti,ab AND malnutrition:ti,ab) OR ('under five':ti AND undernutrition:ti) OR ('under five':ti AND 'under nutrition':ti) OR ('under five':ti AND undernourish*:ti) OR ('under five':ti AND overnutrition:ti) OR ('under five':ti AND underweight:ti) OR ('under five':ti AND leanness:ti) OR ('under five':ti AND thinness:ti) OR ('under five':ti AND slimness:ti) OR ('under five':ti AND overweight:ti) OR ('under five':ti AND obes*:ti) OR ('under five':ti,ab AND height*:ti,ab) OR ('under five':ti,ab AND weight*:ti,ab) OR ('under five':ti,ab AND length:ti,ab) OR ('under five':ti AND 'failure to thrive':ti) OR ('under 5':ti,ab AND growth:ti,ab) OR ('under 5':ti AND 'growth retardation':ti) OR ('under 5':ti AND 'growth disorder*':ti) OR ('under 5':ti AND 'growth falter*':ti) OR ('under 5':ti AND 'growth deficit*':ti) OR ('under 5':ti AND 'growth failure':ti) OR ('under 5':ti AND 'growth trajectory':ti) OR ('under 5':ti AND 'nutrition disorder*':ti) OR ('under 5':ti,ab AND malnutrition:ti,ab) OR ('under 5':ti AND undernutrition:ti) OR ('under*			**4,202**	***4,095***	***4,087***

5':ti AND 'under nutrition':ti) OR ('under 5':ti AND undernourish*:ti) OR ('under 5':ti AND overnutrition:ti) OR ('under 5':ti AND underweight:ti) OR ('under 5':ti AND leanness:ti) OR ('under 5':ti AND thinness:ti) OR ('under 5':ti AND slimness:ti) OR ('under 5':ti AND overweight:ti) OR ('under 5':ti AND obes*:ti) OR ('under 5':ti,ab AND height*:ti,ab) OR ('under 5':ti,ab AND weight*:ti,ab) OR ('under 5':ti,ab AND length:ti,ab) OR ('under 5':ti AND 'failure to thrive':ti) OR ('under two':ti,ab AND overweight:ti,ab) OR ('under two':ti,ab AND growth:ti,ab) OR ('under two':ti AND 'growth retardation':ti) OR ('under two':ti AND 'growth disorder*':ti) OR ('under two':ti AND 'growth falter*':ti) OR ('under two':ti AND 'growth deficit*':ti) OR ('under two':ti AND 'growth failure':ti) OR ('under two':ti AND 'growth trajectory':ti) OR ('under two':ti AND 'nutrition disorder*':ti) OR ('under two':ti,ab AND malnutrition:ti,ab) OR ('under two':ti AND undernutrition:ti) OR ('under two':ti AND 'under nutrition':ti) OR ('under two':ti AND undernourish*:ti) OR ('under two':ti AND overnutrition:ti) OR ('under two':ti AND underweight:ti) OR ('under two':ti AND leanness:ti) OR ('under two':ti AND thinness:ti) OR ('under two':ti AND slimness:ti) OR ('under two':ti AND overweight:ti) OR ('under two':ti AND obes*:ti) OR ('under two':ti,ab AND height*:ti,ab) OR ('under two':ti,ab AND weight*:ti,ab) OR ('under two':ti,ab AND length:ti,ab) OR ('under two':ti AND 'failure to thrive':ti) OR ('under 2':ti,ab AND growth:ti,ab) OR ('under 2':ti AND 'growth retardation':ti) OR ('under 2':ti AND 'growth disorder*':ti) OR ('under 2':ti AND 'growth falter*':ti) OR ('under 2':ti AND 'growth deficit*':ti) OR ('under 2':ti AND 'growth failure':ti) OR ('under 2':ti AND 'growth trajectory':ti) OR ('under 2':ti AND 'nutrition disorder*':ti) OR ('under 2':ti,ab AND malnutrition:ti,ab) OR ('under 2':ti AND undernutrition:ti) OR ('under 2':ti AND 'under nutrition':ti) OR ('under 2':ti AND undernourish*:ti) OR ('under 2':ti AND overnutrition:ti) OR ('under 2':ti AND underweight:ti) OR ('under 2':ti AND leanness:ti) OR ('under 2':ti AND thinness:ti) OR ('under 2':ti AND slimness:ti) OR ('under 2':ti AND overweight:ti) OR ('under 2':ti AND obes*:ti) OR ('under 2':ti,ab AND height*:ti,ab) OR ('under 2':ti,ab AND weight*:ti,ab) OR ('under 2':ti,ab AND length:ti,ab) OR ('under 2':ti AND 'failure to thrive':ti) OR (offspring:ti,ab AND growth:ti,ab) OR (offspring:ti AND 'growth retardation':ti) OR (offspring:ti AND 'growth disorder*':ti) OR (offspring:ti AND 'growth falter*':ti) OR (offspring:ti AND 'growth deficit*':ti) OR (offspring:ti AND 'growth failure':ti) OR (offspring:ti AND 'growth trajectory':ti) OR (offspring:ti AND 'nutrition disorder*':ti) OR (offspring:ti,ab AND malnutrition:ti,ab) OR (offspring:ti AND undernutrition:ti) OR (offspring:ti AND 'under nutrition':ti) OR (offspring:ti AND undernourish*:ti) OR (offspring:ti AND overnutrition:ti) OR (offspring:ti AND underweight:ti) OR (offspring:ti AND leanness:ti) OR (offspring:ti AND thinness:ti) OR (offspring:ti AND slimness:ti) OR (offspring:ti AND overweight:ti) OR (offspring:ti AND obes*:ti) OR (offspring:ti,ab AND height*:ti,ab) OR (offspring:ti,ab AND weight*:ti,ab) OR (offspring:ti,ab AND length:ti,ab) OR (offspring:ti AND 'failure to thrive':ti) OR ('early life':ti,ab AND growth:ti,ab) OR ('early life':ti AND 'growth retardation':ti) OR ('early life':ti AND 'growth disorder*':ti) OR ('early life':ti AND 'growth falter*':ti) OR ('early life':ti AND 'growth deficit*':ti) OR ('early life':ti AND 'growth failure':ti) OR ('early life':ti AND 'growth trajectory':ti) OR ('early life':ti AND 'nutrition disorder*':ti) OR ('early life':ti,ab AND malnutrition:ti,ab) OR ('early life':ti AND undernutrition:ti) OR ('early life':ti AND 'under nutrition':ti) OR ('early life':ti AND undernourish*:ti) OR ('early life':ti AND overnutrition:ti) OR ('early life':ti AND underweight:ti) OR ('early life':ti AND leanness:ti) OR ('early life':ti AND thinness:ti) OR ('early life':ti AND slimness:ti) OR ('early life':ti AND overweight:ti) OR ('early life':ti AND obes*:ti) OR ('early life':ti,ab AND height*:ti,ab) OR ('early life':ti,ab AND weight*:ti,ab) OR ('early life':ti,ab AND length:ti,ab) OR ('early life':ti AND 'failure to thrive':ti) OR (pediatric:ti,ab AND growth:ti,ab) OR (pediatric:ti AND 'growth retardation':ti) OR (pediatric:ti AND 'growth disorder*':ti) OR (pediatric:ti AND 'growth falter*':ti) OR (pediatric:ti AND 'growth deficit*':ti) OR (pediatric:ti AND 'growth failure':ti) OR (pediatric:ti AND 'growth trajectory':ti) OR (pediatric:ti AND 'nutrition disorder*':ti) OR (pediatric:ti,ab AND malnutrition:ti,ab) OR (pediatric:ti AND undernutrition:ti) OR (pediatric:ti AND 'under nutrition':ti) OR (pediatric:ti AND undernourish*:ti) OR (pediatric:ti AND overnutrition:ti) OR (pediatric:ti AND underweight:ti) OR (pediatric:ti AND leanness:ti) OR (pediatric:ti AND thinness:ti) OR (pediatric:ti AND slimness:ti) OR (pediatric:ti AND overweight:ti) OR (pediatric:ti AND obes*:ti) OR (pediatric:ti,ab AND height*:ti,ab) OR (pediatric:ti,ab AND weight*:ti,ab) OR (pediatric:ti,ab AND length:ti,ab) OR (pediatric:ti AND 'failure to thrive':ti) OR 'child health':ti) AND (cohort:ti,ab OR longitudinal:ti,ab OR (cohort:ti,ab AND study:ti,ab) OR (concurrent:ti,ab AND study:ti,ab) OR 'birth cohort':ti,ab OR (cohort:ti,ab AND analysis:ti,ab) OR (cohort:ti,ab AND analyses:ti,ab) OR 'incidence study':ti,ab OR 'follow up study':ti,ab OR 'follow-up study':ti,ab OR 'followup study':ti,ab OR (longitudinal:ti,ab AND study:ti,ab) OR (prospective:ti,ab AND study:ti,ab)) AND [1990-2024]/py					

➢ ERIC Syntax

ERIC Synthax	Output No.	Output No. after importing to Mendeley and removing unacceptable dates	Output No. after removing duplicates
((maternal OR mother OR title:parent) AND (schooling OR education OR educational OR socio-economic OR socioeconomic OR socio-demographic OR sociodemographic OR socio-cultural OR sociocultural or title:"social class" OR title:Illiteracy OR title:literacy)) AND (Length-for-age OR Height-for-age OR Weight-for-age OR Weight-for-height OR weight-for-length OR "Body mass index-for-age" OR BMI-for-age OR title:anthropometric OR title:anthropometrics OR ((child OR children OR infant OR infantile OR infancy OR under-five OR "under five" OR "under 5" OR under-5 OR under-two OR under-2 OR "under 2" OR "under two" OR Offspring OR early-life OR "early life" OR pediatric OR pediatrics) AND (Growth OR title:"Nutrition Disorders" OR title:stunting OR title:stunted OR title:"short stature" OR wasting OR malnutrition OR title:undernutrition OR title:under-nutrition OR title:undernourished OR title:overnutrition OR title:underweight OR title:leanness OR title:thinness OR title:slimness OR title:overweight OR title:obesity OR title:obese OR Height OR weight OR length OR title:"failure to thrive" OR "child health"))) AND (cohort OR longitudinal OR (Concurrent AND Study) OR "incidence study" OR "Follow Up Study" OR "Follow-Up Study" OR "Followup Study" OR (Prospective AND study)) pubyearmin:1990 pubyearmax:2024	**1368**	**1364**	**1363**

➢ Google Scholar Syntax

Google scholar Synthax	Output No.	Output No. after importing to Mendeley and removing unacceptable dates	Output No. after removing duplicates
allintitle: (Education | schooling | socioeconomic | socio-economic | sociodemographic | "social class") + (growth | stunting | malnutrition | underweight | obesity | overweight | anthropometric | BMI | "failure to thrive" | weight | height) + cohort	207		
allintitle:(Education | schooling | socioeconomic | socio-economic | sociodemographic | "social class") + (growth | stunting | malnutrition | obesity | overweight | anthropometric | BMI | "failure to thrive" | weight | height)+longitudinal	159		
allintitle:(Education | schooling | socioeconomic | socio-economic | sociodemographic | "social class") + (growth | stunting | malnutrition | obesity | overweight | anthropometric | BMI | "failure to thrive" | weight | height)+prospective	86		
allintitle:(Education | schooling | socioeconomic | socio-economic | sociodemographic | "social class") + (growth | stunting | malnutrition | obesity | overweight | anthropometric | BMI | "failure to thrive" | weight | height)+follow-up	53		
allintitle:(Education | schooling | socioeconomic | socio-economic | sociodemographic | "social class") + "child health" + cohort	14		
allintitle:(Education | schooling | socioeconomic | socio-economic | sociodemographic | socio-demographic | "social class") + "child health" + follow-up	6		
allintitle:(Education | schooling | socioeconomic | socio-economic | sociodemographic | socio-demographic | "social class") + "child health" + prospective	4		
allintitle:(Education | schooling | socioeconomic | socio-economic | sociodemographic | socio-demographic | "social class") + Body mass index + cohort	36		
allintitle:(Education | schooling | socioeconomic | socio-economic | sociodemographic | socio-demographic | "social class") + Body mass index + longitudinal	27		
allintitle:(Education | schooling | socioeconomic | socio-economic | sociodemographic | socio-demographic | "social class") + Body mass index + prospective	5		
allintitle:(Education | schooling | socioeconomic | socio-economic | sociodemographic | socio-demographic | "social class") + Body mass index + follow-up	5		
My library output	**468**	**417**	**398**
