# Supplementary material for: Maternal education and its influence on child growth and nutritional status during the first two years of life: a systematic review and meta-analysis
Source: eClinicalMedicine. 2024 Apr 4;71:102574. doi: 10.1016/j.eclinm.2024.102574 (PMC11001623; doi:10.1016/j.eclinm.2024.102574)
Supplement: Supplementary File 2 [file mmc2.pdf]

Supplementary 2.

❖ Modified method of GRADE

Downgrading Factors*		
**Risk of bias†**	• All the studies had good and fair quality<br>• Less than 50% of studies had poor quality<br>• 50% or more of the studies had poor quality	0<br>-1<br>-2
**Inconsistency**	• I^2 : 0-40%<br>• I^2 : 30-60%<br>• I^2 : 50-100%	0<br>-1<br>-2
**Imprecision¶**	• The 95% CI of the association measure did not cross the clinical decision thresholdsµ, or the number of studies in the subgroup was greater than 5.<br>• The 95% CI of the association measure crossed one clinical decision threshold, or the number of studies in the subgroup was 4/5.<br>• The 95% CI of association measure crossed more than one clinical decision thresholds or the number of studies in the subgroup was ≤3	0<br>-1<br>-2
**Upgrading Factors¥**		
**Large effect**	• SMD€: <0.2; OR: 0.8-1/1-1.2<br>• SMD: 0.2-0.5; OR: 0.5-0.8/1.2-2<br>• SMD: 0.5 to >0.8; OR: <0.5/>2	0<br>+1<br>+2
**Dose-response effect**	• The effect estimate and its 95%CI conformed to the dose-response curve$<br>• The effect estimate and its 95%CI partially conformed to the dose-response curve<br>• The effect estimate and its 95%CI did not conform to the dose-response curve	0<br>+1<br>+2

* We omitted the assessment of indirectness from the downgrading factors because all included studies should be eligible based on the study question. Additionally, as our results differed in each subgroup, we assessed the certainty of evidence in each subgroup; therefore, we could not evaluate publication bias in subgroups with a small number of studies.
† Because all of the studies were population-based cohort studies, we did not consider the type of study as a factor affecting the GRADE score.
¶ If the 95% CI did not exclude "no effect," but fell within the range of -0.2 to 0.2 for SMDs or 0.8 to 1.2 for ORs, indicating a very small to no effect, we did not downgrade the GRADE score.
µ The thresholds used to interpret the magnitude of effect size were used as clinical decision thresholds.
¥ We omitted the consideration of opposing bias and confounding from the upgrading factors because most of our studies adjusted the association measure for mediators and not confounding factors.
€ The mean difference in WAZ, HAZ, and BMI-Z.
$ Dose-response curves were generated using the restricted cubic spline method and are displayed below.

- **Dose-response relationship between maternal education and child growth and nutritional status.**

WAZ

Middle-income

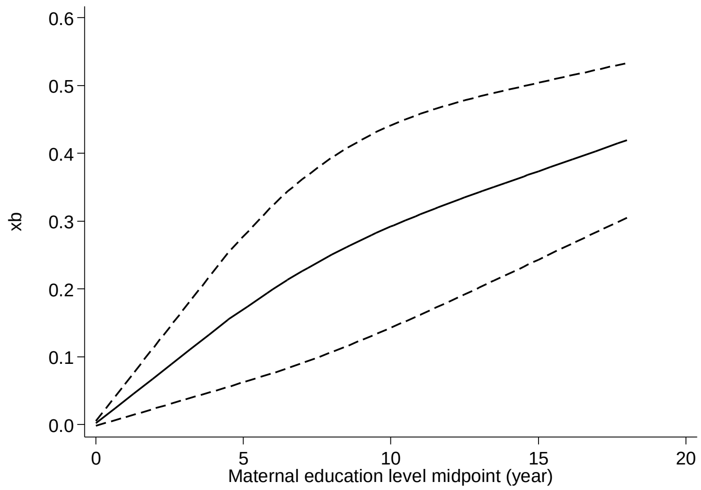

Midpoint[¥μ]	*xb*	*(95% CI)*
0	0.00	(-0.00-0.01) *
4.5	0.16	(0.06-0.25) *
6	0.20	(0.08-0.32) *
6.5	0.21	(0.08-0.34) *
7	0.23	(0.09-0.36) *
8	0.25	(0.11-0.39) *
8.5	0.26	(0.12-0.41) *
9.5	0.28	(0.13-0.43) *
10	0.29	(0.14-0.44) *
10.5	0.30	(0.15-0.45) *
11	0.31	(0.16-0.46) *
11.5	0.32	(0.17-0.47) *
12.5	0.34	(0.19-0.48) *
13	0.34	(0.20-0.48) *
13.5	0.35	(0.21-0.49) *
14.5	0.37	(0.23-0.50) *
15	0.37	(0.24-0.50) *
18	0.42	(0.31-0.53) *

¥ No. of studies=2

μ Midpoints were centered

**p<0.05*

High-income

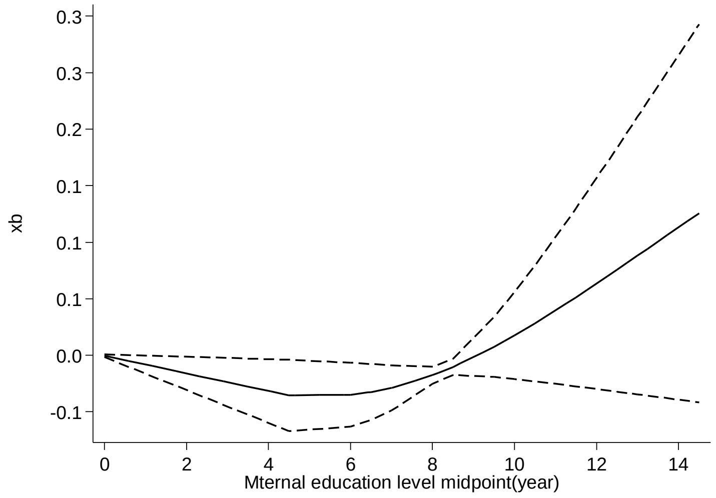

Midpoint[¥μ]	*xb*	*(95% CI)*
0	-0.00	(-0.00 - 0.00) *
4.5	-0.03	(-0.06 - -0.00) *
6	-0.03	(-0.06 - -0.01) *
6.5	-0.03	(-0.05 - -0.01) *
7	-0.03	(-0.05 - -0.01) *
8	-0.02	(-0.02 - -0.01) *
8.5	-0.01	(-0.02 - -0.00) *
9.5	0.01	(-0.02 - 0.03)
10	0.02	(-0.02 - 0.05)
10.5	0.03	(-0.02 - -0.08)
11	0.04	(-0.03 - 0.10)
11.5	0.05	(-0.03 - 0.13)
12.5	0.07	(-0.03 - 0.18)
13	0.09	(-0.04 - -0.21)
13.5	0.10	(-0.04 - 0.24)
14.5	0.13	(-0.05 - -0.30)
15	0.14	(-0.05 - 0.33)
18	0.22	(-0.06 - 0.50)

¥ No. of studies=8

μ Midpoints were centered

Low-educated

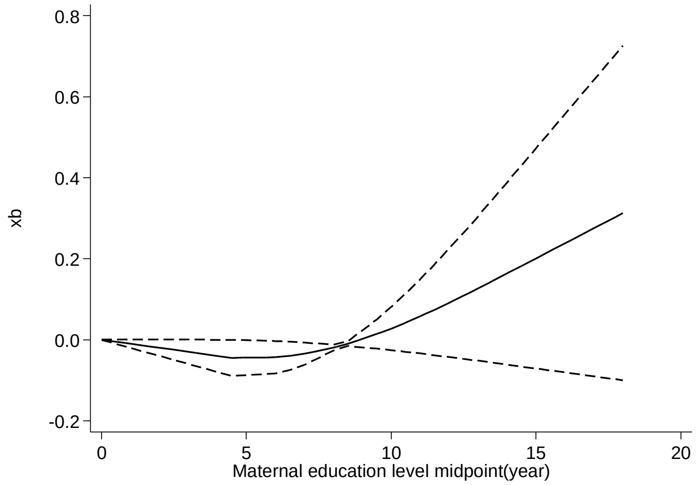

Midpoint ¥ µ	xb	(95% CI)
0	0.00	(-0.00-0.00) *
4.5	-0.04	(-0.09-0.00) *
6	-0.04	(-0.08--0.00) *
6.5	-0.04	(-0.07--0.00) *
7	-0.03	(-0.06--0.01) *
8	-0.02	(-0.03--0.01) *
8.5	-0.01	(-0.02--0.00) *
9.5	0.01	(-0.02-0.05)
10	0.03	(-0.03-0.08)
10.5	0.04	(-0.03-0.11)
11	0.06	(-0.03-0.15)
11.5	0.07	(-0.04-0.19)
12.5	0.11	(-0.05-0.26)
13	0.13	(-0.05-0.30)
13.5	0.14	(-0.06-0.35)
14.5	0.18	(-0.07-0.43)
15	0.20	(-0.07-0.47)
18	0.31	(-0.10-0.73)

¥ No. of studies=4

µ Midpoints were centered

$*p<0.05$

High-educated

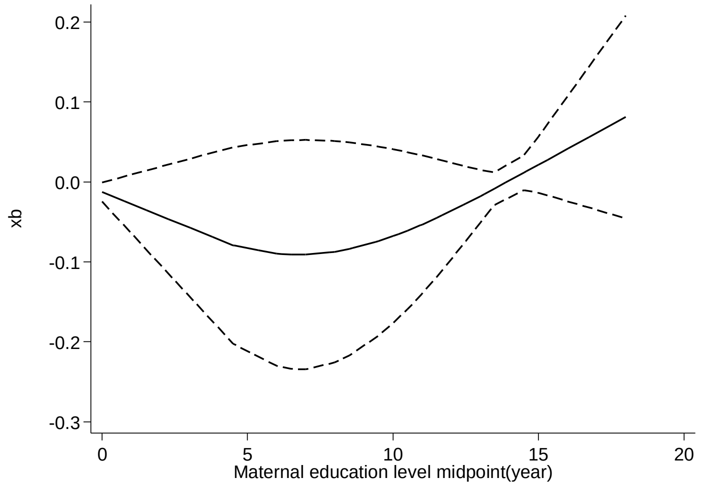

Midpoint ¥ µ	xb	(95% CI)
0	-0.01	(-0.02--0.00) *
4.5	-0.08	(-0.20-0.04)
6	-0.09	(-0.23-0.05)
6.5	-0.09	(-0.23-0.05)
7	-0.09	(-0.23-0.05)
8	-0.09	(-0.23-0.05)
8.5	-0.08	(-0.22-0.05)
9.5	-0.07	(-0.19-0.04)
10	-0.07	(-0.18-0.04)
10.5	-0.06	(-0.16-0.04)
11	-0.05	(-0.14-0.03)
11.5	-0.05	(-0.12-0.03)
12.5	-0.03	(-0.07-0.02)
13	-0.02	(-0.05-0.02)
13.5	-0.01	(-0.03-0.01)
14.5	0.01	(-0.01-0.03)
15	0.02	(-0.01-0.06)
18	0.08	(-0.05-0.21)

¥ No. of studies=6

µ Midpoints were centered

$*p<0.05$

➢ HAZ

Middle-income

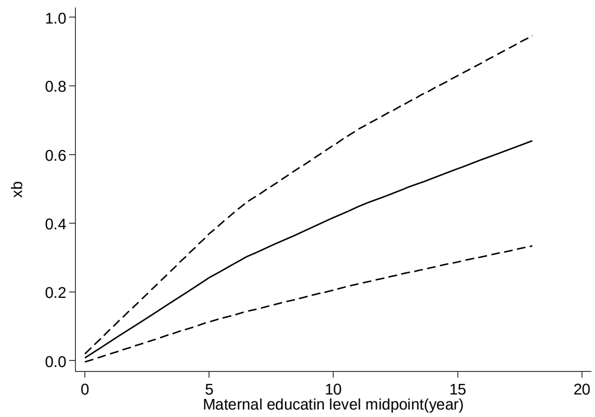

Midpoint¥μ	***xb***	***(95% CI)***
0	0.01	(-0.00-0.02)*
5	0.24	(0.11-0.37)*
6	0.28	(0.13-0.43)*
6.5	0.30	(0.14-0.46)*
11	0.45	(0.22-0.67)*
13	0.50	(0.26-0.75)*
13.5	0.52	(0.26-0.77)*
15	0.56	(0.29-0.83)*
18	0.64	(0.33-0.95)*

¥ No. of studies=5

μ Midpoints were centered

**p<0.05*

High-income

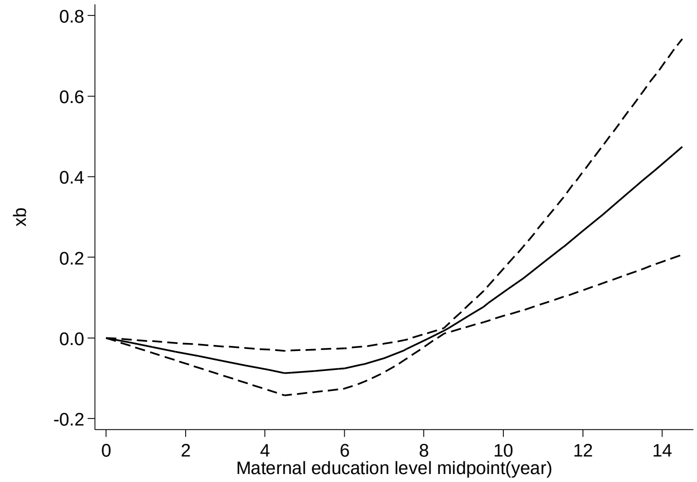

Midpoint¥μ	***xb***	***(95% CI)***
0	-0.00	(-0.00-0.00)*
4.5	-0.09	(-0.14--0.03)*
6	-0.08	(-0.13--0.03)*
6.5	-0.07	(-0.11--0.02)*
7	-0.05	(-0.09--0.01)*
7.5	-0.03	(-0.06--0.01)*
8.5	0.02	(0.01-0.02)*
9.5	0.08	(0.04-0.12)*
10.5	0.15	(0.07-0.23)*
11.5	0.22	(0.10-0.35)*
12.5	0.31	(0.14-0.48)*
13.5	0.39	(0.17-0.61)*
14.5	0.47	(0.21-0.74)*

¥ No. of studies=7

μ Midpoints were centered

**p<0.05*

Low-educated

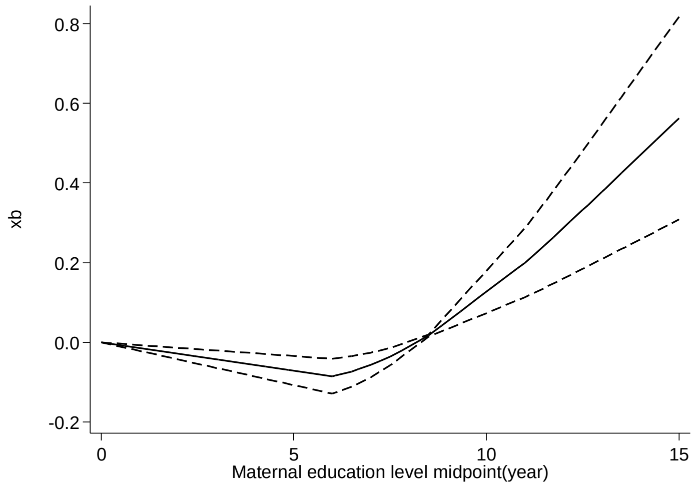

Midpoint ¥ µ	*xb*	*(95% CI)*
0	-0.00	(-0.00--0.00)*
6	-0.09	(-0.13--0.04)*
6.5	-0.07	(-0.11--0.03)*
7	-0.06	(-0.09--0.03)*
7.5	-0.04	(-0.06--0.01)*
8.5	0.02	(0.01-0.02)*
11	0.20	(0.11-0.29)*
11.5	0.24	(0.14-0.35)*
12.5	0.33	(0.18-0.48)*
13	0.38	(0.21-0.55)*
13.5	0.42	(0.23-0.61)*
15	0.56	(0.31-0.82)*

¥ No. of studies=6
µ Midpoints were centered
**p<0.05*

High-educated

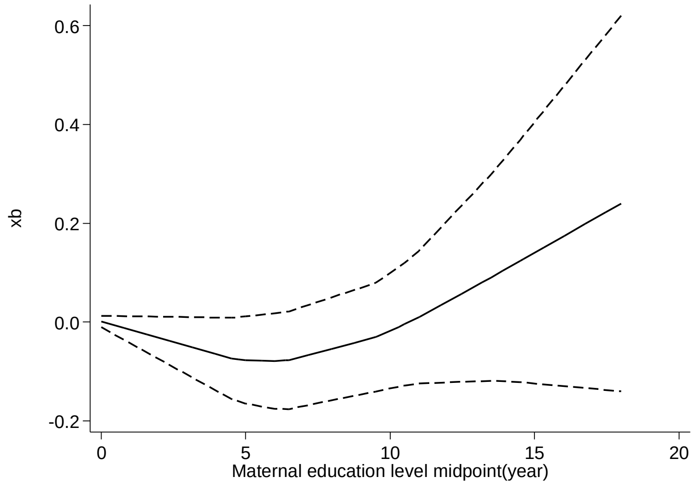

Midpoint ¥ µ	*xb*	*(95% CI)*
0	0.00	(-0.01-0.01)
4.5	-0.07	(-0.16-0.01)
5	-0.08	(-0.17-0.01)
6	-0.08	(-0.18-0.02)
6.5	-0.08	(-0.18-0.02)
9.5	-0.03	(-0.14-0.08)
10.5	-0.00	(-0.13-0.12)
11	0.01	(-0.12-0.14)
13.5	0.09	(-0.12-0.30)
14.5	0.12	(-0.12-0.37)
18	0.24	(-0.14-0.62)

¥ No. of studies=6
µ Midpoints were centered
**p<0.05*

- **BMI-Z**

Middle-income

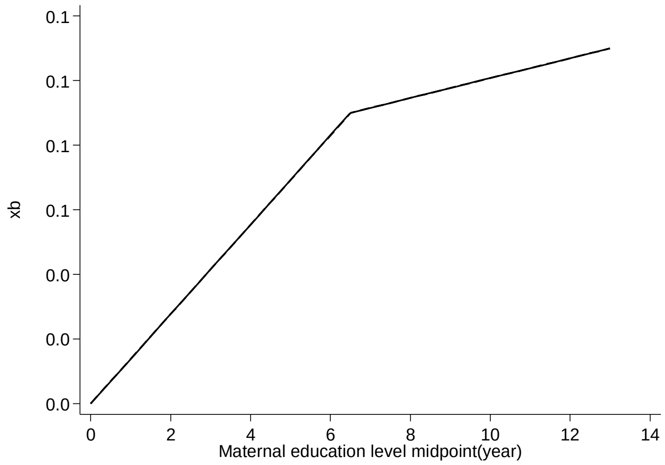

Midpoint ¥ µ	*xb*	*(95% CI)*
0	0.00	(0.00-0.00)*
6.5	0.09	(0.09-0.09)*
13	0.11	(0.11-0.11)*

¥ No. of studies=1
µ Midpoints were centered
**p<0.05*

High-income

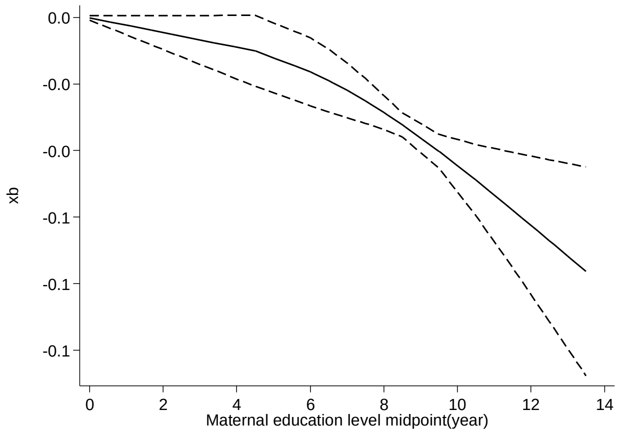

Midpoint ¥ µ	*xb*	*(95% CI)*
0	-0.00	(-0.00-0.00)*
4.5	-0.01	(-0.02-0.00)*
6	-0.02	(-0.03--0.01)*
6.5	-0.02	(-0.03--0.01)*
7	-0.02	(-0.03--0.01)*
7.5	-0.03	(-0.03--0.02)*
8	-0.03	(-0.03--0.02)*
8.5	-0.03	(-0.04--0.03)*
9.5	-0.04	(-0.05--0.04)*
10.5	-0.05	(-0.06--0.04)*
11.5	-0.06	(-0.08--0.04)*
12.5	-0.07	(-0.09--0.04)*
13	-0.07	(-0.10--0.04)*
13.5	-0.08	(-0.11--0.04)*

¥ No. of studies=9
µ Midpoints were centered
**p<0.05*

Low-educated

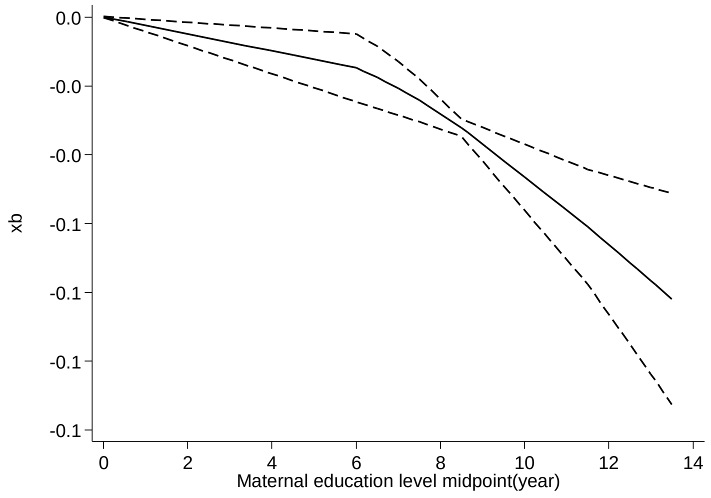

Midpoint ¥ µ	*xb*	*(95% CI)*
0	0.00	(-0.00-0.00)*
6	-0.01	(-0.02--0.00)*
6.5	-0.02	(-0.03--0.01)*
7	-0.02	(-0.03--0.01)*
7.5	-0.02	(-0.03--0.02)*
8.5	-0.03	(-0.03--0.03)*
11.5	-0.06	(-0.08--0.04)*
12.5	-0.07	(-0.10--0.05)*
13	-0.08	(-0.10--0.05)*
13.5	-0.08	(-0.11--0.05)*

¥ No. of studies=5
µ Midpoints were centered
**p<0.05*

High-educated

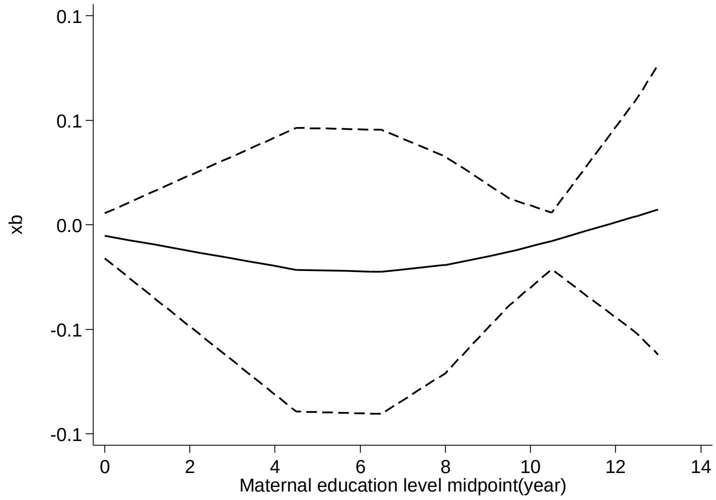

Midpoint ¥ µ	*xb*	*(95% CI)*
0	-0.01	(-0.02-0.01)
4.5	-0.02	(-0.09-0.05)
6.5	-0.02	(-0.09-0.05)
8	-0.02	(-0.07-0.03)
9.5	-0.01	(-0.04-0.01)
10.5	-0.01	(-0.02-0.01)
12.5	0.00	(-0.05-0.06)
13	0.01	(-0.06-0.08)

¥ No. of studies=5
µ Midpoints were centered
**p<0.05*

➢ Overweight

Middle-income

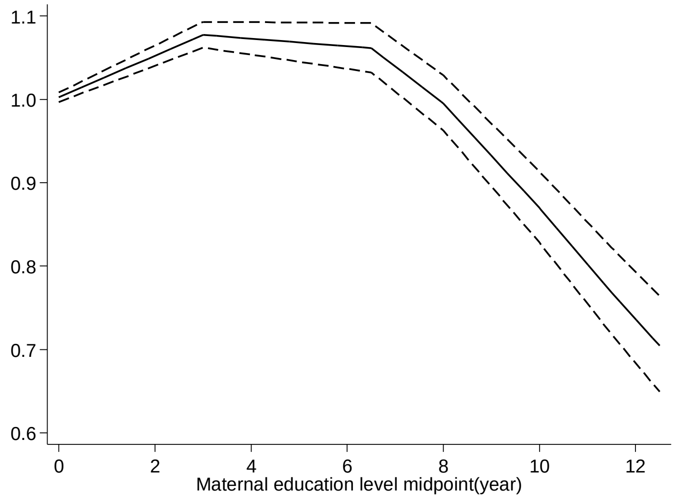

Midpoint¥µ	*exp(xb)*	*(95% CI)*
0	1.00	(1.00-1.01)*
3	1.08	(1.06-1.09)*
6.5	1.06	(1.03-1.09)*
8	1.00	(0.96-1.03)
10	0.87	(0.83-0.91)*
11.5	0.77	(0.72-0.82)*
12.5	0.70	(0.65-0.76)*

¥ No. of studies=3
µ Midpoints were centered
**p<0.05*

High-income

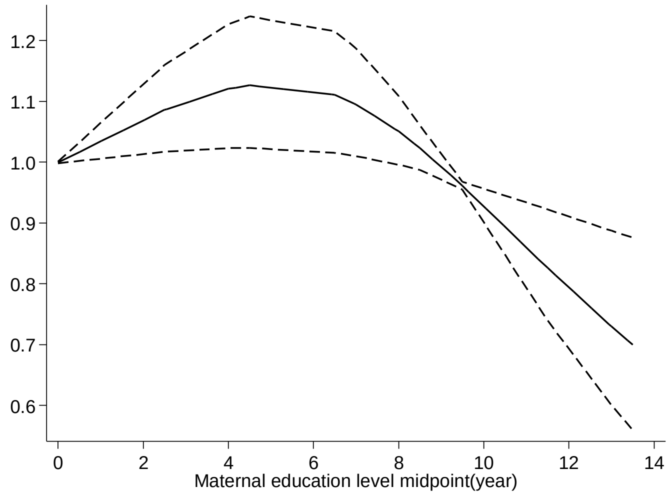

Midpoint¥µ	*exp(xb)*	*(95% CI)*
0	1.00	(1.00-1.00)*
2.5	1.09	(1.02-1.16)*
4	1.12	(1.02-1.23)*
4.5	1.13	(1.02-1.24)*
6.5	1.11	(1.02-1.22)*
7	1.10	(1.01-1.19)*
8	1.05	(1.00-1.11)*
8.5	1.02	(0.99-1.06)
9.5	0.96	(0.95-0.97)*
11.5	0.83	(0.74-0.92)*
13	0.73	(0.60-0.89)*
13.5	0.70	(0.56-0.88)*

¥ No. of studies=7
µ Midpoints were centered
**p<0.05*

Low-educated

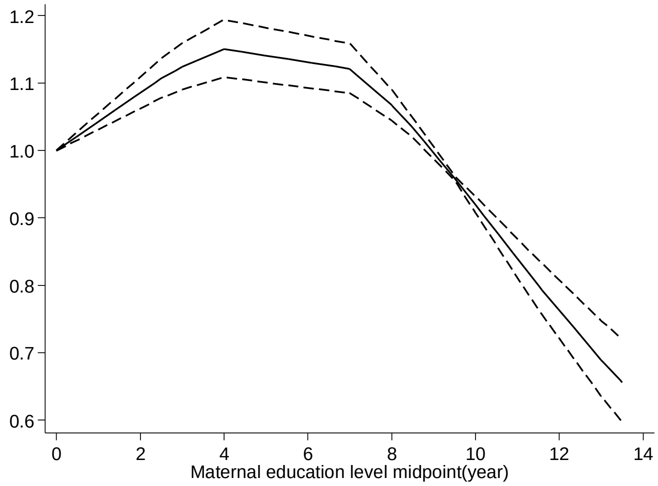

Midpoint [¥ µ]	*exp(xb)*	*(95% CI)*
0	1.00	(1.00-1.00)*
2.5	1.11	(1.08-1.14)*
3	1.12	(1.09-1.16)*
4	1.15	(1.11-1.19)*
7	1.12	(1.08-1.16)*
8	1.07	(1.04-1.09)*
8.5	1.03	(1.02-1.05)*
9.5	0.96	(0.96-0.96)*
10	0.92	(0.91-0.93)*
11.5	0.80	(0.76-0.84)*
13	0.69	(0.64-0.75)*
13.5	0.66	(0.60-0.72)*

¥ No. of studies=4
µ Midpoints were centered
**p<0.05*

High-educated

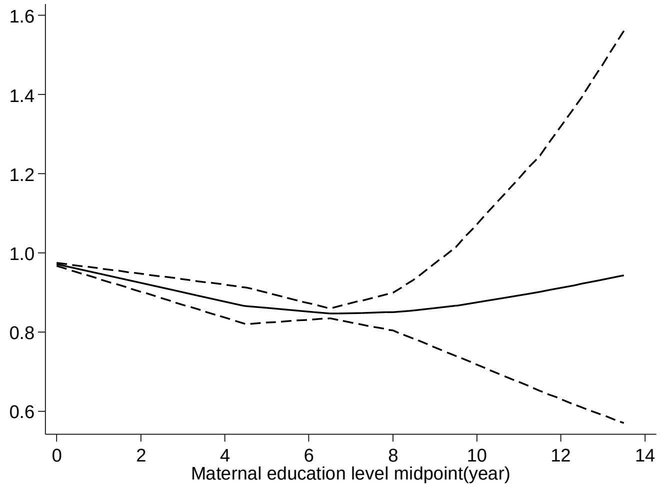

Midpoint [¥ µ]	*exp(xb)*	*(95% CI)*
0	0.97	(0.97-0.98)*
4.5	0.87	(0.82-0.91)*
6.5	0.85	(0.83-0.86)*
8	0.85	(0.80-0.90)*
8.5	0.85	(0.78-0.93)*
9.5	0.87	(0.74-1.01)
11.5	0.90	(0.65-1.25)
12.5	0.92	(0.61-1.39)
13.5	0.94	(0.57-1.56)

¥ No. of studies=6
µ Midpoints were centered
**p<0.05*

➢ Stunting

Lower middle-income

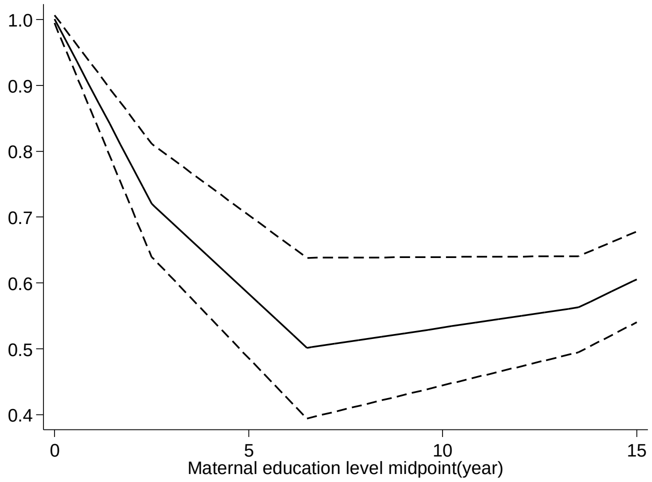

Midpoint ¥ µ	*exp(xb)*	*(95% CI)*
0	1.00	(0.99-1.01)
2.5	0.72	(0.64-0.81)*
6.5	0.50	(0.39-0.64)*
13.5	0.56	(0.49-0.64)*
15	0.61	(0.54-0.68)*

¥ No. of studies=3
µ Midpoints were centered
**p<0.05*

Upper middle-income

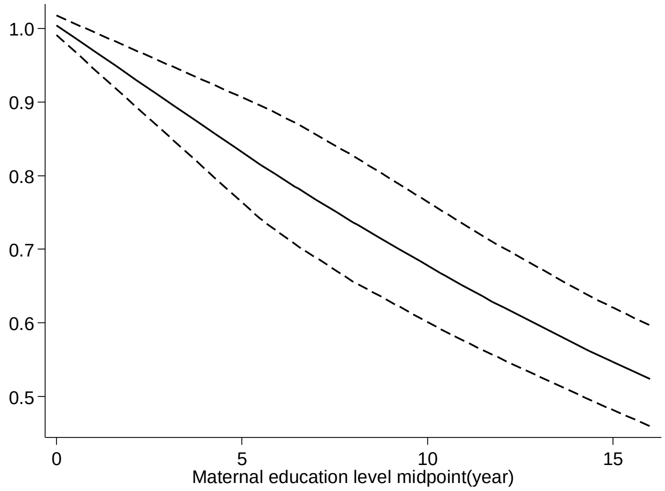

Midpoint ¥ µ	*exp(xb)*	*(95% CI)*
0	1.00	(0.99-1.02)
5.5	0.81	(0.74-0.90)*
6.5	0.78	(0.70-0.87)*
8	0.74	(0.66-0.83)*
10.5	0.66	(0.59-0.75)*
11.75	0.63	(0.56-0.71)*
14.25	0.56	(0.50-0.64)*
15	0.55	(0.48-0.62)*
16	0.52	(0.46-0.60)*

¥ No. of studies=3
µ Midpoints were centered
**p<0.05*

Low-educated

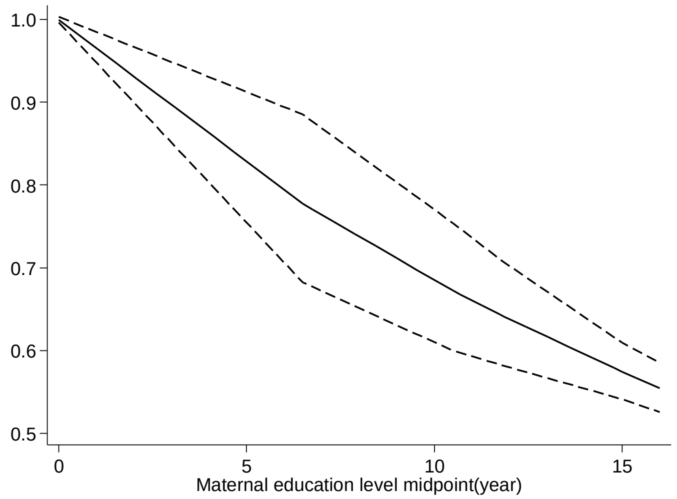

Midpoint ¥ µ	*exp(xb)*	*(95% CI)*
0	1.00	(1.00-1.00)*
6.5	0.78	(0.68-0.89)*
10.5	0.67	(0.60-0.75)*
11.75	0.64	(0.58-0.71)*
15	0.57	(0.54-0.61)*
16	0.55	(0.53-0.59)*

¥ No. of studies=4
µ Midpoints were centered
**p<0.05*

High-educated

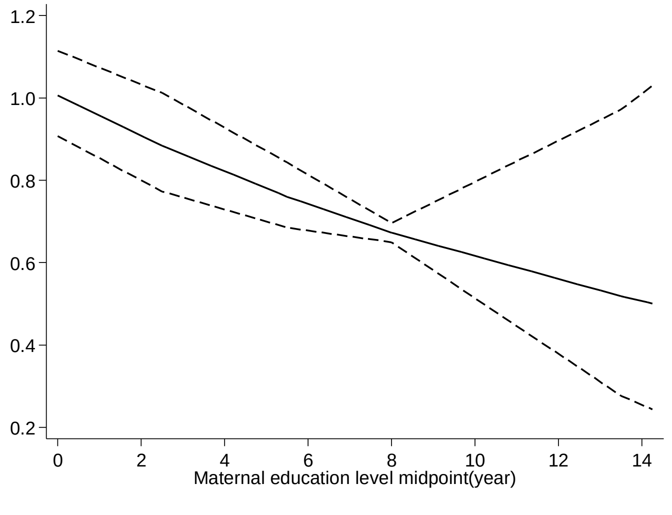

Midpoint ¥ µ	*exp(xb)*	*(95% CI)*
0	1.01	(0.91-1.11)
2.5	0.88	(0.77-1.01)
5.5	0.76	(0.68-0.84)*
8	0.67	(0.65-0.70)*
13.5	0.52	(0.28-0.97)*
14.25	0.50	(0.24-1.03)

¥ No. of studies=2
µ Midpoints were centered
**p<0.05*
