# Supplementary material for: Maternal education and its influence on child growth and nutritional status during the first two years of life: a systematic review and meta-analysis
Source: eClinicalMedicine. 2024 Apr 4;71:102574. doi: 10.1016/j.eclinm.2024.102574 (PMC11001623; doi:10.1016/j.eclinm.2024.102574)

Supplementary 3. Distribution of maternal education levels.

➢ WAS

- **Total**

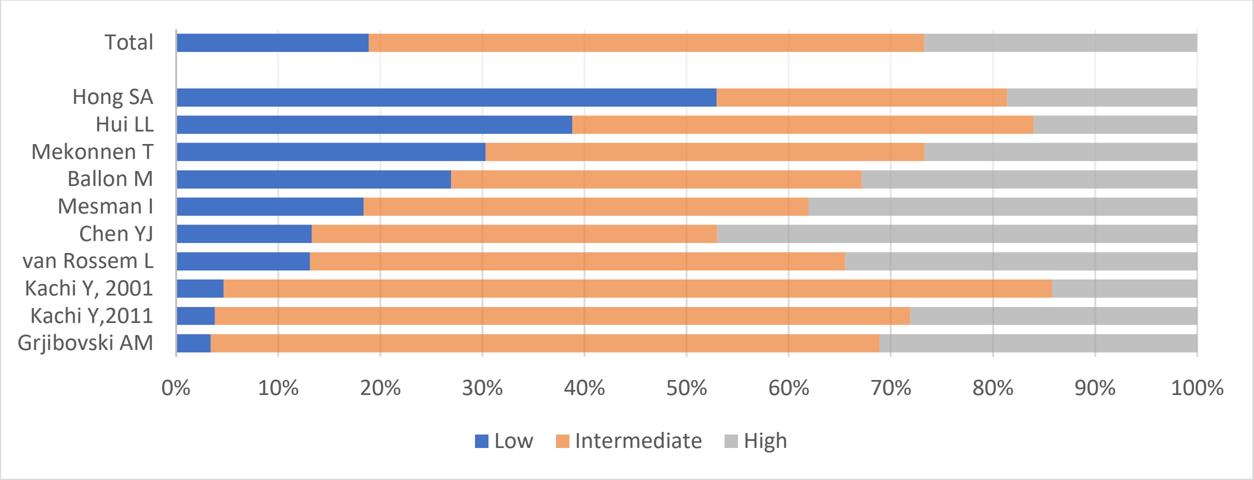

- **Crude**

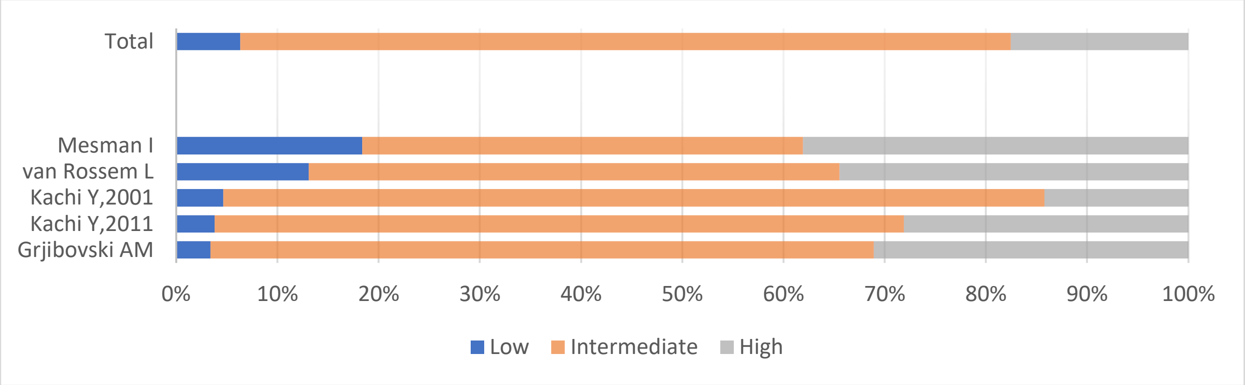

- **Adjusted**

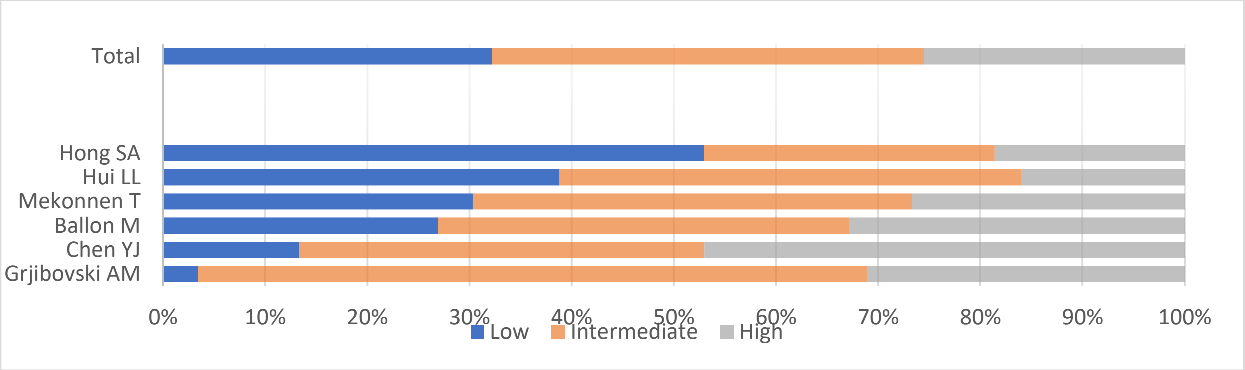

- **HAZ**

- **Total**

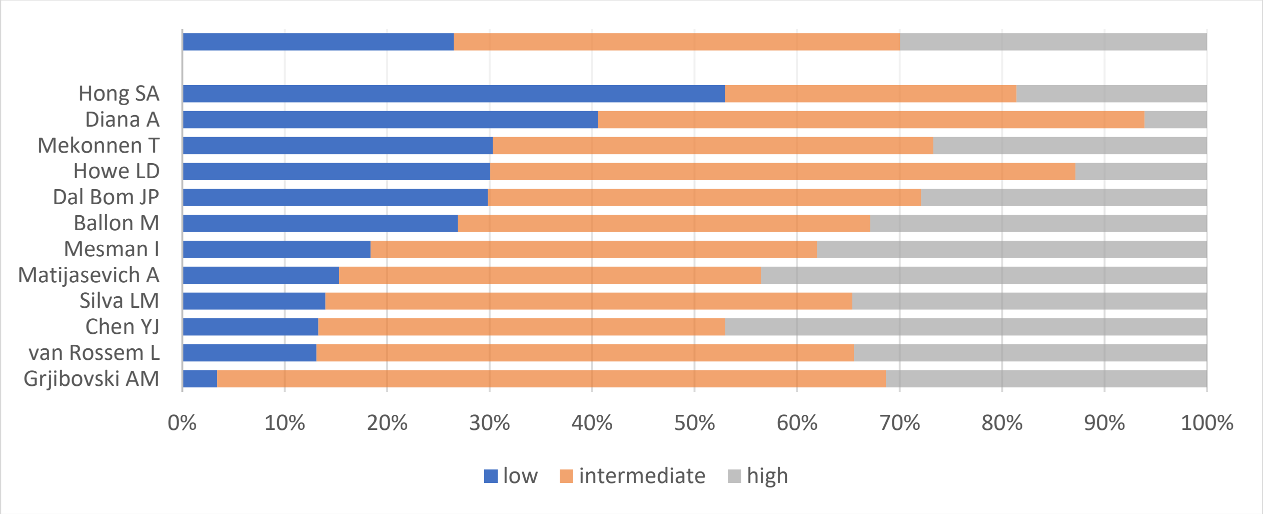

- **Crude**

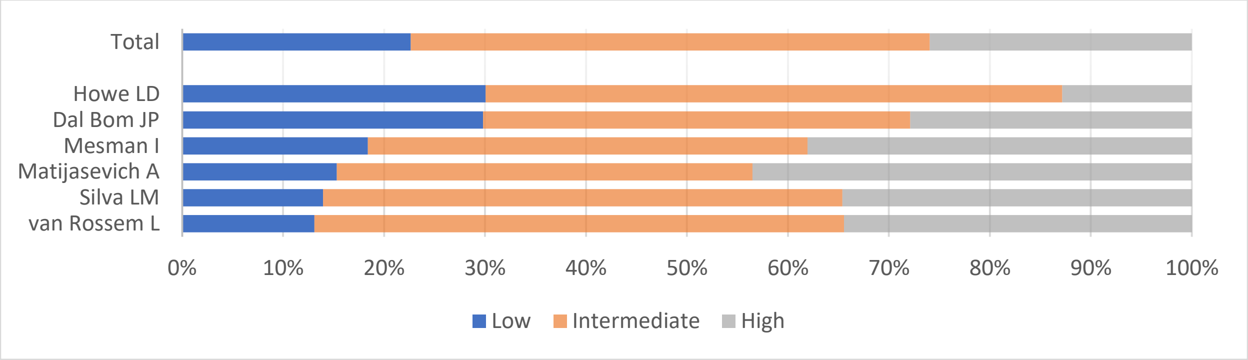

- **Adjusted**

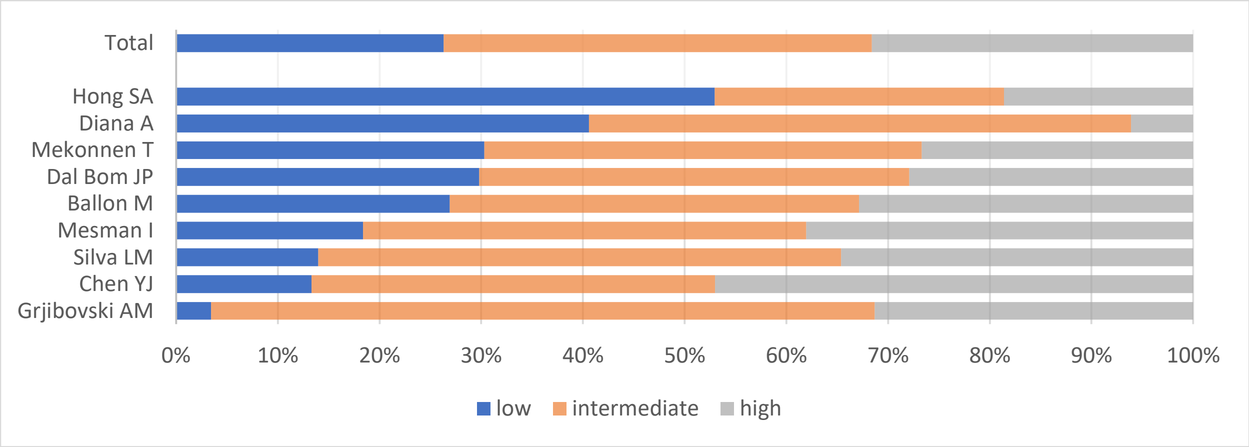

➢ BMIZ

- **Total**

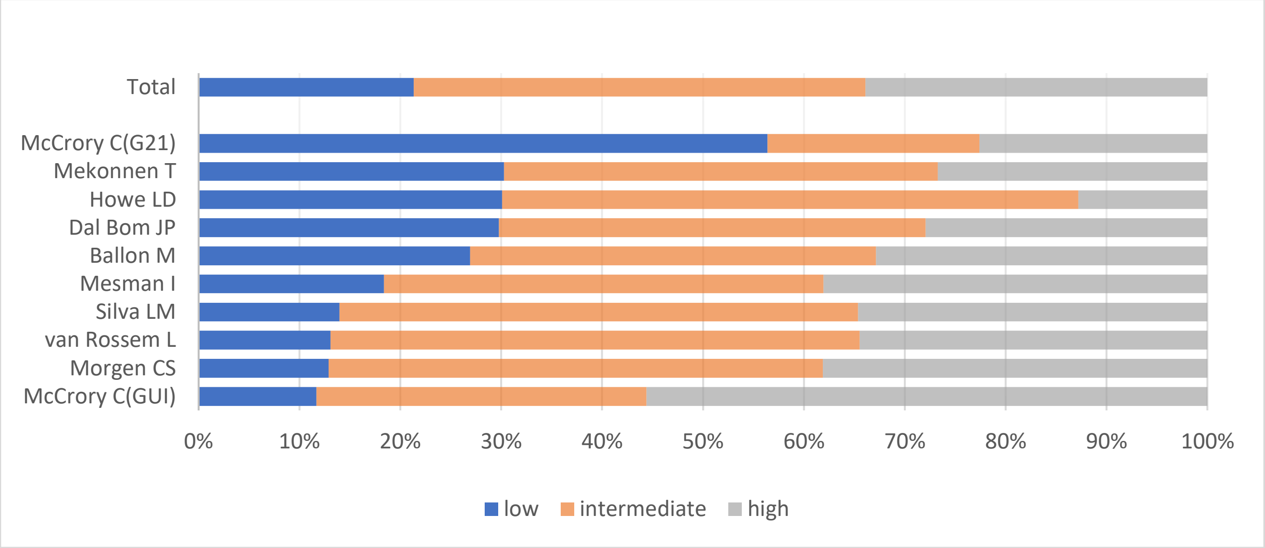

- **Crude**

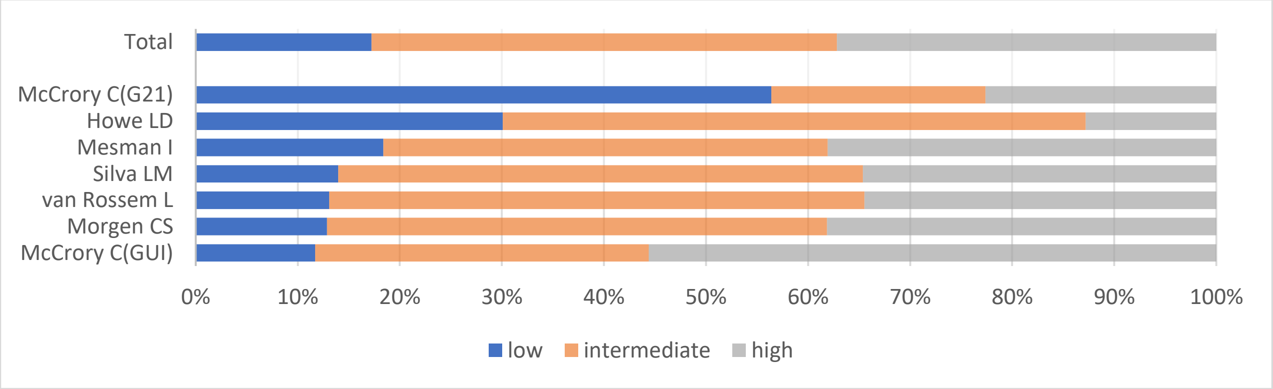

- **Adjusted**

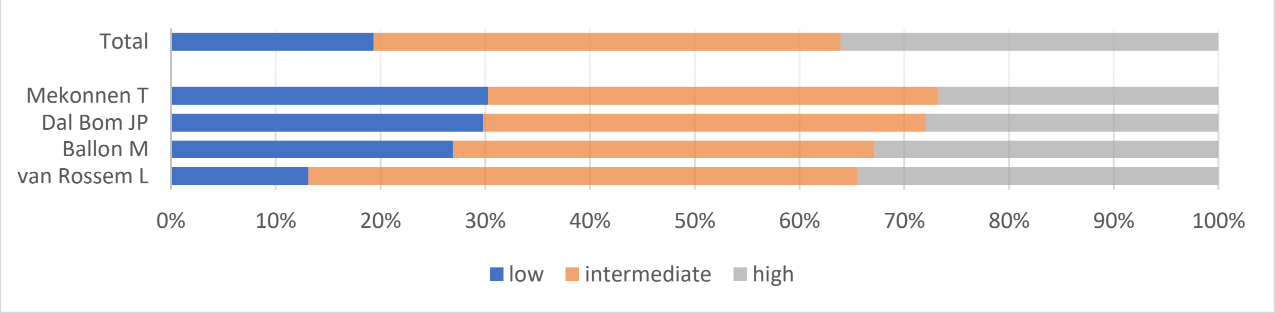

➢ Overweight

- **Total**

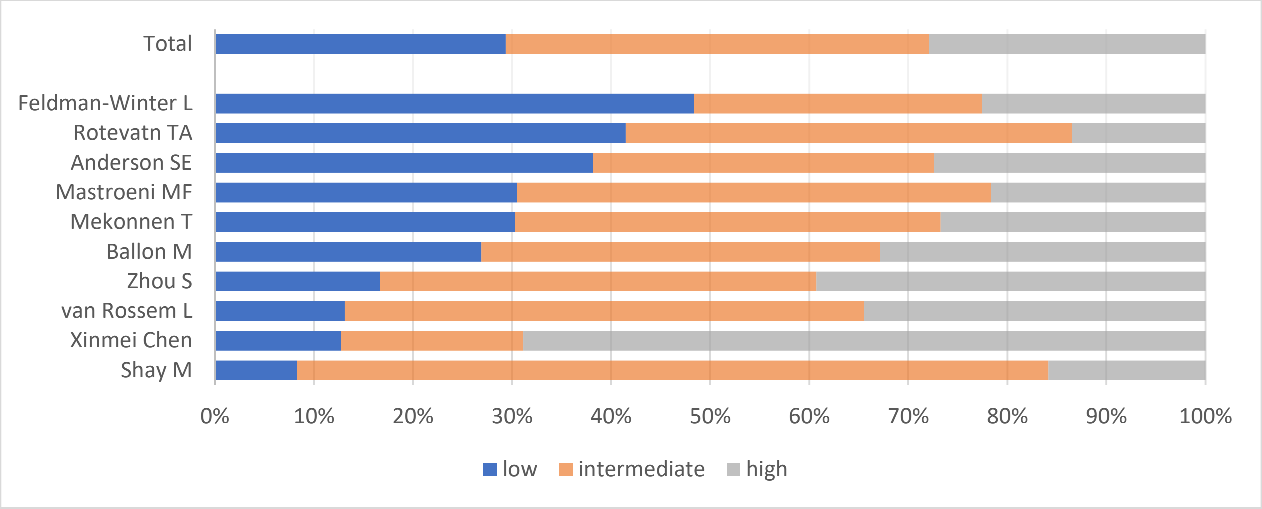

- **Crude**

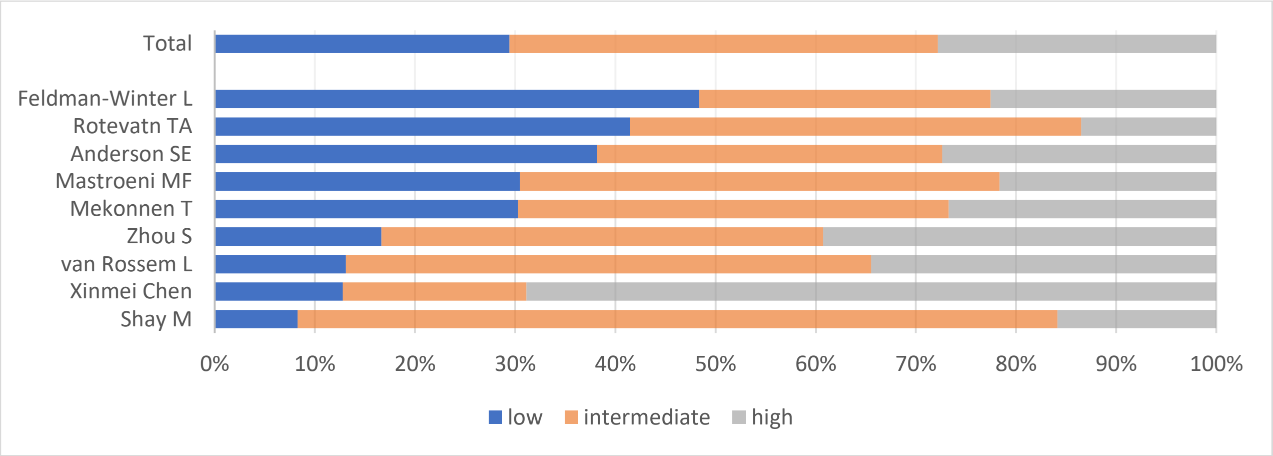

- **Adjusted**

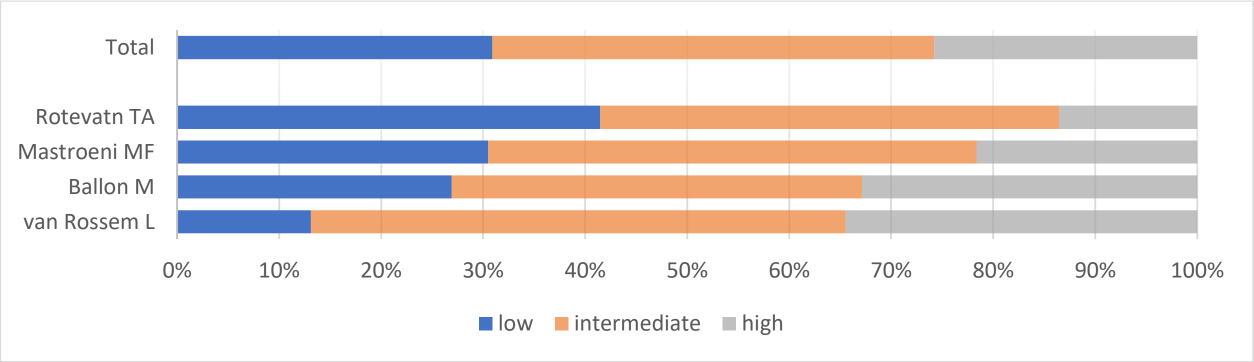

➢ Underweight*

- **Total**

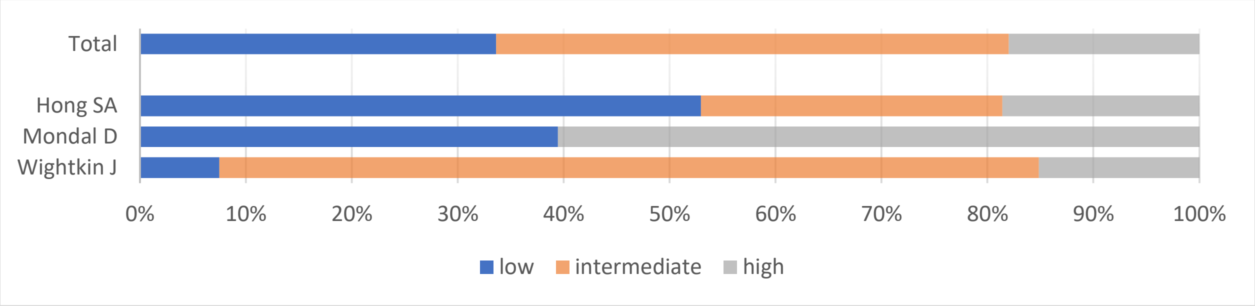

- **Crude**

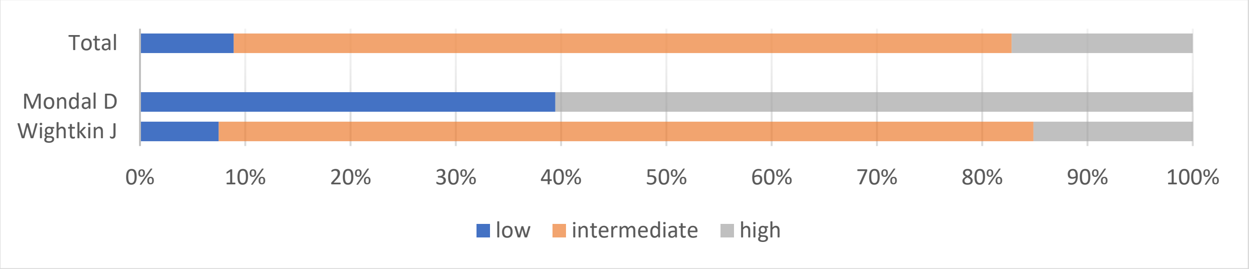

- **Adjusted**

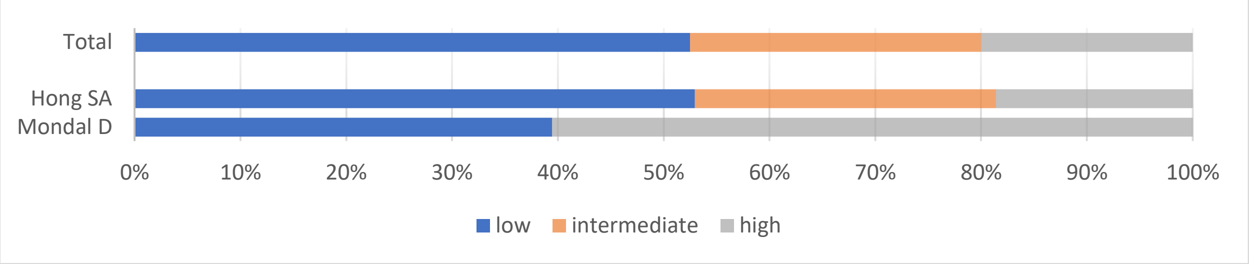

* We categorized the study by Mamabolo RL et al. as involving a low-educated population, given that the research took place in rural villages situated in the central region of Limpopo Province. These villages are marked by semi-rural environments, limited infrastructure, lack of electricity, insufficient sanitation facilities, poorly maintained roads, and under-resourced schools. Subsistence farming serves as the primary livelihood in these areas.

➢ Stunting*

- **Total**

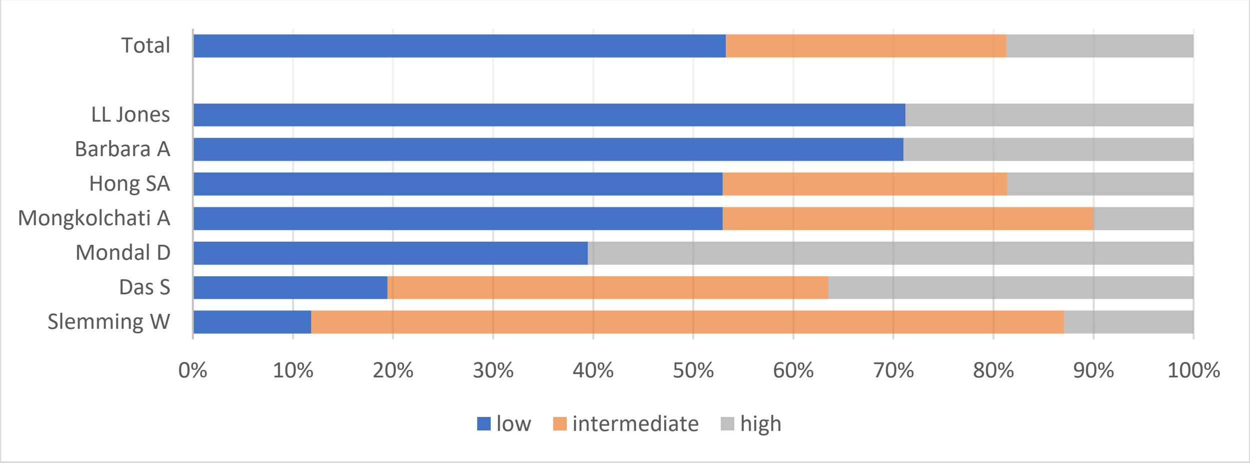

- **Crude**

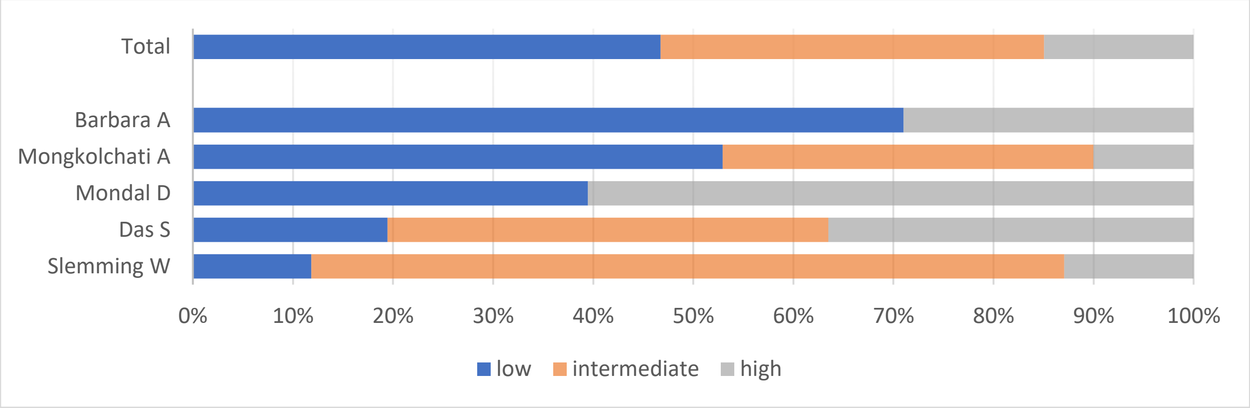

- **Adjusted**

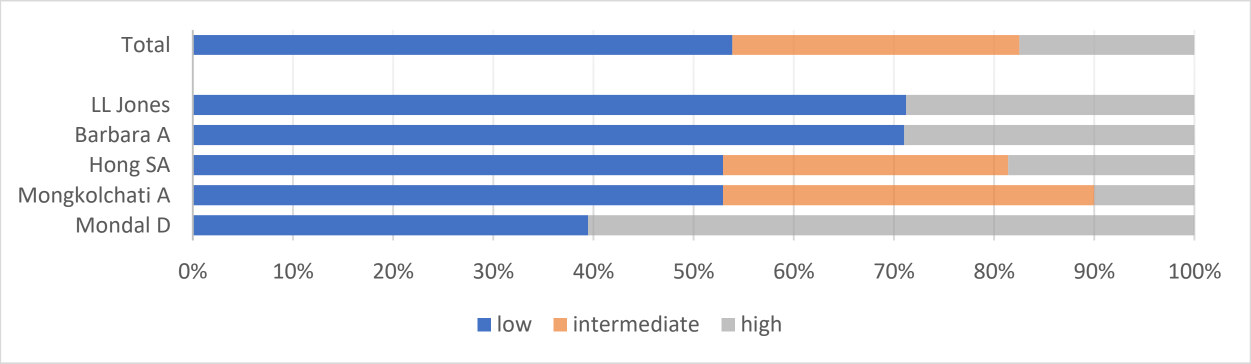

* We categorized the study by Mamabolo RL et al. as involving a low-educated population, given that the research took place in rural villages situated in the central region of Limpopo Province. These villages are marked by semi-rural environments, limited infrastructure, lack of electricity, insufficient sanitation facilities, poorly maintained roads, and under-resourced schools. Subsistence farming serves as the primary livelihood in these areas.

➢ RWG(Crude)

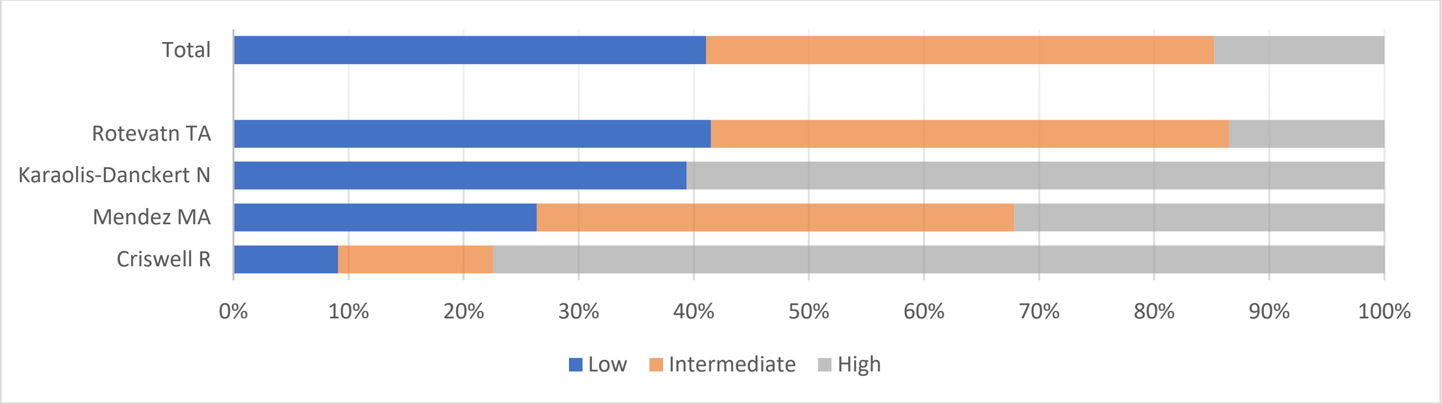
Total
Rotevatn TA
Karaolis-Danckert N
Mendez MA
Criswell R
0%
10%
20%
30%
40%
50%
60%
70%
80%
90%
100%
Low
Intermediate
High

Clustering the prevalence of low maternal education level

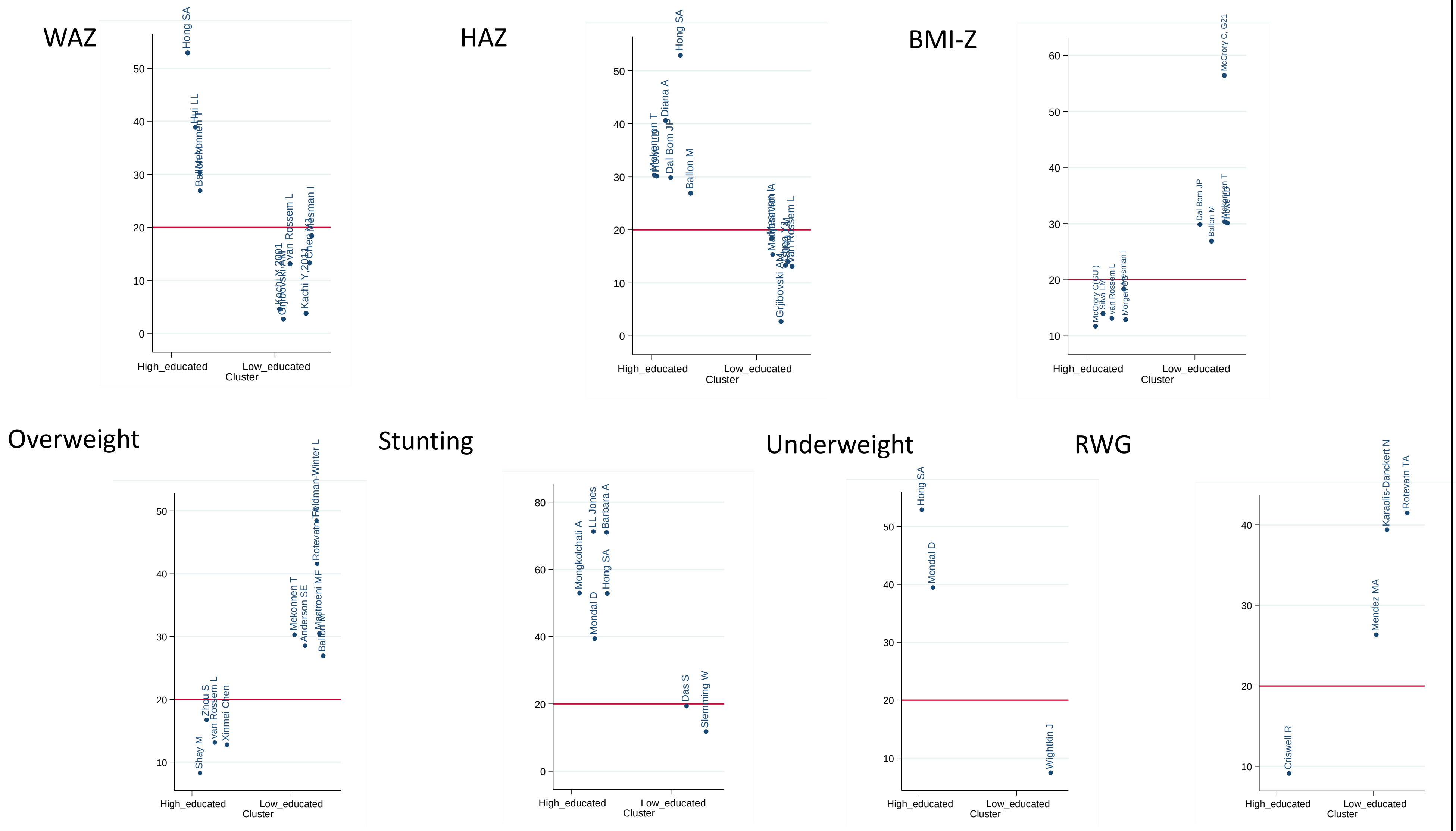

Supplement: Supplementary File 3 [file mmc3.pdf]
