# Supplementary material for: Maternal education and its influence on child growth and nutritional status during the first two years of life: a systematic review and meta-analysis
Source: eClinicalMedicine. 2024 Apr 4;71:102574. doi: 10.1016/j.eclinm.2024.102574 (PMC11001623; doi:10.1016/j.eclinm.2024.102574)
Supplement: Supplementary File 4 [file mmc4.pdf]

Supplementary 4

➢ Excluded studies.

No.	Year	Title	Journal	Author	Cohort	Healthy Children Under 2 Years Old	"Child Growth" as an outcome	"Maternal Education Status" as an exposure	First reviewer decision*			Second reviewer decision			Exclusion Reason
									I	B	E	I	B	E	
1	1991	NICHD Study of Early Child Care. Volume II: 5 Month Manual, 6 Month Manuals, Time Use Manuals.	-	National Inst. of Child Health and Human Development (NIH), Bethesda	-	+	*	*			1			1	Ineligible
2	1991	Maternal Work and Child-Care Strategies in Peri-Urban Guatemala: Nutritional Effects.	Child Development	Engle & Patrice L	-	+	+	+			1			1	Ineligible
3	1991	Long-term prospective evaluation of offspring of diabetic mothers.	Diabetes	Silverman, BL.	+	+	+	-			1			1	Ineligible
4	1991	Underlying and proximate determinants of child health: the Cebu Longitudinal Health and Nutrition Study.	American journal of epidemiology		+	+	+	-			1			1	Ineligible
5	1991	Weight gain variation in infants of an impoverished community: Bellanse, Haiti.	International journal of epidemiology	Van Oyen, HJ.	+	+	+	+	1					1	Weight gain
6	1991	A prospective study of morbidity pattern and nutritional status of a group of healthy newborns during their first year of life in a rural area near Alexandria.	The Journal of the Egyptian Public Health Association	Ahmed, HM.	+	+	+	-			1			1	Ineligible
7	1991	Socio-cultural factors influencing nutritional status of infants -- a longitudinal study.	Indian J Matern Child Health.	Hasan J.	+	+	+	+			1			1	No full text
8	1992	Maternal education in relation to early and late child health outcomes: findings from a Brazilian cohort study.	Social science & medicine (1982)	Victoria, CG.	+	+	+	+		1				1	Insufficient data
9	1992	The National Institute of Child Health and Human Development (NICHD) Study of Early Child Care: A Comprehensive Longitudinal Study of Young Children's Lives.	-	National Inst. of Child Health and Human Development (NIH)	-	+	-	-			1			1	Ineligible
10	1992	NICHD Study of Early Child Care. Volume IV: 23 Month Manual, 24 Month Manuals.	-	National Inst. of Child Health and Human Development (NIH), Bethesda	-	+	-	-			1			1	Ineligible

11	1992	Iron deficiency anemia in 1-year-old children of disadvantaged families in Montreal.	Canadian Medical Association journal	Lehmann, F.	+	+	-	+			1			1	Ineligible
12	1992	Maternal recall of infant feeding events is accurate.	Journal of epidemiology and community health	Launer, LJ.	+	+	+	-			1			1	Ineligible
13	1992	Growth and behavior development in rural infants in relation to malnutrition and environment.	Indian pediatrics	Upadhyay, SK.	+	+	+	-			1			1	Ineligible
14	1992	Growth, behavior, development and intelligence in rural children between 1-3 years of life.	Indian pediatrics	Agarwal, DK.	+	+	+	-			1			1	Ineligible
15	1992	Birth spacing and child health in urban Brazilian children.	Pediatrics	Huttly, S R;Victora, C G;Barros, F C;Vaughan, J P	+	+	+	-			1			1	Ineligible
16	1992	Observations on the development of stunting in children of the Khon Kaen region of Thailand.	European journal of clinical nutrition	Chusilp, K.	+	+	+	+			1			1	No full text
17	1992	SMOCC: Design of a representative cohort-study of live-born infants in the Netherlands	European Journal of Public Health	Herngreen, WP.	+	+	-	-			1			1	Ineligible
18	1992	Mothers' reports of their three-year-old children's control over foods and involvement in food-related activities	Journal of Nutrition Education	Anliker, J A;Laus, M J;Samonds, K W;Beal, V A	+	+	-	*			1			1	Ineligible
19	1992	EUROMAC. A European concerted action: Maternal alcohol consumption and its relation to the outcome of pregnancy and Child Development at 18 months	International Journal of Epidemiology	Du Florey, VC.	+	-	+	+			1			1	Ineligible
20	1992	A child health production function estimated from longitudinal data	Journal of Development Economics	Team, Cebu Study	+	+	-	+			1			1	Ineligible
21	1993	Mortality-discriminating power of some nutritional, sociodemographic, and diarrheal disease indices.	American journal of epidemiology	Bairagi R.	-	+	+	-			1			1	Ineligible
22	1993	Securing Our Future. Proceedings of the Conference on Children--Our Future (Kuala Lumpur, Malaysia, November 19-21, 1991).	-	Chiam Ed.	-	+	+	+			1			1	Ineligible
23	1993	Children's nutritional status in female-headed households in the Dominican Republic.	Social Science & Medicine	Johnson FC.	-	+	+	-			1			1	Ineligible
24	1993	Teenage pregnancy and child health in the urban Sahel.	Studies in family planning	LeGrand, TK.	-	+	-	+			1			1	Ineligible
25	1994	Growth and nutrition patterns of infants associated with a nutrition education and supplementation programme in Gaza, 1987-92.	Bulletin of the World Health Organization	Tulchinsky TH.	+	+	+	-			1			1	Ineligible
26	1994	Effect of prenatal marijuana exposure on the cognitive development of offspring at age three.	Neurotoxicology and Teratology	Day NL..	+	+	-	-			1			1	Ineligible
27	1994	Economic Deprivation and Early Childhood Development	Child Development	Greg J.	-	+	-	+			1			1	Ineligible
28	1994	A reexamination of the association between HOME scores and income	Nursing Research	Bradley RH.	+	+	-	-			1			1	Ineligible
29	1994	Determinants of stunting and recovery from stunting in northwest Uganda.	International journal of epidemiology	Vella, V.	+	-	+	+			1			1	Ineligible
30	1994	Growth and nutrition patterns of infants associated with a nutrition education and supplementation programme in Gaza, 1987-92	Bulletin of the World Health Organization	Tulchinsky, TH.	+	+	+	-			1			1	Ineligible

31	1995	Biochemical assessment of nutritional status in pre- and post-natal Turkish women and outcome of pregnancy	European Journal of Clinical Nutrition	Ackurt, F.	+	+	+	-			1			1	Ineligible
32	1995	Cocaine-exposed children: follow-up through 30 months.	Journal of Developmental & Behavioral Pediatrics	Hurt H.	+	+	+	-			1			1	Ineligible
33	1995	Weight growth in infants born to mothers who smoked during pregnancy.	BMJ (Clinical research ed.)	Conter, V.	+	+	+	-			1			1	Ineligible
34	1995	Undernutrition among Bedouin Arab children: a follow-up of the Bedouin Infant Feeding Study.	The American journal of clinical nutrition	Forman, MR.	+	+	+	-			1	1			Ineligible
35	1996	Determinants of growth in body length from birth to 6 years of age: A longitudinal study of Lublin children.	American journal of human biology : the official journal of the Human Biology Council	Hauspie, RC.	+	+	+	+	1			1			Insufficient data
36	1996	Evaluation of a breastfeeding-support programme with health promoters' participation	Food and Nutrition Bulletin	Alvarado MR.	*	+	+	-			1			1	Ineligible
37	1997	How Does Schooling of Mothers Improve Child Health? Evidence from Morocco. Living Standards Measurement Study Working Paper No. 128.	-	Glewwe, P.	-	*	+	+			1			1	Ineligible
38	1997	Maternal Education and Its Influences on Child Growth and Cognitive Development in Rural Guatemala.	-	Khandke, V.	-	+	-	+			1			1	Ineligible
39	1997	Mothers' Working Models of Caregiving in the Context of Infant Feeding: Change through the First Year.	-	Pridham, K.	+	+	+	-			1			1	Ineligible
40	1997	Longitudinal study of nutrient and food intakes of infants aged 2 to 24 months	Journal of the American Dietetic Association	Skinner, JD.	+	+	-	-			1			1	Ineligible
41	1997	Age at introduction of complementary food and physical growth from 2 to 9 months in rural Senegal.	European journal of clinical nutrition	Simondon, KB & Simondon, F.	+	+	+	-			1			1	Ineligible
42	1997	Outcome of very low birth weight infants at 12 to 18 months of age in Soweto, South Africa.	Pediatrics	Cooper, PA. & Sandler, DL.	+	-	+	-			1			1	Ineligible
43	1997	Postnatal growth patterns of full-term low birth weight infants in Northeast Brazi are related to socioeconomic status.	The Journal of nutrition	Ashworth, A.	+	+	+	-			1			1	Ineligible
44	1997	Maternal anthropometry and infant feeding practices in Israel in relation to growth in infancy: the North African Infant Feeding Study.	The American journal of clinical nutrition	Fawzi, WW.	+	+	+	-			1			1	Ineligible
45	1997	Does intelligence account for the link between maternal literacy and child survival?	Social science & medicine (1982)	Sandiford, P.	+	-	+	+			1			1	Ineligible
46	1997	Nutrition and growth during infancy: The Copenhagen cohort study	Acta Paediatr Suppl.	Michaelsen , KF.	+	+	+	-			1			1	Ineligible
47	1998	Socioeconomic stress, health and child nutritional status in Zimbabwe at a time of economic structural adjustment: a three year longitudinal study	-	Bijlmakers, LA.	-	-	+	+			1			1	Ineligible
48	1998	Social class differences in health until the age of seven years among the Finnish 1987 birth cohort	Social Science & Medicine	Gissler, M.	+	+	-	+			1			1	Ineligible
49	1998	Urbanization and child growth in Nepal.	American journal of human biology	Moffat, T.	-	-	+	*			1	1			Ineligible

50	1998	A longitudinal study of prolonged breastfeeding in relation to child undernutrition.	International journal of epidemiology	Fawzi, WW.	+	+	+	-			1			1	Ineligible
51	1998	Growth in height and weight of South African urban infants from birth to five years: The Birth to Ten Study.	American journal of human biology	Cameron, N.	+	+	+	-			1			1	Ineligible
52	1999	The Role of Maternal Literacy in Child Health and Cognitive Development in Rural Guatemala.	-	Khandke, V.	+	+	-	+			1			1	Ineligible
53	1999	Influence of socioeconomic conditions on growth in infancy: the 1921 Aberdeen birth cohort.	Archives of disease in childhood	Baxter-Jones, AD.	+	+	+	-			1			1	Ineligible
54	1999	Growth of children according to maternal and child HIV, immunological and disease characteristics: a prospective cohort study in Kinshasa, Democratic Republic of Congo.	International journal of epidemiology	Bailey, RC.	+	+	+	-			1			1	Ineligible
55	1999	Influence of the home environment on the development of obesity in children.	Pediatrics	Strauss, RS. & Knight, J.	+	-	+	+			1			1	Ineligible
56	1999	[Growth of breastfed and bottle-fed infants up to 2 years of age: CLACYD (Lactation, Alimentation, Growth and Development) study 1993-1995].	Rev Panam Salud Publica	Agrelo, F. J	+	+	+	-			1			1	Ineligible
57	2000	Premature complementary feeding is associated with poorer growth of vietnamese children.	The Journal of nutrition	Hop, LT.	+	+	+	-			1			1	Ineligible
58	2000	Parental risk factors for infant obesity.	The American journal of maternal child nursing	Sowan, NA. & Stember, ML.	+	+	+	-			1			1	Ineligible
59	2000	Modeling the effects of maternal nutritional status and socioeconomic variables on the anthropometric and psychological indicators of Kenyan infants from age 0-6 months	Am J Phys Anthropol	Bhargava, A.	+	+	+	-			1			1	Ineligible
60	2000	Risk factors for stunting and wasting at age six, twelve and twenty-four months for squatter children of Karachi, Pakistan.	The Journal of the Pakistan Medical Association	Fikree, FF.	+	+	+	+	1			1			Insufficient data
61	2000	Sociodemographic characteristics, care, feeding practices, and growth of cohorts of children born to HIV-1 seropositive and seronegative mothers in Nairobi, Kenya.	Tropical medicine & international health	Sherry, B.	+	+	+	-			1			1	Ineligible
62	2000	Predictors of growth from 1 to 18 months among breast-fed Ghanaian infants	European Journal of Clinical Nutrition	Lartey, A.	+	+	+	+	1			1			Continuous Education
63	2001	Longevity determined by paternal ancestors' nutrition during their slow growth period.	Acta biotheoretica	Bygren, LO.	+	-	+	-			1			1	Ineligible
64	2001	Full breast-feeding for at least four months has differential effects on growth before and after six months of age among children in a Mexican community.	The Journal of nutrition	Eckhardt, CL.	+	+	+	-			1	1			Ineligible
65	2001	Ethnicity and infant health in Southern Brazil. A birth cohort study.	International journal of epidemiology	Barros, FC.	+	+	+	-			1			1	Ineligible
66	2001	Breast-feeding is associated with improved growth in length, but not weight, in rural Senegalese toddlers.	The American journal of clinical nutrition	Simondon, KB.	+	+	+	-			1			1	Ineligible
67	2001	Breastfeeding and complementary feeding practices in rural Malawi.	Acta paediatrica	Vaahtera, MP.	+	+	*	-			1			1	Ineligible
68	2001	Inequalities in the health of newborns in the Czech Republic: Birth cohort 1994-1998	Acta Universitatis Carolinae, Geographica	Dzúrová, D.	+	-	-	+			1		1		Ineligible

69	2002	Livelihoods, nutrition and health in Dhaka slums	Public Health Nutrition	Pryer, JA.	-	-	+	+			1			1	Ineligible
70	2002	The treatment of parental height as a biological factor in studies of birth weight and childhood growth	Archives of disease in childhood	Spencer, NJ. & Logan, S.	+	-	+	-			1			1	Ineligible
71	2002	Weight and weight gain at 4 months (The Netherlands 1998): influences of nutritional practices, socio-economic and ethnic factors.	Paediatric and perinatal epidemiology	Bulk-Bunschoten AM.	-	+	+	+			1	1			Ineligible
72	2002	Determinants of linear growth and predictors of severe stunting during infancy in rural Malawi.	Acta paediatrica (Oslo, Norway : 1992)	Espo, M.	+	+	+	-			1			1	Ineligible
73	2003	Effects of low income on infant health	Canadian Medical Association Journal	Seguin, L.	+	-	-	+			1			1	Ineligible
74	2003	Social inequalities in infant feeding during the first year of life. The Longitudinal Study of Child Development in Québec (LSCDQ 1998-2002).	Public health nutrition	Dubois, L. & Girard, M.	+	+	-	+			1		1		Ineligible
75	2003	Maternal employment and overweight children.	Journal of health economics	Anderson, PM.	+	-	+	+			1			1	Ineligible
76	2003	Longitudinal growth patterns of Pakistani infants in a clinic based growth promotion program.	Indian pediatrics	Qazi, Sh. A.	+	+	+	-			1			1	Ineligible
77	2003	Growth of very low birth weight infants to age 20 years.	Pediatrics	Hack, M.	+	+	+	-			1			1	Ineligible
78	2003	Postnatal depression and infant growth and development in low income countries: a cohort study from Goa, India.	Archives of disease in childhood	Patel, V.	+	+	+	-			1	1			Ineligible
79	2003	Obesity from cradle to grave.	International journal of obesity	Eriksson, J.	+	+	+	-			1			1	Ineligible
80	2003	The Influence of Nutritional and Genetic Factors on Growth and BMI until 5 Years of Age	Monatsschrift fur Kinderheilkunde	Haschke, F. & Van't Hof, MA.	+	+	+	+	1			1			Continuous Education
81	2004	Failure to Thrive in the Term and Preterm Infants of Mothers Depressed in the Postnatal Period: A Population-Based Birth Cohort Study	Journal of Child Psychology and Psychiatry	Drewett, R.	+	+	+	-			1			1	Ineligible
82	2004	The WHO Multicentre Growth Reference Study: planning, study design, and methodology.	Food and nutrition bulletin	de Onis, M.	-	+	+	+			1			1	Ineligible
83	2004	Human immunodeficiency virus infection, diarrheal disease and sociodemographic predictors of child growth.	Acta paediatrica (Oslo, Norway: 1992)	Villamor, E.	-	+	+	+			1	1			Ineligible
84	2004	Predictors of children's body mass index: a longitudinal study of diet and growth in children aged 2-8 y.	International journal of obesity	Skinner, J.	+	+	+	-			1			1	Ineligible
85	2004	Do early infant feeding patterns relate to breast-feeding continuation and weight gain? Data from a longitudinal cohort study.	European journal of clinical nutrition	Casiday, RE.	+	+	-	+			1			1	Ineligible
86	2004	Early environment and child-to-adult growth trajectories in the 1958 British birth cohort.	The American journal of clinical nutrition	Li, L.	+	+	+	-			1			1	Ineligible
87	2004	Predicting preschooler obesity at birth: the role of maternal obesity in early pregnancy.	Pediatrics	Whitaker, RC.	+	-	+	+			1			1	Ineligible
88	2004	Predictors of body size in the first 2 y of life: a high-risk study of human obesity.	International journal of obesity	Stunkard, AJ.	+	+	+	-			1			1	Ineligible
89	2004	Family, socioeconomic and prenatal factors associated with failure to thrive in the Avon Longitudinal Study of Parents and Children (ALSPAC).	International journal of epidemiology	Blair, PS.	+	+	+	-			1		1		Ineligible

90	2004	Impact of maternal depression on infant nutritional status and illness: a cohort study.	Archives of general psychiatry	Rahman, A.	+	+	+	+		1		1			Ineligible
91	2004	Macro shocks and micro outcomes: Child nutrition during Indonesia's crisis	Economics and Human Biology	Block, SA.	-	*	+	*			1			1	Ineligible
92	2005	Understanding the dimensions of socioeconomic status that influence toddlers' health: unique impact of lack of money for basic needs in Quebec's birth cohort	Journal of Epidemiology and Community Health	Seguin, L.	+	+	-	+			1			1	Ineligible
93	2005	A young child feeding index is not associated with either height-for-age or height velocity in rural Senegalese children	Journal of Nutrition	Ntab, B.	-	+	+	*			1			1	Ineligible
94	2005	Dynamics of early childhood overweight	pediatrics	Salsberry, PJ. & Reagan, PB.	-	-	+	+			1			1	Ineligible
95	2005	Truncal adiposity is present at birth and in early childhood in South Indian children.	Indian pediatrics	Krishnaveni , GV.	+	+	+	-			1			1	Ineligible
96	2005	The effect of breastfeeding on weight gain in infants: results of a birth cohort study.	European journal of medical research	Kalies, H.	+	+	+	-			1			1	Ineligible
97	2005	Prevalence and determinants of stunting and overweight in 3-year-old black South African children residing in the Central Region of Limpopo Province, South Africa.	Public health nutrition	Mamabolo, RL.	+	-	+	+			1			1	Ineligible
98	2005	Growth trajectories are influenced by breast-feeding and infant health in an afro-colombian community.	The Journal of nutrition	Alvarado, BE.	*	+	+	*		1		1			Continuous Education
99	2005	The importance of slow weight gain in the first 2 months in identifying children who fail to thrive	Journal of Reproductive and Infant Psychology	Drewett, R.	+	+	+	-			1			1	Ineligible
100	2006	Growth in the neonatal intensive care unit influences neurodevelopmental and growth outcomes of extremely low birth weight infants.	Pediatrics	Ehrenkranz, RA.	+	+	+	-			1			1	Ineligible
101	2006	Enrolment and baseline characteristics in the WHO Multicentre Growth Reference Study.	Acta paediatrica (Oslo, Norway : 1992). Supplement	WHO Multicentre Growth Reference Study Group	*	*	+	-			1			1	Ineligible
102	2006	The influence of maternal socioeconomic and emotional factors on infant weight gain and weight faltering (failure to thrive): data from a prospective birth cohort.	Archives of disease in childhood	Wright, C M.	+	+	+	+		1		1			Ineligible
103	2006	[Growth of full term low and adequate birth weight infants during the first two years of life].	Revista de saude publica	Eickmann, S H.	+	+	+	-			1			1	Ineligible
104	2006	A fresh look at growth assessment of infants and young children in the Czech Republic in context of international developments.	Central European journal of public health	Vignerová, J. & Lhotská, L.	*	*	+	-			1			1	Ineligible
105	2006	Breastfeeding influences on growth and health at one year of age.	Breastfeeding review	Oddy, Wendy H.	+	+	+	-			1			1	Ineligible
106	2006	Assessment of 24 years of infant growth monitoring in a rural area of Senegal (1969-1992)	Cahiers Sante	Enel, C.	*	+	+	-			1			1	Ineligible
107	2007	Duration of poverty and child health in the Quebec Longitudinal Study of Child Development: longitudinal analysis of a birth cohort.	Pediatrics	Seguin, L.	+	+	+	-			1			1	Ineligible
108	2007	Socio-economic, environmental, care, health and nutritional determinants in the aetiology of severe protein-energy malnutrition in Southern Ethiopia (Sidama) with emphasis on kwashiorkor: a one year prospective and	-	Dreschl-Bogale, S.	+	*	+	-			1			1	Ineligible

		retrospective follow-up study to find out predisposing factors													
109	2007	Immigrant generation, socioeconomic status, and economic development of countries of origin: a longitudinal study of body mass index among children	Social science & medicine	Van Hook, J.	+	*	+	-			1			1	Ineligible
110	2007	Polymorphisms in the angiotensin converting enzyme gene and growth in the first year of life.	Annals of human genetics	Hindmarsh, PC.	+	+	+	-			1			1	Ineligible
111	2007	Birth size and accelerated growth during infancy are associated with increased odds of childhood overweight in Mexican children.	Journal of the American Dietetic Association	Jones-Smith, JC.	+	+	+	-			1			1	Ineligible
112	2007	Ethnobotanical knowledge is associated with indices of child health in the Bolivian Amazon.	Proceedings of the National Academy of Sciences of the United States of America	McDade, T W.	+	*	+	-			1			1	Ineligible
113	2007	Size at birth and growth trajectories to young adulthood.	American journal of human biology	Adair, LS	+	+	*	-			1		1		Ineligible
114	2007	Complementary feeding practices in preterm infants: an observational study in a cohort of Italian infants.	Journal of pediatric gastroenterology and nutrition	Fanaro, S.	+	+	-	+			1			1	Ineligible
115	2008	Schooling C, Cowling B, Leung S, Lam T, et al (2008) Are universal standards for optimal infant growth appropriate? Evidence from a Hong Kong Chinese birth cohort	Arch Dis Child	Hui, L.	+	+	+	-			1	1			Ineligible
116	2008	Correlation of 4-month infant feeding modes with their growth and iron status in Beijing	Chinese Medical Journal	Yu-Hua, G.	+	+	+	-			1			1	Ineligible
117	2008	Maternal-child health in Pelotas, Rio Grande do Sul State, Brazil: major conclusions from comparisons of the 1982, 1993, and 2004 birth cohorts.	Cadernos de saude publica	Barros, FC. & Victora, CG.	+	*	-	-			1			1	Ineligible
118	2008	Maternal labour supply and childhood obesity in Canada: Evidence from the NLSCY	Canadian Journal of Economics	Chia, YF.	-	*	+	*			1			1	Ineligible
119	2008	Infant malnutrition and obesity in three population-based birth cohort studies in Southern Brazil: Trends and differences	Cadernos de Saude Publica	Barros, AJD.	+	+	+	-			1			1	Ineligible
120	2008	Does son preference influence children's growth in height? A comparative study of Chinese and Filipino children	Population Studies	Song, S.	-	*	+	*			1			1	Ineligible
121	2008	Birth weight, infant growth, and childhood body mass index: Hong Kong's children of 1997 birth cohort	Archives of Pediatrics and Adolescent Medicine	Hui, LL.	+	*	+	-			1			1	Ineligible
122	2009	Time-independent Maternal and Infant Factors and Time-dependent Infant Morbidities including HIV Infection, Contribute to Infant Growth Faltering during the First 2 Years of Life	Journal of Tropical Pediatrics	Webb, AL.	-	+	+	+		1				1	Ineligible
123	2009	Offspring consume a more obesogenic diet than mothers in response to changing socioeconomic status and urbanization in Cebu, Philippines.	The international journal of behavioral nutrition and physical activity	Kelles, A.	*	-	*	+			1	1			Ineligible
124	2009	Impact of infant feeding practices on childhood obesity.	The Journal of nutrition	Butte, NF.	*	-	+	+			1			1	Ineligible
125	2009	Food prices, access to food outlets and child weight.	Economics and human biology	Powell, LM.	*	-	+	+			1			1	Ineligible

126	2009	[WHO growth standards for infants and young children].	Archives de pediatrie	de Onis, M.	*	+	+	-			1			1	Ineligible
127	2009	Child health and the income gradient: evidence from Australia.	Journal of health economics	Khanam, R.	+	+	*	-			1			1	Ineligible
128	2009	Effect of birth weight and postnatal weight gain on body composition in early infancy: The Generation R Study.	Early human development	Holzhauer S.	+	+	+	-			1			1	Ineligible
129	2009	Maternal Behavior and Infant Weight Gain in the First Year	Journal of Nutrition Education and Behavior	Worobey J.	+	+	+	-			1			1	Ineligible
130	2009	Period-specific growth, overweight and modification by breastfeeding in the GINI and LISA birth cohorts up to age 6 years	European Journal of Epidemiology	Rzehak, P.	+	+	+	-			1			1	Ineligible
131	2009	Mothers' community participation and child health	Journal of Health and Social Behavior	Nobles, J. & Frankenberg, E.	-	*	*	*			1			1	Ineligible
132	2009	Height, wealth, and health: An overview with new data from three longitudinal studies	Economics and Human Biology	Batty, GD.	-	*	*	-			1			1	Ineligible
133	2009	Delayed lactogenesis and excess neonatal weight loss are common across ethnic and socioeconomic categories of primiparous women in Northern California	The FASEB Journal	Dewey, KG.	+	+	+	+			1			1	Insufficient data
134	2010	The early growth and development study: using the prospective adoption design to examine genotype-environment interplay. 2008.	Behavior Genetics	Leve, LD.	+	+	-	-			1			1	Ineligible
135	2010	Low Income, Mexican Mothers' Perception of Their Infants' Weight Status and Beliefs about Their Foods and Physical Activity	Child Psychiatry and Human Development	Jimenez-Cruz, A.	-	+	+	*			1			1	Ineligible
136	2010	Unexpectedly high early prevalence of anaemia in 6-month-old breast-fed infants in rural Bangladesh.	Public health nutrition	Shakur, YA.	+	+	+	-			1			1	Ineligible
137	2010	A prospective study of the effect of delivery type on neonatal weight gain pattern in exclusively breastfed neonates born in Shiraz, Iran.	International breastfeeding journal	Saki, A.	+	+	+	-			1	1			Ineligible
138	2010	Determinants of early ponderal and statural growth in full-term infants in the EDEN mother-child cohort study.	The American journal of clinical nutrition	Regnault, N.	+	+	+	-			1			1	Ineligible
139	2010	Maternal depressive symptoms not associated with reduced height in young children in a US prospective cohort study.	PLOS One	Ertel, KA.	+	+	+	-			1			1	Ineligible
140	2010	Children who recover from early stunting and children who are not stunted demonstrate similar levels of cognition.	The Journal of nutrition	Crookston, BT.	+	-	+	+			1	1			Ineligible
141	2010	Maternal postnatal depression and children's growth and behaviour during the early years of life: exploring the interaction between physical and mental health.	Archives of disease in childhood	Avan, B.	+	+	+	-			1			1	Ineligible
142	2010	Infant growth disparity in the Khanh Hoa province in Vietnam: a follow-up study.	BMC pediatrics	Vaktskjold, A.	+	*	+	-			1			1	Ineligible
143	2010	Dynamics of obesity and chronic health conditions among children and youth	Journal of the American Medical Association	Van Cleave, J.	+	-	+	+			1			1	Ineligible
144	2010	The Generation R Study: Design and cohort update 2010	European Journal of Epidemiology	Jaddoe, VWV.	-	+	+	-			1		1		Ineligible
145	2010	Weight gain in the first two years of life is an important predictor of schooling outcomes in pooled analyses from five birth cohorts from low- and middle-income countries	Journal of Nutrition	Martorell R.	-	+	+	-			1			1	Ineligible

146	2010	Feeding choices and morbidity and mortality among children born to HIV-1 infected mothers during the first 6 months of life in Bobo-Dioulasso (Burkina Faso)	Journal of Pediatric Infectious Diseases	Nacro, B.	+	+	-	-			1			1	Ineligible
147	2010	Infant growth during the first year of life and subsequent hospitalization to 8 years of age	Epidemiology	Hui, LL.	+	+	+	-			1			1	Ineligible
148	2010	Parental education and child health: Evidence from a natural experiment in taiwan	American Economic Journal: Applied Economics	Chou, SY.	+	*	-	*			1			1	Ineligible
149	2010	Growth patterns in early childhood and final attained stature: Data from five birth cohorts from low-and middle-income countries	American Journal of Human Biology	Stein, AD.	+	+	*	-			1			1	Ineligible
150	2010	Poverty and childhood undernutrition in developing countries: A multi-national cohort study	Social Science and Medicine	Petrou, S. & Kupek, E.	+	+	+	-			1			1	Ineligible
151	2010	Prolonged episodes of acute diarrhea reduce growth and increase risk of persistent diarrhea in children	Gastroenterology	Moore, SR.	+	+	+	-			1			1	Ineligible
152	2010	Efects of breastfeding and maternal smoking during pregnancy on body mas index (BMI) trajectories among children in canada	Paediatrics and Child Health	Wilk, P.	*	+	+	-						1	Ineligible
153	2010	The relation of fetal alcohol exposure to growth and body composition in Cape Town, south African children	Alcoholism: Clinical and Experimental Research	Carter, RC.	+	+	+	-			1			1	Ineligible
154	2011	Infant Nutrition and 12 and 18 Months Secure Base Behavior: An Exploratory Study	Infancy	Wachs, TD.	+	+	+	+	1					1	Continuous Education
155	2011	Racial Gaps in Early Childhood: Socio-Emotional Health, Developmental, and Educational Outcomes among African-American Boys. Report	National Center for Children in Poverty	Aratani, Y.	+	+	-	-			1			1	Ineligible
156	2011	Parental and offspring adiposity associations: Insights from the 1958 British birth cohort	Annals of Human Biology	Power, Ch.	+	-	+	+			1			1	Ineligible
157	2011	Associations between severity of obesity in childhood and adolescence, obesity onset and parental BMI: a longitudinal cohort study	International Journal of Obesity	Svensson, V.	+	-	+	*			1			1	Ineligible
158	2011	Maternal smoking during pregnancy and offspring growth in childhood: 1993 and 2004 Pelotas cohort studies.	Archives of disease in childhood	Matijasevich, A.	+	+	+	-			1			1	Ineligible
159	2011	Does maternal autonomy influence feeding practices and infant growth in rural India?	Social science & medicine (1982)	Shroff, MR.	-	+	+	+			1			1	Ineligible
160	2011	Prenatal lead exposure and weight of 0- to 5-year-old children in Mexico city.	Environmental health perspectives	Afeiche, M.	+	+	+	-			1			1	Ineligible
161	2011	[Nutritional characteristics of newborns of overweight and obese mothers].	Anales de pediatria (Barcelona, Spain : 2003)	Ayerza Casas, A.	+	+	+	-			1			1	Ineligible
162	2011	The impact of in utero exposure to diabetes on childhood body mass index growth trajectories: the EPOCH study.	The Journal of pediatrics	Crume, TL.	+	+	+	+		1				1	Special group cohort
163	2011	Socioeconomic status and weight gain in early infancy.	International journal of obesity (2005)	Wijlaars LP	+	+	+	-			1			1	Ineligible
164	2011	Expression of CYP19 and CYP17 is associated with leg length, weight, and BMI.	Obesity (Silver Spring, Md.)	Yousefi, M.	+	*	+	-			1	1			Ineligible

165	2011	Effects of prenatal and early life malnutrition: Evidence from the Greek famine	Journal of Health Economics	Neelsen, S. & Stratmann, T.	+	+	-	*			1			1	Ineligible
166	2011	Civil war, crop failure, and child stunting in Rwanda	Economic Development and Cultural Change	Akresh, R.	*	*	+	-			1			1	Ineligible
167	2011	Child care subsidies and childhood obesity	Review of Economics of the Household	Herbst, C M. & Tekin, E.	+	*	+	-			1			1	Ineligible
168	2011	Is the influence of early maternal menarche on offspring's rapid growth modified during in-utero? Finding from a population-based cohort of 57,296 child mother pairs in South China	Obesity	Min, J.	+	+	+	-			1			1	Ineligible
169	2011	Infant's growth throught the first year of life and maternal pre-pregnancy body mass index	Annals of Nutrition and Metabolism	Giovannini, M.	+	+	+	-			1			1	Ineligible
170	2011	Nutritional characteristics of infants from overweight and obese mothers	International Journal of Obesity	Rodríguez Martínez, G.	+	+	+	-			1			1	Ineligible
171	2011	Effects of prenatal exposure to modern pesticides on birth weight, growth and body composition in childhood; Interactions with maternal smoking and PON1 gene-polymorphisms	Hormone Research in Paediatrics	Wohlfahrt-Veje, C.	+	+	+	-			1			1	Ineligible
172	2011	The relationship between vitamin D, otitis media, and weight	Otolaryngology - Head and Neck Surgery	Weinreich, H. & Daly, K.	+	+	+	-			1	1			Ineligible
173	2011	Factors influencing high rates of catch up growth after early childhood stunting in children of urban slums of southern India: A cohort study	American Journal of Tropical Medicine and Hygiene	Lal, A.	+	+	+	-			1			1	Ineligible
174	2011	Introduction to the IDEFICS study - obesity prevalence in European children	International Journal of Obesity	Ahrens, W.	+	+	+	-			1			1	Ineligible
175	2011	Impact of malaria in pregnancy on gestational age, birthweight and infant growth: A cohort study in Uganda	Tropical Medicine and International Health	De Beaudrap, P.	+	+	+	+		1		1			Special group cohort
176	2011	Child care: Implications for overweight/obesity in Canadian children?	Canadian Journal of Diabetes	McLaren, L.	*	-	+	*			1			1	Ineligible
177	2011	Associations between the home and school environments and child body mass index	Social Science and Medicine	Miller, DP.	+	-	+	*			1			1	Ineligible
178	2011	The mothers and children's environmental health study (a multicenter longitudinal study in Korea)	Epidemiology	Ha, EH.	+	+	*	-			1			1	Ineligible
179	2011	Pointers to childhood obesity from a longitudinal study	Journal of Diabetes	Voss, LD.	+	*	+	-			1			1	Ineligible
180	2011	Idefics study-obesity prevalence and risk factors in European children	American Journal of Epidemiology	Ahrens, W. & Pigeot, I.	*	-	+	+			1			1	Ineligible
181	2012	Early Maternal Employment and Childhood Obesity among Economically Disadvantaged Families in the USA	Early Child Development and Care	Coley, RL.	+	-	+	*			1			1	Ineligible
182	2012	Determinants of linear growth from infancy to school-aged years: a population-based follow-up study in urban Amazonian children	BMC Public Health	Lourenco, BH.	-	+	+	+		1			1		Ineligible
183	2012	Maternal unemployment and childhood overweight: is there a relationship?	Journal of Epidemiology and Community Health	Stewart, L.	*	-	+	-			1			1	Ineligible

184	2012	Using the WHO 2006 child growth standard to assess the growth and nutritional status of rural south Indian infants	Annals of Human Biology	Johnson, W.	-	+	+	+			1			1	Ineligible
185	2012	Maternal antecedents of infants with abnormal head sizes in southwest Nigeria: A community-based study.	Journal of family & community medicine	Olusanya, BO.	-	*	+	+			1			1	Ineligible
186	2012	Postnatal Growth Patterns in a Chilean Cohort: The Role of SES and Family Environment.	International journal of pediatrics	Kang Sim, D E.	+	+	+	-			1			1	Ineligible
187	2012	Breastfeeding in HIV exposed infants significantly improves child health: a prospective study.	Maternal and child health journal	Kindra, G.	+	*	+	-			1			1	Ineligible
188	2012	Safety of tenofovir use during pregnancy: early growth outcomes in HIV-exposed uninfected infants.	AIDS (London, England)	Siberry, GK.	+	+	+	-			1			1	Ineligible
189	2012	The association between early childhood overweight and maternal factors.	Childhood obesity (Print)	Barroso, CS.	-	+	+	+			1			1	Ineligible
190	2012	Immigrant-native differences in child health: does maternal education narrow or widen the gap?	Child Development	Jackson, MI.	+	-	*	*			1			1	Ineligible
191	2012	Race/Ethnic and Nativity Disparities in Child Overweight in the United States and England.	The Annals of the American Academy of Political and Social Science	Martinson, ML.	+	-	+	+			1			1	Ineligible
192	2012	Social inequalities in height: persisting differences today depend upon height of the parents.	PLOS One	Galobardes, B.	+	+	+	+		1				1	Duplication reference population
193	2012	Maternal experiences of racial discrimination and child weight status in the first 3 years of life.	Journal of developmental origins of health and disease	Dixon, B.	+	+	+	-			1			1	Ineligible
194	2012	Growth parameters of NICU admitted low birth weight preterm neonates at corrected ages of 6 and 12 month.	Iranian journal of reproductive medicine	Islami, Z.	+	+	+	-			1			1	Ineligible
195	2012	Maternal depression and infant growth and development in British Pakistani women: a cohort study.	BMJ open	Husain, N.	+	+	+	-			1			1	Ineligible
196	2012	Physical growth during the first year of life. A longitudinal study in rural and urban areas of Hanoi, Vietnam.	BMC pediatrics	Nguyen, HT.	+	+	+	-			1	1			Ineligible
197	2012	Head circumference of infants born to mothers with different educational levels; the Generation R Study.	PLOS One	Bouthoorn, SH.	+	+	+	-			1			1	Ineligible
198	2012	A longitudinal study of food insecurity on obesity in preschool children.	Journal of the Academy of Nutrition and Dietetics	Metallinos-Katsaras, E.	+	-	+	+			1			1	Ineligible
199	2012	The geographic accessibility of child care subsidies and evidence on the impact of subsidy receipt on childhood obesity	Journal of Urban Economics	Herbst, CM. & Tekin, E.	+	*	+	-			1			1	Ineligible
200	2012	How parents' income, time and job quality affect children's health and development	Australian Journal of Social Issues	Nicholson, J M.	*	*	-	+			1			1	Ineligible
201	2012	Trends in socioeconomic inequalities in anthropometric status in a population undergoing the nutritional transition: Data from 1982, 1993 and 2004 pelotas birth cohort studies	BMC Public Health	Matijasevich, A.	+	+	+	-			1			1	Ineligible
202	2012	Maternal smoking during pregnancy and effects on neonatal anthropometry: A prospective study	Turkish Journal of Medical Sciences	Bolat, F.	+	-	+	*			1			1	Ineligible
203	2012	Wars and child health: Evidence from the Eritrean-Ethiopian conflict	Journal of Development Economics	Akresh, R.	-	*	+	*			1			1	Ineligible

204	2012	Elevated insulin concentrations at birth and at prepubescent age are associated with an altered BMI course during childhood-results of the ulm birth cohort study (UBCS)	Hormone Research in Paediatrics	Brandt, S.	+	*	+	-			1			1	Ineligible
205	2012	Lessons learned from a secular trend of diarrheal diseases, risk factors and nutritional status in cohort studies of children in northeast of Brazil	American Journal of Tropical Medicine and Hygiene	Lima, AA.	+	*	+	-			1			1	Ineligible
206	2012	Brief introduction of the collaboration work process and introduction and findings of birth cohort study from Moceh, Korea	Epidemiology	Ha, EH.	+	+	+	-			1			1	Ineligible
207	2012	Socioeconomic disparities in trajectories of overweight in infancy and early childhood	Obesity Facts	Schmidt Morgen, C.	+	-	+	+			1			1	Ineligible
208	2012	Rheumatoid arthritis disease activity during pregnancy affects the postnatal catch-up growth of the child	Arthritis and Rheumatism	de Steenwinke l FD	+	+	+	-			1			1	Ineligible
209	2012	Mercury levels in maternal and cord blood and attained weight through the 24months of life	Epidemiology	Kim, BM.	+	+	+	-			1			1	Ineligible
210	2012	A fetal vocs exposure related to postnatal growth and neurobehavioral development in mothers and children's environmental health (MOCEH) study	Epidemiology	Chang, M..	+	+	+	-			1			1	Ineligible
211	2012	Bmi trajectories in minnesota: An analysis of a 35-year birth cohort	American Journal of Epidemiology	Andrade, KE.	+	-	+	+			1			1	Ineligible
212	2012	Maternal depression and child BMI: Longitudinal findings from a US sample	Pediatric Obesity	Duarte, CS.	*	*	+	-			1			1	Ineligible
213	2012	Health of children born to older mothers in the uk	Archives of Disease in Childhood	Sutcliffe, A.	+	+	+	-			1			1	Ineligible
214	2012	Sociodemographic factors as determinants of body mass index trajectories in childhood	Obesity Facts	Kautiainen, S.	+	+	+	+			1			1	Insufficient data
215	2012	Maternal Height and child growth: Intergenerational perspectives	FASEB Journal	Addo, OY.	+	+	+	-			1			1	Ineligible
216	2013	Low maternal education is associated with increased growth velocity in the first year of life and in early childhood: the ABCD study	European Journal of Pediatrics	den Berg, G.	+	+	+	+	1				1		Weight gain
217	2013	Family Structure and Obesity among U.S. Children	Journal of Applied Research on Children	Augustine, JM. & Kimbro, RT.	+	-	+	+			1			1	Ineligible
218	2013	Socioeconomic status and trajectory of obesity from birth childhood: The early childhood longitudinal study-birth cohort.	American Journal of Epidemiology	Jones-Smith, J.	+	+	+	-			1			1	Ineligible
219	2013	Stronger influence of maternal than paternal obesity on infant and early childhood body mass index: the Fels Longitudinal Study	Pediatric Obesity	Linabery, AM.	+	+	+	-			1			1	Ineligible
220	2013	Child and Adolescent Obesity and Employment Sector in Urban China	Asian Population Studies	Li, Y.	*	*	+	-			1			1	Ineligible
221	2013	Dollars and pounds: the impact of family income on childhood weight	Applied Economics	Chia, YF.	*	-	+	+			1			1	Ineligible
222	2013	Breastfeeding duration, age of starting solids and high BMI risk and adiposity in Indian children.	Maternal & child nutrition	Caleyachett y, A.	+	*	+	-			1			1	Ineligible
223	2013	Timing of solid food introduction and obesity: Hong Kong's "children of 1997" birth cohort.	Pediatrics	Lin, Sh. L.	+	+	+	-			1			1	Ineligible

224	2013	Periods of child growth up to age 8 years in Ethiopia, India, Peru and Vietnam: key distal household and community factors.	Social science & medicine (1982)	Schott, WB.	+	+	-	*		1				1	Ineligible
225	2013	Relationship of exclusive breast feeding for 6 mo to linear growth up to 18 mo of age.	Indian journal of pediatrics	Agarwal, KN.	+	+	+	-			1			1	Ineligible
226	2013	The critical period of infant feeding for the development of early disparities in obesity.	Social science & medicine (1982)	Thompson, AL.	+	+	+	-			1			1	Ineligible
227	2013	Infant feeding practices and children's weight status.	Canadian journal of dietetic practice and research	Rossiter, MD. & Evers, SE.	+	+	-	+			1			1	Ineligible
228	2013	Factors associated with morbidities among infants in three sub centre areas of belgaum district of South India: a longitudinal study.	Indian journal of community medicine	Joseph N.	+	+	+	-			1			1	Ineligible
229	2013	Maternal HIV infection and other factors associated with growth outcomes of HIV-uninfected infants in Entebbe, Uganda.	Public health nutrition	Muhangi L.	-	+	+	+		1				1	Ineligible
230	2013	Child Health, Maternal Marital and Socioeconomic Factors, and Maternal Health.	Journal of family issues	Garbarski, D. & Witt, WP.	+	*	-	*			1	1			Ineligible
231	2013	Trajectories of socioeconomic inequalities in health, behaviours and academic achievement across childhood and adolescence.	Journal of epidemiology and community health	Howe, LD.	+	-	+	+			1	1			Ineligible
232	2013	Grandparental education, parental education and child height: evidence from Hong Kong's "Children of 1997" birth cohort.	Annals of epidemiology	Kwok, MK.	+	+	-	+			1			1	Ineligible
233	2013	Trajectories of BMI from early childhood through early adolescence: SES and psychosocial predictors.	British journal of health psychology	Lane, SP.	+	+	+	-			1			1	Ineligible
234	2013	Critical windows for nutritional interventions against stunting	American Journal of Clinical Nutrition	Prentice, AM.	-	+	+	*			1			1	Ineligible
235	2013	Factors predicting severe childhood obesity in kindergarteners	International Journal of Obesity	Flores, G. & Lin, H.	+	+	+	-			1			1	Ineligible
236	2013	The effect of breastfeeding in body composition of young children	Journal of Human Growth and Development	Corona, LP. & Conde, WL.	-	+	+	*			1			1	Ineligible
237	2013	Unhealthy conditions? A longitudinal analysis of the health of children in one- and two-parent households	Comparative Population Studies	Brockmann, H.	*	*	-	*			1			1	Ineligible
238	2013	Determinants of severe malnutrition of children and obstacles of its management at ouagadougou (Burkina faso)	Annals of Nutrition and Metabolism	Compaore, EWR.	+	+	+	-			1			1	Ineligible
239	2013	Infant and young child feeding practices in rural India: A community based formative research	FASEB Journal	Anwar, F.	+	+	+	-			1			1	Ineligible
240	2013	Breast milk adiponectin is associated with maternal dietary intake and infant growth	Annals of Nutrition and Metabolism	Jan Mohamed, H J.	+	+	+	-			1			1	Ineligible
241	2013	Family sociodemographic factors at birth as determinants of body mass index development of the offspring until 7 years of age	European Journal of Epidemiology	Lehtinen-Jacks, S.	+	+	+	-			1			1	Ineligible
242	2013	Fecundability in women born preterm	American Journal of Epidemiology	Wildenschild, C.	*	*	*	-			1			1	Ineligible
243	2013	When obesity begins: Anthropometric and demographic characteristics of California WIC child participants	FASEB Journal	Papathakis, PC.	+	+	+	-			1			1	Ineligible

244	2013	Impact of assisted reproduction techniques (ART) on the health of newborns. Preliminary results of the spanish group of assisted reproduction tecniques and the health of newborn group (trasarn)	Journal of Neonatal-Perinatal Medicine	López-Azorín, M.	+	+	*	-			1			1	Ineligible
245	2013	Parental feeding practices and their association with growth trajectories in a Portuguese Birth Cohort during the first years of life	European Journal of Epidemiology	Severo, M.	+	+	+	-			1			1	Ineligible
246	2013	Unpredicted growth from ages 1 to 5 and 5 to 8 years in Ethiopia, India, Peru and Vietnam: Key distal household and community factors	FASEB Journal	Crookston, BT.	+	+	+	-			1			1	Ineligible
247	2013	Relapses from acute malnutrition in a community-based management program in burkina-faso	Annals of Nutrition and Metabolism	Somasse, YE.	+	*	+	-			1			1	Ineligible
248	2013	Impact ofmaternal depressive symptoms and infant temperament on early infant growth and motor development: Results from a population based study in bangladesh	Archives of Women's Mental Health	Nasreen, HE.	+	+	+	+	1					1	Continuous Education
249	2013	Hospital support for malnourished children	Annals of Nutrition and Metabolism	Ousmane, O.	*	*	+	-			1			1	Ineligible
250	2013	Adiposity of healthy, full-term breast-fed and formula-fed infants: A prospective cohort study	Archives of Disease in Childhood: Education and Practice Edition	Gale, C.	+	+	+	-			1			1	Ineligible
251	2013	Impact of perinatal different intrauterine environments on child growth and development in the first six months of life-ivapsa birth cohort	Annals of Nutrition and Metabolism	Bernardi JR.	+	+	*	-			1			1	Ineligible
252	2013	Trajectories and predictors of developmental skills in healthy twins up to 24 months of age	Infant Behavior & Development	Nan, C.	+	*	+	-			1			1	Ineligible
253	2013	Intergenerational and socioeconomic gradients of child obesity	Social Science & Medicine	Costa-Font, J.	-	-	+	+			1			1	Ineligible
254	2013	Influence of feeding, parental anthropometry, socio-cultural and perinatal aspects on infant body size in spain	Annals of Nutrition and Metabolism	Oves, B.	+	+	+	+			1			1	Insufficient data
255	2013	Maternal smoking and the impact on infant weight at 9 months	Journal of Women's Health	Braid, S.	+	+	+	-			1			1	Ineligible
256	2013	Child's sleep problems and risk of childhood overweight: A longitudinal study	American Journal of Epidemiology	Wang, L. & Alamian, A.	+	+	+	-			1			1	Ineligible
257	2014	Association between socioeconomic household level and z-score of BMI in children: results of a longitudinal study (624.14)	The FASEB Journal	Liria, M.	+	-	+	+			1			1	Ineligible
258	2014	Socioeconomic status and trajectory of overweight from birth to mid-childhood: the early childhood longitudinal study-birth cohort	PLOS One	Jones-Smith, JC.	+	+	+	-			1			1	Ineligible
259	2014	Breastfeeding offers protection against obesity in children of recently immigrated Latina women.	Journal of community health	Verstraete, SG.	+	-	+	+			1			1	Ineligible
260	2014	Maternal HIV is associated with reduced growth in the first year of life among infants in the Eastern region of Ghana: the Research to Improve Infant Nutrition and Growth (RIING) Project.	Maternal & child nutrition	Lartey, A.	+	+	+	-			1			1	Ineligible
261	2014	Diet and growth in infancy: relationship to socioeconomic background and to health and development in the Avon Longitudinal Study of Parents and Children.	Nutrition reviews	Emmett, PM. & Jones, LR.	+	+	+	-			1			1	Ineligible
262	2014	Socioeconomic differences in childhood length/height trajectories in a middle-income country: a cohort study.	BMC public health	Patel, R.	-	+	+	+		1				1	Ineligible

263	2014	Socioeconomic status, infant feeding practices and early childhood obesity.	Pediatric obesity	Gibbs, BG. & Forste, R.	+	+	+	-			1			1	Ineligible
264	2014	Effect of tobacco smoke exposure during pregnancy and preschool age on growth from birth to adolescence: a cohort study.	BMC pediatrics	Muraro, AP.	+	-	+	+			1			1	Ineligible
265	2014	Determinants of infant formula use and relation with growth in the first 4 months.	Maternal & child nutrition	Betoko A.	+	+	+	-			1			1	Ineligible
266	2014	Neighbourhood deprivation, individual-level familial and socio-demographic factors and diagnosed childhood obesity: a nationwide multilevel study from Sweden.	Obesity facts	Li, X.	+	-	+	+			1			1	Ineligible
267	2014	PREVALENCE OF ACUTE MALNUTRITION IN PRE-SCHOOL CHILDREN IN A RURAL AREA OF NORTHERN SUDAN.	East African medical journal	Mohamed, S. & Hussein, MD.	-	*	+	*			1			1	Ineligible
268	2014	The MAL-ED cohort study in Mirpur, Bangladesh.	Clinical infectious diseases	Ahmed T.	-	*	+	-			1			1	Ineligible
269	2014	How resource dynamics explain accumulating developmental and health disparities for teen parents' children.	Demography	Mollborn, S.	+	*	-	+			1			1	Ineligible
270	2014	Socioeconomic determinants of infant growth: The Perspective Cohort Study of Thai Children.	Japan journal of nursing science	Phuphaibul, R.	+	+	+	+	1					1	Continuous Education
271	2014	[Nutritional deficit in children in a major city of the interior of the state of Bahia, Brazil].	Ciencia & saude coletiva	de Jesus, GM.	+	-	+	*			1			1	Ineligible
272	2014	Dietary diversity at 6 months of age is associated with subsequent growth and mediates the effect of maternal education on infant growth in urban Zambia.	The Journal of nutrition	Mallard, SR.	-	+	+	+			1		1		Ineligible
273	2014	Overweight in infancy: which pre- and perinatal factors determine overweight persistence or reduction? A birth cohort followed for 11 years.	Annals of nutrition & metabolism	van Rossem, L.	+	-	+	+			1			1	Ineligible
274	2014	The role of early life factors in the development of ethnic differences in growth and overweight in preschool children: a prospective birth cohort.	BMC public health	van Rossem, L.	+	-	+	*			1			1	Ineligible
275	2014	Height-for-age z scores increase despite increasing height deficits among children in 5 developing countries	American Journal of Clinical Nutrition	Lundeen, EA.	+	+	+	-			1			1	Ineligible
276	2014	Associations between infant feeding and the size, tempo and velocity of infant weight gain: SITAR analysis of the Gemini twin birth cohort	International Journal of Obesity	Johnson, L.	+	+	+	-			1	1			Ineligible
277	2014	Effect of breastfeeding on childhood BMI and obesity: The China family panel studies	Medicine (United States)	Jing, H.	*	*	+	-			1			1	Ineligible
278	2014	Is breast truly best? Estimating the effects of breastfeeding on long-term child health and wellbeing in the United States using sibling comparisons	Social Science and Medicine	Colen, CG. & Ramey, DM.	*	-	-	*			1			1	Ineligible
279	2014	The causal effect of family income on child health in the UK	Journal of Health Economics	Kuehnle, D.	+	*	-	*			1			1	Ineligible
280	2014	Important periods of weight development in childhood: A population-based longitudinal study	BMC Public Health	Glavin, K.	+	*	-	-			1			1	Ineligible
281	2014	Prenatal influences on size, velocity and tempo of infant growth: Findings from three contemporary cohorts	PLOS One	Pizzi, C.	+	+	+	-		1		1			Ineligible
282	2014	Modeling environmental influences on child growth in the MAL-ED cohort study: Opportunities and challenges	Clinical Infectious Diseases	Richard SA.	+	+	+	-			1			1	Ineligible
283	2014	Longitudinal studyof alcohol-exposed infants and toddlers in south African communities	Alcoholism: Clinical and	Kalberg, WO.	*	+	+	*			1			1	Special group cohort

			Experimental Research												
284	2014	Prevalence of weight for age > 90th percentile and z-score > 2 for healthy children ages 6 months to 2 years in a pediatric clinic	Journal of Investigative Medicine	Petty, M.	*	+	+	-			1			1	Ineligible
285	2014	The role of maternal vitamin d status in fetal and neonatal growth	Reproductive Sciences	Salari, K.	+	+	+	-			1			1	Ineligible
286	2014	What money can buy: Family income and childhood obesity	Economics and Human Biology	Jo, Y.	*	*	+	-			1			1	Ineligible
287	2014	Body Mass Index Development from Birth to Early Adolescence; Effect of Perinatal Characteristics and Maternal Migration Background in a Swedish Cohort	PLOS ONE	Pour, MB.	+	+	+	-			1			1	Ineligible
288	2014	Changes in family income status and the development of overweight and obesity from 2 to 15 years: a longitudinal study	BMC Public Health	Demment, MM.	+	+	+	-			1			1	Ineligible
289	2014	Beverage Intake in Early Childhood and Change in Body Fat from Preschool to Adolescence	Childhood obesity	Hasnain, SR.	+	*	+	-			1			1	Ineligible
290	2014	IVF conceived children Health: A retrospective growth study	Human Reproduction	Boyer Gervoise, M.	+	+	+	-			1			1	Ineligible
291	2014	Caesarean delivery, caesarean delivery on maternal request and childhood overweight: A Chinese birth cohort study of 181380 children	Pediatric Obesity	Li, H.	+	-	+	+			1			1	Ineligible
292	2014	Postnatal growth patterns of preterm and late term infants, and differences in this according to maternal education	Journal of Paediatrics and Child Health	Howe, LD.	+	*	+	+			1			1	Insufficient data
293	2015	Association between maternal age at childbirth and child and adult outcomes in the off spring: a prospective study in five low-income and middle-income countries (COHORTS collaboration)	The Lancet. Global health	Fall CH.	+	+	+	+	1					1	Insufficient data
294	2015	Determinants of Weight Gain during the First Two Years of Life-The GECKO Drenthe Birth Cohort	PLOS ONE	Küpers LK.	+	+	+	+	1			1			Insufficient data
295	2015	Longitudinal relations between children's sleep and body mass index: the moderating role of socioeconomic risk	Sleep Health: Journal of the National Sleep Foundation	Bagley, EJ.	*	-	+	*			1			1	Ineligible
296	2015	Influence of early life factors on body mass index trajectory during childhood: a population-based longitudinal analysis in the Western Brazilian Amazon	Maternal & Child Nutrition	Lourenço BH.	+	+	+	-			1			1	Ineligible
297	2015	Associations between gestational anthropometry, maternal HIV, and fetal and early infancy growth in a prospective rural/semi-rural Tanzanian cohort, 2012-13.	BMC pregnancy and childbirth	Wilkinson, AL.	+	+	+	+		1				1	Special group cohort
298	2015	Child Weight Growth Chart and Its Associated Factors in Birth Cohort of Maku Using a Growth Curve Model and LMS Method.	Global journal of health science	Ayatollahi, SMT	+	+	+	-			1			1	Ineligible
299	2015	Maternal obesity and offspring dietary patterns at 9 months of age.	European journal of clinical nutrition	Andersen, LBB.	+	+	+	-			1			1	Ineligible
300	2015	The association of exposure to the 2009 south war with the physical, psychological, and family well-being of Saudi children.	Saudi medical journal	El Hatw, MM.	+	-	+	*			1			1	Ineligible
301	2015	Maternal ratings of child health and child obesity, variations by mother's race/ethnicity and nativity.	Maternal and child health journal	Baker, EH. & Altman, CE.	+	*	+	-			1			1	Ineligible

302	2015	Body mass index trajectories from ages 1 to 20: Results from two nationally representative canadian longitudinal cohorts.	Obesity (Silver Spring, Md.)	Tu, AW.	*	-	+	*			1			1	Ineligible
303	2015	The Relationship Between Maternal Education and Child Health Outcomes in Urban Australian Children in the First 12 Months of Life.	Maternal and child health journal	Davey, TM.	+	+	-	+			1			1	Ineligible
304	2015	Ethnic differences in risk factors for obesity in New Zealand infants.	Journal of epidemiology and community health	Howe, LD.	+	+	+	-			1			1	Ineligible
305	2015	Breastfeeding duration and weight gain trajectory in infancy.	Pediatrics	Carling, SJ.	+	+	+	+		1				1	Growth trajectory
306	2015	Parental weight changes as key predictors of child weight changes.	BMC public health	Andriani, H.	+	+	+	-			1			1	Ineligible
307	2015	Early child care and obesity at 12 months of age in the Danish National Birth Cohort	International Journal of Obesity	Benjamin N.	+	+	+	-			1			1	Ineligible
308	2015	Short-term and long-term associations between household wealth and physical growth: A cross-comparative analysis of children from four low- and middle-income countries	Global Health Action	Krishna, A.	+	+	+	-			1			1	Ineligible
309	2015	Overweight/obesity and human capital formation from infancy to adolescence: Evidence from two large US cohorts	Journal of Biosocial Science	Murasko, JE.	+	+	-	*			1			1	Ineligible
310	2015	Maternal mental health, and child growth and development, in four low-income and middle-income countries	Journal of Epidemiology and Community Health	Bennett, IM.	+	+	+	-			1			1	Ineligible
311	2015	The optimal postnatal growth trajectory for term small for gestational age babies: A prospective cohort study	Journal of Pediatrics	Lei, X.	+	+	+	-			1			1	Ineligible
312	2015	Growth patterns in the first year of life differ in infants born to perinatally vs. Nonperinatally HIV-infected women	AIDS	Jao, J.	+	+	+	-			1			1	Ineligible
313	2015	Growth of Indonesian infants compared with world health organization growth standards	Journal of Pediatric Gastroenterology and Nutrition	Dwipoerwa ntoro, PG.	+	+	+	-			1			1	Ineligible
314	2015	Internal migration, international migration, and physical growth of left-behind children: A study of two settings	Health and Place	Lu, Y	-	*	+	*			1			1	Ineligible
315	2015	Impact of maternal obesity, gestational diabetes, gestational weight gain, and breastfeeding on childhood obesity at age 2 years	Diabetes	Bider, Z.	+	+	+	-			1			1	Ineligible
316	2015	Cardiovascular disease risk factors and obesity modelling among indian children: Findings from two ethnic groups of India	Atherosclerosis	Bhasin, P. & Kapoor, S.	*	+	+	-			1			1	Ineligible
317	2015	A survival analysis approach to assess the association between maternal prepregnancy overweight and childhood overweight: Results of the ulm birth cohort study (UBCS)	Hormone Research in Paediatrics	Brandt, S.	+	+	+	-			1			1	Ineligible
318	2015	Effect of air pollution on children's growth over the follow-up period of 24-Ã months: Mothers and childreng's environmental health study	European Journal of Epidemiology	Kim, E.	+	+	+	-			1			1	Ineligible
319	2015	Not weight status but parental education and perception of weight and health are related to health related quality of life in children and adolescents	Obesity Facts	Ligthart, KA.	+	+	*	-			1			1	Ineligible
320	2015	Prenatal Exposure to Traffic Pollution: Associations with Reduced Fetal Growth and Rapid Infant Weight Gain	Epidemiology	Fleisch, AF.	+	+	+	-			1			1	Ineligible
321	2015	Maternal Cultural Orientation and Child Growth in New Zealand Pacific Families	Childhood Obesity	Tseng, M.	+	+	+	-			1			1	Ineligible

322	2015	Variation in Child Body Mass Index Patterns by Race/Ethnicity and Maternal Nativity Status in the United States and England	Maternal and Child Health Journal	Martinson, ML.	+	-	+	+			1			1	Ineligible
323	2015	The role of maternal education in child health: Evidence from a compulsory schooling law	Economics of Education Review	Guenes, PM.	-	*	+	+			1			1	Ineligible
324	2015	Early Changes in Socioeconomic Status Do Not Predict Changes in Body Mass in the First Decade of Life	Annals of Behavioral Medicine	Starkey, L.	+	-	+	+			1			1	Ineligible
325	2015	Association between parental socioeconomic status with underweight and obesity in children from two Spanish birth cohorts: a changing relationship	Annals of Behavioral Medicine	Martinez-Vizcaino, V.	*	-	+	+			1			1	Ineligible
326	2016	Causal pathways between socioeconomic disadvantage and growth in the Scottish Longitudinal Study, 1991-2001	PLOS One	Silverwood, RJ.	+	-	+	+			1			1	Ineligible
327	2016	Mediating Pathways in the Socio-Economic Gradient of Child Development: Evidence from Children 6-42 Months in Bogota	International Journal of Behavioral Development	Rubio-Codina, M.	*	+	-	+			1			1	Ineligible
328	2016	Disentangling the longitudinal relations of race, sex, and socioeconomic status, for childhood body mass index trajectories	Journal of pediatric psychology	Banks, GG.	+	-	+	-			1			1	Ineligible
329	2016	[Research on the social determinants of malnutrition among children under the age of 5 in China].	Beijing Da Xue Xue Bao Yi Xue Ban.	Man, S Lm. & Guo, Y.	*	-	+	+			1			1	Ineligible
330	2016	The Linked CENTURY Study: linking three decades of clinical and public health data to examine disparities in childhood obesity.	BMC pediatrics	Hawkins, SSh.	*	+	-	-			1			1	Ineligible
331	2016	Early growth in preterm infants after hospital discharge in rural Kenya: longitudinal study.	The Pan African medical journal	Sammy, DM.	+	+	+	+		1				1	Special group cohort
332	2016	Development of a New Growth Standard for Breastfed Chinese Infants: What Is the Difference from the WHO Growth Standards?	PLOS One	Huang, X.	+	+	+	-			1			1	Ineligible
333	2016	Patterns and Determinants of Double-Burden of Malnutrition among Rural Children: Evidence from China.	PLOS One	Zhang, N.	+	-	+	+			1			1	Ineligible
334	2016	Brief Report: Maternal Age of Menarche and Adiposity: Evidence from Hong Kong's "Children of 1997" Birth Cohort.	Epidemiology (Cambridge, Mass.)	Lai, TCh.	+	*	+	-			1			1	Ineligible
335	2016	INTERGENERATIONAL EDUCATIONAL ATTAINMENT, FAMILY CHARACTERISTICS AND CHILD OBESITY.	Journal of biosocial science	Jones, A.	*	-	+	*			1			1	Ineligible
336	2016	Influence of early-life and parental factors on childhood overweight and obesity.	Journal of endocrinological investigation	Parrino, C.	-	-	+	+			1			1	Ineligible
337	2016	Protein and Micronutrient Intakes Are Associated with Child Growth and Morbidity from Infancy to Adulthood in the Philippines.	The Journal of nutrition	Bhargava, A.	+	+	+	+	1					1	Continuous Education
338	2016	Stunted at 10 Years. Linear Growth Trajectories and Stunting from Birth to Pre-Adolescence in a Rural Bangladeshi Cohort.	PLOS One	Svefors, P.	+	-	+	+			1	1			Ineligible
339	2016	The Independent and Cumulative Effect of Early Life Risk Factors on Child Growth: A Preliminary Report.	Childhood obesity (Print)	Musaad, SMA.	+	+	+	+		1		1			Growth trajectory
340	2016	Linear growth trajectories in Zimbabwean infants.	The American journal of clinical nutrition	Gough, EK.	+	+	+	+		1				1	Growth trajectory
341	2016	Comparison of Obesity Rates in Early Childhood (4 to 80 months) by Parental Socioeconomic Status Using National Cohort Dataset in Korea.	Asian nursing research	Lee, I.	+	+	+	-			1			1	Ineligible

342	2016	Growth and obesity through the first 7 y of life in association with levels of maternal glycemia during pregnancy: a prospective cohort study.	The American journal of clinical nutrition	Zhu Y.	+	+	+	-			1			1	Ineligible
343	2016	Predominantly nighttime feeding and weight outcomes in infants.	The American journal of clinical nutrition	Cheng TS.	+	+	+	-			1		1		Ineligible
344	2016	Early childhood obesity: Association with healthcare expenditure in Australia.	Obesity (Silver Spring, Md.)	Hayes, A.	+	+	+	-			1			1	Ineligible
345	2016	Feeding practices and nutritional status of HIV-exposed and HIV-unexposed infants in the Western Cape.	Southern African journal of HIV medicine	Rossouw, ME.	+	+	+	-			1			1	Ineligible
346	2016	Infertility treatment and children's longitudinal growth between birth and 3 years of age.	Human reproduction (Oxford, England)	Yeung, EH.	+	+	+	-			1			1	Ineligible
347	2016	Rural–Urban Child Height for Age Trajectories and Their Heterogeneous Determinants in Four Developing Countries	Population Research and Policy Review	Nolan, LB.	*	-	+	-			1			1	Ineligible
348	2016	Short- and long-run associations between birth weight and children's height	Economics and Human Biology	Krishna, A.	+	+	+	-			1			1	Ineligible
349	2016	Social protection for all ages? Impacts of Ethiopia's Productive Safety Net Program on child nutrition	Social Science and Medicine	Porter, C. & Goyal, R.	*	*	+	-			1			1	Ineligible
350	2016	Maternal Education and Investments in Children's Health	Journal of Marriage and Family	Prickett, KC. & Augustine, JM.	+	+	-	+			1			1	Ineligible
351	2016	Growth and development in children born very low birthweight	Archives of Disease in Childhood: Fetal and Neonatal Edition	Scharf, RJ.	+	+	+	-			1			1	Ineligible
352	2016	Growth references of preschool children based on the Taiwan Birth Cohort Study and compared to World Health Organization growth standards	Pediatrics and Neonatology	Li, YF.	*	+	+	-			1			1	Ineligible
353	2016	Assessing the impact of maternal exposure to drought on child growth	Journal of Pediatric Gastroenterology and Nutrition	Phiri, TE.	+	*	+	-			1			1	Ineligible
354	2016	BMI and food behavior in a cohort of Russian young children aged 12-35 months	Journal of Pediatric Gastroenterology and Nutrition	Surzhik, A.	+	+	+	-			1			1	Ineligible
355	2016	Predictors of improved HAZ in rural Egyptian children	FASEB Journal	Winham, DM.	+	+	+	-			1			1	Ineligible
356	2016	Is there an association between growth trajectory or season of birth with Tourette syndrome (TS) or chronic tic disorder (CT)? findings from the avon longitudinal study of parents and children (ALSPAC) cohort	Developmental Medicine and Child Neurology	Rattihalli, RR.	+	+	+	-			1			1	Ineligible
357	2016	The impact of cord blood leptin levels on growth in early infancy	Placenta	McDonald, E.	+	+	+	-			1			1	Ineligible
358	2016	Risk and resilience related to nutritional, social and lifestyle factors in children prenatally exposed to alcohol	Alcoholism: Clinical and Experimental Research	Chambers, CD. & Coles, CD	+	+	*	-			1			1	Ineligible

359	2016	Protein intake, breastfeeding frequency and breastfeeding duration affect BMI from infancy to mid-childhood	FASEB Journal	Wright, M.	+	+	+	-			1			1	Ineligible
360	2016	Can vaginal delivery reduce the intergenerational transmission of overweight and obesity? findings from the boston birth cohort	Circulation	Mueller, NT.	+	+	+	-			1			1	Ineligible
361	2016	Differential eating behavior patterns in children with prenatal alcohol exposure are related to postnatal growth trajectories	Alcoholism: Clinical and Experimental Research	Chambers, CD.	+	+	+	-			1			1	Ineligible
362	2016	Effects of family disposable income on development of height and BMI from birth up to eight years of age	Obesity Facts	Bramsved, R.	+	+	+	-			1			1	Ineligible
363	2016	Association of maternal menarcheal age with anthropometric dimensions and blood pressure in children from Greater Bilbao	Annals of Human Biology	Jelenkovic, A.	*	+	+	-			1			1	Ineligible
364	2016	Childhood obesity and the income gradient: evidence from Australia	Applied Economics	Nghiem, S. & Khanam, R.	+	-	+	+			1			1	Ineligible
365	2016	Skipping breakfast in early childhood and its associations with maternal and child BMI: a study of 2-5-year-old Australian children	European Journal of Clinical Nutrition	Alsharairi, NA. & Somerset, SM.	+	*	+	-			1			1	Ineligible
366	2016	Inappropriate Feeding Behavior: One of the Important Causes of Malnutrition in 6-to 36-Month-Old Children in Myanmar	American Journal of Tropical Medicine and Hygiene	Zhao, A.	*	+	+	-			1			1	Ineligible
367	2016	FACTORS ASSOCIATED WITH CHILDREN'S HEIGHT AND BODY MASS INDEX IN LITHUANIA, 1990-2008	Journal of Biosocial Science	Suchomlinov, A. & Tutkuviene, J.	+	+	+	-			1			1	Ineligible
368	2016	Impact of Food Assistance Programs on Obesity in Mothers and Children: A Prospective Cohort Study in Peru	American Journal of Public Health	Carrillo-Larco, RM.	*	-	+	*			1			1	Ineligible
369	2016	Early Childhood Disadvantage for Sons of Mexican Immigrants: Body Mass Index Across Ages 2-5	American Journal of Health Promotion	Lawrence, E.	+	-	+	*			1			1	Ineligible
370	2016	Early life factors predicting change in childhood body mass index	Obesity Facts	Kerr, JA.	+	*	+	-			1			1	Ineligible
371	2017	Disparities in children's vocabulary and height in relation to household wealth and parental schooling: A longitudinal study in four low-and middle-income countries. SSM Popul Health. 2017 Dec; 3: 767–786	-	Reynolds, SA.	+	+	+	-			1			1	Ineligible
372	2017	Growth of HIV-exposed uninfected, compared with HIV-unexposed, Zambian children: a longitudinal analysis from infancy to school age.	BMC pediatrics	Rosala-Hallas, A.	+	+	+	-			1			1	Ineligible
373	2017	Childhood obesity and maternal education in Ireland.	Economics and human biology	Madden, D.	+	-	+	+			1			1	Ineligible
374	2017	Catch-up growth in stunted children: Definitions and predictors.	PLOS One	Desmond, Ch. & Casale, D.	+	-	+	+			1			1	Ineligible
375	2017	The impact of maternal employment on children's adiposity: Evidence from China's labor policy reform.	Health economics	Jo, Y. & Wang, Q.	*	-	+	+			1			1	Ineligible
376	2017	Does vaginal delivery mitigate or strengthen the intergenerational association of overweight and obesity? Findings from the Boston Birth Cohort.	International journal of obesity (2005)	Mueller, NT.	+	*	+	-			1			1	Ineligible

377	2017	Status of High Body Weight Among Nurse-Family Partnership Children.	MCN. The American journal of maternal child nursing	Thorland, W.	+	+	+	-			1			1	Ineligible
378	2017	The associations of large-for-gestational-age and infant feeding practices with children's body mass index z-score trajectories: the Early Childhood Longitudinal Study, Birth Cohort.	Clinical obesity	Salahuddin, M.	+	+	+	-			1			1	Ineligible
379	2017	Association between Sleep and Body Weight: A Panel Data Model Based on a Retrospective Longitudinal Cohort of Chinese Infants.	International journal of environmental research and public health	Sha, T.	+	+	+	*	1			1			Continuous Education
380	2017	Longitudinal associations between maternal feeding and overweight in low-income toddlers.	Appetite	Lumeng, JC.	+	+	+	-			1			1	Ineligible
381	2017	Childhood stunting in relation to the pre- and postnatal environment during the first 2 years of life: The MAL-ED longitudinal birth cohort study.	PLoS medicine	MAL-ED Network Investigator s	+	+	+	-			1			1	Ineligible
382	2017	Acculturation and weight change in Asian-American children: Evidence from the ECLS-K:2011.	Preventive medicine	Diep, CS.	*	-	+	+			1			1	Ineligible
383	2017	Access to Obstetric Care and Children's Health, Growth and Cognitive Development in Vietnam: Evidence from Young Lives	Maternal and Child Health Journal	Lavin, T.	+	+	+	-			1			1	Ineligible
384	2017	Increase in weight in low birth weight and very low birth weight infants fed fortified breast milk versus formula milk: A retrospective cohort study	Nutrients	Lok, KYW.	+	+	+	-			1			1	Ineligible
385	2017	Association between parental socio-demographic factors and declined linear growth of young children in Jakarta	Medical Journal of Indonesia	Gunardi, H.	+	+	-	+		1		1			Growth trajectory
386	2017	The influence of breastfeeding duration and introduction of animal source foods, fruit juice and vegetables on the risk of weight gain in Thai children	Sri Lanka Journalof Child Health	Hong, SA.	+	+	+	-			1	1			Ineligible
387	2017	The Simultaneous Effects of Socioeconomic Disadvantage and Child Health on Children's Cognitive Development	Demography	Lee, D. & Jackson, M.	*	*	-	+			1			1	Ineligible
388	2017	Evidence that children born at early term (37-38 6/7 weeks) are at increased risk for diabetes and obesity-related disorders	American Journal of Obstetrics and Gynecology	Paz Levy, D.	+	*	+	-			1			1	Ineligible
389	2017	Studying Impact of Nutrition on Growth (SING): A prospective cohort for comparing the health outcomes of young children with the dietary quality score	BMJ Open	Lee, A.	+	+	+	-			1			1	Ineligible
390	2017	Children with access to improved sanitation but not improved water are at lower risk of stunting compared to children without access: a cohort study in Ethiopia, India, Peru, and Vietnam	BMC Public Health	Dearden, KA.	+	+	+	-			1			1	Ineligible
391	2017	The relationship between family characteristics and height in Sardinia at the turn of the twentieth century	History of the Family	Mazzoni, S.	*	-	+	*			1			1	Ineligible
392	2017	Childhood Health and Prenatal Exposure to Seasonal Food Scarcity in Ethiopia	World Development	Miller, R.	+	+	+	-			1			1	Ineligible
393	2017	Effects of socioeconomic position and social mobility on linear growth from early childhood until adolescence	Revista Brasileira de Epidemiologia	Muraro, AP.	+	-	+	+			1			1	Ineligible
394	2017	Aflatoxin exposure during the first 36 months of life was not associated with impaired growth in Nepalese children: An extension of the MAL-ED study	PLOS One	Mitchell, NJ.	+	+	+	-			1			1	Ineligible

395	2017	Prenatal care and child growth and schooling in four low- and medium-income countries	PLOS One	Liu, X.	-	+	+	-			1			1	Ineligible
396	2017	Predicting Undernutrition at Age 2 Years with Early Attained Weight and Length Compared with Weight and Length Velocity	Journal of Pediatrics	Schwinger, C.	+	+	+	-			1			1	Ineligible
397	2017	The impact of gestational endocrine disorders of breast milk fat characteristics and infant growth	FASEB Journal	Ellsworth, L.	+	+	+	-			1			1	Ineligible
398	2017	Associations of delivery mode and labor with measures of childhood adiposity: Findings from Project Viva	FASEB Journal	Mueller, NT.	+	*	+	-			1			1	Ineligible
399	2017	Maternal generation and overweight and obesity in 2-11 year old children: Evidence from birth cohort of the longitudinal study of Australian children	Obesity Facts	Zulfqar, T.	*	*	+	-			1			1	Ineligible
400	2017	Impact of maternal sth infection on infant height and weight gains at 24 months of age	Tropical Medicine and International Health	Gyorkos, TW.	+	+	+	-			1	1			Ineligible
401	2017	Causal analysis of under-nutrition in jeju and melka belo districts of Oromia region of Ethiopia	Annals of Nutrition and Metabolism	Asamo, FM.	*	+	+	-			1			1	Ineligible
402	2017	The association between Helicobacter pylori and growth in children less than 18 months of age	Helicobacter	Dror, G.	+	+	+	-			1	1			Ineligible
403	2017	Prevalence of hospital malnutrition in children under five years of age in a hospital of reference	Annals of Nutrition and Metabolism	Peralta M.	*	+	+	-			1			1	Ineligible
404	2017	Neuropsychological outcomes in a two year african-based pediatric observational study	Topics in Antiviral Medicine	Boivin, MJ.	*	*	+	-			1			1	Ineligible
405	2017	Growth changes from infancy to childhood and glucose tolerance in late adolescence: Evidence from hong kong's “children of 1997” birth cohort	Annals of Nutrition and Metabolism	Cheng, TS.	+	*	*	-			1			1	Ineligible
406	2017	Stunting in extreme nutritional vulnerability: Eastern of Democratic Republic of Congo crisis case	Tropical Medicine and International Health	Bisimwa, B.	+	+	+	-			1	1			Ineligible
407	2017	Early childhood stunting among HIV-exposed, uninfected infants in Kenya; the impact of maternal and infant diarrhea	American Journal of Tropical Medicine and Hygiene	Deichsel, EL.	+	+	+	-			1			1	Ineligible
408	2017	Early life factors and childhood habits influencing excess body weight in a Spanish multicultural cohort (calina cohort)	Annals of Nutrition and Metabolism	Rodríguez, G.	+	+	+	-			1			1	Ineligible
409	2017	Health-Related Parenting Among US Families and Young Children's Physical Health	JOURNAL OF MARRIAGE AND FAMILY	Augustine, JM.	+	*	+	-			1			1	Ineligible
410	2017	Risk factors for childhood obesity: Do the birth weight, type of delivery, and mother's overweight have an implication on current weight status?	WORLD JOURNAL OF PEDIATRICS	Vehapoglu, A.	*	-	+	+			1			1	Ineligible
411	2017	Breastfeeding duration and its relation to weight gain, eating behaviours and positive maternal feeding practices in infancy	APPETITE	Rogers, SL.	+	+	+	-			1	1			Ineligible
412	2017	The Longitudinal Association Between Early Childhood Obesity and Fathers' Involvement in Caregiving and Decision-Making	OBESITY	Wong, MS.	+	+	+	-			1			1	Ineligible
413	2017	Maternal and infant factors had a significant impact on birthweight andlongitudinal growth in a South African birth cohort	ACTA PAEDIATRICA	Budree, S.	+	+	+	-			1			1	Ineligible
414	2017	Intergrowth 21 st project: What is new for newborn anthropometric assessment?	Annals of Nutrition and Metabolism	Medina, M.	*	+	+	-			1			1	Ineligible

415	2017	Becoming malnourished children, one year after nutrition education in kaya (burkina faso)	Annals of Nutrition and Metabolism	Garanet, F.	+	*	+	-			1			1	Ineligible
416	2018	Growth patterns from birth to 24 months in Chinese children: a birth cohorts study across China.	BMC pediatrics	Ouyang, F.	+	+	+	-			1			1	Ineligible
417	2018	Socioeconomic status, and health during childhood: A longitudinal examination of racial/ethnic differences in parental socioeconomic timing and child obesity risk	Int J Environ Res Public Health	Race Jones, A. & Jones, A.	+	-	+	+			1			1	Ineligible
418	2018	The Effect of Paternal Education on Child Health in South Africa: A Longitudinal Analysis		Pillay, SR.	-	+	+	+			1			1	Ineligible
419	2018	Micronutrient adequacy is poor, but not associated with stunting between 12-24 months of age: A cohort study findings from a slum area of Bangladesh.	PLOS One	Sanin, KI.	+	+	+	+	1					1	Continuous Education
420	2018	Comparative Models of Biological and Social Pathways to Predict Child Growth through Age 2 Years from Birth Cohorts in Brazil, India, the Philippines, and South Africa.	The Journal of nutrition	Richter, LM.	*	+	+	-			1			1	Ineligible
421	2018	Prospective associations between social vulnerabilities and children's weight status. Results from the IDEFICS study.	International journal of obesity (2005)	Iguacel, I.	*	-	+	+			1			1	Ineligible
422	2018	Enteroaggregative Escherichia coli Subclinical Infection and Coinfections and Impaired Child Growth in the MAL-ED Cohort Study.	Journal of pediatric gastroenterology and nutrition	Lima AAM.	+	+	+	-			1	1			Ineligible
423	2018	Role of maternal preconception nutrition on offspring growth and risk of stunting across the first 1000 days in Vietnam: A prospective cohort study.	PLOS One	Young, MF.	+	+	+	+		1				1	Ineligible
424	2018	Prenatal risk factors influencing childhood BMI and overweight independent of birth weight and infancy BMI: a path analysis within the Danish National Birth Cohort.	International journal of obesity (2005)	Morgen, CS.	+	-	+	+			1			1	Ineligible
425	2018	Physical and psychosocial development of Mapuche and nonindigenous Chilean toddlers: A modest role of ethnicity.	Development and psychopathology	Navarrete, MA.	+	-	+	+			1			1	Ineligible
426	2018	[Early childhood development and risk factors in rural China: a cohort study].	Zhonghua er ke za zhi = Chinese journal of pediatrics	Cui, Y.	+	+	-	*			1			1	Ineligible
427	2018	Association of maternal depression with dietary intake, growth, and development of preterm infants: a cohort study in Beijing, China.	Frontiers of medicine	Wang, H.	+	+	+	-			1			1	Ineligible
428	2018	Infectious Morbidity, Mortality and Nutrition in HIV-exposed, Uninfected, Formula-fed Infants: Results From the HPTN 040/PACTG 1043 Trial.	The Pediatric infectious disease journal	Yeganeh, N.	*	+	+	-			1			1	Ineligible
429	2018	Racial and Ethnic Disparities in Early Childhood Obesity.	Pediatrics	Isong, IA.	+	-	+	+			1			1	Ineligible
430	2018	Does Village Chicken-Keeping Contribute to Young Children's Diets and Growth? A Longitudinal Observational Study in Rural Tanzania.	Nutrients	de Bruyn, J.	+	-	+	*			1	1			Ineligible
431	2018	The role of fathers in overweight prevention: an analysis of a Caribbean cohort.	Global health, epidemiology and genomics	Smith, JA.	-	+	+	+		1				1	Ineligible
432	2018	Picky eating, pressuring feeding, and growth in toddlers.	Appetite	Lumeng, JC.	+	+	+	-			1			1	Ineligible
433	2018	Association of Elective and Emergency Cesarean Delivery With Early Childhood Overweight at 12 Months of Age.	JAMA network open	Cai, M.	+	+	+	-			1			1	Ineligible

434	2018	Maternal preconception weight trajectories, pregnancy complications and offspring's childhood physical and cognitive development.	Journal of developmental origins of health and disease	Adane, AA.	+	*	-	*			1			1	Ineligible
435	2018	Early maternal perceived stress and children's BMI: longitudinal impact and influencing factors.	BMC public health	Leppert, B.	+	+	+	-			1			1	Ineligible
436	2018	Causal Effect of Parental Schooling on Early Childhood Undernutrition: Quasi-Experimental Evidence From Zimbabwe.	American journal of epidemiology	De Neve, JW.	-	+	+	+			1			1	Ineligible
437	2018	Body Mass Index Changes in Early Childhood.	The Journal of pediatrics	Jabakhanji, SB.	+	+	+	-			1			1	Ineligible
438	2018	Somatic growth from birth to 6 months in low birth weight, in Bukavu, South Kivu, Democratic Republic of the Congo.	Revue d'epidemiologie et de sante publique	Mbusa-Kambale, R.	+	+	+	-			1			1	Ineligible
439	2018	Parental Socioeconomic Instability and Child Obesity.	Biodemography and social biology	Jones, A.	+	-	+	+			1			1	Ineligible
440	2018	Fetal vitamin D concentration and growth, adiposity and neurodevelopment during infancy.	European journal of clinical nutrition	Wang, H.	+	+	+	-			1			1	Ineligible
441	2018	Prenatal exposure to preeclampsia is associated with accelerated height gain in early childhood.	PLOS One	Gunnarsdottir, J.	+	+	+	-			1			1	Ineligible
442	2018	The Associations of Breast Feeding with Infant Growth and Body Mass Index to 16 years: 'Children of 1997'.	Paediatric and perinatal epidemiology	Cheng, TS	+	+	+	-			1		1		Ineligible
443	2018	Duration of Breastfeeding, but Not Timing of Solid Food, Reduces the Risk of Overweight and Obesity in Children Aged 24 to 36 Months: Findings from an Australian Cohort Study.	International journal of environmental research and public health	Bell, S.	+	-	+	+			1			1	Ineligible
444	2018	Estimating the pathways through which maternal education affects stunting: evidence from an urban cohort in South Africa.	Public health nutrition	Casale, D.	+	+	+	+	1					1	Continuous Education
445	2018	Social vulnerabilities as determinants of overweight in 2-, 4- and 6-year-old Spanish children.	European journal of public health	Iguacel, I.	+	-	+	+			1			1	Ineligible
446	2018	Socioeconomic differences in childhood BMI trajectories in Belarus.	International journal of obesity (2005)	Patel, R.	-	+	+	+		1		1			Ineligible
447	2018	Parental education and family income affect birthweight, early longitudinal growth and body mass index development differently.	Acta paediatrica (Oslo, Norway : 1992)	Bramsved, R.	+	+	+	-			1			1	Ineligible
448	2018	Association of maternal gestational weight gain with their offspring's anthropometric outcomes at late infancy and 6 years old: mediating roles of birth weight and breastfeeding duration.	International journal of obesity (2005)	Liu, JX.	+	+	+	-			1			1	Ineligible
449	2018	Sociodemographic factors and overweight in children participating in a government program for fortified milk distribution	Journal of Human Growth and Development	Escaldelai, FMD.	+	+	+	-			1	1			Ineligible
450	2018	Exclusive breastfeeding and partial breastfeeding reduce the risk of overweight in childhood: A nationwide longitudinal study in Korea	Obesity Research and Clinical Practice	Park, SJ.	+	+	+	-			1			1	Ineligible
451	2018	Regional variation in weight-for-height z-scores and surface area/body mass ratio of Chilean children from birth to 3 years of age	American Journal of Physical Anthropology	Retamal, R.	+	+	+	-			1			1	Ineligible

452	2018	Adolescent mothers' anthropometrics and grandmothers' schooling predict infant anthropometrics in Ethiopia, India, Peru, and Vietnam	Annals of the New York Academy of Sciences	Schott, W.	+	-	+	*			1			1	Ineligible
453	2018	Growth and biochemical markers of preterm newborns up to six months of corrected age	Journal of Human Growth and Development	Barreto, GMS.	+	+	+	-			1			1	Ineligible
454	2018	FRAGILE ENVIRONMENT, SEASONALITY AND MATERNAL AND CHILDHOOD UNDERNUTRITION IN BANGLADESH	Journal of Biosocial Science	Mohsena, M.	*	*	+	-			1			1	Ineligible
455	2018	The impact of maternal mental health shocks on child health: Estimates from fixed-effects instrumental variables models for two cohorts of Australian children	American Journal of Health Economics	Le, HT. & Nguyen, HT.	+	+	+	-			1			1	Ineligible
456	2018	Body composition during early infancy and developmental progression from 1 to 5 years of age: the Infant Anthropometry and Body Composition (iABC) cohort study among Ethiopian children	British Journal of Nutrition	Abera, M.	+	*	-	-			1	1			Ineligible
457	2018	Family size effects on childhood obesity: Evidence on the quantity-quality trade-off using the NLSY	Economics & Human Biology	Dasgupta, K.	*	*	+	-			1			1	Ineligible
458	2018	The Influence of Ethnicity on Exclusively Breast-Fed Infants' Anthropometry in a Multiethnic Asian Population	Annals of the Academy of Medicine Singapore	Huang, JG.	+	+	+	-			1			1	Ineligible
459	2018	Prenatal stress exposure and early childhood BMI: Exploring associations in a New Zealand context	American Journal of Human Biology	Farewell, ChV.	+	+	+	+	1			1			Continuous Education
460	2018	The Associations of Breast Feeding with Infant Growth and Body Mass Index to 16years: ``Children of 1997'	Paediatric and Perinatal Epidemiology	Cheng, TS.	+	+	+	-			1			1	Ineligible
461	2018	Relationship between socioeconomic status and weight gain during infancy: The BeeBOFT study	PLOS ONE	Wang, L.	-	+	+	+		1				1	Ineligible
462	2018	Parents with overweight children two and five years of age did not perceive them as weighing too much	Acta Paediatrica	Berggren, S.	+	+	+	-			1			1	Ineligible
463	2018	Household Food Insecurity and Early Childhood Health and Cognitive Development Among Children of Immigrants	Journal of Family Issues	Huang, Y.	+	*	-	-			1			1	Ineligible
464	2018	Associations among High-Quality Protein and Energy Intake, Serum Transthyretin, Serum Amino Acids and Linear Growth of Children in Ethiopia	Nutrients	Tessema, M.	-	+	+	+		1				1	Ineligible
465	2019	Associations of less healthy snack food consumption with infantweight-for-length Z-score trajectories: Findings from the nurture cohort study	Nutrients	Moore, AM.	+	+	+	-			1			1	Ineligible
466	2019	Relative importance of prenatal and postnatal determinants of stunting: data mining approaches to the MINIMat cohort, Bangladesh.	BMJ open	Svefors, P.	-	+	+	+		1				1	Ineligible
467	2019	Western Australian pregnancy cohort (Raine) Study: Generation 1.	BMJ open	Dontje, ML.	+	*	-	-			1			1	Ineligible
468	2019	Infant nutrition and growth: trends and inequalities in four population-based birth cohorts in Pelotas, Brazil, 1982-2015.	International journal of epidemiology	Gonçalves, H.	+	+	+	-			1			1	Ineligible
469	2019	The relationship between responsive caregiving and child outcomes: evidence from direct observations of mother-child dyads in Pakistan.	BMC public health	Scherer, E.	+	*	+	-			1			1	Ineligible
470	2019	Sex effect on growth faltering in an indigenous ethnic minority population of infants in Israel.	Public health nutrition	Bilenko, N.	+	+	+	-			1			1	Ineligible
471	2019	The role of offspring's birthweight on the association between pre-pregnancy obesity and offspring's childhood anthropometrics: a mediation analysis.	Journal of developmental	Adane, AA.	+	-	+	*			1			1	Ineligible

			origins of health and disease												
472	2019	Long work hours of mothers and fathers are linked to increased risk for overweight and obesity among preschool children: longitudinal evidence from Germany.	Journal of epidemiology and community health	Li, J.	+	-	+	+			1			1	Ineligible
473	2019	Analysing child linear growth trajectories among under-5 children in two Nairobi informal settlements.	Public health nutrition	Faye, ChM.	+	-	+	*			1			1	Ineligible
474	2019	The double burden of malnutrition among youth: Trajectories and inequalities in four emerging economies.	Economics and human biology	Schott, W.	*	-	+	+			1			1	Ineligible
475	2019	Adolescent pregnancy and linear growth of infants: a birth cohort study in rural Ethiopia.	Nutrition journal	Workicho, A.	+	+	+	+	1			1			Height gain
476	2019	BMI trajectories and risk factors among 2-11-year-old children by their immigrant status: evidence from the Longitudinal Study of Australian Children.	BMJ open	Zulfiqar, T.	+	+	+	-			1			1	Ineligible
477	2019	Antibiotic use in early childhood and risk of obesity: longitudinal analysis of a national cohort.	World journal of pediatrics	Kelly, D.	+	-	+	+			1			1	Ineligible
478	2019	The association between antibiotics in the first year of life and child growth trajectory.	BMC pediatrics	Dawson-Hahn, EE. & Rhee, KE.	+	-	+	-			1			1	Ineligible
479	2019	Factors associated with the evolution of weight of children in a supplementary feeding program.	Revista brasileira de epidemiologia = Brazilian journal of epidemiology	Ortelan, N.	+	-	+	+			1			1	Ineligible
480	2019	Undernutrition of HEU infants in their first 1000 days of life: A case in the urban-low resource setting of Mukuru Slum, Nairobi, Kenya.	Heliyon	Wambura, JN. & Marnane, B.	+	+	+	-			1			1	Ineligible
481	2019	Low head circumference during early childhood and its predictors in a semi-urban settlement of Vellore, Southern India.	BMC pediatrics	Sindhu KN.	+	+	-	+			1			1	Ineligible
482	2019	Provision of low-iron micronutrient powders on alternate days is associated with lower prevalence of anaemia, stunting, and improved motor milestone acquisition in the first year of life: A retrospective cohort study in rural Ethiopia.	Maternal & child nutrition	Geletu, A.	+	+	+	-			1			1	Ineligible
483	2019	Effects of infant feeding practices and maternal characteristics on early childhood obesity.	Archivos argentinos de pediatria	Ardic, C.	+	-	+	+			1			1	Ineligible
484	2019	Factors associated with catch-up growth in early infancy in rural Pakistan: A longitudinal analysis of the women's work and nutrition study.	Maternal & child nutrition	Pradeilles R.	+	+	+	+	1			1			Height gain
485	2019	Stunting trajectories from post-infancy to adolescence in Ethiopia, India, Peru, and Vietnam	Maternal and Child Nutrition		+	+	+	-			1			1	Ineligible
486	2019	High Maternal Circulating Cotinine during Pregnancy is Associated with Persistently Shorter Stature from Birth to Five Years in an Asian Cohort	Nicotine and Tobacco Research	Ng, S.	+	+	+	-			1			1	Ineligible
487	2019	Haemophilus influenzae type b vaccination and anthropometric, cognitive, and schooling outcomes among Indian children	Annals of the New York Academy of Sciences	Nandi A.	+	+	+	-			1			1	Ineligible
488	2019	Correction: Examining the relationship between socio-economic status, WASH practices and wasting (PLOS One (2017) 12: 3 (e0172134) DOI: 10.1371/journal.pone.0172134)	PLOS One	Raihan MJ.	-	+	+	-			1			1	Ineligible

489	2019	Childhood Obesity Differences in Single-Father and Single-Mother Families	Sociological Focus	Noonan, M. & Turchi, J.	*	-	+	*			1			1	Ineligible
490	2019	Lifetime economic impact of the burden of childhood stunting attributable to maternal psychosocial risk factors in 137 low/middle-income countries	BMJ Global Health	Smith Fawzi, MC.	-	*	+	+			1			1	Ineligible
491	2019	The relationship between wasting and stunting: A retrospective cohort analysis of longitudinal data in Gambian children from 1976 to 2016	American Journal of Clinical Nutrition	Schoenbuchner, SM.	+	+	+	-			1			1	Ineligible
492	2019	Postnatal depression and infant growth in an urban area of Bangladesh	Midwifery	Sharmin, KN.	+	*	+	-			1			1	Ineligible
493	2019	Labor migration in Indonesia and the health of children left behind	IZA Journal of Development and Migration	Ng, J.	-	*	+	-			1			1	Ineligible
494	2019	Malnutrition in children of northern Mozambique: Only a small picture of a global reality!	European Journal of Pediatrics	Santos, JC.	*	+	+	-			1			1	Ineligible
495	2019	Growth in children conceived by assisted reproductive technologies: The Norwegian Mother and Child Cohort Study	Norsk Epidemiologi	Magnus, MC.	+	+	+	-			1			1	Ineligible
496	2019	Validity of LATCH Score in predicting exclusive breast feeding and weight velocity at 6 weeks in healthy term Indian infants	Journal of Pediatric Gastroenterology and Nutrition	Shah, MH.	+	+	+	-			1			1	Ineligible
497	2019	Early life exposure to cigarettes is associated with adverse long-term health outcomes in a large, multicentre cohort	Journal of Cystic Fibrosis	Rosenfeld, M..	+	*	+	-			1			1	Ineligible
498	2019	DNA methylation biomarkers of early life exposures and subsequent obesity: Findings from the Newcastle Thousand Families Study and the Avon Longitudinal Study of Parents and Children	Mutagenesis	Robinson, N.	+	+	+	-			1			1	Ineligible
499	2019	The influence of age-appropriate feeding practices on the growth of Cambodian children: A longitudinal study	Annals of Nutrition and Metabolism	Hondru, G.	+	+	+	-			1			1	Ineligible
500	2019	Prevalence of stunting among Malaysian infants aged 6 months and its associated factors	Annals of Nutrition and Metabolism	Shazalli, FH.	+	+	+	-			1	1			Insufficient data
501	2019	Comparative Study of Growth and Development in Children with Cleft Lip and Palate Versus Children without Cleft	Journal of Oral and Maxillofacial Surgery	Cordero, E.	+	+	+	-			1			1	Ineligible
502	2019	Small intestine bacterial overgrowth is associated with linear growth delay in a longitudinal analysis of Bangladeshi children	American Journal of Tropical Medicine and Hygiene	Donowitz, J.	+	+	+	-			1			1	Ineligible
503	2019	The relationship between socioeconomic status and obesity in Korean children: Analysis from the Korean national survey (2007-2015)	Journal of Pediatric Gastroenterology and Nutrition	Lee, HJ. & Lee, JH.	*	-	+	+			1			1	Ineligible
504	2019	Associations of growth from birth to puberty with glycemic indicators at similar to 17.5years: Evidence from Hong Kong's ``Children of 1997{''} birth cohort	PEDIATRIC DIABETES	Cheng, TS.	+	+	+	-			1	1			Ineligible
505	2019	Infant nutrition and growth: trends and inequalities in four population-based birth cohorts in Pelotas, Brazil, 1982-2015	International Journal of Epidemiology	Gonçalves H.	+	+	+	-			1			1	Ineligible
506	2019	Mother and Infant Predictors of Rapid Infant Weight Gain	Clinical Pediatrics	Pesch, MH.	+	+	+	-			1			1	Ineligible
507	2019	Factors associated with recovery from stunting among under-five children in two Nairobi informal settlements	PLOS One	Faye, ChM.	+	-	+	*			1			1	Ineligible

508	2020	DIRECT AND INDIRECT CAUSES OF STUNTING AT FIVE SUB-DISTRICT IN CENTRAL BOGOR DISTRICT, BOGOR CITY (A QUALITATIVE STUDY OF CHILD DEVELOPMENT COHORT, 2019)	Buletin Penelitian Sistem Kesehatan	Rosha, BCh.	+	+	+	-			1			1	Ineligible
509	2020	Pathways to inflammation in adolescence through early adversity, childhood depressive symptoms, and body mass index: A prospective longitudinal study of Chilean infants.	Brain, behavior, and immunity	Reid, BM.	+	*	*	-			1			1	Ineligible
510	2020	Are Household Expenditures on Food Groups Associated with Children's Future Heights in Ethiopia, India, Peru, and Vietnam?	International journal of environmental research and public health	Weingarten , SE.	+	-	+	*			1			1	Ineligible
511	2020	BMI mobility and obesity transitions among children in Ireland.	Economics and human biology	Madden, D.	+	-	+	+			1			1	Ineligible
512	2020	Prenatal anxiety, breastfeeding and child growth and puberty: linking evolutionary models with human cohort studies.	Annals of human biology	English, S.	+	*	+	-			1			1	Ineligible
513	2020	Baseline Characteristics of Study Participants in the Early Life Interventions for Childhood Growth and Development in Tanzania (ELICIT) Trial.	The American journal of tropical medicine and hygiene	Parpia TC.	-	+	+	+		1				1	Ineligible
514	2020	[A new examination of the determinants of weight in early childhood].	Revista chilena de pediatria	Salinas, V. & Goldsmith Weil, J.	-	+	+	+			1			1	Ineligible
515	2020	Factors associated with early childhood stunted growth in a 2012-2015 birth cohort monitored in the rural Msambweni area of coastal Kenya: a cross-sectional study.	BMC pediatrics	Martin, Sh.	+	+	+	-			1			1	Ineligible
516	2020	[The LIFE Child study: A cohort study investigating Child Development in changing environmental conditions].	Zeitschrift fur Psychosomatische Medizin und Psychotherapie	Poulain, T.	+	*	*	-			1			1	Ineligible
517	2020	Prenatal exposure to traffic and ambient air pollution and infant weight and adiposity: The Healthy Start study.	Environmental research	Starling, AnP.	+	+	-	+			1			1	Ineligible
518	2020	Associations between growth from birth to 18 years, intelligence, and schooling in a Brazilian cohort.	The American journal of clinical nutrition	Baptista M.	+	+	+	-			1			1	Ineligible
519	2020	Factors associated with head circumference and indices of cognitive development in early childhood.	BMJ global health	Nicolaou, L.	+	+	-	-			1			1	Ineligible
520	2020	Maternal nutritional status mediates the linkage between household food insecurity and mid-infancy size in rural Bangladesh.	The British journal of nutrition	Na, M.	-	+	+	+		1				1	Ineligible
521	2020	Sociodemographic predictors of early postnatal growth: evidence from a Chilean infancy cohort.	BMJ open	Von Holle, A.	-	+	+	+		1		1			Ineligible
522	2020	Maternal Socioeconomic Factors and Racial/Ethnic Differences in Neonatal Anthropometry.	International journal of environmental research and public health	Lambert, C.	+	-	+	+			1			1	Ineligible
523	2020	Social determinants of overweight and obesity in the mother-child binomial: evidences from Mexico.	Archives of public health = Archives belges de sante publique	Arredondo, A.	+	*	-	+			1			1	Ineligible

524	2020	Social and economic factors, maternal behaviours in pregnancy and neonatal adiposity in the PANDORA cohort.	Diabetes research and clinical practice	Longmore, DK.	+	-	+	+			1			1	Ineligible
525	2020	Determinants of infant and young complementary feeding practices among children 6-23 months of age in urban Pakistan: a multicenter longitudinal study.	BMC nutrition	Ariff, Sh.	+	+	-	+			1	1			Ineligible
526	2020	Can shorter mothers have taller children? Nutritional mobility, health equity and the intergenerational transmission of relative height	Economics and Human Biology	Finaret, AB. & Masters, WA.	-	*	+	*			1			1	Ineligible
527	2020	Early Child Care and Weight Status in a Cohort of Predominantly Black Infants in the Southeastern United States	Childhood Obesity	Benjamin-Neelon, SE.	+	+	+	-			1			1	Ineligible
528	2020	Machine learning model demonstrates stunting at birth and systemic inflammatory biomarkers as predictors of subsequent infant growth – a four-year prospective study	BMC Pediatrics	Harrison, E.	+	+	+	-			1			1	Ineligible
529	2020	Dynamics of stunting from childhood to youthhood in Ethiopia: Evidence from the Young Lives panel data	PLOS One	Astatkie, A.	*	-	+	*			1	1			Ineligible
530	2020	Nutrition in Pregnancy and Growth in Southwest China (NPGSC) cohort: Design, implementation, and characteristics	Paediatric and Perinatal Epidemiology	Zhang, X.	*	+	*	-			1			1	Ineligible
531	2020	Association of plasma total cysteine and anthropometric status in 6–30 months old indian children	Nutrients	Schwinger, C.	+	+	+	-			1			1	Ineligible
532	2020	Genetic and environmental influences on human height from infancy through adulthood at different levels of parental education	Scientific Reports	Jelenkovic A.	-	+	+	-			1	1			Ineligible
533	2020	Stunting prevalence and its relationship to birth length of 18–23 months old infants in Indonesia	Enfermeria Clinica	Hastuti	+	+	+	-			1			1	Ineligible
534	2020	Trajectory of inequality of opportunity in child height growth: Evidence from the Young Lives study	Demographic Research	Aizawa, T.	*	+	+	-			1			1	Ineligible
535	2020	Environment-wide association study on childhood obesity in the U.S.	Environmental Research	Uche, UI.	*	+	+	-			1			1	Ineligible
536	2020	Prenatal air pollution exposure and growth and cardio-metabolic risk in preschoolers	Environment International	Fossati, S.	+	+	+	-			1			1	Ineligible
537	2020	Age-appropriate feeding practices in cambodia and the possible influence on the growth of the children: A longitudinal study	Nutrients	Hondru, G.	*	-	+	+		1		1			Ineligible
538	2020	A biocultural examination of home food production and child growth in highland Peru	American Journal of Human Biology	Hoke, MK.	*	+	+	-			1			1	Ineligible
539	2020	Association of Risperidone Treatment and Change in BMI in Children Under 5 Years Old: A Retrospective Cohort Study	Biological Psychiatry	Avrahami, M.	+	*	+	-			1			1	Ineligible
540	2020	Malnutrition in HIV-exposed uninfected children in long-term observational follow-up	Topics in Antiviral Medicine	Stranix-Chibanda, L.	+	*	+	-			1			1	Ineligible
541	2020	BMI from birth to age 10 years by types of maternal diabetes during pregnancy	Diabetes	Sidell, MA.	+	+	+	-			1			1	Ineligible
542	2020	Early Infant Feeding and BMI Trajectories in the First 5 Years of Life	Obesity	Zheng, M.	+	+	+	-			1			1	Ineligible
543	2020	Using longitudinal data to understand nutrition and health interactions in rural Gambia	Annals of Human Biology	Moore, SE.	+	*	*	-			1			1	Ineligible
544	2020	Why Who Marries Whom Matters: Effects of Educational Assortative Mating on Infant Health in the United States 1969-1994	Social Forces	Rauscher, E.	+	+	-	+			1	1			Ineligible
545	2021	The excess weight of 0 to 7 year-old children. Risk factors	Obesity Facts	Pereda-Pereda, E.	+	+	+	-			1			1	Ineligible

546	2021	1019 Socioeconomic inequities in 5-year weight trajectories in association with baseline weight-related perceptions: a longitudinal study	International Journal of Epidemiology	Feng, X. & Wilson, A.	+	*	+	-			1			1	Ineligible
547	2021	Poor weight gain and its predictors among preterm neonates admitted at Muhimbili National Hospital in Dar-es-salaam, Tanzania: a prospective cohort study	BMC Pediatrics	Ndembo, VP.	+	+	+	+		1				1	Special group cohort
548	2021	Cord blood metabolic signatures predictive of childhood overweight and rapid growth	International Journal of Obesity	Handakas E.	+	+	+	-			1			1	Ineligible
549	2021	The Biological and Social Determinants of Childhood Obesity: Comparison of 2 Cohorts 50 Years Apart	Journal of Pediatrics	Robinson, N.	*	+	+	-			1			1	Ineligible
550	2021	Linear Growth Trajectories in Early Childhood and Adult Cognitive and Socioemotional Functioning in a Guatemalan Cohort	Journal of Nutrition	Ramírez-Luzuriaga, MJ.	+	-	*	*			1			1	Ineligible
551	2021	Association between perinatal factors, genetic susceptibility to obesity and age at adiposity rebound in children of the EDEN mother-child cohort	International Journal of Obesity	Cisse, AH.	+	-	+	*			1			1	Ineligible
552	2021	Are there birth cohort effects in disparities in child obesity by maternal education?	International Journal of Obesity	Le-Scherban, F.	*	+	+	+		1				1	Insufficient data
553	2021	Associations between maternal obesity, gestational cytokine levels and child obesity in the NEST cohort.	Pediatric obesity	Maguire, RL.	+	*	+	-			1			1	Ineligible
554	2021	Longitudinal analysis of growth trajectories in young children of Chinese-born immigrant mothers compared with Australian-born mothers living in Victoria, Australia.	BMJ open	Bolton, KA.	+	+	+	-			1			1	Ineligible
555	2021	Longitudinal outcomes of body mass index in overweight and obese children with chronic kidney disease.	Pediatric nephrology (Berlin, Germany)	Rodig, NM.	+	-	+	+			1			1	Ineligible
556	2021	Factors affecting the growth of children till the age of three years with overweight whose mothers have diabetes mellitus: A population-based cohort study.	BMC pediatrics	Huang, YD.	+	+	+	-			1			1	Ineligible
557	2021	Diarrheal Pathogens Associated with Growth and Neurodevelopment.	Clinical infectious diseases	Donowitz JR.	+	+	+	-			1			1	Ineligible
558	2021	Maternal and infant prediction of the child BMI trajectories; studies across two generations of Northern Finland birth cohorts.	International journal of obesity (2005)	Nedelec, R.	+	*	+	-			1			1	Ineligible
559	2021	Anthropometrics of Estonian children in relation to family disruption: Thrifty phenotype and Trivers-Willard effects.	Evolution, medicine, and public health	Valge, M.	+	*	+	-			1			1	Ineligible
560	2021	Early childhood weight gain: Latent patterns and body composition outcomes.	Paediatric and perinatal epidemiology	Norris, T.	+	+	+	+		1				1	Growth trajectory
561	2021	Socioeconomic disparities and infancy growth trajectory: a population-based and longitudinal study.	BMC pediatrics	Shao, ZY.	+	+	+	+		1				1	Growth trajectory
562	2021	Maternal Tobacco and Alcohol Use in Relation to Child Malnutrition in Gauteng, South Africa: A Retrospective Analysis.	Children (Basel, Switzerland)	Modjadji, P.	-	+	+	*			1			1	Ineligible
563	2021	Exploring the relationships between wasting and stunting among a cohort of children under two years of age in Niger.	BMC public health	Kohlmann, K.	-	+	+	+		1				1	Ineligible
564	2021	Prenatal exposure to ambient air pollutants and early infant growth and adiposity in the Southern California Mother's Milk Study.	Environmental health	Patterson, WB.	+	+	+	-			1			1	Ineligible
565	2021	Influences on catch-up growth using relative versus absolute metrics: evidence from the MAL-ED cohort study.	BMC public health	Richard, SA.	+	+	+	-			1			1	Ineligible

566	2021	Growth patterns and their contributing factors among HIV-exposed uninfected infants.	Maternal & child nutrition	Ndiaye, A.	+	*	+	*		1				1	Special group cohort
567	2021	Off the Charts: Identifying and Visualizing Body Mass Index Trajectories of Rural, Poor Youth.	The Journal of pediatrics	Rollins, BY.	+	+	+	*		1				1	Growth trajectory
568	2021	Long-term growth in offspring of infertile parents: A 20-year follow-up study.	Acta obstetricia et gynecologica Scandinavica	Hinchely Ebdrup, N.	+	*	+	-			1			1	Ineligible
569	2021	High sugar content and body mass index: modelling pathways around the first 1000 d of life, BRISA cohort.	Public health nutrition	Pinto, DAS.	+	+	+	-			1			1	Ineligible
570	2021	Spatial Variation of Child Stunting and Maternal Malnutrition after Controlling for Known Risk Factors in a Drought-Prone Rural Community in Southern Ethiopia.	Annals of global health	Belayneh, M.	+	-	+	*			1			1	Ineligible
571	2021	Gestational weight gain and childhood body mass index across three generations: Results from the 1993 Pelotas (Brazil) Birth Cohort.	Pediatric obesity	Schneider, BC.	+	*	+	-			1			1	Ineligible
572	2021	Associations between the Mediterranean Diet Pattern and Weight Status and Cognitive Development in Preschool Children.	Nutrients	Granziera, F.	*	-	+	*			1			1	Ineligible
573	2021	Common Determinants of Dental Caries and Obesity in Children: A Multi-Ethnic Nested Birth Cohort Study in the United Kingdom.	International journal of environmental research and public health	Uerlich, MF.	+	+	+	-			1			1	Ineligible
574	2021	Complementary Feeding Habits in Children Under the Age of 2 Years Living in the City of Adama in the Oromia Region in Central Ethiopia: Traditional Ethiopian Food Study.	Frontiers in nutrition	Gudeta, AN.	+	+	-	-			1	1			Ineligible
575	2021	Maternal Smoking During Pregnancy and Offspring Head Growth in Comparison to Height and Weight Growth Up to 6 Years of Age: A Longitudinal Study.	Clinical epidemiology	Karvonen, M.	+	+	+	-			1			1	Ineligible
576	2021	Association between parental socioeconomic status and offspring overweight/obesity from the China Family Panel Studies: a longitudinal survey.	BMJ open	Ding, S.	-	*	+	+		1				1	Ineligible
577	2021	Sexual dimorphism in growth pattern among the infants with respect to weight during the first year of life: A longitudinal study	Sri Lanka Journal of Child Health	Sinha, NK.	+	+	+	-			1			1	Ineligible
578	2021	Prenatal exposure to air pollutants and early childhood growth trajectories: A population-based prospective birth cohort study	Environmental Research	Tan, Y.	+	+	+	+		1				1	Growth trajectory
579	2021	Relationship between maternal employment and 9-12 month-old infants' growth indexes in ahwaz (2017)	Iranian Journal of Epidemiology	Najibi, S.	+	+	+	+		1		1			Growth trajectory
580	2021	Trajectory of Body Mass Index from Ages 2 to 7 Years and Age at Peak Height Velocity in Boys and Girls	Journal of Pediatrics	Chen, LK.	+	-	+	*			1			1	Ineligible
581	2021	Association of maternal depression, family composition and poverty with maternal care and physical health of children in the first year of life	Journal of Human Growth and Development	Scherrer, IRS. & Alves, CRL.	+	+	-	+			1			1	Ineligible
582	2021	Comparison of Postnatal Growth Charts of Singleton Preterm and Term Infants Using World Health Organization Standards at 40–160 Weeks Postmenstrual Age: A Chinese Single-Center Retrospective Cohort Study	Frontiers in Pediatrics	Zhang, L.	+	+	+	-			1			1	Ineligible
583	2021	Agricultural production diversity and child nutrition in Ethiopia	Food Security	Bakhtsiyarava, M. & Grace, K.	-	-	+	*			1			1	Ineligible

584	2021	Childhood growth of term singletons born after frozen compared with fresh embryo transfer	Reproductive BioMedicine Online	Terho, AM.	+	+	+	-			1			1	Ineligible
585	2021	Feeding patterns and BMI trajectories during infancy: a multi-ethnic, prospective birth cohort	BMC Pediatrics	Sirkka, O.	+	+	+	-			1			1	Ineligible
586	2021	Weight gain in early years and subsequent body mass index trajectories across birth weight groups: A prospective longitudinal study	European Journal of Public Health	Lu, Y.	+	+	+	-			1			1	Ineligible
587	2021	The relationship between wasting and stunting in Cambodian children: Secondary analysis of longitudinal data of children below 24 months of age followed up until the age of 59 months	PLOS One	Mutunga, M.	+	-	+	+			1	1			Ineligible
588	2021	Longitudinal associations between intake of fruit and vegetables and height attainment from preschool to school entry	International Journal of Environmental Research and Public Health	Rosário, R.	+	-	+	*			1			1	Ineligible
589	2021	Sociodemographic, biological and cultural factors affecting morbidities among infants: A longitudinal study in rural Karnataka	Clinical Epidemiology and Global Health	Mathad, V.	+	+	-	+			1			1	Ineligible
590	2021	Infant feeding and growth trajectories in early childhood: the application and comparison of two longitudinal modelling approaches	International Journal of Obesity	Zheng, M.	+	+	+	-			1			1	Ineligible
591	2021	Aflatoxin exposure was not associated with childhood stunting: Results from a birth cohort study in a resource-poor setting of Dhaka, Bangladesh	Public Health Nutrition	Mahfuz, M.	+	+	+	-			1			1	Ineligible
592	2021	Effect of Birth Interval on Foetal and Postnatal Child Growth	Scientifica	Saaka, M. & Aggrey, B.	+	+	+	-			1			1	Ineligible
593	2021	Prenatal dietary diversity may influence underweight in infants in a Ugandan birth-cohort	Maternal and Child Nutrition	Madzorera I..	+	+	+	-			1			1	Ineligible
594	2021	Body composition trajectories in HIV-exposed uninfected and HIV-unexposed uninfected infants in Western Kenya	Paediatric and Perinatal Epidemiology	Rickman, R.	+	+	+	-			1			1	Ineligible
595	2021	The effect of blood lipid profiles during pregnancy on longitudinal offspring health	Obesity Facts	Yelverton, C.	+	+	+	-			1	1			Ineligible
596	2021	RISK FACTORS for MORBIDITY and MORTALITY in VERY LOW BIRTH WEIGHT INFANTS from RURAL VERSUS URBAN COMMUNITIES	Journal of Investigative Medicine	Ondusko, DS.	+	*	*	-			1			1	Ineligible
597	2021	Cord blood Leptin as a predictor of age at adiposity rebound-the EDEN mother-child cohort	Obesity Facts	Cisse, A.	+	+	+	-			1			1	Ineligible
598	2021	Egyptian Mother's Breast Milk: Protein and Lipid Content in Correlation with Growth of Their Newborns	QJM	Shaaban, SY.	+	+	+	-			1			1	Ineligible
599	2021	Exclusivity of breastfeeding and body composition: learnings from the Baby-bod study	International Breastfeeding Journal	Jayasinghe, S.	+	+	+	-			1			1	Ineligible
600	2021	A longitudinal analysis of the effect of maternal region-of-birth on transitions in children's bodyweight status from early childhood to late adolescence in Australia: A population-based cohort study	Preventive Medicine	Hartono, S.	+	*	+	-			1			1	Ineligible
601	2022	Associations of maternal non-nutritive sweetener intake during pregnancy with offspring body mass index and body fat from birth to adolescence	International Journal of Obesity	Plows, JF.	+	+	+	-			1			1	Ineligible
602	2022	Play & Grow: prospective observational cohort of toddlers to inform obesity prevention, Columbus, Ohio, USA.	BMJ open	Parrott, A.	+	*	*	-			1			1	Ineligible

603	2022	COVID-19-Related Life Experiences, Outdoor Play, and Long-term Adiposity Changes Among Preschool- and School-Aged Children in Singapore 1 Year After Lockdown.	JAMA pediatrics	Sum KK, CS.	+	+	+	-			1			1	Ineligible
604	2022	Association of early migration with child growth, cognition and behaviour in South Africa.	Tropical medicine & international health	Christodoulou J.	+	*	+	-			1			1	Ineligible
605	2022	Influence of Maternal Childhood Trauma on Perinatal Depression, Observed Mother-Infant Interactions, and Child Growth.	Matern Child Health J.	Choi KW.	+	+	+	-			1			1	Ineligible
606	2022	Trends in Weight Gain Among Breastfed Infants Versus Bottle-Fed Infants at a Tertiary Care Hospital in Karachi, Pakistan.	Cureus.	Bai G	+	+	+	-			1			1	Ineligible
607	2022	Cesarean section and body mass index in children: is there a causal effect?	Cad Saude Publica.	Cavalcante LFP.	+	+	+	-			1			1	Ineligible
608	2022	Mediators of differences by parental education in weight-related outcomes in childhood and adolescence in Norway.	Sci Rep.	Mekonnen T.	+	+	+	-			1			1	Ineligible
609	2022	Early-life determinants of excessive weight gain among low-income children: Examining the adherence of theoretical frameworks to empirical data using structural equation modelling.	Pediatr Obes.	Dourado BLLFS	+	+	+	+			1			1	Weight gain
610	2022	Potentially modifiable mediators for socioeconomic disparities in childhood obesity in the United States.	Obesity (Silver Spring)	Wen X	+	-	+	+			1			1	Ineligible
611	2022	Linkage Between 2 Information Systems: Combined Live Births and Food and Nutrition Surveillance as a Public Health Tool for Investigation of the Determinants of Obesity Among Children and Adolescents in Southern Brazil.	Food Nutr Bull.	Mariante Giesta J	+	-	+	+			1			1	Ineligible
612	2022	Association between hyperglycaemia in pregnancy and growth of offspring in early childhood: The PANDORA study.	Pediatr Obes.	Titmuss A	+	+	+	-			1			1	Ineligible
613	2022	Secular Growth Trends in Early Childhood-Evidence from Two Low-Income Birth Cohorts Recruited over a Decade in Vellore, India.	Am J Trop Med Hyg.	Koshy B.	+	-	+	-			1			1	Ineligible
614	2022	Child feeding indexes measuring adherence to New Zealand nutrition guidelines: Development and assessment.	Matern Child Nutr.	Gontijo de Castro T.	+	+	+	-			1			1	Ineligible
615	2022	National, longitudinal NASCITA birth cohort study to investigate the health of Italian children and potential influencing factors.	BMJ Open.	Pandolfini C.	+	+	+	-			1			1	Ineligible
616	2022	Socio-economic determinants of child nutrition and growth: a longitudinal study in India.	IASSI-Quarterly	Gunjan G.	-	+	+	+			1			1	Ineligible
617	2022	The associations between stunting and wasting at 12 months of age and developmental milestones delays in a cohort of Cambodian children	Scientific Reports	Van Beekum M.	+	+	-	-			1			1	Ineligible
618	2022	Longitudinal Study of Growth Variation and Its Determinants in Body Weight of Children Aged 1-15 Years in Ethiopia	IRANIAN JOURNAL OF PEDIATRICS	Wake S.	-	+	+	+			1			1	Ineligible
619	2022	Factors Associated With Stunting of Children at 24 Months in the AMANHI Pakistan Study	Current Developments in Nutrition	Khan DS	+	+	+	+			1			1	Insufficient data
620	2022	Factors Associated with Malnutrition among Children Aged Six Months to Five Years in a Semi-Rural Area of the Western Cape, South Africa	Child Care in Practice	Loots R.	-	+	+	+			1			1	Ineligible

621	2022	A LOGISTIC REGRESSION ANALYSIS OF DETERMINANTS OF CHILD MALNUTRITION IN UTTAR PRADESH, INDIA	Journal of Community Positive Practices	Kumari R.	-	+	+	+			1			1	Ineligible
622	2022	Infant appetite and weight gain in early infancy: Moderating effects of controlling feeding styles	Appetite	Shriver LH.	+	-	+	+			1			1	Ineligible¶
623	2023	Parental determinants associated with early growth after the first year of life by race and ethnicity.	Front Pediatr.	Aguayo L.	+	+	+	+			1			1	Insufficient data
624	2023	Linking infant size and early growth with maternal lifestyle and breastfeeding - the first year of life in the CRIBS cohort.	Ann Hum Biol.	Šarac J	+	+	+	+			1			1	Continuous Education
625	2023	Association of Maternal Education With Nutritional Outcomes of Poor Children With Stunting in Indonesia.	Asia Pac J Public Health.	Handayani NS	+	-	+	+			1			1	Ineligible
626	2023	Mapping Concurrent Wasting and Stunting Among Children Under Five in India: A Multilevel Analysis.	Int J Public Health.	Khura B.	-	+	+	+			1			1	Ineligible
627	2023	Associations between primary healthcare and infant health outcomes: a cohort analysis of low-income mothers in Rio de Janeiro, Brazil.	Lancet Reg Health Am.	Hone T.	+	+	-	-			1			1	Ineligible
628	2023	Maternal depressiveness and infant growth outcomes: Findings from the MAASTHI cohort study in India.	J Psychosom Res.	Shriyan P.	+	+	+	-			1			1	Ineligible
629	2023	Longitudinal trends and determinants of stunting among children aged 1-15 years.	Arch Public Health.	Wake SK	+	-	+	+			1			1	Ineligible
630	2023	African American mothers' perceptions of infant body size are prospectively associated with infant growth and BMI.	Am J Hum Biol.	Rothman J.	+	+	+	-			1			1	Ineligible
631	2023	National, longitudinal NASCITA birth cohort study: prevalence of overweight at 12 months of age in children born healthy.	BMJ Paediatr Open.	Clavenna A.	+	+	+	-			1			1	Ineligible
632	2023	Influence of maternal age on birth and infant outcomes at 6 months: a cohort study with quantitative bias analysis.	Int J Epidemiol.	Gebreegziabher E.	+	+	+	-			1			1	Ineligible
633	2023	Cardiometabolic risk profile among children with migrant parents and role of parental education: the IDEFICS/I.Family cohort.	Int J Obes (Lond)	Lindblad A.	+	+	+	-			1			1	Ineligible
634	2023	Association between sleep duration and latency, nocturnal awakenings, and body mass index among infants.	Rev Paul Pediatr.	Oliveira PMB	+	+	+	-			1			1	Ineligible
635	2023	Ultra-Processed Food as Mediator of the Association between Birthweight and Childhood Body Weight Outcomes: A Retrospective Cohort Study	Nutrients	Hou M.	+	-	+	+			1			1	Ineligible
636	2023	Relationship between Birth Order and Postnatal Growth until 4 Years of Age: The Japan Environment and Children's Study	Children	Yoshida A.	+	+	+	-			1			1	Ineligible
637	2023	Infant body composition at 6 and 24 months: what are the driving factors?	European Journal of Clinical Nutrition	Santos IS.	+	+	+	-			1			1	Ineligible
638	2023	Association between Meal Frequency and Weight Status in Spanish Children: A Prospective Cohort Study	Nutrients	Juton C.	+	+	+	-			1			1	Ineligible
639	2023	Association and potential mediators between socioeconomic status and childhood obesity in China: findings from a national cohort study	Global Health Journal	Zhou H.	+	-	+	+			1			1	Ineligible
640	2024	Obesity Prevention in Early Life (OPEL) study: linking longitudinal data to capture obesity risk in the first 1000 days	BMJ NUTRITION, PREVENTION \& HEALTH	Cheng E.	+	+	+	-			1			1	Ineligible

*I: Include; B: Borderline; E: Exclude.

¶ The authors calculated rapid weight gain between birth and various child ages (ranging from 6 to 11 months), employing a methodology similar to that of cross-sectional studies.
