# Supplementary material for: Maternal education and its influence on child growth and nutritional status during the first two years of life: a systematic review and meta-analysis
Source: eClinicalMedicine. 2024 Apr 4;71:102574. doi: 10.1016/j.eclinm.2024.102574 (PMC11001623; doi:10.1016/j.eclinm.2024.102574)
Supplement: Supplementary File 5 [file mmc5.pdf]

Supplementary 5. *Quality assessment of included studies using Newcastle-Ottawa scale adapted for cohort studies.*

Indicator	***Author***	***Selection***				***Comparability***	***Outcome***			***Quality***
		**1.** Representativeness of the exposed cohort	**2.** Selection of the non-exposed cohort	**3.** Ascertainment of exposure	**4.** Demonstration that outcome of interest was not present at start of study	**1.** Comparability of cohorts on the basis of the design or analysis	**1.** Assessment of outcome	**2.** Was follow up long enough for outcomes to occur	**3.** Adequacy of follow up of cohorts	
*WAZ**	Grjibovski AM	1	1	1	1	2	1	1	1	good
	Mesman I	1	1	0	0	0	1	1	0	poor
	Hui LL	1	1	0	0	2	2	1	1	good
	van Rossem L	1	1	1	0	0	1	1	0	poor
	Chen YJ	1	1	1	1	2	1	1	0	good
	Hong SA	1	1	1	0	1	1	1	1	good
	Kachi Y,2001	1	1	0	0	0	1	1	0	poor
	Kachi Y,2011	1	1	0	0	0	1	1	0	poor
	Ballon M	1	1	0	0	1	1	1	1	fair
	Mekonnen T	1	1	0	0	2	1	1	0	fair
*HAZ**	Grjibovski AM	1	1	1	1	2	1	1	1	good
	Mesman I	1	1	0	0	2	1	1	0	good
	van Rossem L	1	1	1	0	0	1	1	0	poor
	Matijasevich A	1	1	1	1	0	1	1	1	poor
	Howe LD	1	1	0	0	0	1	1	1	poor
	Silva LM	1	1	1	0	2	1	1	0	good
	Chen YJ	1	1	1	1	2	1	1	1	good
	Hong SA	1	1	1	0	1	1	1	1	good
	Ballon M	1	1	0	0	1	1	1	1	fair
	Dal Bom JP	1	1	1	0	1	1	1	0	fair
	Mekonnen T	1	1	0	0	2	1	1	0	fair
	Diana A	1	1	1	1	2	1	1	1	good
*BMIZ**	Howe LD	1	1	0	0	0	2	1	0	poor
	Mesman I	1	1	0	0	0	1	1	0	poor
	van Rossem L	1	1	1	0	2	1	1	0	good
	Silva LM	1	1	1	0	0	1	1	1	poor
	Morgen CS	1	1	1	0	0	1	1	1	poor
	Ballon M	1	1	0	0	1	1	1	1	fair
	McCrory C, G21	1	1	1	0	0	1	1	1	poor
	McCrory C(GUI)	1	1	1	0	0	1	1	1	poor
	Dal Bom JP	1	1	1	0	1	1	1	0	fair
	Mekonnen T	1	1	0	0	2	1	1	0	fair
Overweight	van Rossem L	1	1	1	0	2	1	1	0	good
	Anderson SE	1	1	0	0	0	1	1	0	poor
	Mastroeni MF	0	1	1	0	2	1	1	0	fair
	Feldman-Winter L	0	1	1	0	0	1	1	0	poor
	Ballon M	1	1	0	0	1	1	1	1	fair
	Rotevatn TA	1	1	1	0	2	1	1	0	good
	Shay M	1	1	0	0	0	0	1	1	poor
	Xinmei Chen	1	1	0	0	0	1	1	1	poor
	Mekonnen T	1	1	0	0	2	1	1	0	poor
	Zhou S	0	1	1	0	0	1	1	0	poor
Underweight	Mamabolo RL	0	1	1	0	0	1	1	0	poor
	Wightkin J	1	1	1	0	0	1	1	0	poor
	Mondal D	0	1	1	1	2	1	1	1	good
	Hong SA	1	1	1	0	1	1	1	1	good
Stunting	Mamabolo RL	0	1	2	0	0	1	1	0	poor
	Willey, Barbara A	1	1	1	1	2	1	1	0	good
	LL Jones	1	1	0	1	2	1	1	0	fair
	Mongkolchati A	1	1	1	1	2	1	1	1	good
	Mondal D	0	1	1	0	2	1	1	1	good
	Slemming W.	1	1	1	0	0	1	1	0	poor
	Hong SA	1	1	1	0	1	1	1	1	good
	Das S	0	1	1	0	0	1	1	1	poor

*W**	Mamabolo RL	0	1	1	0	0	1	1	0	poor
	Hong SA	1	1	1	0	1	1	1	1	good
*RWG**	Karaolis-Danckert	0	1	1	1	0	1	1	0	poor
	Mendez MA	1	1	1	1	0	1	1	1	poor
	Criswell R	0	1	0	1	0	0	1	1	poor
	Rotevatn TA	1	1	1	1	0	1	1	0	poor

** The numbers represent the number of stars assigned to each domain. WAZ: Weight for age z-score; HAZ: Height for age z-score; BMIZ: BMI for age z-score; W: Wasting; RWG: Rapid weight gain.*
