# Supplementary material for: Maternal education and its influence on child growth and nutritional status during the first two years of life: a systematic review and meta-analysis
Source: eClinicalMedicine. 2024 Apr 4;71:102574. doi: 10.1016/j.eclinm.2024.102574 (PMC11001623; doi:10.1016/j.eclinm.2024.102574)
Supplement: Supplementary File 6 [file mmc6.pdf]

Supplementary 6. The subgroup analysis was performed by A. country income group, B. education status of the country, C. loss to follow-up rate and D. study quality, for evaluating the effect of high vs. low maternal education level on child weight for age z-score (WAZ); height for age z-score (HAZ) ; BMI for age z-scores (BMI Z-score); overweight and E.stunting. Mean difference (MD) and odds ratio (OR) were used as the effect sizes.

➢ WAZ

A

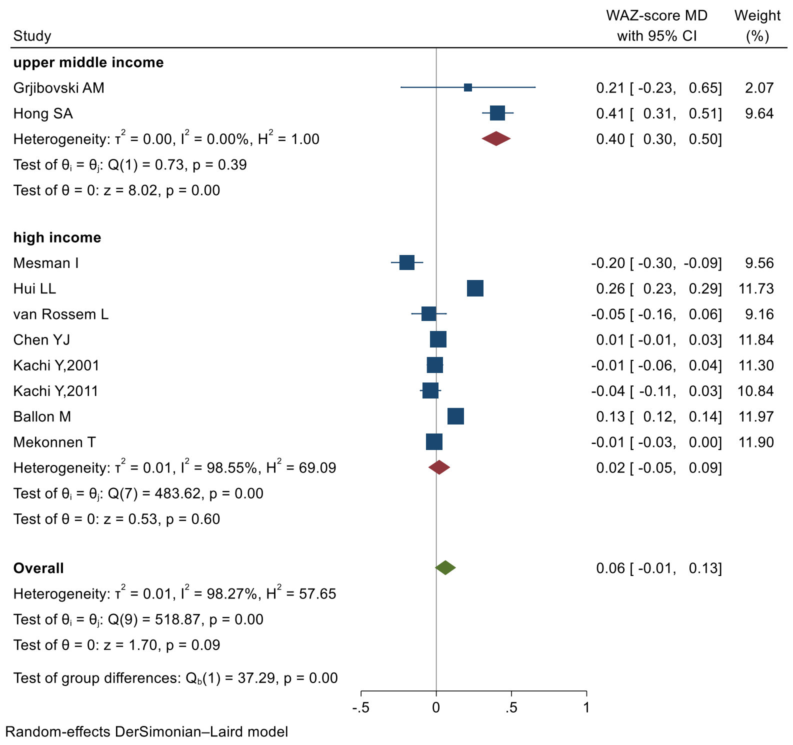

B

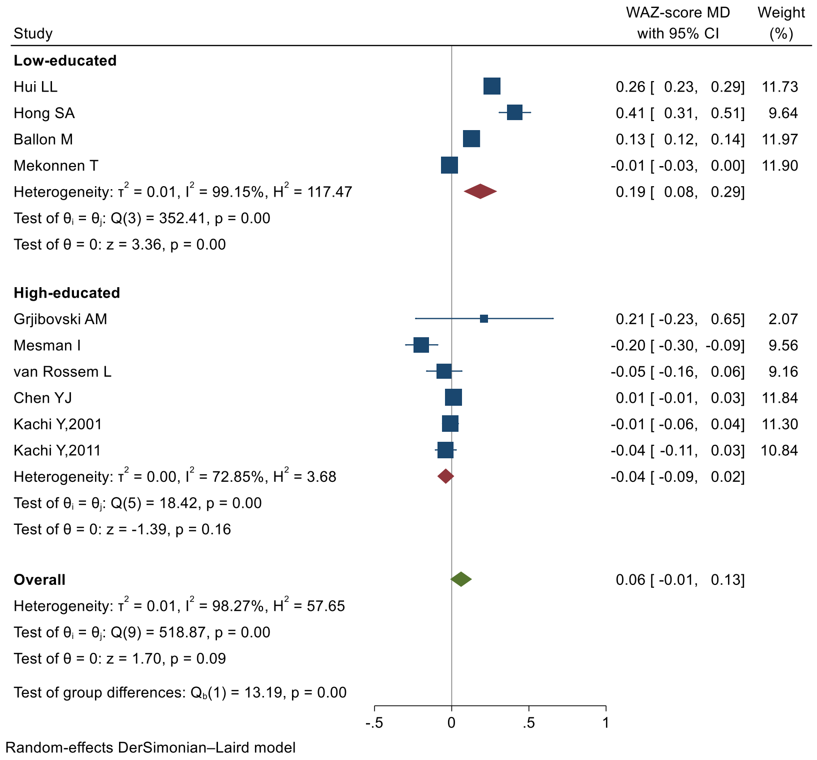

C

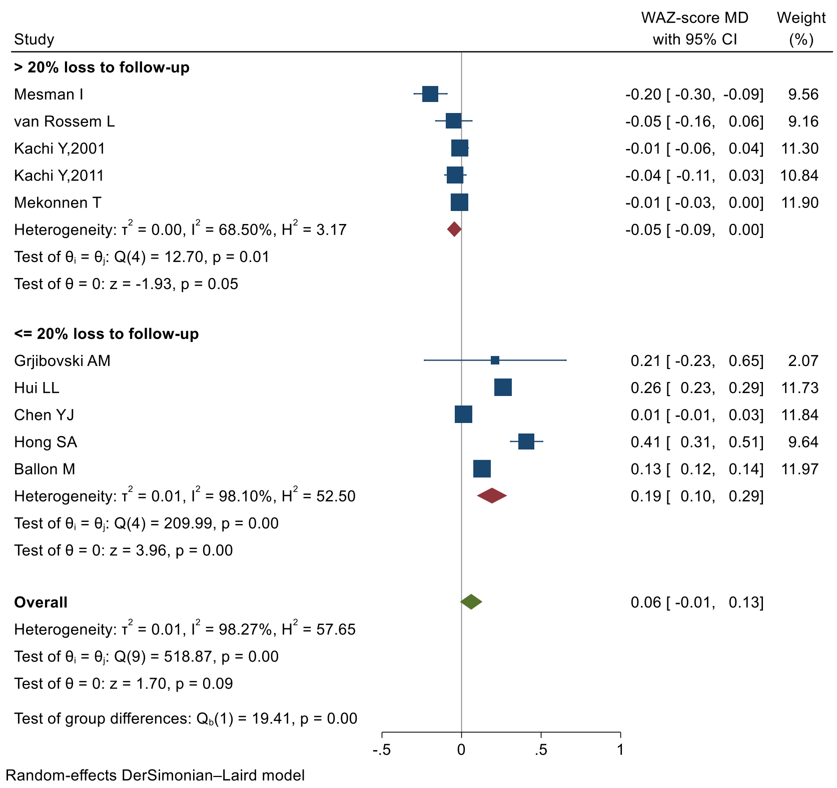

Study
WAZ-score MD with 95% CI
Weight (%)
> 20% loss to follow-up
Mesman I -0.20 [-0.30, -0.09] 9.56
van Rossem L -0.05 [-0.16, 0.06] 9.16
Kachi Y,2001 -0.01 [-0.06, 0.04] 11.30
Kachi Y,2011 -0.04 [-0.11, 0.03] 10.84
Mekonnen T -0.01 [-0.03, 0.00] 11.90
Heterogeneity: τ² = 0.00, I² = 68.50%, H² = 3.17 -0.05 [-0.09, 0.00]
Test of θi = θj: Q(4) = 12.70, p = 0.01
Test of θ = 0: z = -1.93, p = 0.05
<= 20% loss to follow-up
Grjibovski AM 0.21 [-0.23, 0.65] 2.07
Hui LL 0.26 [0.23, 0.29] 11.73
Chen YJ 0.01 [-0.01, 0.03] 11.84
Hong SA 0.41 [0.31, 0.51] 9.64
Ballon M 0.13 [0.12, 0.14] 11.97
Heterogeneity: τ² = 0.01, I² = 98.10%, H² = 52.50 0.19 [0.10, 0.29]
Test of θi = θj: Q(4) = 209.99, p = 0.00
Test of θ = 0: z = 3.96, p = 0.00
Overall 0.06 [-0.01, 0.13]
Heterogeneity: τ² = 0.01, I² = 98.27%, H² = 57.65
Test of θi = θj: Q(9) = 518.87, p = 0.00
Test of θ = 0: z = 1.70, p = 0.09
Test of group differences: Qb(1) = 19.41, p = 0.00
-.5 0 .5 1
Random-effects DerSimonian–Laird model

D

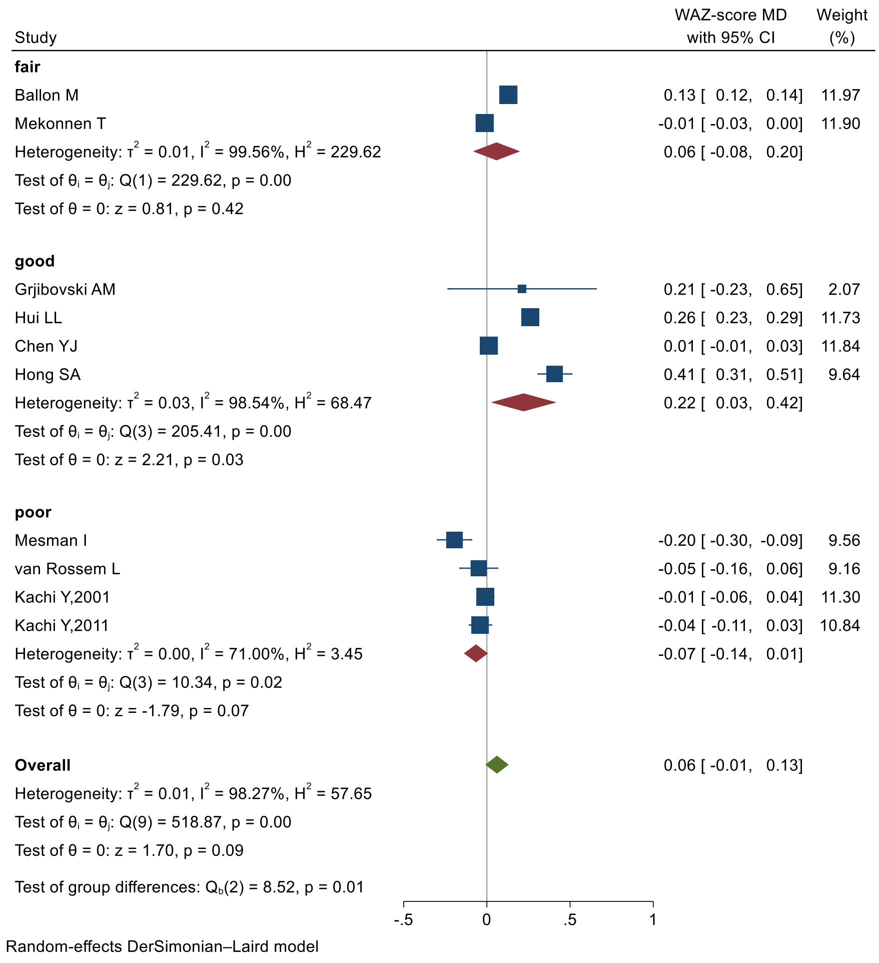

Study
WAZ-score MD with 95% CI
Weight (%)
fair
Ballon M 0.13 [0.12, 0.14] 11.97
Mekonnen T -0.01 [-0.03, 0.00] 11.90
Heterogeneity: τ² = 0.01, I² = 99.56%, H² = 229.62 0.06 [-0.08, 0.20]
Test of θi = θj: Q(1) = 229.62, p = 0.00
Test of θ = 0: z = 0.81, p = 0.42
good
Grjibovski AM 0.21 [-0.23, 0.65] 2.07
Hui LL 0.26 [0.23, 0.29] 11.73
Chen YJ 0.01 [-0.01, 0.03] 11.84
Hong SA 0.41 [0.31, 0.51] 9.64
Heterogeneity: τ² = 0.03, I² = 98.54%, H² = 68.47 0.22 [0.03, 0.42]
Test of θi = θj: Q(3) = 205.41, p = 0.00
Test of θ = 0: z = 2.21, p = 0.03
poor
Mesman I -0.20 [-0.30, -0.09] 9.56
van Rossem L -0.05 [-0.16, 0.06] 9.16
Kachi Y,2001 -0.01 [-0.06, 0.04] 11.30
Kachi Y,2011 -0.04 [-0.11, 0.03] 10.84
Heterogeneity: τ² = 0.00, I² = 71.00%, H² = 3.45 -0.07 [-0.14, 0.01]
Test of θi = θj: Q(3) = 10.34, p = 0.02
Test of θ = 0: z = -1.79, p = 0.07
Overall 0.06 [-0.01, 0.13]
Heterogeneity: τ² = 0.01, I² = 98.27%, H² = 57.65
Test of θi = θj: Q(9) = 518.87, p = 0.00
Test of θ = 0: z = 1.70, p = 0.09
Test of group differences: Qb(2) = 8.52, p = 0.01
-.5 0 .5 1
Random-effects DerSimonian–Laird model

➢ HAZ

A

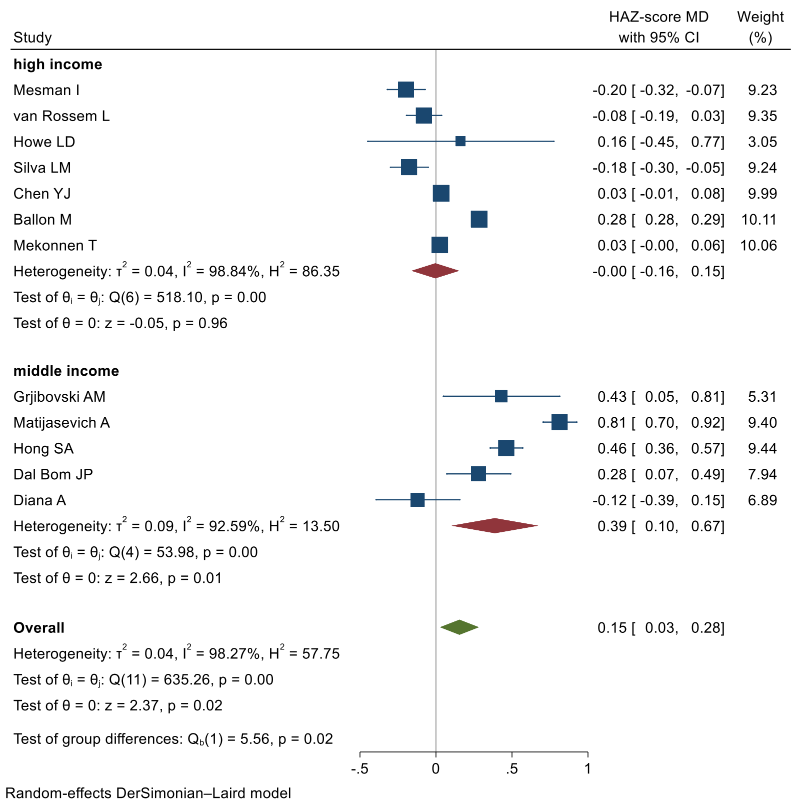
Study
HAZ-score MD with 95% CI
Weight (%)
high income
Mesman I -0.20 [-0.32, -0.07] 9.23
van Rossem L -0.08 [-0.19, 0.03] 9.35
Howe LD 0.16 [-0.45, 0.77] 3.05
Silva LM -0.18 [-0.30, -0.05] 9.24
Chen YJ 0.03 [-0.01, 0.08] 9.99
Ballon M 0.28 [0.28, 0.29] 10.11
Mekonnen T 0.03 [-0.00, 0.06] 10.06
Heterogeneity: τ² = 0.04, I² = 98.84%, H² = 86.35 -0.00 [-0.16, 0.15]
Test of θᵢ = θⱼ: Q(6) = 518.10, p = 0.00
Test of θ = 0: z = -0.05, p = 0.96
middle income
Grjibovski AM 0.43 [0.05, 0.81] 5.31
Matijasevich A 0.81 [0.70, 0.92] 9.40
Hong SA 0.46 [0.36, 0.57] 9.44
Dal Bom JP 0.28 [0.07, 0.49] 7.94
Diana A -0.12 [-0.39, 0.15] 6.89
Heterogeneity: τ² = 0.09, I² = 92.59%, H² = 13.50 0.39 [0.10, 0.67]
Test of θᵢ = θⱼ: Q(4) = 53.98, p = 0.00
Test of θ = 0: z = 2.66, p = 0.01
Overall 0.15 [0.03, 0.28]
Heterogeneity: τ² = 0.04, I² = 98.27%, H² = 57.75
Test of θᵢ = θⱼ: Q(11) = 635.26, p = 0.00
Test of θ = 0: z = 2.37, p = 0.02
Test of group differences: Q_b(1) = 5.56, p = 0.02
-.5
0
.5
1
Random-effects DerSimonian–Laird model

B

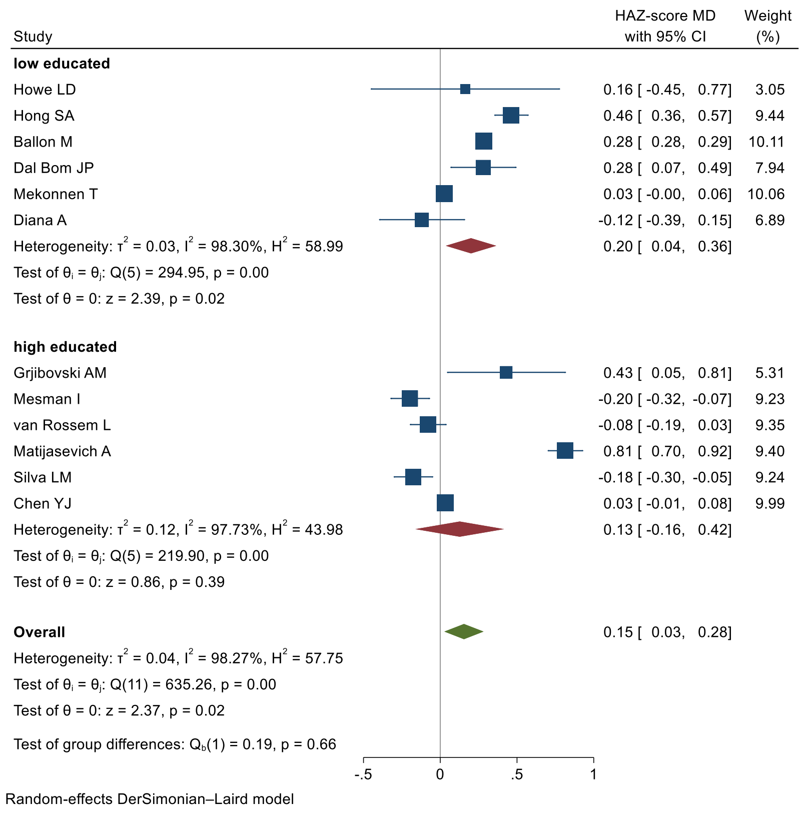
Study
HAZ-score MD with 95% CI
Weight (%)
low educated
Howe LD 0.16 [-0.45, 0.77] 3.05
Hong SA 0.46 [0.36, 0.57] 9.44
Ballon M 0.28 [0.28, 0.29] 10.11
Dal Bom JP 0.28 [0.07, 0.49] 7.94
Mekonnen T 0.03 [-0.00, 0.06] 10.06
Diana A -0.12 [-0.39, 0.15] 6.89
Heterogeneity: τ² = 0.03, I² = 98.30%, H² = 58.99 0.20 [0.04, 0.36]
Test of θᵢ = θⱼ: Q(5) = 294.95, p = 0.00
Test of θ = 0: z = 2.39, p = 0.02
high educated
Grjibovski AM 0.43 [0.05, 0.81] 5.31
Mesman I -0.20 [-0.32, -0.07] 9.23
van Rossem L -0.08 [-0.19, 0.03] 9.35
Matijasevich A 0.81 [0.70, 0.92] 9.40
Silva LM -0.18 [-0.30, -0.05] 9.24
Chen YJ 0.03 [-0.01, 0.08] 9.99
Heterogeneity: τ² = 0.12, I² = 97.73%, H² = 43.98 0.13 [-0.16, 0.42]
Test of θᵢ = θⱼ: Q(5) = 219.90, p = 0.00
Test of θ = 0: z = 0.86, p = 0.39
Overall 0.15 [0.03, 0.28]
Heterogeneity: τ² = 0.04, I² = 98.27%, H² = 57.75
Test of θᵢ = θⱼ: Q(11) = 635.26, p = 0.00
Test of θ = 0: z = 2.37, p = 0.02
Test of group differences: Q_b(1) = 0.19, p = 0.66
-.5
0
.5
1
Random-effects DerSimonian–Laird model

C

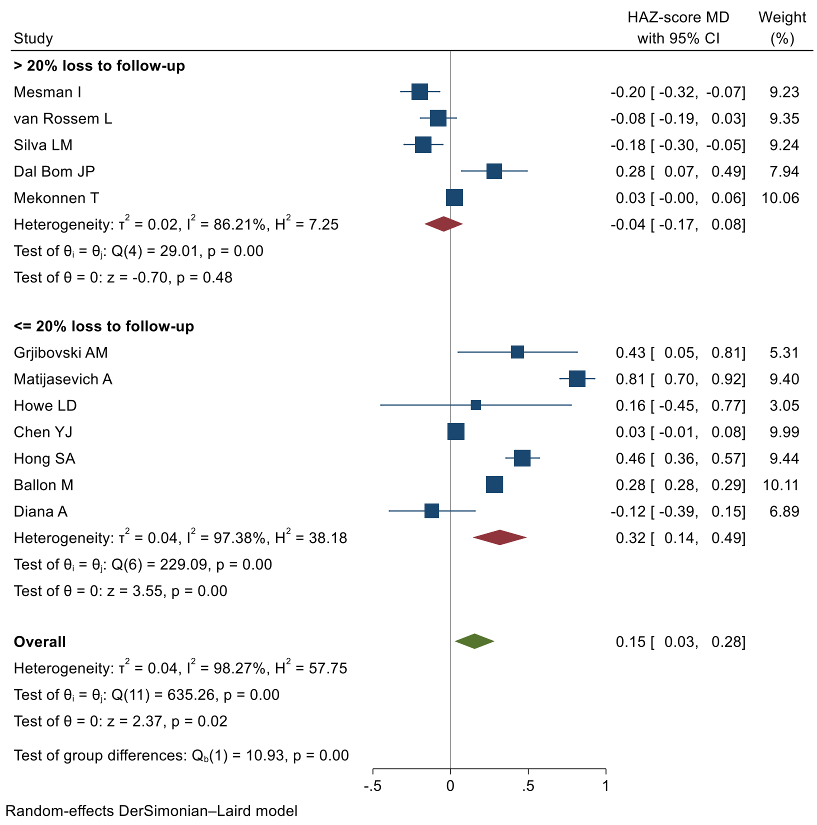

Study
HAZ-score MD with 95% CI
Weight (%)
> 20% loss to follow-up
Mesman I -0.20 [-0.32, -0.07] 9.23
van Rossem L -0.08 [-0.19, 0.03] 9.35
Silva LM -0.18 [-0.30, -0.05] 9.24
Dal Bom JP 0.28 [0.07, 0.49] 7.94
Mekonnen T 0.03 [-0.00, 0.06] 10.06
Heterogeneity: τ^2 = 0.02, I^2 = 86.21%, H^2 = 7.25 -0.04 [-0.17, 0.08]
Test of $\theta_i = \theta_j$: Q(4) = 29.01, p = 0.00
Test of θ = 0: z = -0.70, p = 0.48
<= 20% loss to follow-up
Grjibovski AM 0.43 [0.05, 0.81] 5.31
Matijasevich A 0.81 [0.70, 0.92] 9.40
Howe LD 0.16 [-0.45, 0.77] 3.05
Chen YJ 0.03 [-0.01, 0.08] 9.99
Hong SA 0.46 [0.36, 0.57] 9.44
Ballon M 0.28 [0.28, 0.29] 10.11
Diana A -0.12 [-0.39, 0.15] 6.89
Heterogeneity: τ^2 = 0.04, I^2 = 97.38%, H^2 = 38.18 0.32 [0.14, 0.49]
Test of $\theta_i = \theta_j$: Q(6) = 229.09, p = 0.00
Test of θ = 0: z = 3.55, p = 0.00
Overall 0.15 [0.03, 0.28]
Heterogeneity: τ^2 = 0.04, I^2 = 98.27%, H^2 = 57.75
Test of $\theta_i = \theta_j$: Q(11) = 635.26, p = 0.00
Test of θ = 0: z = 2.37, p = 0.02
Test of group differences: $Q_b(1)$ = 10.93, p = 0.00
-.5
0
.5
1
Random-effects DerSimonian–Laird model

D

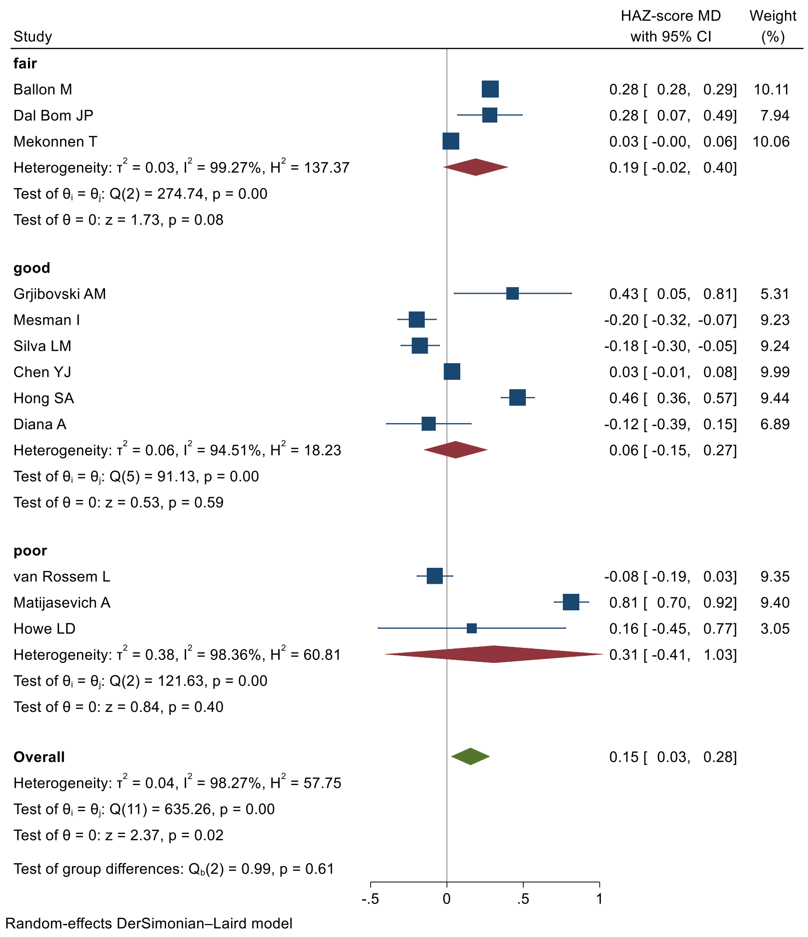

Study
HAZ-score MD with 95% CI
Weight (%)
fair
Ballon M 0.28 [0.28, 0.29] 10.11
Dal Bom JP 0.28 [0.07, 0.49] 7.94
Mekonnen T 0.03 [-0.00, 0.06] 10.06
Heterogeneity: τ^2 = 0.03, I^2 = 99.27%, H^2 = 137.37 0.19 [-0.02, 0.40]
Test of $\theta_i = \theta_j$: Q(2) = 274.74, p = 0.00
Test of θ = 0: z = 1.73, p = 0.08
good
Grjibovski AM 0.43 [0.05, 0.81] 5.31
Mesman I -0.20 [-0.32, -0.07] 9.23
Silva LM -0.18 [-0.30, -0.05] 9.24
Chen YJ 0.03 [-0.01, 0.08] 9.99
Hong SA 0.46 [0.36, 0.57] 9.44
Diana A -0.12 [-0.39, 0.15] 6.89
Heterogeneity: τ^2 = 0.06, I^2 = 94.51%, H^2 = 18.23 0.06 [-0.15, 0.27]
Test of $\theta_i = \theta_j$: Q(5) = 91.13, p = 0.00
Test of θ = 0: z = 0.53, p = 0.59
poor
van Rossem L -0.08 [-0.19, 0.03] 9.35
Matijasevich A 0.81 [0.70, 0.92] 9.40
Howe LD 0.16 [-0.45, 0.77] 3.05
Heterogeneity: τ^2 = 0.38, I^2 = 98.36%, H^2 = 60.81 0.31 [-0.41, 1.03]
Test of $\theta_i = \theta_j$: Q(2) = 121.63, p = 0.00
Test of θ = 0: z = 0.84, p = 0.40
Overall 0.15 [0.03, 0.28]
Heterogeneity: τ^2 = 0.04, I^2 = 98.27%, H^2 = 57.75
Test of $\theta_i = \theta_j$: Q(11) = 635.26, p = 0.00
Test of θ = 0: z = 2.37, p = 0.02
Test of group differences: $Q_b(2)$ = 0.99, p = 0.61
-.5
0
.5
1
Random-effects DerSimonian–Laird model

➢ BMI Z-score

A

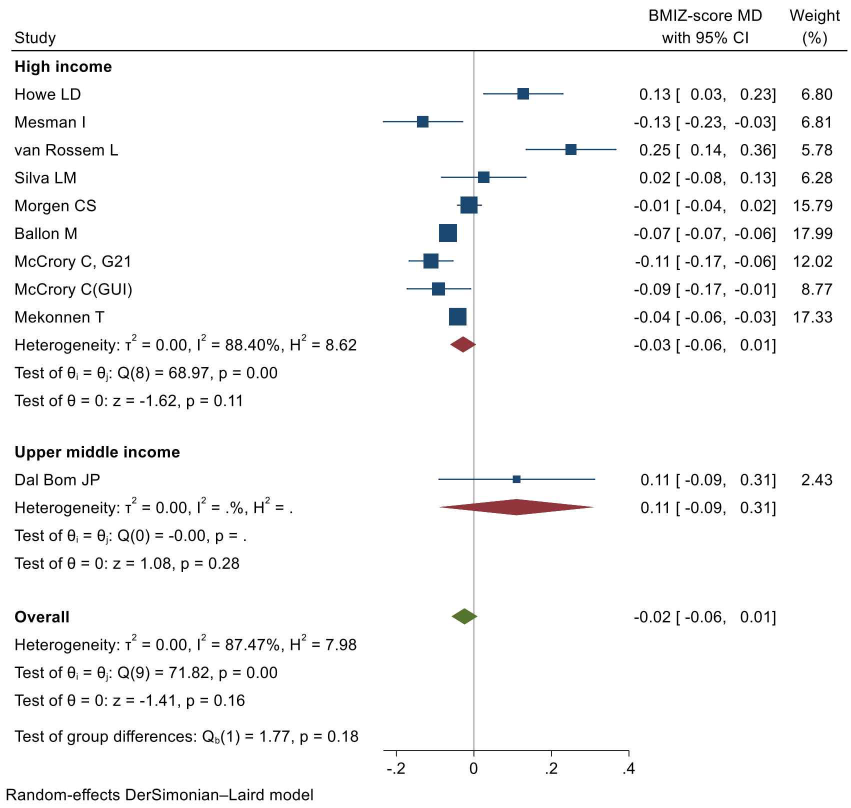

B

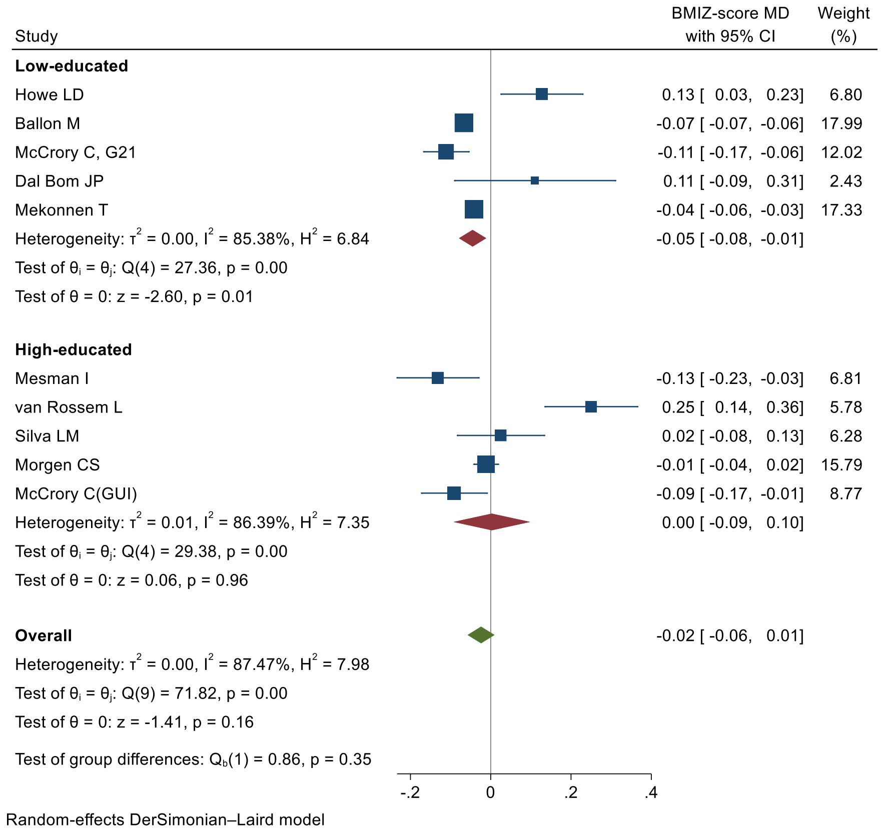

C

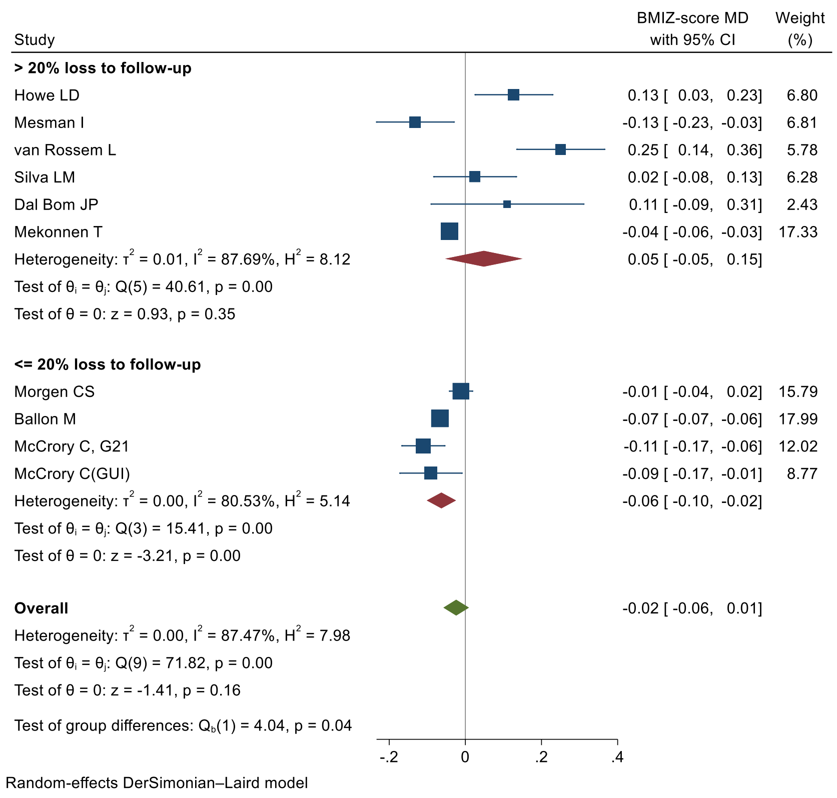

BMIZ-score MD with 95% CI
Weight (%)
Study
> 20% loss to follow-up
Howe LD 0.13 [0.03, 0.23] 6.80
Mesman I -0.13 [-0.23, -0.03] 6.81
van Rossem L 0.25 [0.14, 0.36] 5.78
Silva LM 0.02 [-0.08, 0.13] 6.28
Dal Bom JP 0.11 [-0.09, 0.31] 2.43
Mekonnen T -0.04 [-0.06, -0.03] 17.33
Heterogeneity: τ² = 0.01, I² = 87.69%, H² = 8.12 0.05 [-0.05, 0.15]
Test of θi = θj: Q(5) = 40.61, p = 0.00
Test of θ = 0: z = 0.93, p = 0.35
<= 20% loss to follow-up
Morgen CS -0.01 [-0.04, 0.02] 15.79
Ballon M -0.07 [-0.07, -0.06] 17.99
McCrory C, G21 -0.11 [-0.17, -0.06] 12.02
McCrory C(GUI) -0.09 [-0.17, -0.01] 8.77
Heterogeneity: τ² = 0.00, I² = 80.53%, H² = 5.14 -0.06 [-0.10, -0.02]
Test of θi = θj: Q(3) = 15.41, p = 0.00
Test of θ = 0: z = -3.21, p = 0.00
Overall -0.02 [-0.06, 0.01]
Heterogeneity: τ² = 0.00, I² = 87.47%, H² = 7.98
Test of θi = θj: Q(9) = 71.82, p = 0.00
Test of θ = 0: z = -1.41, p = 0.16
Test of group differences: Qb(1) = 4.04, p = 0.04
-.2
0
.2
.4
Random-effects DerSimonian–Laird model

D

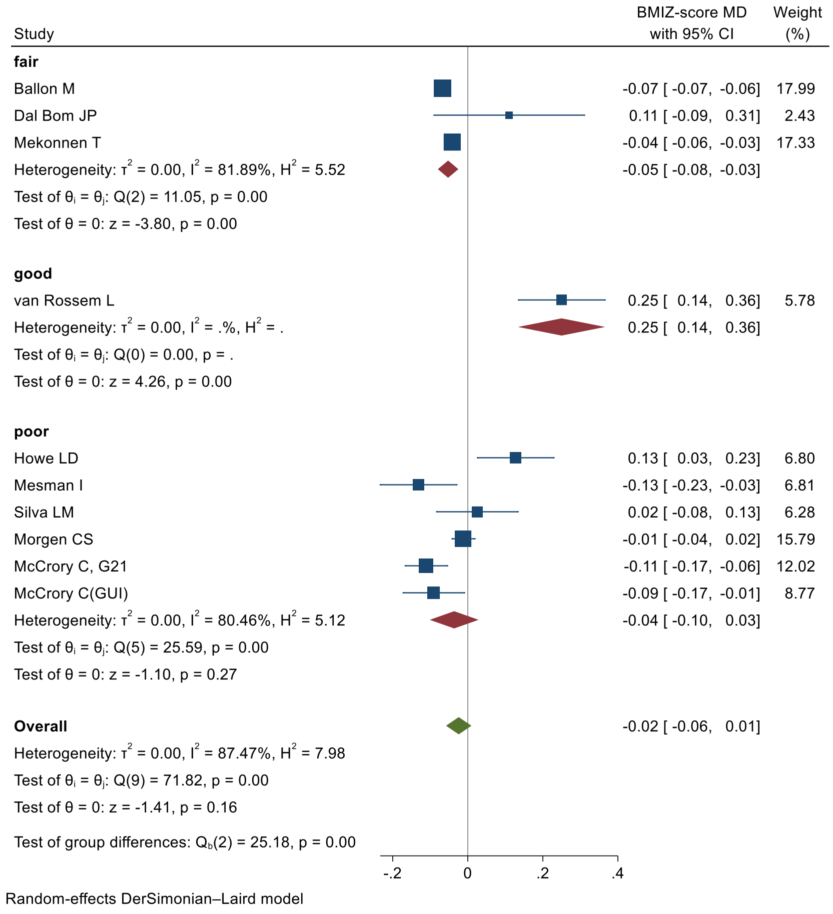

BMIZ-score MD with 95% CI
Weight (%)
Study
fair
Ballon M -0.07 [-0.07, -0.06] 17.99
Dal Bom JP 0.11 [-0.09, 0.31] 2.43
Mekonnen T -0.04 [-0.06, -0.03] 17.33
Heterogeneity: τ² = 0.00, I² = 81.89%, H² = 5.52 -0.05 [-0.08, -0.03]
Test of θi = θj: Q(2) = 11.05, p = 0.00
Test of θ = 0: z = -3.80, p = 0.00
good
van Rossem L 0.25 [0.14, 0.36] 5.78
Heterogeneity: τ² = 0.00, I² = .%, H² = . 0.25 [0.14, 0.36]
Test of θi = θj: Q(0) = 0.00, p = .
Test of θ = 0: z = 4.26, p = 0.00
poor
Howe LD 0.13 [0.03, 0.23] 6.80
Mesman I -0.13 [-0.23, -0.03] 6.81
Silva LM 0.02 [-0.08, 0.13] 6.28
Morgen CS -0.01 [-0.04, 0.02] 15.79
McCrory C, G21 -0.11 [-0.17, -0.06] 12.02
McCrory C(GUI) -0.09 [-0.17, -0.01] 8.77
Heterogeneity: τ² = 0.00, I² = 80.46%, H² = 5.12 -0.04 [-0.10, 0.03]
Test of θi = θj: Q(5) = 25.59, p = 0.00
Test of θ = 0: z = -1.10, p = 0.27
Overall -0.02 [-0.06, 0.01]
Heterogeneity: τ² = 0.00, I² = 87.47%, H² = 7.98
Test of θi = θj: Q(9) = 71.82, p = 0.00
Test of θ = 0: z = -1.41, p = 0.16
Test of group differences: Qb(2) = 25.18, p = 0.00
-.2
0
.2
.4
Random-effects DerSimonian–Laird model

➢ Overweight

A

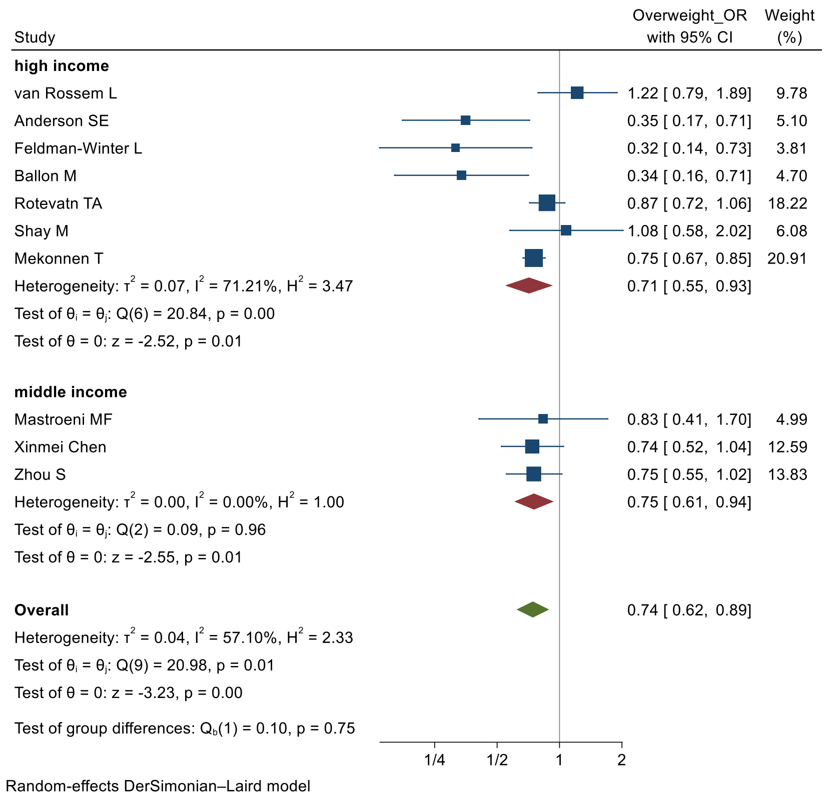

B

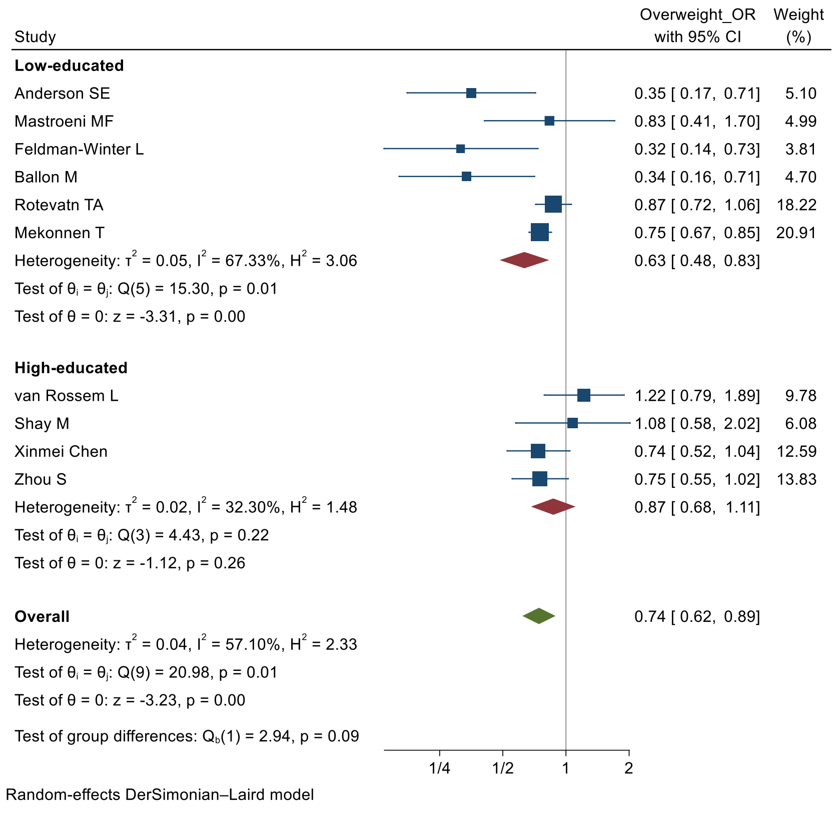

C

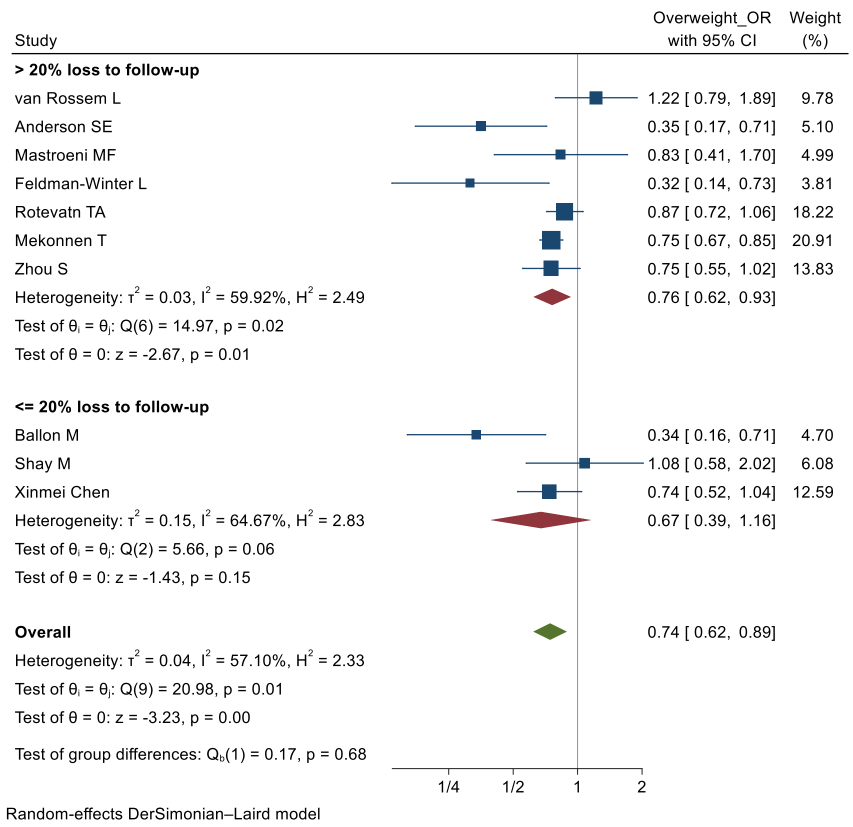
Study
Overweight_OR with 95% CI
Weight (%)
> 20% loss to follow-up
van Rossem L 1.22 [0.79, 1.89] 9.78
Anderson SE 0.35 [0.17, 0.71] 5.10
Mastroeni MF 0.83 [0.41, 1.70] 4.99
Feldman-Winter L 0.32 [0.14, 0.73] 3.81
Rotevatn TA 0.87 [0.72, 1.06] 18.22
Mekonnen T 0.75 [0.67, 0.85] 20.91
Zhou S 0.75 [0.55, 1.02] 13.83
Heterogeneity: τ^2 = 0.03, I^2 = 59.92%, H^2 = 2.49 0.76 [0.62, 0.93]
Test of $\theta_i = \theta_j$: Q(6) = 14.97, p = 0.02
Test of θ = 0: z = -2.67, p = 0.01
<= 20% loss to follow-up
Ballon M 0.34 [0.16, 0.71] 4.70
Shay M 1.08 [0.58, 2.02] 6.08
Xinmei Chen 0.74 [0.52, 1.04] 12.59
Heterogeneity: τ^2 = 0.15, I^2 = 64.67%, H^2 = 2.83 0.67 [0.39, 1.16]
Test of $\theta_i = \theta_j$: Q(2) = 5.66, p = 0.06
Test of θ = 0: z = -1.43, p = 0.15
Overall 0.74 [0.62, 0.89]
Heterogeneity: τ^2 = 0.04, I^2 = 57.10%, H^2 = 2.33
Test of $\theta_i = \theta_j$: Q(9) = 20.98, p = 0.01
Test of θ = 0: z = -3.23, p = 0.00
Test of group differences: $Q_b(1)$ = 0.17, p = 0.68
1/4
1/2
1
2
Random-effects DerSimonian–Laird model

D

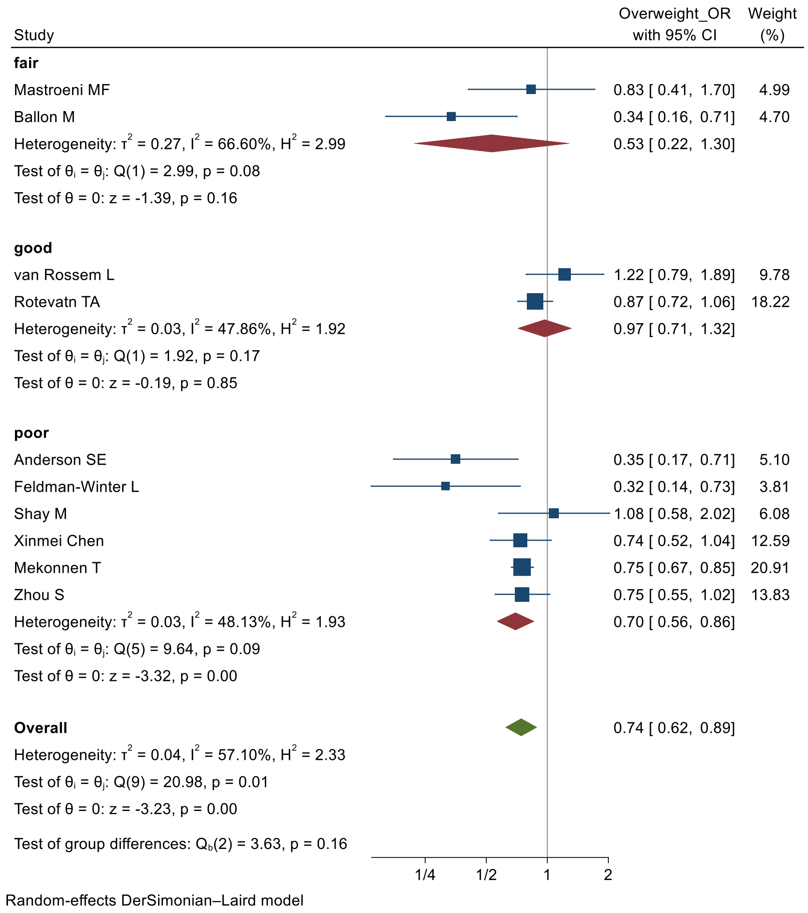
Study
Overweight_OR with 95% CI
Weight (%)
fair
Mastroeni MF 0.83 [0.41, 1.70] 4.99
Ballon M 0.34 [0.16, 0.71] 4.70
Heterogeneity: τ^2 = 0.27, I^2 = 66.60%, H^2 = 2.99 0.53 [0.22, 1.30]
Test of $\theta_i = \theta_j$: Q(1) = 2.99, p = 0.08
Test of θ = 0: z = -1.39, p = 0.16
good
van Rossem L 1.22 [0.79, 1.89] 9.78
Rotevatn TA 0.87 [0.72, 1.06] 18.22
Heterogeneity: τ^2 = 0.03, I^2 = 47.86%, H^2 = 1.92 0.97 [0.71, 1.32]
Test of $\theta_i = \theta_j$: Q(1) = 1.92, p = 0.17
Test of θ = 0: z = -0.19, p = 0.85
poor
Anderson SE 0.35 [0.17, 0.71] 5.10
Feldman-Winter L 0.32 [0.14, 0.73] 3.81
Shay M 1.08 [0.58, 2.02] 6.08
Xinmei Chen 0.74 [0.52, 1.04] 12.59
Mekonnen T 0.75 [0.67, 0.85] 20.91
Zhou S 0.75 [0.55, 1.02] 13.83
Heterogeneity: τ^2 = 0.03, I^2 = 48.13%, H^2 = 1.93 0.70 [0.56, 0.86]
Test of $\theta_i = \theta_j$: Q(5) = 9.64, p = 0.09
Test of θ = 0: z = -3.32, p = 0.00
Overall 0.74 [0.62, 0.89]
Heterogeneity: τ^2 = 0.04, I^2 = 57.10%, H^2 = 2.33
Test of $\theta_i = \theta_j$: Q(9) = 20.98, p = 0.01
Test of θ = 0: z = -3.23, p = 0.00
Test of group differences: $Q_b(2)$ = 3.63, p = 0.16
1/4
1/2
1
2
Random-effects DerSimonian–Laird model

➢ Stunting

A

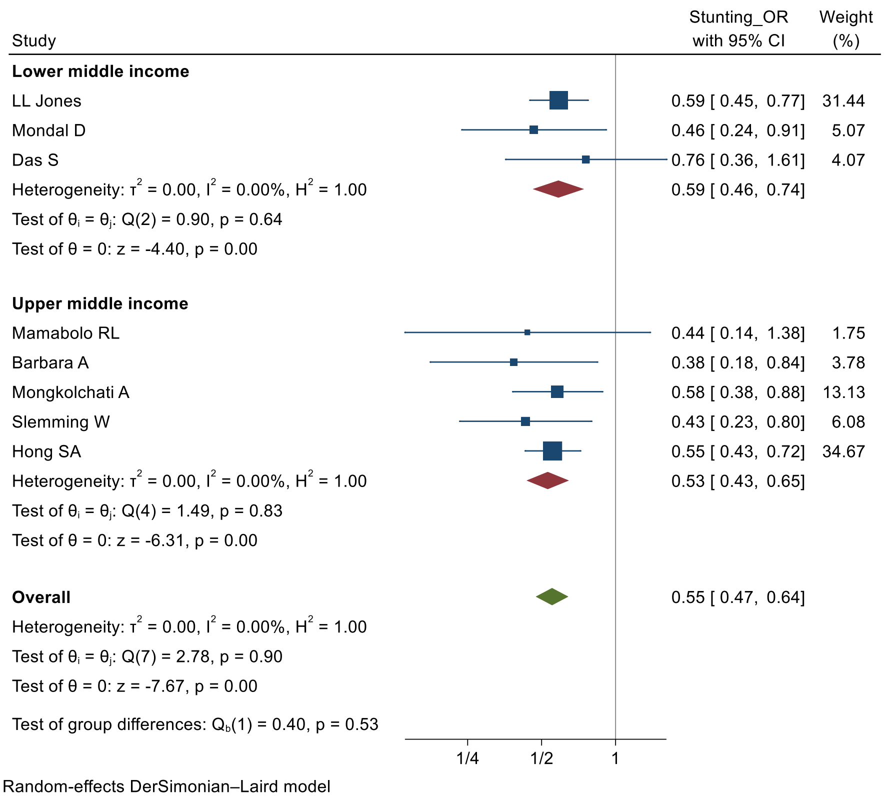

Study
Stunting_OR with 95% CI
Weight (%)
Lower middle income
LL Jones 0.59 [0.45, 0.77] 31.44
Mondal D 0.46 [0.24, 0.91] 5.07
Das S 0.76 [0.36, 1.61] 4.07
Heterogeneity: τ^2 = 0.00, I^2 = 0.00%, H^2 = 1.00 0.59 [0.46, 0.74]
Test of $\theta_i = \theta_j$: Q(2) = 0.90, p = 0.64
Test of θ = 0: z = -4.40, p = 0.00
Upper middle income
Mamabolo RL 0.44 [0.14, 1.38] 1.75
Barbara A 0.38 [0.18, 0.84] 3.78
Mongkolchati A 0.58 [0.38, 0.88] 13.13
Slemming W 0.43 [0.23, 0.80] 6.08
Hong SA 0.55 [0.43, 0.72] 34.67
Heterogeneity: τ^2 = 0.00, I^2 = 0.00%, H^2 = 1.00 0.53 [0.43, 0.65]
Test of $\theta_i = \theta_j$: Q(4) = 1.49, p = 0.83
Test of θ = 0: z = -6.31, p = 0.00
Overall 0.55 [0.47, 0.64]
Heterogeneity: τ^2 = 0.00, I^2 = 0.00%, H^2 = 1.00
Test of $\theta_i = \theta_j$: Q(7) = 2.78, p = 0.90
Test of θ = 0: z = -7.67, p = 0.00
Test of group differences: $Q_b(1)$ = 0.40, p = 0.53
1/4
1/2
1
Random-effects DerSimonian–Laird model

B

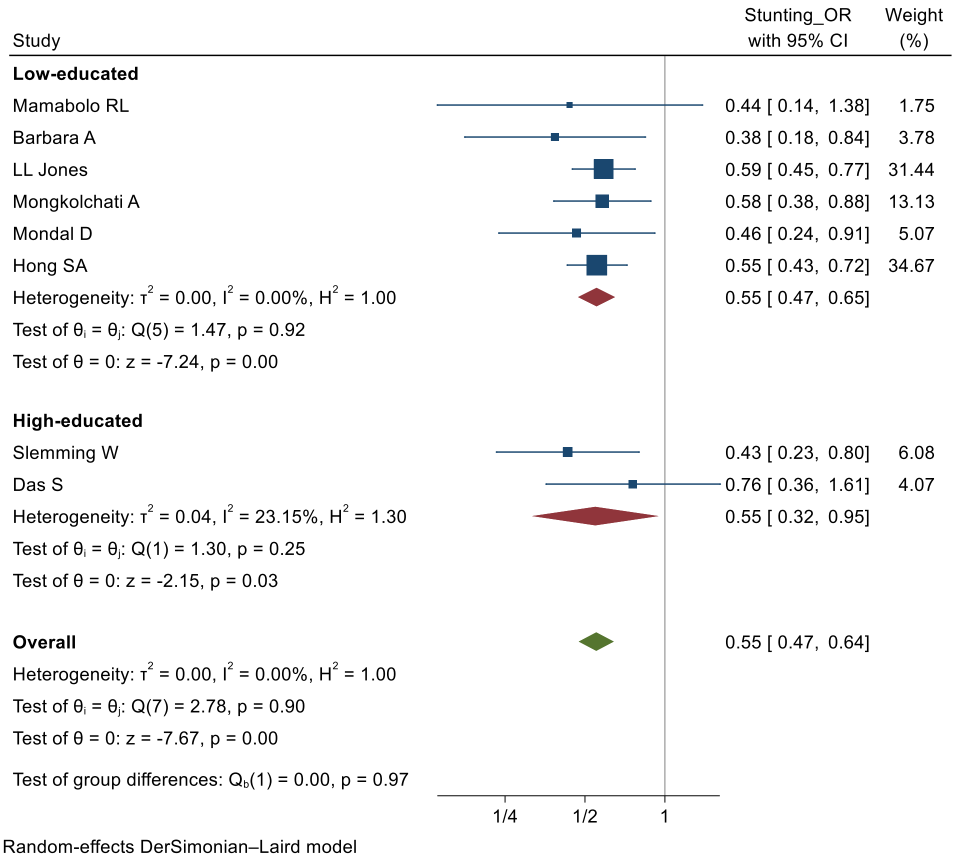

Study
Stunting_OR with 95% CI
Weight (%)
Low-educated
Mamabolo RL 0.44 [0.14, 1.38] 1.75
Barbara A 0.38 [0.18, 0.84] 3.78
LL Jones 0.59 [0.45, 0.77] 31.44
Mongkolchati A 0.58 [0.38, 0.88] 13.13
Mondal D 0.46 [0.24, 0.91] 5.07
Hong SA 0.55 [0.43, 0.72] 34.67
Heterogeneity: τ^2 = 0.00, I^2 = 0.00%, H^2 = 1.00 0.55 [0.47, 0.65]
Test of $\theta_i = \theta_j$: Q(5) = 1.47, p = 0.92
Test of θ = 0: z = -7.24, p = 0.00
High-educated
Slemming W 0.43 [0.23, 0.80] 6.08
Das S 0.76 [0.36, 1.61] 4.07
Heterogeneity: τ^2 = 0.04, I^2 = 23.15%, H^2 = 1.30 0.55 [0.32, 0.95]
Test of $\theta_i = \theta_j$: Q(1) = 1.30, p = 0.25
Test of θ = 0: z = -2.15, p = 0.03
Overall 0.55 [0.47, 0.64]
Heterogeneity: τ^2 = 0.00, I^2 = 0.00%, H^2 = 1.00
Test of $\theta_i = \theta_j$: Q(7) = 2.78, p = 0.90
Test of θ = 0: z = -7.67, p = 0.00
Test of group differences: $Q_b(1)$ = 0.00, p = 0.97
1/4
1/2
1
Random-effects DerSimonian–Laird model

C

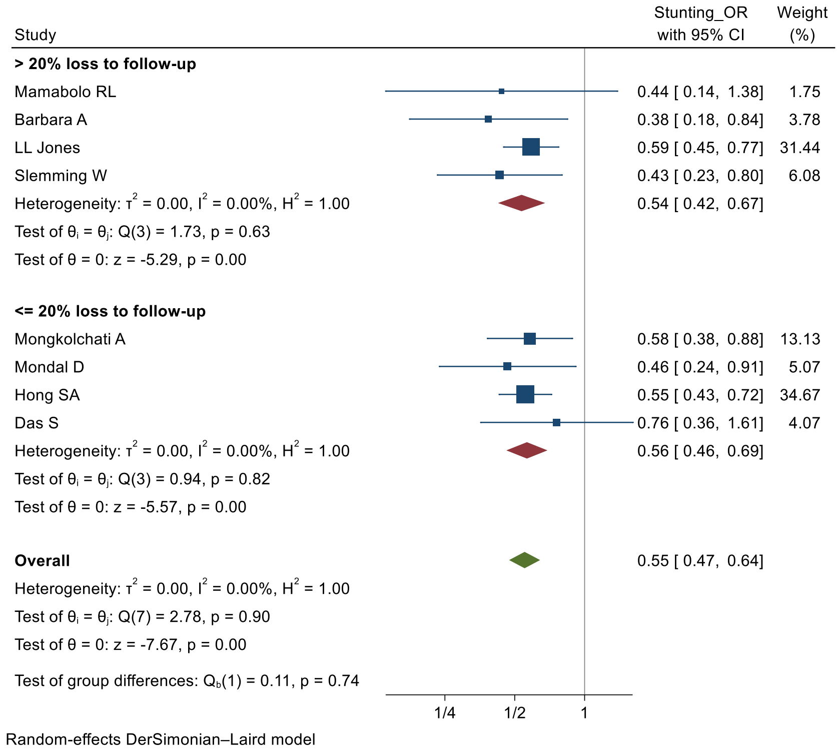
Study
Stunting_OR with 95% CI
Weight (%)
> 20% loss to follow-up
Mamabolo RL 0.44 [0.14, 1.38] 1.75
Barbara A 0.38 [0.18, 0.84] 3.78
LL Jones 0.59 [0.45, 0.77] 31.44
Slemming W 0.43 [0.23, 0.80] 6.08
Heterogeneity: τ² = 0.00, I² = 0.00%, H² = 1.00 0.54 [0.42, 0.67]
Test of θi = θj: Q(3) = 1.73, p = 0.63
Test of θ = 0: z = -5.29, p = 0.00
<= 20% loss to follow-up
Mongkolchati A 0.58 [0.38, 0.88] 13.13
Mondal D 0.46 [0.24, 0.91] 5.07
Hong SA 0.55 [0.43, 0.72] 34.67
Das S 0.76 [0.36, 1.61] 4.07
Heterogeneity: τ² = 0.00, I² = 0.00%, H² = 1.00 0.56 [0.46, 0.69]
Test of θi = θj: Q(3) = 0.94, p = 0.82
Test of θ = 0: z = -5.57, p = 0.00
Overall 0.55 [0.47, 0.64]
Heterogeneity: τ² = 0.00, I² = 0.00%, H² = 1.00
Test of θi = θj: Q(7) = 2.78, p = 0.90
Test of θ = 0: z = -7.67, p = 0.00
Test of group differences: Qb(1) = 0.11, p = 0.74
1/4
1/2
1
Random-effects DerSimonian–Laird model

D

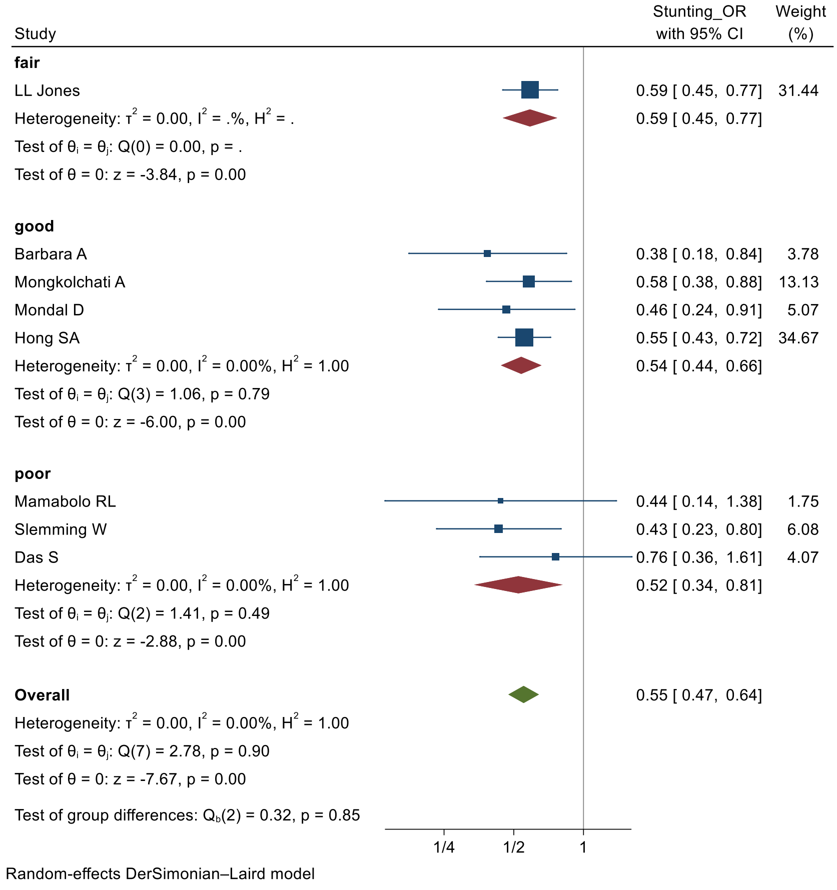
Study
Stunting_OR with 95% CI
Weight (%)
fair
LL Jones 0.59 [0.45, 0.77] 31.44
Heterogeneity: τ² = 0.00, I² = .%, H² = . 0.59 [0.45, 0.77]
Test of θi = θj: Q(0) = 0.00, p = .
Test of θ = 0: z = -3.84, p = 0.00
good
Barbara A 0.38 [0.18, 0.84] 3.78
Mongkolchati A 0.58 [0.38, 0.88] 13.13
Mondal D 0.46 [0.24, 0.91] 5.07
Hong SA 0.55 [0.43, 0.72] 34.67
Heterogeneity: τ² = 0.00, I² = 0.00%, H² = 1.00 0.54 [0.44, 0.66]
Test of θi = θj: Q(3) = 1.06, p = 0.79
Test of θ = 0: z = -6.00, p = 0.00
poor
Mamabolo RL 0.44 [0.14, 1.38] 1.75
Slemming W 0.43 [0.23, 0.80] 6.08
Das S 0.76 [0.36, 1.61] 4.07
Heterogeneity: τ² = 0.00, I² = 0.00%, H² = 1.00 0.52 [0.34, 0.81]
Test of θi = θj: Q(2) = 1.41, p = 0.49
Test of θ = 0: z = -2.88, p = 0.00
Overall 0.55 [0.47, 0.64]
Heterogeneity: τ² = 0.00, I² = 0.00%, H² = 1.00
Test of θi = θj: Q(7) = 2.78, p = 0.90
Test of θ = 0: z = -7.67, p = 0.00
Test of group differences: Qb(2) = 0.32, p = 0.85
1/4
1/2
1
Random-effects DerSimonian–Laird model
