# Supplementary material for: Maternal education and its influence on child growth and nutritional status during the first two years of life: a systematic review and meta-analysis
Source: eClinicalMedicine. 2024 Apr 4;71:102574. doi: 10.1016/j.eclinm.2024.102574 (PMC11001623; doi:10.1016/j.eclinm.2024.102574)

Supplementary 7. Leave-one-out sensitivity analysis results.

- **WAZ**

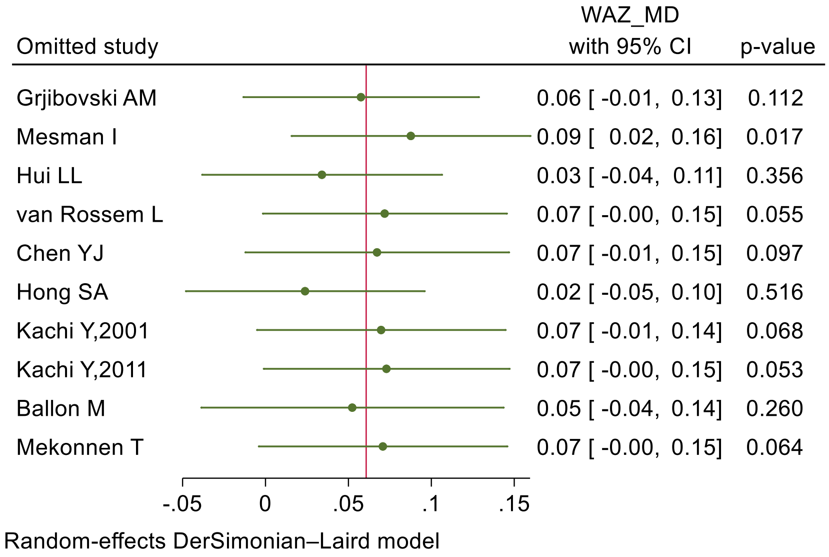

- **HAZ**

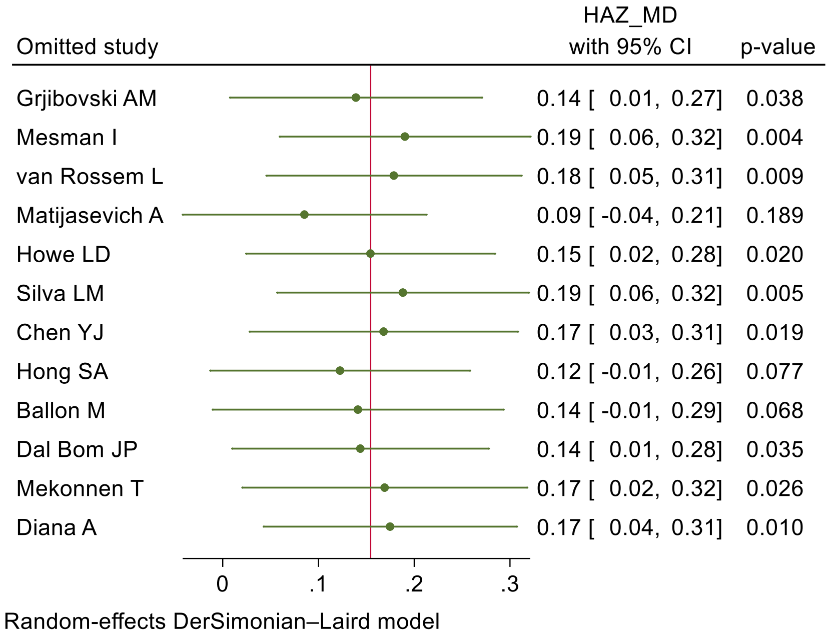

- **BMI z-score**

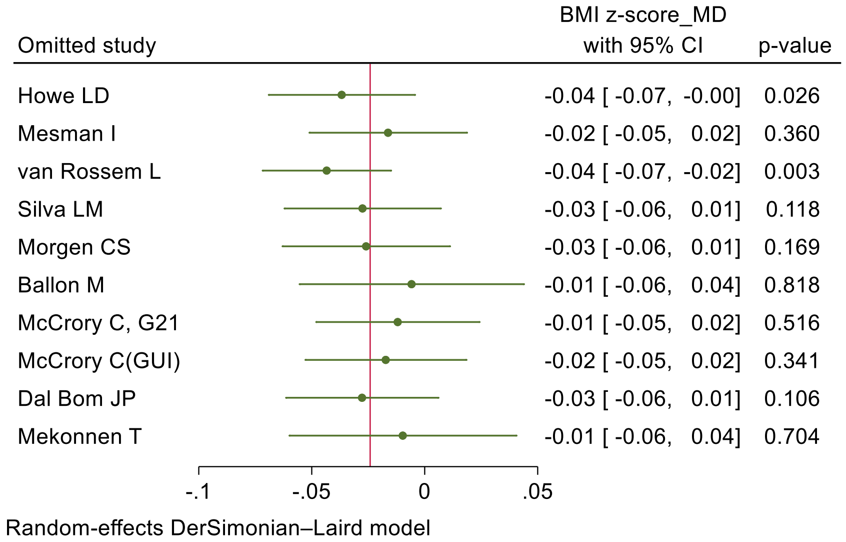

- **Overweight**

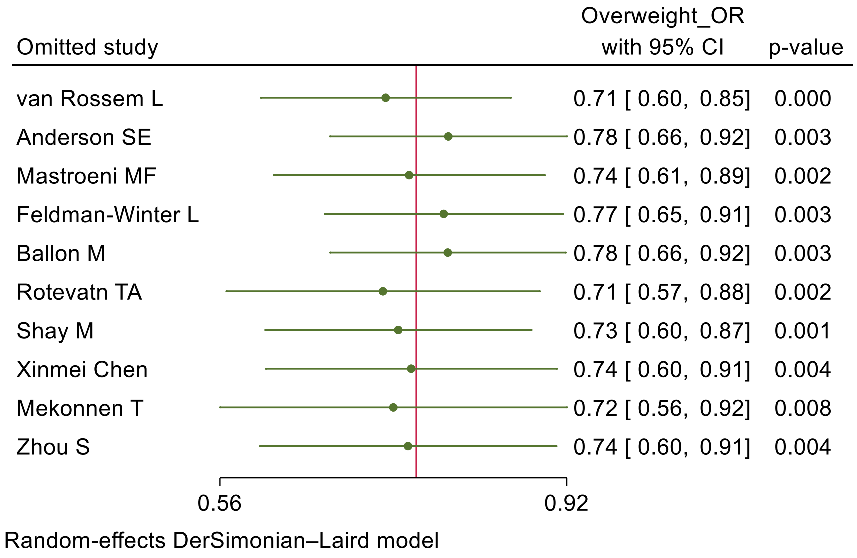

- **Underweight**

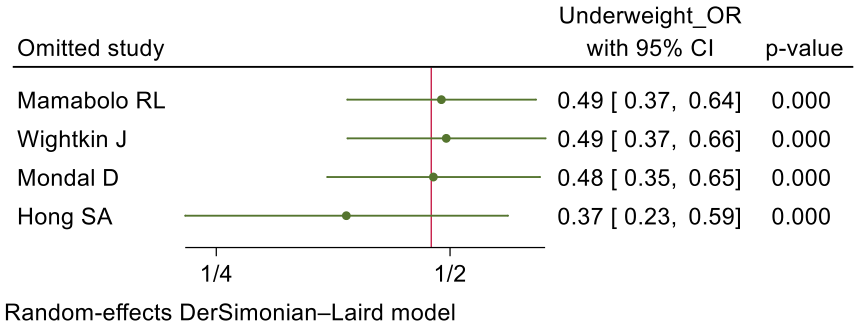

➢ **Stunting**

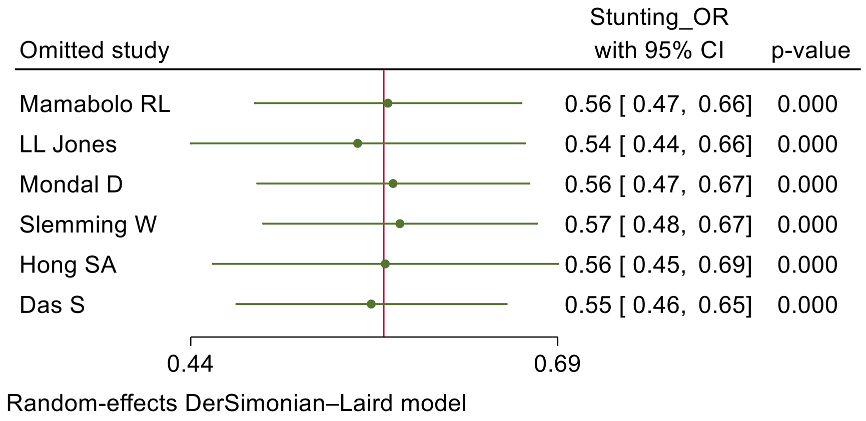

➢ **Rapid weight gain**

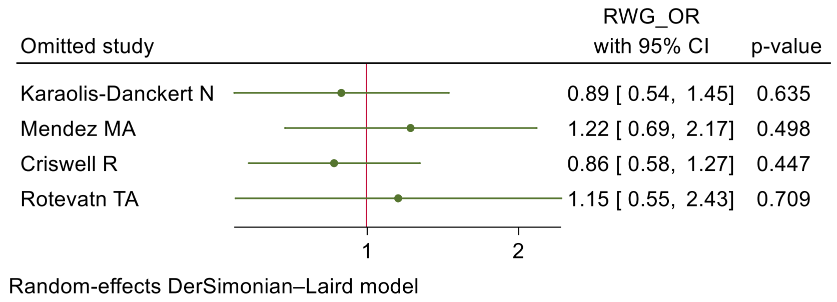

Supplement: Supplementary File 7 [file mmc7.pdf]
