# Supplementary material for: Maternal education and its influence on child growth and nutritional status during the first two years of life: a systematic review and meta-analysis
Source: eClinicalMedicine. 2024 Apr 4;71:102574. doi: 10.1016/j.eclinm.2024.102574 (PMC11001623; doi:10.1016/j.eclinm.2024.102574)
Supplement: Supplementary File 8 [file mmc8.pdf]

Supplementary 8. Publication bias.

- **WAZ**

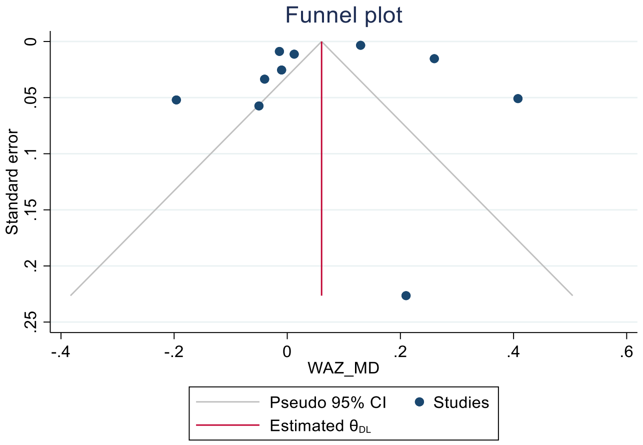

test	*p-value*
Begg	0.7205
Egger	0.8351

STUDIES	WAZ(MD)	95% CI
**OBSERVED**	0.061	-0.009 to 0.131
**OBSERVED + IMPUTED**	0.061	-0.009 to 0.131

- **HAZ**

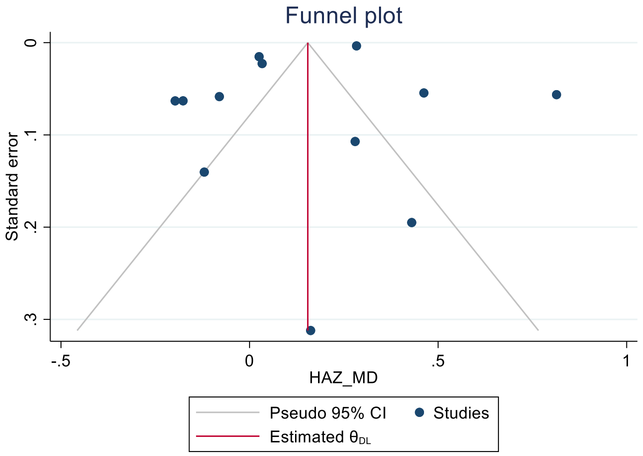

test	*p-value*
Begg	0.8370
Egger	0.8606

STUDIES	HAZ(MD)	95% CI
**OBSERVED**	0.154	0.027 to 0.282
**OBSERVED + IMPUTED**	0.154	0.027 to 0.282

➢ BMI z-score

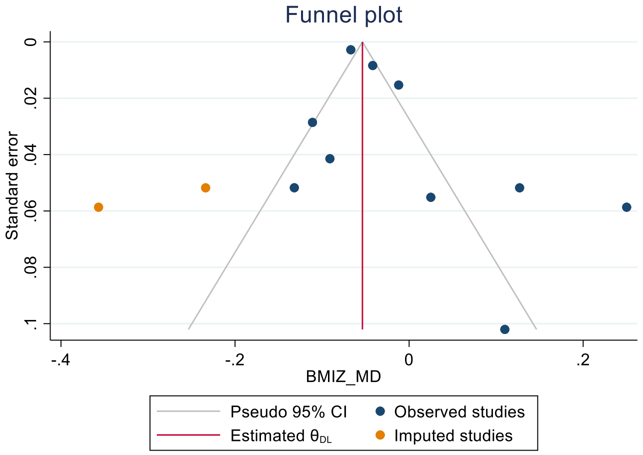

test	*p-value*
Begg	0.1524
Egger	0.0077

STUDIES	BMI Z-SCORE (MD)	95% CI
**OBSERVED**	-0.024	-0.058 to 0.009
**OBSERVED + IMPUTED**	-0.054	-0.090 to -0.017

➢ Overweight

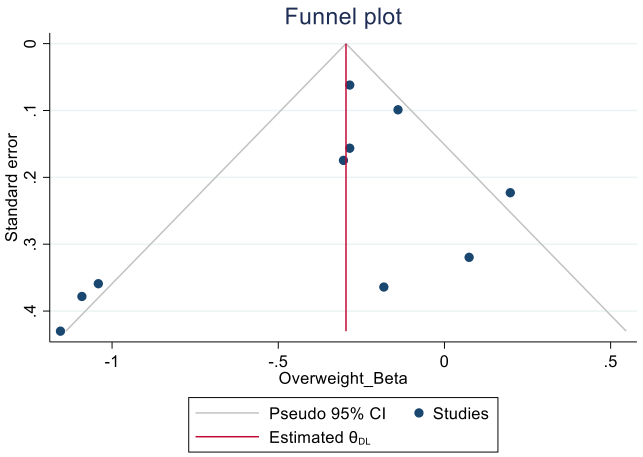

test	*p-value*
Begg	0.2831
Egger	0.0756

STUDIES	OVERWEIGHT(OR)	95% CI
**OBSERVED**	0.744	0.621 to 0.890
**OBSERVED + IMPUTED**	0.744	0.621 to 0.890

- ## Underweight

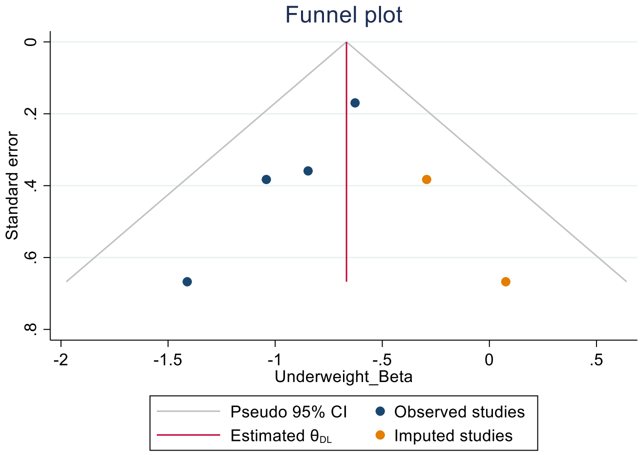

test	*p-value*
Begg	0.0894
Egger	0.1506

STUDIES	UNDERWEIGHT(OR)	95% CI
**OBSERVED**	0.473	0.360 to 0.621
**OBSERVED + IMPUTED**	0.513	0.399 to 0.660

- ## Stunting

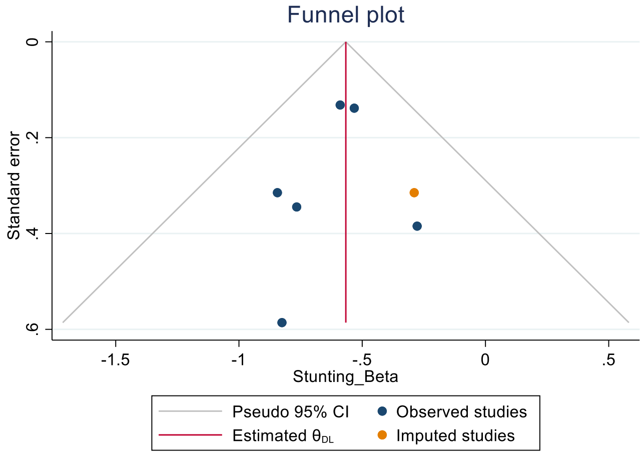

test	*p-value*
Begg	1
Egger	0.6337

STUDIES	STUNTING(OR)	95% CI
**OBSERVED**	0.556	0.471 to 0.657
**OBSERVED + IMPUTED**	0.568	0.483 to 0.667

- **Rapid weight gain**

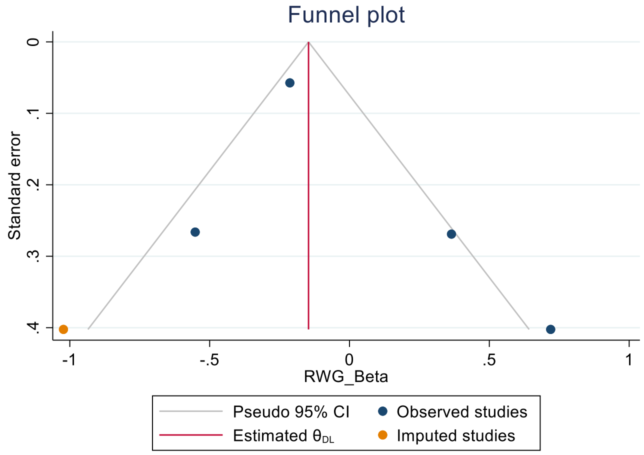

test	*p-value*
Begg	0.0894
Egger	0.3305

STUDIES	RWG(OR)	95% CI
**OBSERVED**	0.997	0.648 to 1.534
**OBSERVED + IMPUTED**	0.864	0.565 to 1.320
